# Decoding the Role of Isolated Ga^+^ in PdGa@MFI Catalyst Promoting a Direct CO_2_ Hydrogenation Path to DME

**DOI:** 10.1021/jacs.5c20643

**Published:** 2026-02-09

**Authors:** Minjie Zhao, Daviel Gómez, Vlad Martin-Diaconescu, Laura Simonelli, Miguel Lopez-Haro, Jose Juan Calvino, Avelino Corma, Patricia Concepción

**Affiliations:** † Instituto de Tecnología Química, 16379 Universitat Politècnica de València-Consejo Superior de Investigaciones Científicas (UPV-CSIC), Avenida de los Naranjos s/n, Valencia 46022, Spain; ‡ CELLS—ALBA Synchrotron Radiation Facility, Carrer de la Llum 2-26, Cerdanyola del Vallès 08290, Spain; § Departamento de Ciencia de los Materiales e Ingeniería Metalúrgica y Química Inorgánica. Facultad Ciencias, Universidad de Cádiz. Campus Rio San Pedro. Puerto Real, Cádiz 11510, Spain

## Abstract

This work presents a strategy to control not only the distance and proximity of active sites at the atomic or nanoscale but also the nature of sites in zeolite-based catalysts, promoting the coupling rate of surface intermediate species and accordingly the formation rate of dimethyl ether (DME) by a direct CO_2_ hydrogenation path. We use a one-pot synthesis strategy and a thermal-induced detachment process of framework elements, such as Ga^3+^ ions, to stabilize PdGa alloys and Ga^+^ sites in close proximity to Brønsted acid sites under reductive conditions. Using this strategy, a production of oxygenates of up to 42,864 g_MeOH+DME_·kg_Pd_^–1^·h^–1^ at 45 bar, 260 °C, and WHSV = 15,000 mL·g_cat_^–1^·h^–1^, is obtained with 80% selectivity to oxygenated (19% methanol/61% DME), outperforming the most active Pd-based CO_2_ hydrogenation catalysts in the literature. Time-resolved kinetic studies, in situ X-ray adsorption, and in situ and operando IR offer strong proof of the key role of isolated Ga^+^ Lewis acid sites in close proximity to Brønsted acid sites in stabilizing monoformate intermediate species and facilitating the direct production of DME. Finally, this work highlights the key role of the zeolite in metal confinement, conferring excellent stability, oxidation resistance, hydrophilicity, and close proximity of active sites together with the stabilization of low-coordinated Lewis acid sites.

## Graphical abstract

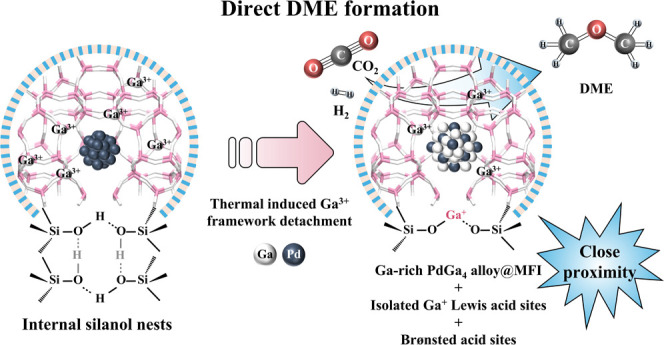

## Introduction

CO_2_ capture and utilization constitute one of the pillars in the energetic transition, targeting net zero CO_2_ emission for 2050. In the context of a circular economy, the thermocatalytic CO_2_ reduction to produce high-value chemicals is of clear interest. In this direction, attention has been paid to the production of methanol and dimethyl ether (DME) from CO_2_, being DME preferred to methanol due to its higher energy density and its potential as an environmentally friendly ultra clean fuel, as well as a building block for high-value added chemicals like acetic acid, ethanol, and methyl acetate.^1–3^ CO_2_ to DME is usually formed in a two-step process where CO_2_ is first hydrogenated to methanol in one reactor and further converted to DME in a second reactor. Combining the two catalytic processes in the same reactor by a tandem catalytic approach with a bifunctional catalyst offers economic, kinetic, and thermodynamic advantages with respect to the conventional process involving two reactors.^3,4^ In this direction, the synergistic cooperation of two catalytic functions, one for methanol synthesis and an acid site for methanol dehydration to DME is key for improved catalytic performance. Most of the catalytic systems studied in the literature are physically mixed hybrid catalysts,^1^ displaying maxima DME production of around 500 g_DME_·kg_cat_^–1^·h^–1^ as it is the case of a CuZnAl/zeolite tandem catalytic system operating at 20–60 bar and 180–300 °C.^5,6^ In the last years, new catalyst formulations based on novel synthesis strategies have appeared in which the contact between the two functionalities is maximized at the microscopic level. For instance, core@shell structures and the immobilization of CuO and ZnO nanoparticles onto an acid support^3,4,7^ result in higher DME selectivity than over conventional hybrid systems, though it still requires significant improvement. The critical point in all these studies is to control the contact and distance between the active phases, which has a strong influence on the reactivity of surface intermediates, mass transfer phenomena, and the coupling between the individual reactions in order to shift the thermodynamic equilibrium. With this in mind, we believe that the design of a better performing catalyst will require control of the nature and the distance between the active sites. If this is achieved, it should be possible not only to promote the coupling of two individual reactions but also to favor the direct methoxy-DME versus the standard methoxy-methanol-DME path (where methanol is desorbed and readsorbed on an adjacent acid site), with the corresponding thermodynamic and kinetic benefit for the one step, i.e., direct, CO_2_ hydrogenation to DME.^1^ To our knowledge, such a direct non-methanol intermediate route has only been reported for GaN catalysts but with the drawback of operating at high temperature (360 °C) and pressure (20 bar), resulting in high CO selectivity (60%).^8^

Zeolites provide an ideal platform to control the distance between active sites, allowing the stabilization of Lewis sites with tunable local environments as well as the stabilization of metal nanoparticles encapsulated within the zeolite channels in close proximity to non-acidic silanols or to Brønsted acid sites.^9–11^ Normally, copper (Cu) or palladium (Pd) are active metals for CO_2_ hydrogenation to methanol.^12–14^ In the last case, while Pd metal is less selective to methanol, promoting CO formation via the reverse water–gas shift reaction (RWGS), Pd-based alloys, and in particular PdGa alloys, are promising candidates in CO_2_ hydrogenation, suppressing CO formation and resulting in ∼50–80% methanol selectivity at 220–300 °C.^15–17^ This has been attributed to the unique electronic properties of Pd in the PdGa alloys. Besides the role of the metallic component, Copéret et al. disclosed that isolated tetrahedral Ga­(III) Lewis species generated on a PdGa@SiO_2_ catalyst under reaction conditions due to a dynamic dealloying/realloying process play a key role in the stabilization of methoxy species and accordingly in methanol production.^18^ Other authors postulate a synergetic effect between PdGa and Ga_2_O_3_, where CO_2_ intermediate species are stabilized on Lewis sites of the metal oxide, with H_2_ being dissociated on the metallic component.^16^

The objective of the present work is to synthesize DME from CO_2_ in a one-step direct reaction path using a zeolite with Ga^3+^ in framework positions of a MFI zeolite where small PdGa particles are confined within the micropores. This strategy allows us to control at the nanoscale the distance between two functionalities, metal and acid sites, promoting a tandem reaction route. Moreover, it offers the unique opportunity to regulate the nature of active sites by controlling the migration of Ga^3+^ species from framework positions to extra-framework with the stabilization of isolated Ga^+^ Lewis acid sites and partial alloying of Ga with Pd. In this way, by stabilizing the adequate sites (PdGa alloys, Ga^+^ Lewis sites, and Brønsted acid sites) in a confined space, it is possible to drive the reaction through a direct CO_2_ to DME hydrogenation path, maximizing DME production beyond the reported values for Pd-based catalysts. Following the methodology presented here, we report a production of oxygenates of up to 25,659 g_MeOH+DME_·kg_Pd_^–1^·h^–1^ at 20 bar, 260 °C, and WHSV = 15,000 mL·g_cat_^–1^·h^–1^, with 75% selectivity to oxygenated (18% methanol/57% DME), outperforming the most active catalysts in the literature.^16^

In general, this work provides fundamental insights into the possibility to direct the CO_2_ to DME reaction path through a direct reaction mechanism by controlling not only the distance between active sites but also the nature of active sites in zeolite-based materials. In addition, we show the importance of several parameters, such as active site confinement for enhancing catalyst stability and resistance to oxidation, hydrophilicity–hydrophobicity, and low-coordinated Lewis acid sites, resulting in better-performing catalysts for CO_2_ direct transformation into DME.

## Results and Discussion

### Synthesis of PdGa Alloy Compounds in a Host MFI Zeolite

Pd nanoparticles of ∼2 nm size have been successfully confined inside MFI zeolite using a modified one-pot hydrothermal synthesis strategy to anchor Pd clusters inside the channels of Ga-MFI (Figure S1), followed by thermally induced framework Ga^3+^ migration to extra-framework isolated Ga^+^ species and partial alloyed Pd–Ga species. The use of common literature reported complexing ligands such as amino (ethylenediamine),^19^ mercapto [(3-mercaptopropyl)­trimethoxysilane][Bibr ref20] are not applicable for Pd confinement inside the Ga-MFI, as shown in Figures S2–S4, where Pd is partially precipitated during hydrothermal synthesis on the MFI surface, resulting in large Pd particles with sizes from 10 to 50 nm. On the contrary, we have found that the use of [3-(2-aminoethylamino)­propyl]­trimethoxysilane (APTMS) as a ligand to complex with Pd­(NH_3_)_4_Cl_2_ is an efficient approach to confine Pd clusters inside the Ga-MFI channels (Figures S5–S7). As illustrated in Figure S2, APTMS is a bifunctional ligand where the amino groups (–NH_2_) bind strongly with Pd avoiding the formation and precipitation of Pd­(OH)_2_ at high pH conditions, while the alkoxysilane moiety of the APTMS ligand undergoes hydrolysis in alkaline media to form covalent Si–O–Si bonds and create linkages that enforce encapsulation of Pd clusters inside the channel of MFI zeolite during the crystallization process. For the synthesis of PdGa@MFI samples, Pd­(NH_3_)_4_Cl_2_ is first reacted with APTMS and then added into the synthesis gel containing the desired amount of Ga­(NO_3_)_3_, allowing the simultaneous incorporation of Ga in the zeolite framework (as Ga^3+^) and the Pd clusters inside the zeolite channel during the crystallization process (Figure S5). In this study, PdGa@MFI samples were prepared, keeping a constant 0.6 wt % Pd loading while changing the Ga loading from 0 to 1.6 wt % (Table S1). The maximum Ga loading achieved in this synthesis method is 1.6 wt % with a Si/Ga = 74 molar ratio and a Ga/Pd atomic ratio of 4/1. Under these conditions, ^17^Ga-NMR indicates that all Ga is located in the framework of the zeolite (Figure S9). To force partial migration of the framework Ga^3+^ and its alloying with Pd, direct H_2_ reduction at high temperature (≥500 °C) was performed, and different reduction temperatures (500, 600, 700, and 800 °C) were explored. The complete removal of the organic precursor during the reduction process is confirmed by elemental analysis, indicating nearly no C and N after reduction (Table S1). The samples are labeled as PdGa_*x*_@MFI-AS for the as-prepared samples before thermal treatment, where x represents the molar Ga/Pd ratio in the synthesis gel. PdGa_*x*_@MFI-CAL refer to the calcined sample and PdGa_*x*_@MFI-T-RED to the reduced one, being T the reduction temperature. The effect of the reduction temperature on the structural, morphological, and chemical properties of the final material was analyzed by IR-pyridine, XRD, CO chemisorption, IR-CO, and high-resolution high-angle annular dark-field scanning transmission electron microscopy (HR-HAADF-STEM) combined with integrated differential phase contrast (iDPC) imaging (see Supporting Information, Section S4). From this study, 700 °C is defined as the optima temperature to promote the partial migration of framework Ga^3+^ to form PdGa alloy while simultaneously stabilizing isolated Ga^+^ species on surrounding silanol nests. Accordingly, this temperature is chosen as the default reduction temperature for investigating the catalytic performance of samples with different Ga/Pd atomic ratios.

### Influence of the Ga/Pd Ratio in PdGa_*x*_@MFI-700RED Samples

In order to modify the relative proportion of the different Pd and Ga species that will be later correlated with the potential active sites, samples with different Ga loadings, corresponding to theoretical Ga/Pd molar ratios of 0, 0.5, 1, 2, and 4 were studied. In all samples, the reduction temperature was 700 °C. The structure of the PdGa_*x*_@MFI-700RED samples was characterized by STEM, including HR-HAADF-STEM and iDPC imaging. STEM images show in all cases a good metal dispersion, with a particle size of ∼2.2 nm in all samples (Figures 1 and S49–S57). Based on paired HR-HAADF-STEM and iDPC imaging, Pd-containing nanoparticles were shown to be located in the channel of the MFI structure (details in Section S10). Further catalytic investigations using phenylacetylene and diphenylacetylene hydrogenation as test reactions have verified the location of Pd inside the zeolite channels (Figure S8).

**Figure 1:**
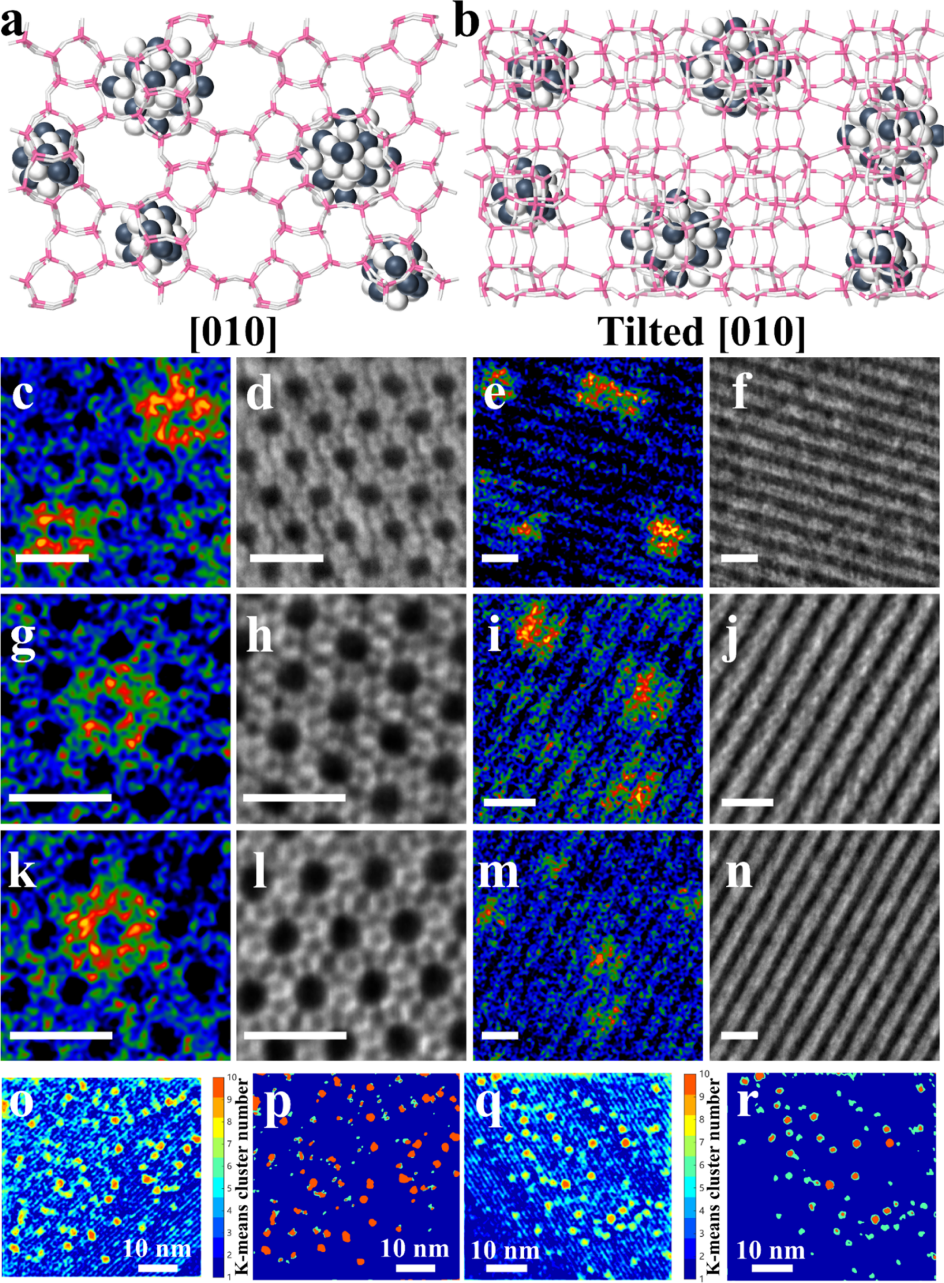
Identification of the location of PdGa alloy inside MFI zeolite. (a,b) MFI zeolite framework structure in [010] and tilted [010] orientations. (c–f) PdGa_1_@MFI-700RED, (g–j) PdGa_2_@MFI-700RED, and (k–n) PdGa_4_@MFI-700RED, each showing HR-HAADF-STEM and corresponding iDPC images, acquired from both [010] and tilted [010] views (scale bar: 2 nm). K-means clustering analysis was applied to HR-HAADF-STEM images of PdGa_1_@MFI-700RED (o,p) and PdGa_4_@MFI-700RED (q,r) to map the distribution of Pd/PdGa (red) and Ga (cyan) species. The clustering, based on Z-contrast differences, resulted in (o,q) clustered HR-HAADF-STEM images and (p,r) corresponding distribution maps.

The distribution of Pd and Ga in the zeolite crystallites was shown to be homogeneous according to energy-dispersive X-ray mapping (Figures S34 and S35 , S38 and S39, S52 and S53, S56 and S57). The K-means clustering analysis reveals that Pd or PdGa are confined inside PdGa_1_@MFI-700RED (Figure 1o,p), while Ga species are closely surrounded by Pd atoms (favoring Pd–Ga interaction) in PdGa_4_@MFI-700RED sample (Figure 1q,r).

The X-ray adsorption (XAS) analysis performed at the Ga K-edge on the PdGa_0.5_@MFI-700RED and PdGa_4_@MFI-700RED samples shows a significant impact of the Ga/Pd atomic ratio on the nature of Ga species. In Figure 2a, the X-ray absorption near edge structure (XANES) collected at the Ga K-edge on ex situ reduced PdGa_0.5_@MFI-700RED and PdGa_4_@MFI-700RED samples are reported and compared with the spectra corresponding to the Ga_4_@MFI-700RED, Ga_2_O_3_, and Ga­(acac)_3_ references. The spectra obtained under in situ H_2_ reduction and CO_2_/H_2_ reaction conditions are reported in Figure 2c–h. All the XANES spectra available from the different data sets were subjected to multivariate curve resolution-alternating least-squares (MCR-ALS) unmixing (details in Supporting Information, Section S7 and Figure S89). Five components were determined to fit the complexity of the spectra and conform the best model, as shown in Figure 2b. They have been found to correspond to tetrahedral Ga^3+^ species as in the Ga_4_@MFI-700RED pristine material (A), PdGa alloy (B), Ga^+^ reduced species (C), octahedral Ga­(III) oxide phase (D), and a reduced Ga species, compatible with a disordered Ga-OPd/Ga cluster (E).^18,21–23^ Regarding the Ga^+^ species assignation reported here, this could be questionable. Indeed, it has been demonstrated that changes in the identity and number of gallium nearest neighbors can give rise to changes in XANES spectra similar to those attributed in literature to changes in oxidation state.^22^ The comparison of the XAS and the below reported IR results allowed discrimination between the different possible scenarios, supporting the assignation of the MCR component C to Ga^+^ reduced species. According to MCR-ALS analysis, the ex situ reduced PdGa_4_@MFI-700RED sample contains around 76% of component A (tetrahedral Ga^3+^), 11% of component C (Ga^+^), 9% of component B (PdGa alloy), and 4% of component D (octahedral Ga­(III) oxide phase) (see Figure 2c). Very differently, ex situ reduced PdGa_0.5_@MFI-700RED sample, shows around 32% of component A (tetrahedral Ga^3+^), 8% of component C (Ga^+^), 23% of component B (PdGa alloy), 22% of component D (octahedral Ga­(III) oxide phase), and 15% of component E (see Figure 2d). Under in situ H_2_ reduction from 25 to 350 °C, in both systems, component A (tetrahedral Ga^3+^) decreases in favor of component B (PdGa), but only in the PdGa_4_@MFI-700RED sample, the component C (Ga^+^), which is already present in the ex situ reduced sample, increases (Figure 2c, compared to 2d). The exposure to reaction conditions in a CO_2_/H_2_ flow (1:3), 10 bar, and 260 °C, does not change markedly the component fraction in both samples.

**Figure 2:**
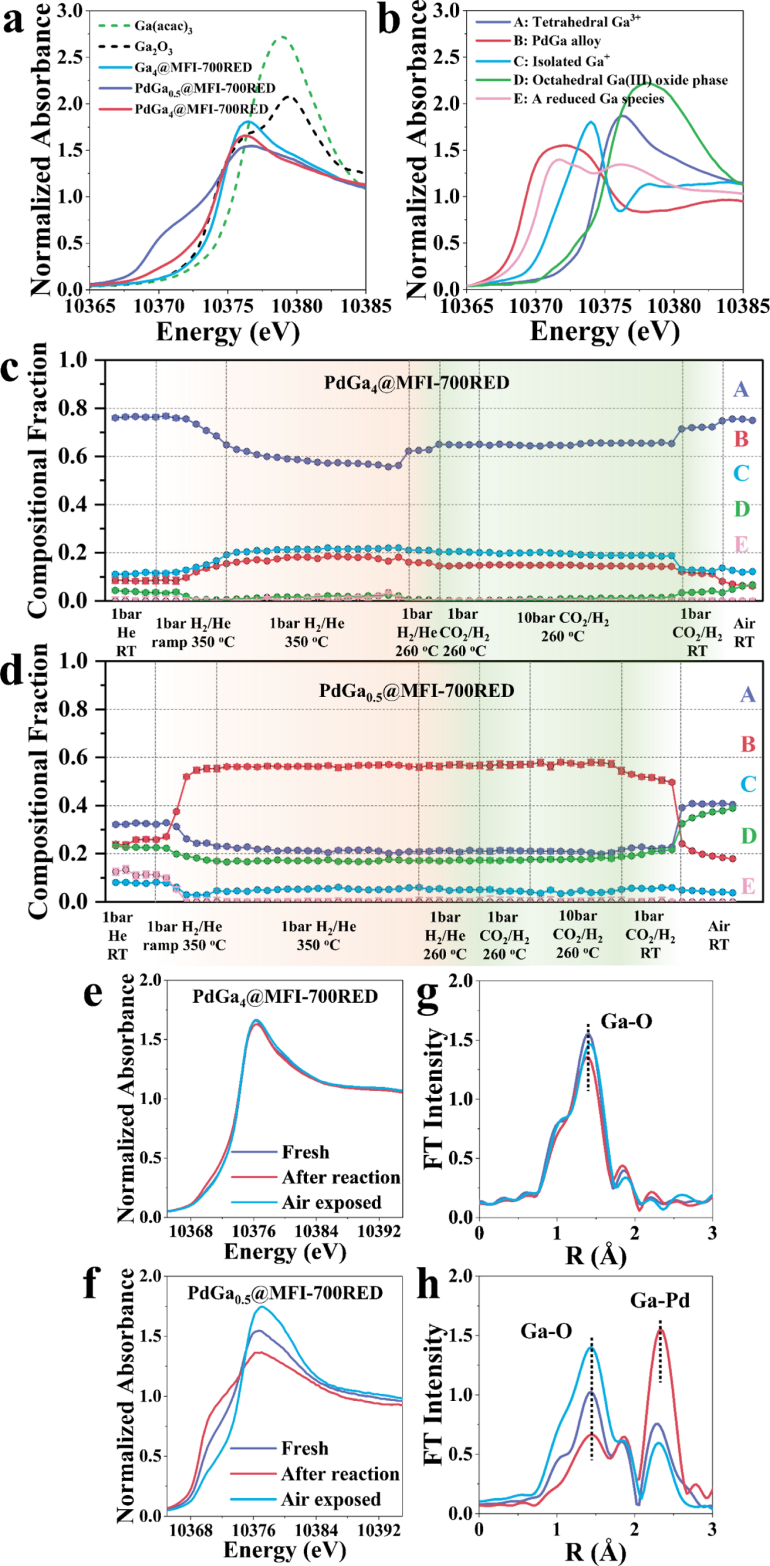
MCR-ALS analysis on PdGa_4_@MFI-700RED and PdGa_0.5_@MFI-700RED samples and MCR obtained components dynamic evolution under in situ H_2_ reduction and CO_2_/H_2_ reaction conditions. (a) Ga K-edge XANES spectrum of PdGa_4_@MFI-700RED, PdGa_0.5_@MFI-700RED, and Ga_4_@MFI-700RED systems and compared with the Ga_2_O_3_ and Ga­(acac)_3_ references. (b) The 5 MCR components, which allow to deconvolute all the data sets. A = tetrahedral Ga^3+^, B = PdGa alloy, C = isolated Ga^+^, D = octahedral Ga­(III) oxide phase, and E = a reduced Ga species, compatible with a disordered Ga-OPd/Ga cluster. Quantitative evolution of the MCR components for (c) PdGa_4_@MFI-700RED and (d) PdGa_0.5_@MFI-700RED under operando conditions. (e,f) Key XANES spectra representing the different conditions (ex situ reduced, labeled as fresh, after H_2_ reduction and then reaction, and exposed to air after reaction) and (g,h) corresponding Fourier transforms of the *k* ^2^ weighted EXAFS oscillations. Notice that exposure to air after reaction allow the PdGa_4_@MFI-700RED catalyst to recover the fresh state highlighting the reversibility of the process, while the PdGa_0.5_@MFI-700RED is not completely reversible, presenting an increase of the oxidized component D (octahedral Ga­(III) oxide phase) and tetrahedral Ga^3+^ at the expenses of component B (PdGa alloy) respect to the starting material.

The Fourier transforms of the Ga K-edge extended X-ray absorption fine structure for the ex situ reduced PdGa_4_@MFI-700RED and PdGa_0.5_@MFI-700RED systems, after in situ H_2_ reduction and further reaction, and exposed to air after reaction are shown in Figure 2e–h, respectively. For both systems, the contribution at around 1.7 Å corresponds to the Ga–O bond, as expected for the Ga-MFI structure. For the PdGa_0.5_@MFI-700RED sample, the additional contribution visible at around 2.3 Å is assigned to the Pd–Ga bond, confirming the formation of the PdGa alloy, which increases after in situ H_2_ reduction and stays constant under reaction conditions, while it decreases after air exposure. However, in the PdGa_4_@MFI-700RED system (Figure 2g), the Pd–Ga bond formation is not detectable by Ga K-edge EXAFS because of its relatively small amount compared to the total Ga and its highly disordered nature, while it is detectable by MCR over the Ga K-edge XANES region (around 9% of the total Ga species in the PdGa_4_@MFI-700RED vs 23% for the PdGa_0.5_@MFI-700RED system). In addition, Pd–Ga and Pd–Pd contributions can be seen in the Pd K-edge collected on the same system (Figure S90), confirming the presence of PdGa alloy. The reported EXAFS spectra have been fitted in the 1–3 Å range (details in Supporting Information, Section S7, Figures S90–S93, Tables S16 and S17), where the obtained Pd–Ga coordination number agrees in the error bar with the optically characterized particle size and suggest larger PdGa particles size corresponding to a higher Pd/Ga molar ratio.

From the XAS data, it is interesting to remark on the higher fraction of isolated Ga^+^ species in the PdGa_4_@MFI-700RED sample, compared to that of the PdGa_0.5_@MFI-700RED sample. This can be related to the different Brønsted acidity and silanol nest density of the samples prior to reduction, affecting the redistribution of Ga species during the migratory phase (see Figures S14 and S41).

The existence of isolated Ga^+^ Lewis sites and PdGa alloyed species in the different samples is supported by IR-CO spectroscopy. IR of CO as probe molecule, gives interesting information about the existence of Pd species in different surface local configurations, i.e., as diluted sites in PdGa alloys or as aggregated species in Pd domains, which can be determined from the relative intensity of linear coordinated CO (IR band at 2095 cm^–1^) and bridge or tricoordinated CO species (IR band at 1961 and 1912 cm^–1^),^16^ respectively. The IR spectra in Figures S47–S48 show, in all Ga-containing samples, a clear decrease in the intensity of the IR bands associated with CO coordinated on multiple Pd sites, indicating that Ga addition promotes the formation of alloyed species where Pd becomes diluted on the surface, favoring linear CO coordination (Figure S48). Moreover, the lack of bridge CO IR bands in the PdGa_4_@MFI-700RED sample, at least at the detection limit of the technique, suggests that there are no surface Pd domains present, which, on the contrary, coexist on the other samples. This is in line with the CO chemisorption analysis, displaying a decrease in the CO adsorption capacity at increasing the Ga loading in the sample (Table S6). In addition, H_2_–D_2_ isotopic exchange studies (Table S7 and Figure S63) show a gradual increase in the apparent activation energy of HD formation together with a decrease in the rate of HD formation when increasing the Ga loading, inferring a different chemical composition of Pd species depending on the Ga/Pd molar ratio. Furthermore, IR-CO spectra displays in the PdGa_4_@MFI-700RED sample, an IR band at 2185 cm^–1^ (Figure S47f), which according to the literature, can be associated with low-coordinated Ga^+^ sites,^24,25^ which, on the other hand, supports the XANES analysis. The absence of this band in the IR spectra of the PdGa_0.5_@MFI-700RED sample (Figure S47c), while present in the XANES spectra, indicates their existence in a markedly lower proportion. A detailed discussion of the identification and assignation of Ga^+^ species can be found in Section S4.4.

### Catalytic Studies in the CO_2_ Hydrogenation

The catalytic performance in the CO_2_ hydrogenation at 20 bar, CO_2_/H_2_ = 1:3, and space velocity WHSV = 15,000 mL·g_cat_^–1^·h^–1^ is summarized in Tables S8 and S9 and graphically displayed in Figures 3a, S64 and S65. Pd@MFI and PdGa_0.5_@MFI-700RED show very low activity and selectivity to oxygenates in the 220–300 °C temperature range, with CO as the predominant product, while both the CO_2_ conversion and the selectivity to oxygenates (MeOH + DME) increase by increasing the Ga loading in the sample. Thus, a maximum MeOH + DME production of 25,659 g_MeOH+DME_·kg_Pd_^–1^·h^–1^ with 75% selectivity to oxygenates (18% methanol/57% DME) is obtained on the PdGa_4_@MFI-700RED sample at 260 °C and 20 bar (Figure 3a).

**Figure 3:**
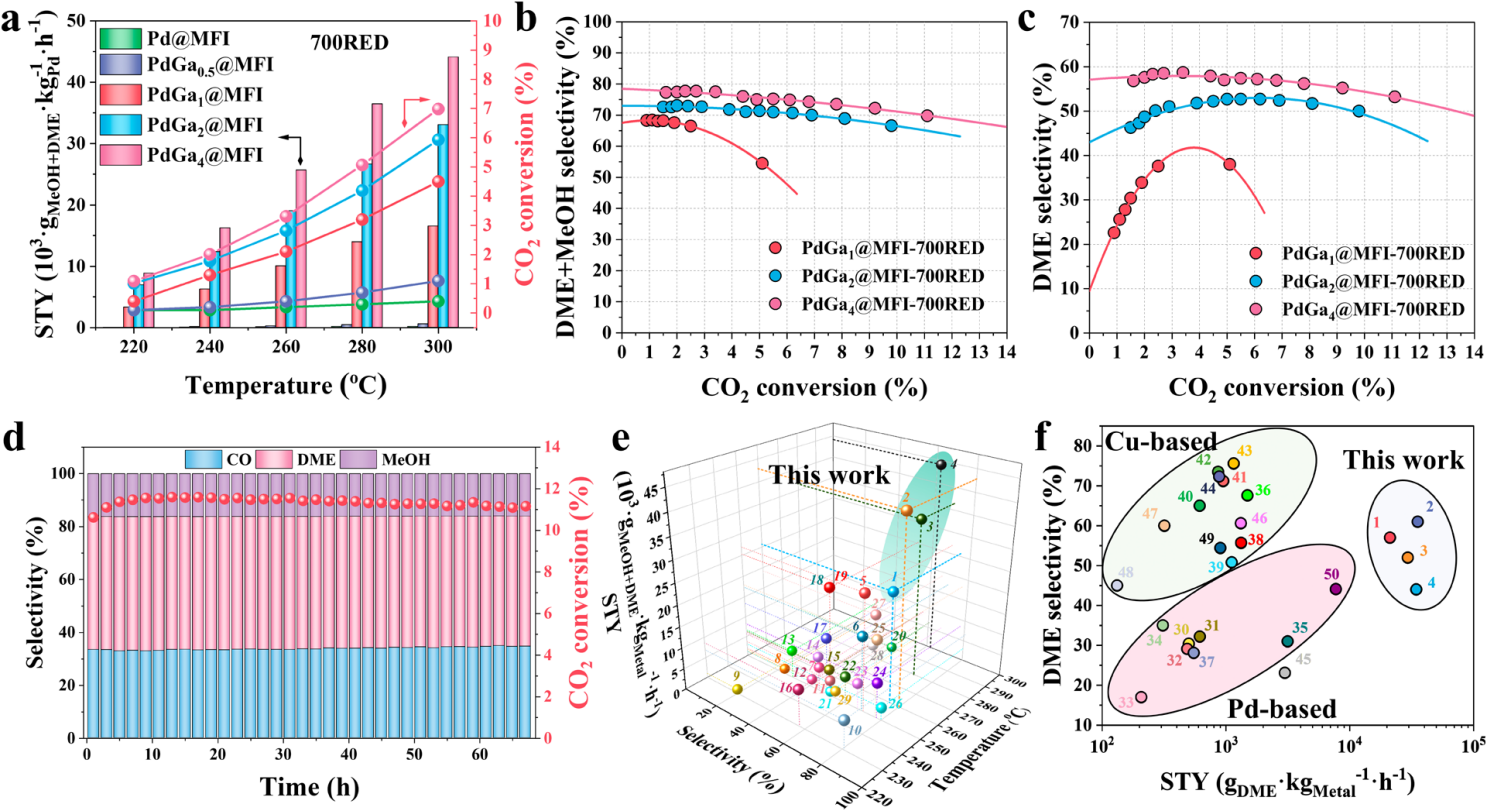
Catalytic performance of PdGa_*x*_@MFI-700RED samples, long-term stability, and comparison with state-of-the-art catalysts. (a) Space time yield and CO_2_ conversion on PdGa_*x*_@MFI-700RED catalysts at different temperatures, 20 bar, and WHSV = 15,000 mL·g_cat_^–1^·h^–1^. (b,c) DME + MeOH and DME selectivity as a function of CO_2_ conversion on PdGa_*x*_@MFI-700RED catalysts, at 260 °C and 20 bar. (d) Stability test on PdGa_4_@MFI-700RED catalyst at 260 °C, 20 bar and WHSV = 2100 mL·g_cat_^–1^·h^–1^. (e,f) Comparison with state-of-the-art catalysts. Numbers correspond to the entries of Tables S10 and S11.

The variation of the selectivity to oxygenates (DME + MEOH) and to DME with the CO_2_ conversion at a constant temperature of 260 °C is presented in Figure 3b,c, respectively, for the different catalysts. Higher selectivity to oxygenates, and in particular to DME, is observed on the PdGa_4_@MFI-700RED sample, independent of the CO_2_ conversion. For example, at 5% CO_2_ conversion, a selectivity of 75% to oxygenates and 57% to DME is obtained on the PdGa_4_@MFI-700RED sample, higher than that obtained on the PdGa_1_@MFI-700RED sample, with a selectivity of 55% to oxygenates and 38% to DME, at the same conversion. In addition, a long-term stability test shows a constant 11% CO_2_ conversion, at 260 °C, 20 bar, and WHSV = 2100 mL·g_cat_^–1^·h^–1^ for a stream time of 60 h on the most active PdGa_4_@MFI-700RED sample (Figure 3d). No particle agglomeration is observed on this catalyst (Figure S68). Based on literature data, the highest methanol and DME production so far for PdGa-based catalysts (Table S10 of state-of-the-art, and Figure 3e) has been reported by Liu et al.,^16^ over a 1 wt % Pd/Ga_2_O_3_/Al_2_O_3_ catalyst obtained using an atomic layer deposition methodology. In this case, a MeOH + DME production of 26,960 g_MeOH+DME_·kg_Pd_^–1^·h^–1^ with 70% selectivity to oxygenates (63% methanol/7% DME) and 7.1% conversion of CO_2_ is obtained at 250 °C and 45 bar (WHSV = 15,000 mL·g_cat_^–1^·h^–1^). Under similar conditions, our most active catalyst (PdGa_4_@MFI-700RED) gives 42,864 g_MeOH+DME_·kg_Pd_^–1^·h^–1^ with 80% selectivity to oxygenates (19% methanol/61% DME) and 5.2% CO_2_ conversion (Figure S66). Moreover, the DME production in our catalyst is competitive with literature data where two physically mixed catalysts are usually employed. Indeed, compared to Cu-based catalysts, which are usually more selective and active to methanol and DME than Pd-based catalysts (Figure 3f), the herein reported catalyst shows competitive DME selectivity at similar CO_2_ conversion (Figure 3f, S69 and Table S11 of state-of-the-art), representing an interesting alternative to actual catalysts with the advantage of integrating both functionalities in only one catalyst.

### Structure–Activity Correlations

From the above-reported data, it is evident that the presence of Pd domains, which are detected by IR-CO in the PdGa_*x*_@MFI-700RED samples (*x* = 0.5, 1, 2), reduces the selectivity to oxygenates, promoting CO formation. Indeed, CO is the predominant product formed on the Pd@MFI-700RED sample, composed of pure Pd nanoparticles confined in the MFI channels. Next, in order to correlate the catalytic activity of the different Pd and Ga surface species with their role in the CO_2_ hydrogenation to DME and MeOH, a detailed kinetic and spectroscopic study was conducted.

From a kinetic approach, initial reaction rates to each product (CO, MeOH and DME) have been calculated at 260 °C setting the CO_2_ conversion below 2% (Figures S70–S74 and Table S12) and the corresponding values are compared in Table S13 and displayed in Figure 4. The initial rate of methanol formation decreases when increasing the Ga loading in the sample (from 2.26 mmol·g_cat_^–1^ h^–1^ in PdGa_1_@MFI-700RED to 1.46 mmol·g_cat_^–1^ h^–1^ in PdGa_4_@MFI-700RED), while the DME initial rate increases at increasing Ga loading (from 0.37 mmol·g_cat_^–1^ h^–1^ in PdGa_1_@MFI-700RED to 3.90 mmol·g_cat_^–1^ h^–1^ in PdGa_4_@MFI-700RED) (Figure 4f). Interestingly, while DME is usually considered as a secondary product formed on acid sites by methanol dehydration (see schema in Figure 4e),^6^ the results of Figure 4f and Table S13 clearly indicate that DME appears as a primary product together with methanol in all the PdGa_*x*_@MFI sample, probably formed by a separated pathway. The initial DME formation rate is higher on the PdGa_4_@MFI-700RED sample compared with that of the other samples, being even higher than that of methanol production. Detailed analysis of the reaction path can be extracted from a Delplot-type analysis, as shown in Figure S75. For PdGa_2_@MFI-700RED and PdGa_4_@MFI-700RED, the Delplots for DME formation are consistent with predominately first-order behavior, indicating that a direct DME formation route from CO_2_ and H_2_ dominates for these samples. In contrast, on the PdGa_1_@MFI-700RED sample, the DME formation shows features compatible with both first- and second-order trends. Based on these results, the involvement of different sites in the methanol and DME synthesis following two different reaction paths has been hypothesized and investigated by in situ and operando IR studies.

**Figure 4:**
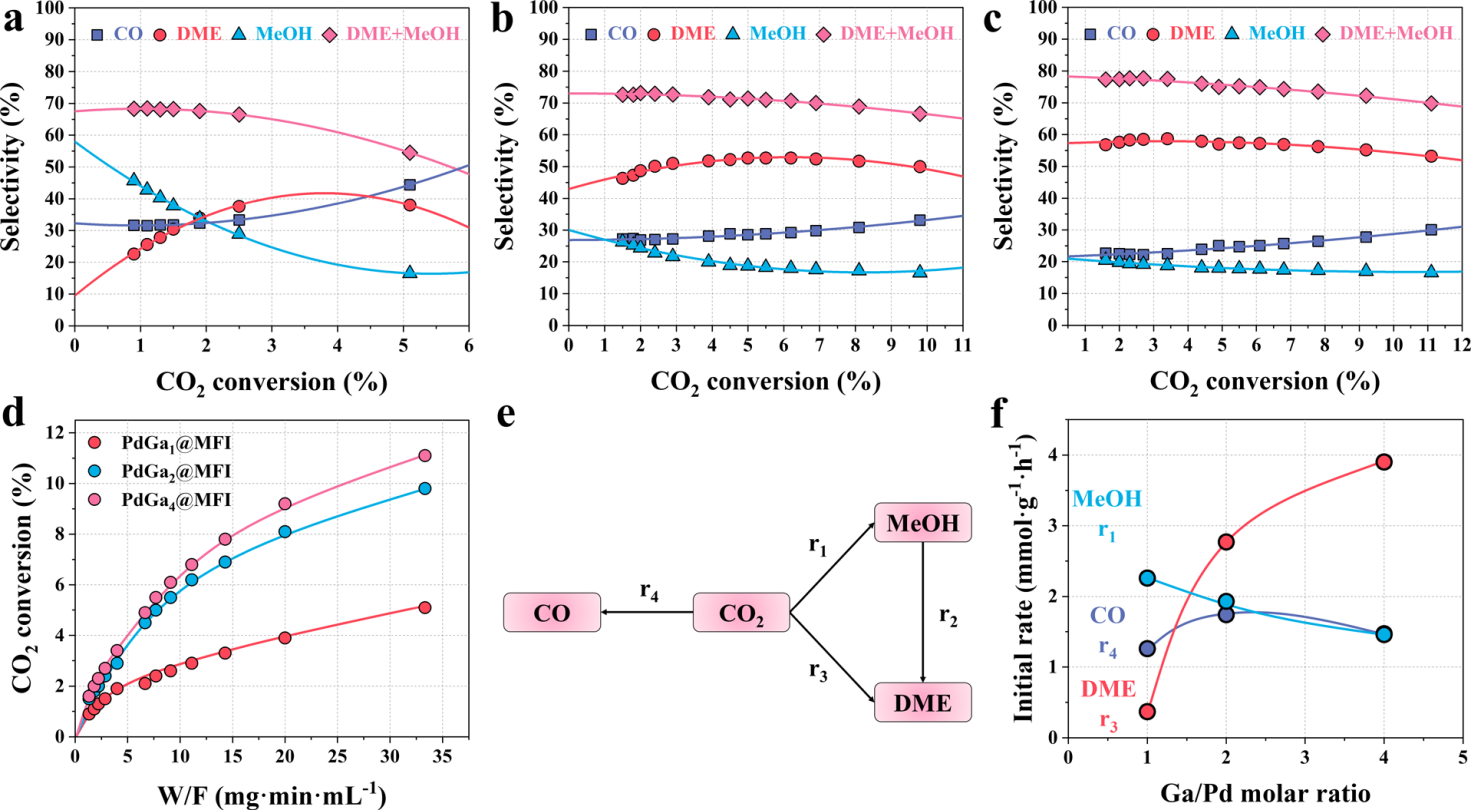
Selectivity variation for each product with respect to the CO_2_ conversion and initial formation rates for each product on the PdGa_*x*_@MFI-700RED catalysts. (a–c) CO, DME, and MeOH selectivities as a function of CO_2_ conversion on PdGa_1_@MFI-700RED (a), PdGa_2_@MFI-700RED (b), and PdGa_4_@MFI-700RED (c) catalysts at 260 °C and 20 bar. (d) CO_2_ conversion as a function of different contact times at 260 °C and 20 bar on PdGa_*x*_@MFI-700RED catalysts. (e) Reaction networks in CO_2_ hydrogenation reaction. (f) Initial rates for CO, DME, and MeOH formation on PdGa_*x*_@MFI-700RED with different Ga/Pd molar ratios. (Initial formation rates and selectivities were extrapolated using a second-order polynomial fit of the various contact time experimental data.)

In situ IR spectra under steady-state CO_2_ hydrogenation reaction conditions at 220–260 °C and 10 bar (Figure S79) on both PdGa_4_@MFI-700RED and PdGa_1_@MFI-700RED samples show the stabilization of monodentate (IR at ∼1650 cm^–1^ [ν_as_(OCO)]) and bidentate (IR at 1595–1610 cm^–1^ [ν_as_(OCO)]) formate intermediate species.^26^ The maxima of the peak associated with the bidentate formate component are located at higher frequency (∼1610 cm^–1^) in the PdGa_1_@MFI-700RED sample compared to that of the PdGa_4_@MFI-700RED sample (∼1595 cm^–1^), which may be related to formate species coordinated to different surface elements. In fact, the nature of Pd species determined by CO chemisorption, IR-CO and H_2_–D_2_ data, confirm Pd–Ga alloy nanoparticles with the coexistence of Pd domains in the PdGa_1_@MFI-700RED sample and Pd–Ga alloys in the PdGa_4_@MFI-700RED sample. In addition, a peak at 1906 cm^–1^ due to CO in bridge coordination with Pd domain is observed under steady-state CO_2_ + H_2_ reaction conditions in the PdGa_1_@MFI-700RED sample, while it is practically not observed in the PdGa_4_@MFI-700RED one. Changing the reactant feed from CO_2_ + H_2_ to H_2_ + He, the intensity of the mono- and bidentate formate species in the IR spectra decreases in intensity, but due to the high spectra noise, it is difficult to follow the evolution of each species (Figures S80–S81). Therefore, additional temperature resolved IR studies done at the 200–260 °C temperature have been performed, collecting spectra at each temperature (details in the Supporting Information, Figures S82 and S83). Under these conditions, when increasing the reaction temperature from 220 to 260 °C on the PdGa_4_@MFI-700RED sample, the band due to bidentate formate species (1602 cm^–1^) practically do not change, while it is possible to observe a decrease of the IR band at 1641 cm^–1^ associated with monodentate formate species in parallel with an increase of the IR band at 1701 cm^–1^ due to CH_2_O*,^27^ which can be attributed to the conversion of monodentate formate to CH_2_O* species (Figure S82). CH_2_O* has been reported as intermediate in both methanol and DME.^28,29^ In contrast, in the PdGa_1_@MFI-700RED sample, the IR signals corresponding to formate intermediate species practically does not modify at increasing reaction temperature, while CO is detected in this case after depressurization of the IR cell (Figure S83d), not being observed in the previous sample.

The fact that CO is detected together with formate intermediate species opens the question if CO participates in the reaction mechanism as a reaction intermediate in the methanol/DME synthesis. In order to support this assumption, a kinetic study was done in a fixed bed microreactor where small amounts of CO (8–20 vol %) is added to the reactant feed once the catalyst has achieved steady-state operation conditions, following the response to products by mass spectrometry (Figure 5). Interestingly, methanol and DME are promoted by cofeeding CO in the PdGa_1_@MFI-700RED sample, confirming its participation in the reaction mechanism. However, in the PdGa_4_@MFI-700RED sample, methanol is inhibited in the presence of CO due to a competitive adsorption of CO over CO_2_ on the active sites, blocking CO_2_ hydrogenation, while DME remains unaffected after CO coaddition. The blocking of CO has been shown to be reversible, recovering methanol production after removal of CO from the reactant feed and neglecting catalyst restructuring under reaction conditions. These results confirm that CO is not participating in the reaction path of the PdGa_4_@MFI-700RED sample, while it follows a CO_2_ hydrogenation path via formate intermediate species as confirmed by IR studies. On the other hand, it confirms the involvement of two separate active sites for methanol and DME, responding differently under CO cofeeding.

**Figure 5:**
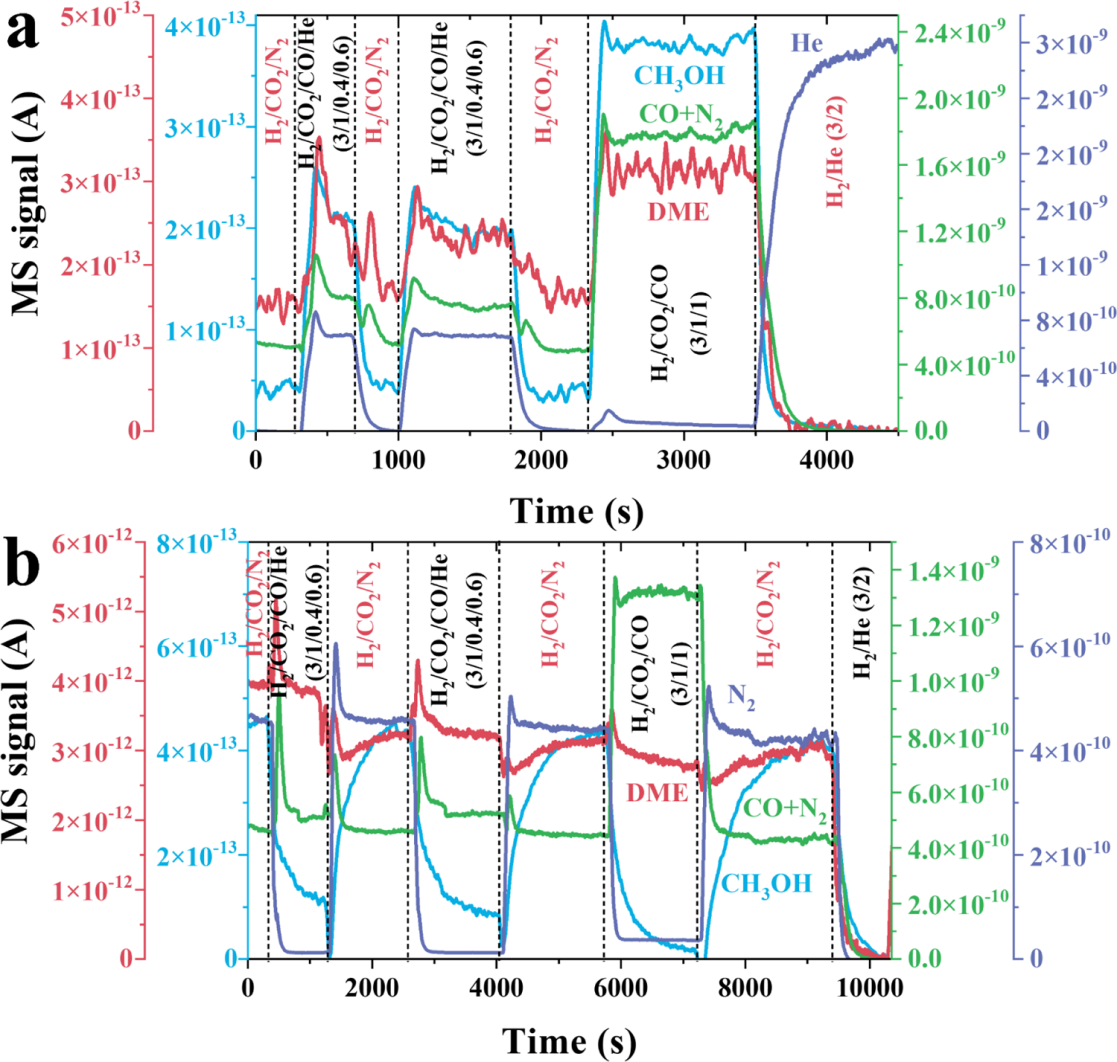
Kinetic studies of the effect of CO on the CO_2_ hydrogenation to methanol and DME on PdGa@MFI-700RED catalysts. Kinetic studies of the effect of adding small amounts of CO in the reactant feed in the CO_2_ hydrogenation to methanol over (a) PdGa_1_@MFI-700RED and (b) PdGa_4_@MFI-700RED catalysts. Effluent gas is measured with a mass spectrometer; signals *m*/*z* 4 (He), 14 (N_2_), 28 (CO + N_2_), 31 (CH_3_OH), and 46 (DME). Reaction conditions: 90 mg catalyst, 260 °C, 10 bar, 25 mL/min, and gas mixtures: H_2_/CO_2_/N_2_ (3/1/1), H_2_/CO_2_/CO/He (3/1/0.4/0.6 and 3/1/1/0), and H_2_/He (3/2).

Interestingly, the in situ IR studies described above show clearly two different intermediate formate species, mono- and bidentate on the PdGa_4_@MFI-700RED sample, while at that moment it was not possible to discriminate which one participates in the reaction path to methanol and DME formation. In view of this, a similar experiment of CO cofeeding is done in the operando IR study in order to discriminate, which formate species is involved in DME and in the methanol synthesis. By doing so, it can be seen that monodentate formate species and the production of DME remain unperturbed after CO cofeeding, appearing as possible intermediates in the direct DME formation. Meanwhile, bidentate formate species decrease in intensity after CO feeding, with a corresponding decrease in methanol production (Figure 6). Due to the high affinity of Pd sites for CO, it is expected that the bidentate formate species reported above involves at least one Pd atom in its configuration, whereas monoformate species are expected to be stabilized on the oxophilic Ga sites (isolated Ga^+^), which has a lower CO affinity than Pd. Additional IR experiments of adsorption of formic acid on representative samples, i.e., Ga_4_@MFI-CAL and Ga_4_@MFI-RED, PdGa_4_@MFI-700RED, and Pd@MFI-700RED, support the assignment of the IR band at 1643 cm^–1^ to monoformate species stabilized on Ga^+^ sites (Figures S84–S88 and discussion in Section S7.1.3). These results suggest that isolated Ga^+^ are involved in the direct path of DME formation assisted by adjacent acid sites, whereas Pd–Ga sites are involved in methanol formation, following two distinct reaction pathways: (i) a formate path as on the PdGa_4_@MFI-700RED sample containing isolated Pd sites in a Ga rich phase, and (ii) a CO-mediated reaction path as on the PdGa_1_@MFI-700RED sample, containing Pd domains in a Pd-rich PdGa phase.

**Figure 6:**
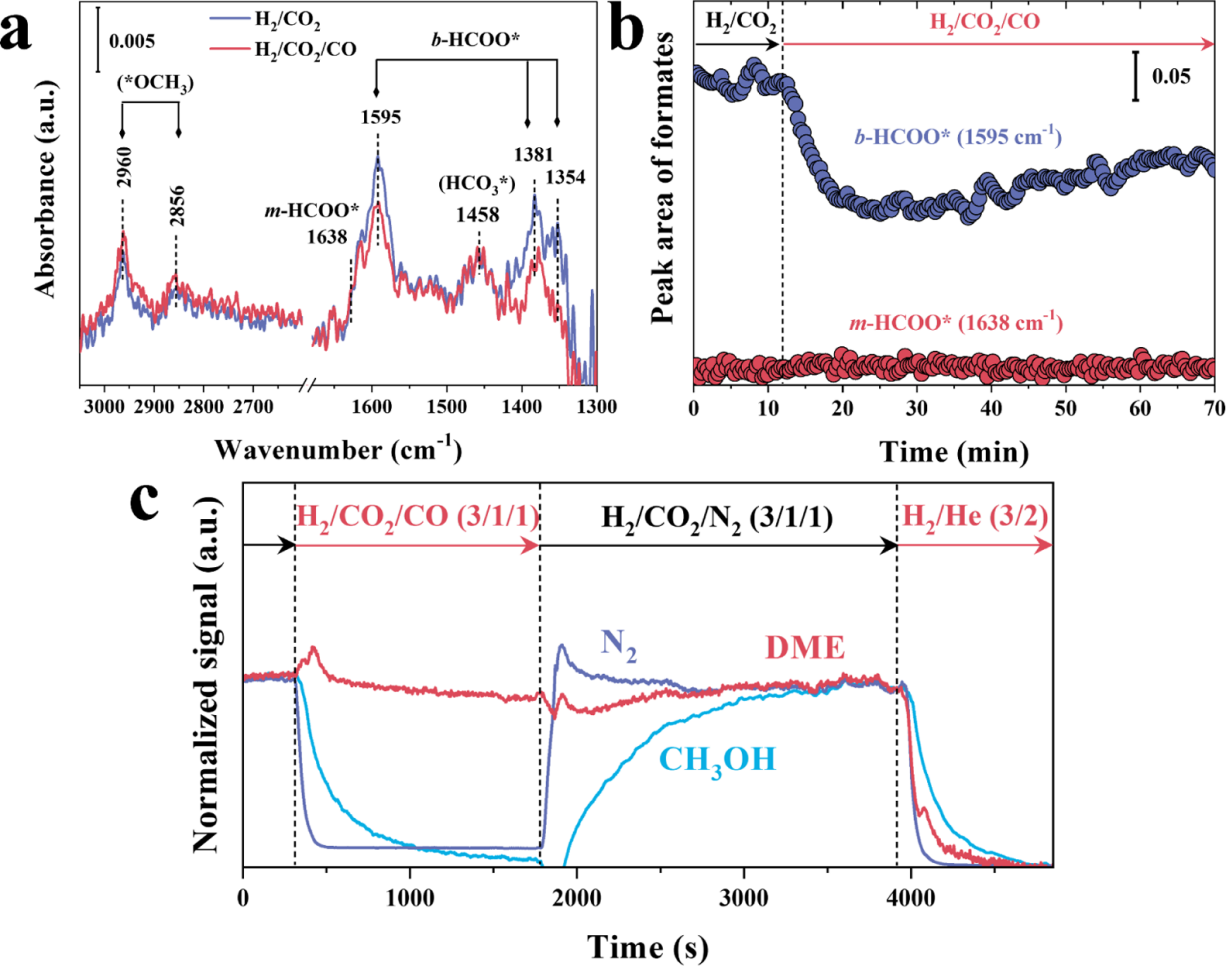
IR transient study of the effect of CO on the CO_2_ hydrogenation to methanol and DME over the PdGa_4_@MFI-700RED catalyst. (a) IR spectra in steady-state conditions under H_2_/CO_2_ and H_2_/CO_2_/CO. (b) Variation of formate peak area as a function of time by changing the gas mixture feed. (c) Mass spectra analysis of the effluent gas measured with a mass spectrometer; signals *m*/*z* 14 (N_2_), 31 (CH_3_OH), and 46 (DME). Reaction conditions: 200 °C, 10 bar, 25 mL/min, and gas mixtures: H_2_/CO_2_/N_2_ (3/1/1), H_2_/CO_2_/CO (3/1/1), and H_2_/He (3/2). Signal normalization was done using as reference value of the respective signal during the H_2_/CO_2_/N_2_ condition. Surface intermediates: methoxy species (2960 and 2856 cm^–1^), formate species (1638, 1595, 1381, and 1354 cm^–1^), and bicarbonate (1458 cm^–1^).

Based on the above, a general scheme of the probable reaction routes that take place on the PdGa_*x*_@MFI-700RED samples is given in Figure 7, highlighting the key role of isolated Ga^+^ and adjacent Brønsted acid sites for the direct DME formation.

**Figure 7:**
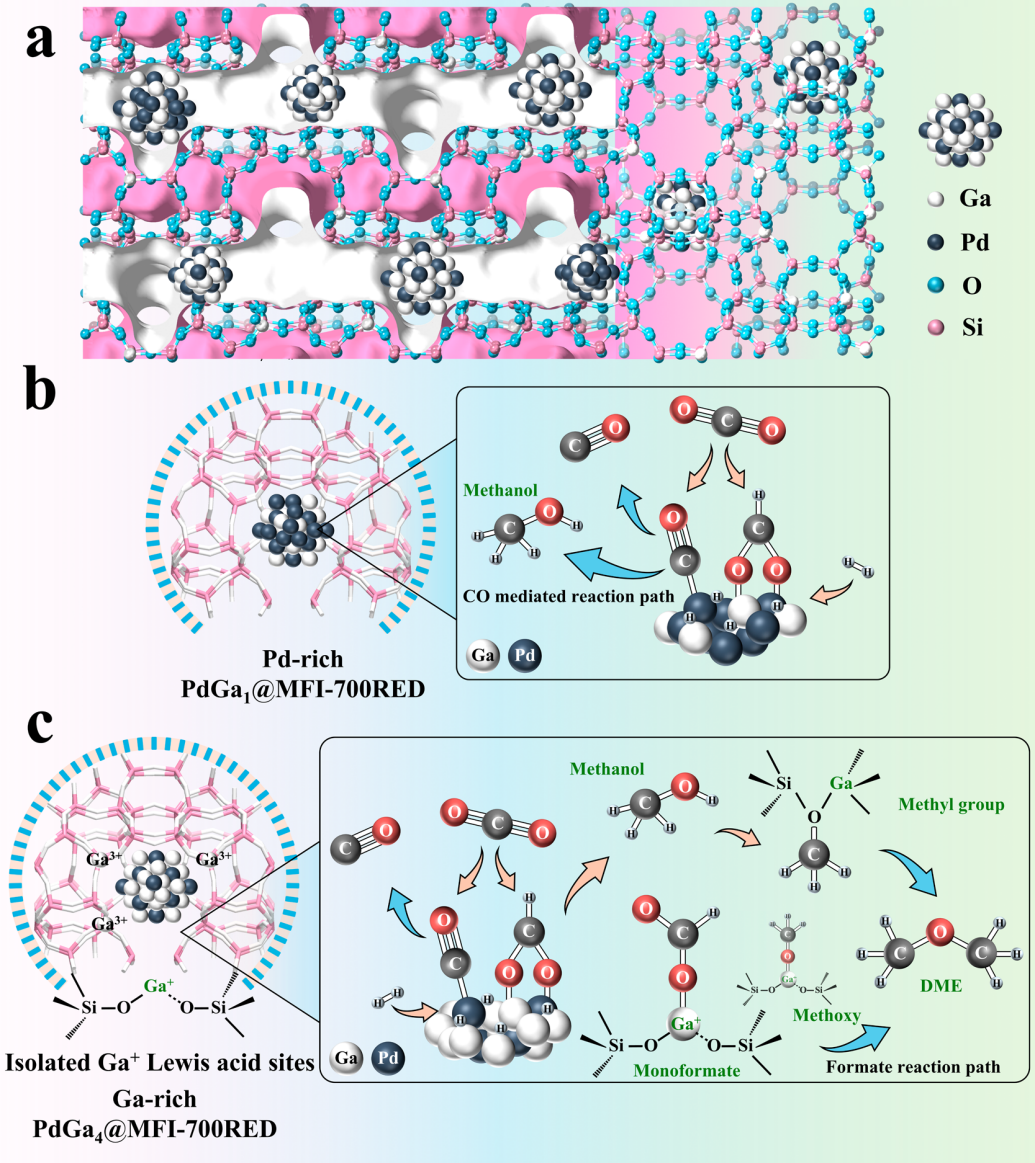
Schematic vision of the possible reaction paths in the CO_2_ hydrogenation to methanol and DME on PdGa_*x*_@MFI samples. (a) Illustration of PdGa nanoparticles confined inside MFI zeolite and CO_2_ hydrogenation to methanol or DME routes on (b) Pd-rich PdGa_1_@MFI-700RED and (c) Ga-rich PdGa_4_@MFI-700RED catalysts containing Ga^+^ sites.

Thus, we can conclude that on the Pd-rich PdGa_1_@MFI-700RED sample, methanol is likely produced by two reaction paths, a CO and a formate-mediated path. On the Ga-rich PdGa_4_@MFI-700RED sample, methanol is formed on the PdGa alloy via a formate path, which on an adjacent acid site is partially dehydrated to methyl species. On the other hand, DME is formed on Ga^+^ sites via monoformate intermediates, which transform into methoxy and, in the presence of adjacent methyl species, result in DME formation.

### Role of Zeolite and Metal Confinement

As discussed previously and summarized in Figure 7, the high selectivity to oxygenated surfaces with low CO formation and enhanced DME formation requires Ga-rich PdGa alloys and Ga^+^ sites in close proximity to acid sites. This strategy can be achieved by confining Pd in a Ga@MFI zeolite and forcing Ga^3+^ migration to be alloyed with the Pd while stabilizing Ga^+^ species on silanol nests or on acid sites. The important role of metal confinement in the zeolite is discussed in this section, comparing the PdGa_4_@MFI sample with that of a PdGa_*x*_/SiO_2_ impregnated sample with a similar PdGa particle size as in the zeolite sample.

PdGa_*x*_/SiO_2_ (*x* = 1 and 4) with 2 nm particle size is prepared by the incipient wetness impregnation method using amorphous SiO_2_ (Aerosil 200) as support and functionalizing with amino groups in order to ensure a good dispersion of metal sites (see Supporting Information for more details, Section S8). The sample is ex situ reduced at 700 °C. The ∼2 nm particle size is confirmed from HR-HAADF-STEM analysis (Figures S95–S101).

Compared to the PdGa_4_@MFI-700RED catalyst, the impregnated PdGa_1_/SiO_2_ and PdGa_4_/SiO_2_ samples behave less active and selective to oxygenates under similar reaction conditions (Figure S107 and Table S21). The higher level of CO formation of the impregnated samples is related to the higher contribution of Pd domains as determined from IR-CO analysis (Figure S106). The unique surface configuration enabling isolated-single Pd site stabilized in a Ga-rich PdGa phase is hard to obtain using an impregnation approach, even if the Ga loading is increased to a ratio Ga/Pd = 4. This indicates the close proximity of Ga species surrounding the Pd particle, a key aspect for the formation of the surface Ga-rich PdGa alloy structure, which is possible on zeolite-based samples taking advantage of metal confinement in the channels of the zeolite and Ga^3+^ located in framework positions. On the other hand, in contrast to zeolite-based samples, a very fast oxidation of Ga species, resulting in the formation of a GaO_*x*_ shell covering the PdGa surface, is observed after air exposure in the impregnated PdGa_*x*_/SiO_2_ samples, behavior also observed by other authors.^30^ The formation of a thick shell is confirmed by XAS (Figure S104), IR-CO (Figure S106), CO chemisorption (Table S20), and STEM (Figure S98). This shell is enhanced if a PdGa_4_ composition is used (see Figure S106 and Table S20) in the impregnated samples, but it is not observed in zeolite samples. Furthermore, in contrast to zeolite-based samples, for the supported PdGa_1_/SiO_2_-700RED sample, a much higher in situ reduction temperature (500 °C versus 350 °C for zeolite-based materials) is needed for complete removal of the GaO_*x*_ shell on the PdGa surface (see Table S20), indicating a protecting role of the zeolite avoiding extensive surface oxidation and GaO_*x*_ segregation and ensuring catalyst stability.

While Ga^+^ species are stabilized on the supported 2 nm PdGa_1_/SiO_2_ sample (see XAS data in Figure S104) using amorphous Aerosil 200 as support, the absence of adjacent Brønsted acid sites inhibits the direct DME formation path (Figure S108). Notably, the initial rate of methanol formation (see Table S13) is much higher in the 2 nm PdGa_1_/SiO_2_ sample (4.56 mmol·g_cat_^–1^ h^–1^) than in the PdGa_4_@MFI-700RED catalyst (1.46 mmol·g_cat_^–1^ h^–1^), but similar if in the last sample the two sites for methanol and DME are considered together (i.e., by adding the initial rates to methanol and DME (1.46 + 3.90 mmol·g_cat_^–1^ h^–1^, respectively)). In this sense, it is possible to assume that depending on whether adjacent Brønsted acid sites exists or not close to PdGa alloys for the stabilization of methyl species (CH_3_*), the methoxy species stabilized on Ga^+^ (H_3_CO*) may evolve into either DME (via coupling with CH_3_*) or methanol (via hydrogenation in absence of adjacent CH_3_*), respectively. This indicates the potential participation of Ga^+^ species also in methanol synthesis, explaining the higher methanol formation rate of the PdGa_1_/SiO_2_ sample.

Finally, the hydrophilic character of the zeolite offers an additional advantage over the catalytic performance, which is not found on the impregnated samples. In this sense, MeOH and DME production on the zeolite sample (PdGa_4_@MFI-700RED) exhibit Gaussian-like behavior in controlled experiments where small amounts of water are added to the reactant feed (0–3.8 vol %) (Figure S109), while this effect is not observed on the impregnated samples (Figure S110). Such a promotion effect of water on hydrophilic samples has already been discussed in the literature.^31^

### Alternative Synthesis Approach

Besides the one-pot hydrothermal synthesis method, where both Pd and Ga salts are added simultaneously in the synthesis gel, alternative methods based on Ga loading on Pd@S-1 or Pd@ZSM-5 samples via wet impregnation (IM-Ga_4_/Pd@ZSM-5-700RED), ionic exchange (IE-Ga_4_/Pd@ZSM-5-700RED), or using physical mixing methods (PM-Ga_2_O_3_/Pd@ZSM-5-700RED) have also been explored here since it is a method widely used in the literature. Detailed characterizations of the samples are included in the Supporting Information, and its catalytic activity is summarized in Section S9 of the Supporting Information. Among the different synthesis strategies, Ga impregnated on Pd@ZSM-5 is the most promising approach to get close activity to PdGa_4_@MFI-700RED (Figure S120), but it behaves less selective to oxygenates as displayed in Figure 8. This difference among the IM-Ga_4_/Pd@ZSM-5-700RED and PdGa_4_@MFI-700RED catalysts is due to the coexistence of Pd domains and PdGa alloy in the impregnated one (Figure S116). Ga^+^ sites are less stabilized on the IM-Ga_4_/Pd@ZSM-5-700RED sample based on XAS analysis (Figure S117), indicating that the stabilization of Ga^+^ is related to hydroxyl nests and acid sites associated with the isomorphous substitution of Ga^3+^ in the zeolite framework. Once more, this result highlights the unique properties of the one-pot hydrothermal synthesis method in achieving well distributed single Pd sites in a Ga-rich environment and Ga^+^ sites enabling high selectivity to oxygenated (DME and methanol) production.

**Figure 8:**
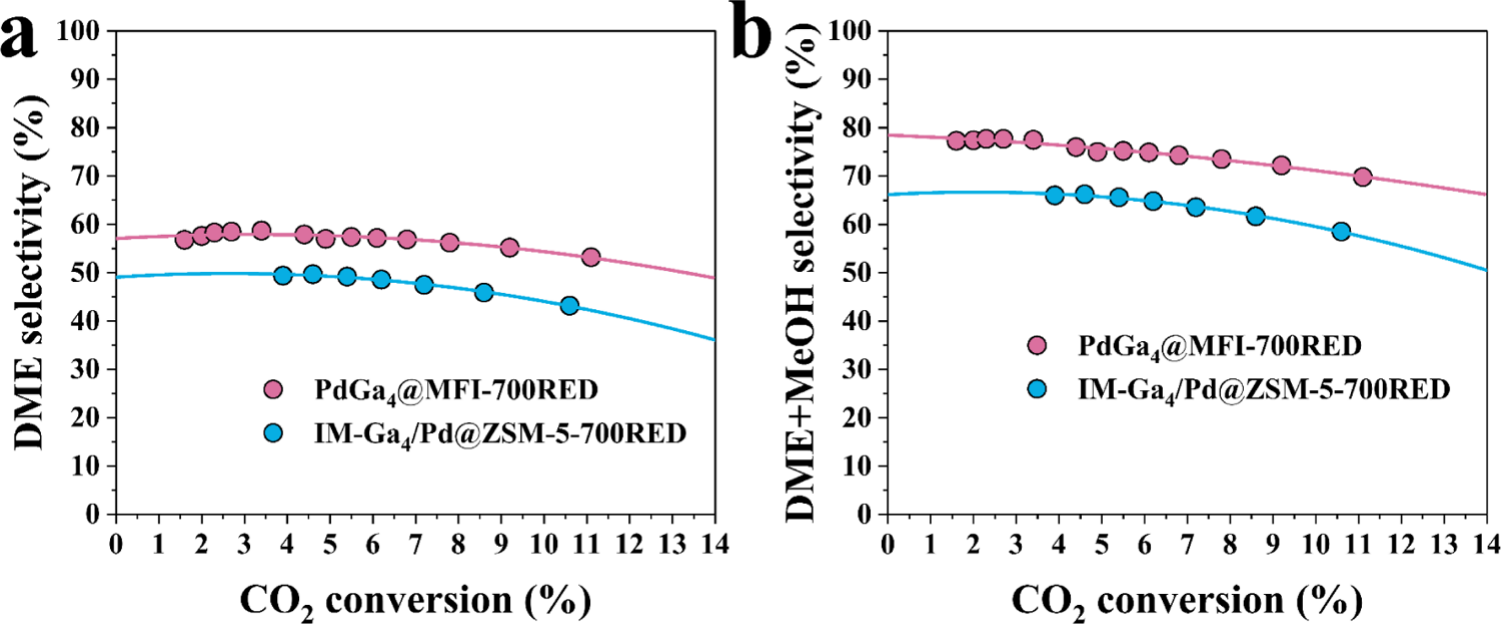
Comparative study of the variation of the selectivity to DME and oxygenates (DME + MeOH) over a Ga_4_/Pd@ZMS-5-700RED and PdGa_4_@MFI-700RED samples at increasing CO_2_ conversion. (a) DME selectivity versus CO_2_ conversion and (b) oxygenates (DME + MeOH) selectivity versus CO_2_ conversion at 260 °C, 20 bar, and WHSV = 1800–45,000 mL·g_cat_^–1^·h^–1^ on PdGa_4_@MFI-700RED and IM-Ga_4_/Pd@ZSM-5-700RED catalysts.

## Conclusion

In this work, the direct CO_2_ hydrogenation to dimethyl ether (DME) path has been investigated. The formation of DME by direct CO_2_ hydrogenation involves several consecutive reactions occurring on the catalyst surface involving tandem reactions on several active sites. We demonstrate that it is possible to reach STY 42,864 g_MeOH+DME_·kg_Pd_^–1^·h^–1^ with 80% selectivity to oxygenates (19% methanol/61% DME) and 5.2% CO_2_ conversion at 260 °C, 45 bar, and WHSV = 15,000 mL·g_cat_^–1^·h^–1^, with a catalyst that combines three catalytic functions, i.e., PdGa alloys, Ga^+^ Lewis acid sites, and Brønsted acid sites, outperforming state-of-the-art PdGa catalysts. PdGa alloys catalyze CO_2_ hydrogenation to methanol, which on an adjacent acid site, is dehydrated to methyl species, suppressing CO formation. Ga^+^ Lewis acid sites are responsible for the stabilization of methoxy species to form methanol, and in the presence of methyl species, DME is favored. The key role of isolated Ga^+^ Lewis acid sites in close proximity to Brønsted acid sites for stabilizing monoformate intermediate species and facilitating the direct production of DME has been confirmed by time-resolved kinetic studies, in situ XAS, and operando IR.

In addition, this work highlights the important role of the zeolite in metal confinement, conferring excellent stabilization of the Lewis acid sites, oxidation resistance, hydrophilicity, and close proximity of the active sites in the multifunctional catalyst.

## Supplementary Material



## Notes

Detailed experimental procedures, characterization methods, and other supporting characterization results (PDF)
