# Supplementary material for: Decoding the Role of Isolated Ga+ in PdGa@MFI Catalyst Promoting a Direct CO2 Hydrogenation Path to DME
Source: J Am Chem Soc. 2026 Feb 9;148(6):6531–43. doi: 10.1021/jacs.5c20643 (PMC12921912; doi:10.1021/jacs.5c20643)
Supplement: Supplementary file 1 [file ja5c20643_si_001.pdf]

Supplementary Information

Decoding the role of isolated Ga^+ in PdGa@MFI catalyst promoting a direct CO_2 hydrogenation path to DME

Minjie Zhao[a], Daviel Gómez[a], Vlad Martin Diaconescu[b], Laura Simonelli[b], Miguel Lopez Haro[c], Jose Juan Calvino[c], Avelino Corma[a]*, Patricia Concepción[a]*

[a] *Instituto de Tecnología Química, Universitat Politècnica de València-Consejo Superior de Investigaciones Científicas (UPV-CSIC), Avenida de los Naranjos s/n, 46022 Valencia, Spain.*

[b] *CELLS - ALBA Synchrotron Radiation Facility, Carrer de la Llum 2-26, 08290 Cerdanyola del Vallès, Spain.*

[c] *Departamento de Ciencia de los Materiales e Ingeniería Metalúrgica y Química Inorgánica. Facultad Ciencias. Universidad de Cádiz. Campus Rio San Pedro. Puerto Real, 11510-Cádiz, Spain.*

Table of contents

1. Material and methods
2. Synthesis of PdGa@MFI samples
3. Physico-chemical characterization of $PdGa_x$@MFI samples
4. Effect of the reduction temperature on the $PdGa_4$@MFI sample
5. Influence of the Ga/Pd ratio in $PdGa_x$@MFI samples
6. Catalytic performance of $PdGa_x$@MFI in CO_2 hydrogenation
7. *In-situ* spectroscopic studies on on $PdGa_x$@MFI-700RED samples
8. $PdGa_x/SiO_2$ supported samples
9. Alternative approaches for the preparation of PdGa@MFI
10. Appendix
11. Reference

1. Material and methods

1.1. Chemical reagents. All chemicals and materials were obtained from commercial sources and used without further purification unless otherwise specified. Tetrapropylammonium bromide (TPABr), tetraethylorthosilicate (TEOS), ethylenediamine (EDA), (3-mercaptopropyl)trimethoxysilane (MPTMS), 3-[2-(2-aminoethylamino)ethylamino]propyltrimethoxysilane (APTMS), (3-aminopropyl)triethoxysilane (APTES), palladium (II) chloride ($PdCl_2$), tetraamminepalladium (II) chloride monohydrate ($Pd(NH_3)_4Cl_2{\cdot}H_2O$), palladium (II) nitrate dihydrate ($Pd(NO_3)_2{\cdot}2H_2O$), palladium(II) acetylacetonate ($Pd(acac)_2$), gallium nitrate hydrate ($Ga(NO_3)_3{\cdot}xH_2O$), commercial gallium(III) oxide (β-Ga_2O_3), ethanol and ammonia solution (25wt%) were purchased from Sigma Adrich, 99.9% purity. Anion exchange resin (Amberlite® IRN-78) was purchased from Thermo Scientific. SiO_2 (Aerosil 200) was purchased from Evonik Degussa Germany company, High-Surface-Area mesoporous SiO_2 was purchased from Sigma Adrich. Tetrapropylammonium hydroxide (TPAOH) was obtained by ionic exchange of TPABr with anion exchange resin. Deionized (DI) water from Milli-Q integral water purification system (Millipore, 18.2 $M\Omega{\cdot}cm^{-1}$).

1.2. Catalyst synthesis. PdGa@MFI is synthetized by conventional hydrothermal synthesis with tetraorthoethylsilicate (TEOS) as silica source and tetrapropylammonium hydroxide (TPAOH) as organic template. For the synthesis of PdGa@MFI, SiO_2:TPAOH:H_2O:Pd:Ga=1:0.4:25:0.0034:n (n=0, 0.0017, 0.0034, 0.0068, 0.0135) are used, representing $PdGa_x$@MFI (x=0, 0.5, 1, 2 and 4) with 0.6wt% Pd loading. Normally, 0.3567g $Pd(NH_3)_4Cl_2{\cdot}H_2O$ is dissolved in 8g H_2O, then 2g APTMS is added and ultrasonic for 10 min to form a uniform Pd-APTMS complex. For the synthesis of $PdGa_4$@MFI, for example, 8.32g TEOS, 8g TPAOH (40wt%), 20g Milli-Q water, 0.1824g $Ga(NO_3)_3{\cdot}xH_2O$ is mixed and kept stirring overnight. Then 1g Pd-TAPTS is added into the clear zeolite synthesis gel and transferred to a 35mL Teflon-lined autoclave and heated to 170 °C under agitation for 4 days for crystallization. When the

crystallization was finished, the solid product was collected by filtration and washed thoroughly with distilled water and dried in air at 100 °C. The dried sample is directly reduced in a tubular oven in pure H_2 (50 mL/min) with a heating rate of 10 °C/min to 700 °C and maintain 6 hours. The obtained sample was named $PdGa_4$@MFI-700RED. The sample under 500, 600 and 800 ºC H_2 post-treatments are named as $PdGa_4$@MFI-500RED, $PdGa_4$@MFI-600RED and $PdGa_4$@MFI-800RED. The sample after 550 ºC calcination 6h with 1.5 °C/min ramping rate and then 700 ºC H_2 reduction is named as $PdGa_4$@MFI-CAL-700RED. Different weight of $Ga(NO_3)_3{\cdot}xH_2O$ (0, 0.0228, 0.0456, 0.0912g) are added to get Pd@MFI-700RED, $PdGa_{0.5}$@MFI-700RED, $PdGa_1$@MFI-700RED and $PdGa_2$@MFI-700RED, separately.

1.3. Characterization methods. Samples for electron microscopy studies were prepared by dropping the suspension of PdGa@MFI catalysts, using CH_2Cl_2 as the solvent, directly onto holey-carbon-coated Cu grids. The measurements were performed using a JEOL 2100F microscope operating at 200 kV, both in transmission (TEM) and scanning-transmission modes (STEM). HR-HAADF-STEM and iDPC imaging were performed using a double aberration-corrected, monochromated FEI Titan3 Themis 60-300 microscope operating at 300 kV. Specifically, 2048 x 2048 HAADF-iDPC image pairs were acquired with a convergence angle of 18.6 mrad and collection angles of 48-198 mrad. This configuration optimized signal collection on the HAADF and FEI DF4 detectors. To minimize electron beam damage, a fast image recording protocol was employed, utilizing a beam current of 10 pA, a 1.25-μs dwell time (corresponding to a dose rate of 2500 $e^-/Å^2$), and automated fine-tuning alignment of A1 and C1 using OptiSTEM software. The microscope was also used for chemical mapping via STEM X-ray Energy Dispersive Spectroscopy (XEDS) at medium magnifications. This involved the microscope's high-efficiency SuperX G2 detection system, which integrates four windowless detectors surrounding the sample and high-performance signal processing hardware. STEM-XEDS was used to map the spatial distribution of metallic (Pd, Ga). For these nanoanalytical experiments, the beam current was increased to 60 pA and the dwell time to 1.2 ms to achieve a good signal-to-noise ratio. The elemental maps were then

background-subtracted and smoothed using a Gaussian blur with a sigma of 0.7. The quantitative analyses were performed on Pd L and Ga K families lines using the K-factor using a Schreiber-Wims ionization cross section model provided by the Velox software. To determine the spatial distribution of the metallic species within the zeolite framework, a specific methodology for the digital analysis of the experimental images has been developed and coded in a home-made MATLAB script. First, to improve the signal-to-noise, the HR-HAADF STEM images were denoised by combining the Anscomb transform and Undecimated Wavelet Transforms (UWVT). Then, a user-independent, fully automated, segmentation of image contrasts by clustering techniques (K-means method) was applied to recognize and classify the metallic entities, which is a requirement to guarantee statistically meaningful and unbiased results.

Inductively Coupled Plasma Mass Spectrometry (ICP) was carried out with a Varian 715-ES ICP-Optical Emission spectrometer and analyzed with a SCHN FISONS elemental analyzer. Textural properties like BET surface area, micropore volume and external surface area were measured on Micromeritics ASAP2000. Solid-state ^{29}Si and ^{71}Ga MAS NMR spectra were recorded at room temperature under magic angle spinning in a Brucker AV-400 spectrometer. Powder X-ray diffraction was performed with a HTPhilips X'Pert MPD diffractometer equipped with a PW3050 goniometer using Cu Kα radiation and a multisampling handler ($\lambda = 0.15406$ nm).

Pyridine adsorbed IR measurements were conducted on a Nicolet 6700 instrument, equipped with an MCT detector. Take $PdGa_4$@MFI-700RED as example, ~20 mg catalyst power was pressed into a wafer with a diameter of 1/2 inch, which was then vertically placed in a customized transmission cell. This cell was equipped with a vacuum manifold connected to a mechanical pump and a diffusion pump, achieving a vacuum level below 0.01 mTorr. In addition, the cell was wrapped with heating tapes and the temperature was controlled by a PID system with a thermocouple near the sample. Typically, the sample was dehydrated at 400 ºC for 2h under vacuum to remove adsorbed molecules. After dehydration, the sample was cooled down to room temperature under vacuum. Pyridine vapor was then introduced into vacuum cell for a specific duration to ensure full saturation

of pyridine. Finally, the sample was heated the sample at 150, 250 and 350 ºC to selectively desorb pyridine adsorbed on different acid sites.

H_2/D_2 isotopic exchange experiments were performed in a flow reactor. The reaction products (H_2, HD, D_2) were analyzed with a mass spectrometer (Omnistar, Balzers). Firstly, 5mg PdGa@MFI and supported PdGa/SiO_2 samples were pre-activated at 350 or 500 °C for 2h with a ramping rate of 10 °C/min. After activation, H_2 temperature programmed desorption was carried out at inert gas Ar atmosphere at 500 ºC for 30min to remove possible Pd-hydride. Subsequently, the reactor was cooled down to 25 ºC and H_2/D_2 isotopic exchange experiments were carried out at 25, 60, 90 and 120 ºC.

The dispersion and mono-layer CO adsorption capacity of Pd in PdGa@MFI samples was estimated from CO adsorption using the double isotherm method on a Quantachrome Autosorb-1C instrument. the samples (200-300 mg) were reduced *in-situ* in a flow of pure H_2 at 25 ml/min H_2 under desired temperature for 2h. After the reduction treatment, the samples were cooled down to 25 ºC and degassed at 1.33 Pa for 2h. Subsequently, pure CO was admitted and the first adsorption isotherm (representing the total CO uptake) was measured. After evacuation at 25 °C, the second isotherm (representing the reversible CO uptake) was measured. The amount of chemisorbed CO was then obtained by subtracting the two isotherms. The pressure range studied was between $(0.5\text{-}11)\times 10^4$ Pa. The dispersion of Pd (D) was calculated based on the amount of irreversibly adsorbed CO, assuming a stoichiometry of Pd/CO = 1.

IR spectra of CO adsorbed on PdGa@MFI and supported PdGa/SiO_2 catalysts were recorded with Bruker 70V spectrometer under -100 ºC. Operando IR-MS were performed in a commercial IR catalytic cell (Aabspec) connected "online" to a mass spectrometer (MS) (Balzer (QMG 220 M1)) under 9 bar of reaction gas.

In-situ X-ray absorption spectra (XAS) spectroscopy was acquired at CLÆSS beamline 22 of the ALBA synchrotron. XAS of powder pellets were acquired at the Ga and Pd K-edge diluted in boron nitride when necessary using a Si (311) double crystal monochromator. Reference spectra were collected in transmission mode, while data on

samples were collected in fluorescence mode using a multichannel SDD fluorescence detector available at the CLÆSS beamline of the ALBA synchrotron[1]. Several XAS repeats were collected to ensure reproducibility and statistics. Spectra processing and analysis was carried out with the Athena software package[2]. Energy calibration for the Ga K-edge was carried out by setting the first inflection point of Pt foil L3-edge to 11564 eV while for the Pd K-edge, the maximum of the first derivative of the spectra of Pd foil was set to 24350 eV.

Ga K-edge XANES were analysed by advanced statistical methods. A principal component analysis (PCA) allowed to identify the presence of a minimum number of components (5) in the analyzed data set. 5 components were then used in multivariate curve resolution-alternating least squares (MCR ALS) approach within using the MCR toolbox[3,4]. The initial estimate for the spectra of the pure components was done by selecting the spectra with higher variability directly from the data set, and then the ALS algorithm was operated using as constraints non-negativity for spectra and concentration profiles, and equality to 1 for the last points of the spectra.

The MCR component assignations has been done by comparing to reference spectra and literature.

EXAFS were extracted using the AUTOBK algorithm. At the Ga K-edge a spline in the range of 1 to 15.5 $Å^{-1}$ with an R_{bkg} of 1.1 was used while at the Pd K-edge a spline in the 1.5 to 15 $Å^{-1}$ region of k-space with an R_{bkg} of 1.2 was employed. The FEFF6 code[5,6] was used for scattering path generation, and multi (k^1, k^2, k^3)-weighted fits of the data were carried out in r-space over r- and k- ranges. The S_0^2 value was set to 0.9, expected value considering the atomic pairs involved to minimize the existing correlations, and a global E_0 was employed with the initial E_0 value set to the first inflection point of the rising edge. Single scattering paths were fit in terms of a Δreff and σ^2, which represent the deviation from the expected interatomic distances and the structural disorder, respectively. To assess the goodness of the fits both the R_{factor} (%R) and the reduced χ^2 (χ^2_v) were minimized, ensuring that the data was not over-fit. Coordination number for best fit model has been

estimated by simulating the EXAFS signal with a progressively increasing grid search where the coordination number was investigated, initially in steps of 0.5, and refined in steps of 0.1 by employing Larch, the Python implementation of Artemis[7]. This is an approach which helps in addressing the natural correlations existing in between the coordination number, the Debye Waller factor, and S_0^2.

1.4. Catalytic activity evaluation. The catalytic activity for CO_2 hydrogenation was evaluated in a fixed-bed tubular reactor with an inner diameter of 11 mm and length of 240 mm, in the temperature range 220-300 °C at 20 bar. A synthetic $CO_2/H_2/N_2$ (23.7/71.3/5, vol%) mixture with molar ratio CO_2/H_2=1/3 to reach a total flow rate 50 mL/min for reaction. Before reaction, PdGa@MFI samples were *in-situ* reduced at 350 °C for 2h under 20 mL/min H_2 flow at atmospheric pressure with a heating rate of 10 °C/min, while for supported PdGa/SiO_2 samples, 500 °C *in-situ* reduction were conducted. Typically, 200 mg catalysts (sieved into 400-600 µm) were diluted with 1650 mg SiC granules (Fisher Scientific, 600-800 µm) to achieve an isothermal packed bed with a weight hour space velocity (WHSV) of 15000 mL·g_{cat}^{-1}·h^{-1}. Various contact times (space velocities) experiments were done on 200 or 1000 mg catalysts by changing the total flow from 150 to 30 mL/min. Stability test was done on 1000 mg $PdGa_4$@MFI-700RED sample at 260 °C, 20 bar and 35 mL/min to reach high CO_2 conversion. The products at the outlet of the reactor were on-line analyzed with Agilent 8860 equipped with a TCD (HP-Plot/Q plus HP-Molesieve) and an FID (HP-Plot/U) detector. All product lines were heated at 150 °C to prevent condensation of products in the lines. Product quantification was performed using chromatographic response factors referenced to N_2 as an internal standard. CO_2 conversion, product selectivity, space time yield (STY), turnover frequencies (TOF), apparent activation energies (Ea) and carbon balance were calculated according to equations 1-6. The elemental balance of carbon was 100 ± 5%. The TOF was calculated using the number of Pd exposed sites by CO chemisorption.

$$Conversion\ (X_{CO_2}) = \left(\frac{F_{CO_{out}} + F_{CH_4{}_{out}} + F_{CH_3OH_{out}} + F_{DME_{out}}}{F_{CO_2{}_{out}} + F_{CO_{out}} + F_{CH_4{}_{out}} + F_{CH_3OH_{out}} + F_{DME_{out}}}\right) * 100\ (\%) \quad (1)$$

$$S_i = \left(\frac{F_{i_{out}}}{F_{CO_{out}} + F_{CH_4{}_{out}} + F_{CH_3OH_{out}} + F_{DME_{out}}}\right) * 100\ (\%) \quad (2)$$

$$STY_i\ (mol \cdot g^{-1} \cdot h^{-1}) = \frac{V_{CO_2} * X_{CO_2} * S_i * 60}{V_m * m_{cat}} \quad (3)$$

$$TOF\ (h^{-1}) = \frac{STY_i * M_{Pd}}{m_{cat} * D_{Pd} * W_{Pd}} \quad (4)$$

$$Ln(STY_i) = -\frac{E_{a_i}}{R}\frac{1}{T} + C \quad (5)$$

$$Carbon\ balance = \frac{Total\ carbon\ in\ products}{Total\ carbon\ in\ feed\ components} \quad (6)$$

Where F_i stands for the molar flow of i-compound based on N_2 internal standard using calibrated response factors. V_{CO2} is the flow of CO_2, V_m is the molar volume of ideal gas at standard temperature and pressure, M_{Pd} is the molar mass of Pd, D_{Pd} is the dispersion of Pd, W_{Pd} is the loading weight of Pd on catalyst, R is molar gas constant. For DME STY with $g_{DME}\cdot kg_{cat}^{-1}\cdot h^{-1}$ unit, molar mass of DME (46.07 $g\cdot mol^{-1}$) is multiplied with equation (3), same calculation principle is applied for methanol and CO. Initial formation rates and selectivities were extrapolated using a second-order polynomial fit of the various contact time experimental data. At each presented experiment data, reaction was last for 1h to reach steady state with three points for average.

Kinetic experiments on the PdGa catalysts were conducted at 260 °C and 100 mL/min (WHSV=30000 $mL\cdot g_{cat}^{-1}\cdot h^{-1}$) with the CO_2 conversions below 5%. Same set-up and procedures were used as the activity evaluations. In the reaction order of H_2, the CO_2 pressure was kept at 4.74 bar, while the H_2 pressure was changed from 2.37 to 14.26 bar with CO_2/H_2 molar ratio range from 2/1 to 1/3. In the reaction order of CO_2, the H_2 pressure was kept at 9.5 bar, while the CO_2 pressure was changed from 1.9 to 9.5 bar with CO_2/H_2 molar ratio range from 1/5 to 1/1. The apparent activation energy was determined by measuring steady-state reaction rates between 220 and 300 °C at intervals of 20 °C. All the external diffusion, internal diffusion, and heat transfer limitations are neglected in our system, which are verified in below calculations.

1.5. Exclude mass and heat transfer limitations.

External mass diffusion calculation:

In this study, fixed-bed reactor is assimilated to an isothermal and isobaric plug flow reactor. The mass transfer between reactants and catalyst particle outer surface follows below equations under continuous steady process. D_a: Damköhler number, which is the ratio between reaction rate to external diffusion rate, reflecting the influence extent of external diffusion during reaction[8,9].

$$\frac{d_{n_A}}{d_t} = k_g S_S \varphi (C_{Ag} - C_{AS}) \quad 1$$

$$\frac{d_{n_A}}{d_t} = k_g S_S \varphi (C_{Ag} - C_{AS}) = (-R_A)V_S = \eta V_S k f(C_{AS}) \quad 2$$

$$C_{Ag} - C_{AS} = \frac{\eta V_S k}{k_g S_S \varphi} f(C_{AS}) = D_a f(C_{AS}) \quad 3$$

$$D_a = \frac{\eta V_S k}{k_g S_S \varphi} = \frac{(-R_A)V_S}{k_g S_S \varphi} \quad 4$$

$$J_D = \frac{k_g \rho_g}{G} (S_C)^{\frac{2}{3}} \quad 5$$

$$Re_m = \frac{d_S G}{\varphi \mu_g (1 - \varepsilon_B)} \quad 6$$

J_D is the function to Re_m,

0.3<Re_m<300, $J_D = 2.10 {Re_m}^{-0.51}$,

300<Re_m<6000, $J_D = 1.19 {Re_m}^{-0.41}$.

$\frac{d_{n_A}}{d_t}$: mole number of reactant A transfer at unit time, mol·s^{-1};

k_g: mass transfer coefficient between gas and the surface of catalyst particle, cm·s^{-1};

C_{Ag} and C_{AS}: reactant A concentration at gas phase and catalyst surface, mol·cm^{-3};

S_S: catalyst surface area, cm^2;

φ: effectiveness factor of catalyst particle surface, for sphere particles φ=1;

R_A: reaction rate, mol·g_{cat}^{-1}·h^{-1};

η: effectiveness factor during reaction;

k: reaction constant;

J_D: mass transfer coefficient;

ρ_g: gas density, 6.08×10^{-3} g·cm^{-3};

G: gas mass flow, 5.33×10^{-3} g·cm^{-2}·s^{-1};

μ_g: gas mixture viscosity, 1.65×10^{-4} g·cm^{-1}·s^{-1};

S_C: Schmidt number, $S_C = \mu_g/(\rho_g D_{CO_2,m})$;

$D_{CO_2,m}$: diffusion coefficient of CO_2 in gas mixture, 0.6 cm^2·s^{-1};

Re_m: Reynolds number;

d_S: catalyst particle area-equivalent diameter, 0.05 cm;

ε_B: bed porosity, 0.57.

For the most active $PdGa_4$@MFI-700RED sample, at 260 °C and 20 bar with WHSV of 15000 mL·g_{cat}^{-1}·h^{-1} reaction condition:

$$-R_A = 6.83\ mmol_{CO_2} \cdot g_{cat}{}^{-1} \cdot h^{-1} \quad (\text{TOF}=575.27\ \text{h}^{-1})$$

$$S_S = 4\pi d^2 = 4\pi \times (0.05/2)^2 = 7.85 \times 10^{-3}\ cm^2$$

$$V_S = \frac{4}{3}\pi d^3 = \frac{4}{3}\pi \times (0.05/2)^3 = 6.54 \times 10^{-5}\ cm^3$$

$$Re_m = 3.76$$

$$J_D = 2.10 Re_m{}^{-0.51} = 1.0687$$

$$k_g = \frac{J_D G}{\rho_g} \left(\frac{\mu_g}{\rho_g D}\right)^{-\frac{2}{3}} = 7.38\ cm \cdot s^{-1}$$

$$D_a = \frac{(-R_A)V_S}{k_g S_S \varphi} = 1.80 \times 10^{-4}$$

Thus, the limitation of external mass diffusion is negligible in our reaction condition.

Internal mass diffusion calculation:

Weisz-Prater number relates the reaction rate with respect to the diffusion in the pores of particles. If it is less than 1, the internal mass diffusion limitation is negligible[8,9].

$$C_{WP} = \frac{-R_A \rho_c d_S{}^2}{D_{eff} C_{AS}} < 1 \qquad 7$$

$$D_{eff} = D_{AB}\frac{\varepsilon_P}{\tau} \quad 8$$

$$D_{AB} = 0.436\frac{T^{1.5}\left(\frac{1}{M_{CO_2}}+\frac{1}{M_{H_2}}\right)^{0.5}}{P\left(V_{CO_2}^{\frac{1}{3}}+V_{H_2}^{\frac{1}{3}}\right)^2} \quad 9$$

ρ_c: solid density of catalyst, 0.9 g·cm^{-3};

d_S: catalyst particle area-equivalent diameter, 0.05 cm;

D_{AB}: dual molecule diffusion, cm^2·s^{-1};

ε_P: pellet porosity, 0.4;

τ: tortuosity, 3;

T: temperature, 260 °C (533 K);

P: pressure, 20 bar (2000 kPa);

M_{CO_2} and M_{H_2} are molecular weight of CO_2 and H_2;

V_{CO_2} and V_{H_2} are CO_2 and H_2 diffusion volume, which are 26.9 and 7.07 cm^3·mol^{-1}.

For the most active $PdGa_4$@MFI-700RED sample, at 260 °C and 20 bar with WHSV of 15000 mL·g_{cat}^{-1}·h^{-1} reaction condition:

$$-R_A = 6.83\ mmol_{CO_2}\cdot g_{cat}{}^{-1}\cdot h^{-1}$$

$$D_{AB} = \frac{160.53}{P} = 0.08027\ cm^2\cdot s^{-1}$$

$$D_{eff} = 0.01070\ cm^2\cdot s^{-1}$$

$$C_{AS} = \frac{P_{CO_2}}{RT} = \frac{20\times 0.238}{8.314\times(273+260)} = 1.074\times 10^{-4}\ mol\cdot cm^{-3}$$

$$C_{WP} = \frac{6.83\times 10^{-3}\times 0.9\times 0.05^2}{3600\times 0.01070\times 1.074\times 10^{-4}} = 3.7146\times 10^{-3} < 1$$

Thus, the limitation of internal mass diffusion is negligible in our reaction condition.

External heat transfer calculation:

According to Mears' criterion[10], heat transfer limitation is negligible when radial heat transfer gradient ΔT_{rad}:

$$\Delta T_{rad} = \frac{-R_A|\Delta H^0|(1-\varepsilon_B)\rho_B(1-B){d_t}^2}{32\lambda_{er}} < \frac{0.05R{T_W}^2}{E_a} \quad 10$$

ΔH^0: reaction enthalpy, herein using directly CO_2 hydrogenation to DME value, -122 KJ·mol^{-1};

B: volume fraction of inert materials, SiC, 0.5;

ρ_B: catalyst bed density, 0.38 g·cm^{-3};

d_t: catalyst bed diameter, 0.055 cm;

λ_{er}: effective radial thermal conductivity in the bed, 1.53 W·m^{-1}·K^{-1};

T_W: reactor wall temperature, 260 °C (533K);

R: gas constant, 8.314 J·mol^{-1}·K^{-1};

E_a: apparent activation energy of CO_2 to methanol and DME, ~50 kJ·mol^{-1}.

For the most active $PdGa_4$@MFI-700RED sample, at 260 °C and 20bar with WHSV of 15000 mL·g_{cat}^{-1}·h^{-1} reaction condition:

$$-R_A = 6.83\, mmol_{CO_2} \cdot {g_{cat}}^{-1} \cdot h^{-1}$$

$$\Delta T_{rad} = 1.1684 \times 10^{-4}\, K < 2.3619\, K$$

Thus, the limitation of heat transfer is negligible in our reaction condition.

2. Synthesis of PdGa@MFI samples

Literature reported metal complexing agent as ethylenediamine (EDA)[11], (3-mercaptopropyl)trimethoxysilane (MPTMS)[12] are used for the synthesis of PdGa@MFI samples. The synthesis illustration is displayed in Figure S1. As shown in Figure S3 and 4, partially Pd precipitation is inevitable. EDX mapping or point analysis indicate the white counterpart is related to Pd precipitates.

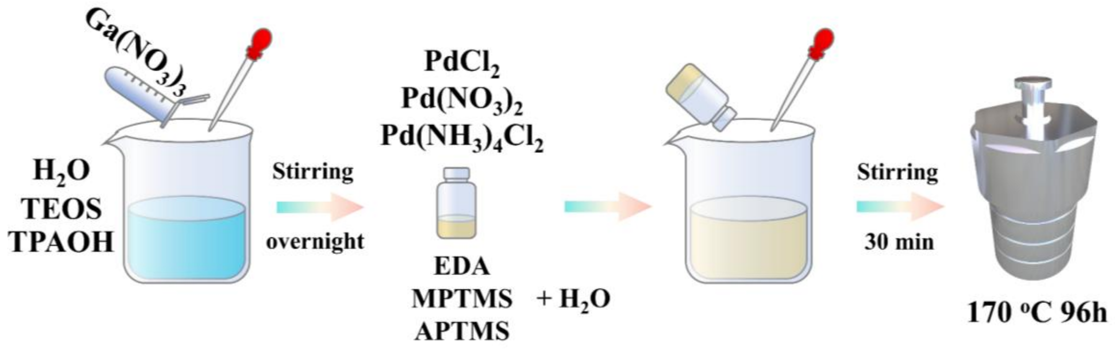

Figure S1. Synthesis illustration of different Pd salt with EDA/MPTMS/APTMS complexing agents (EDA with $PdCl_2$, MPTMS with $Pd(NO_3)_2$ and APTMS with $Pd(NH_3)_4Cl_2$).

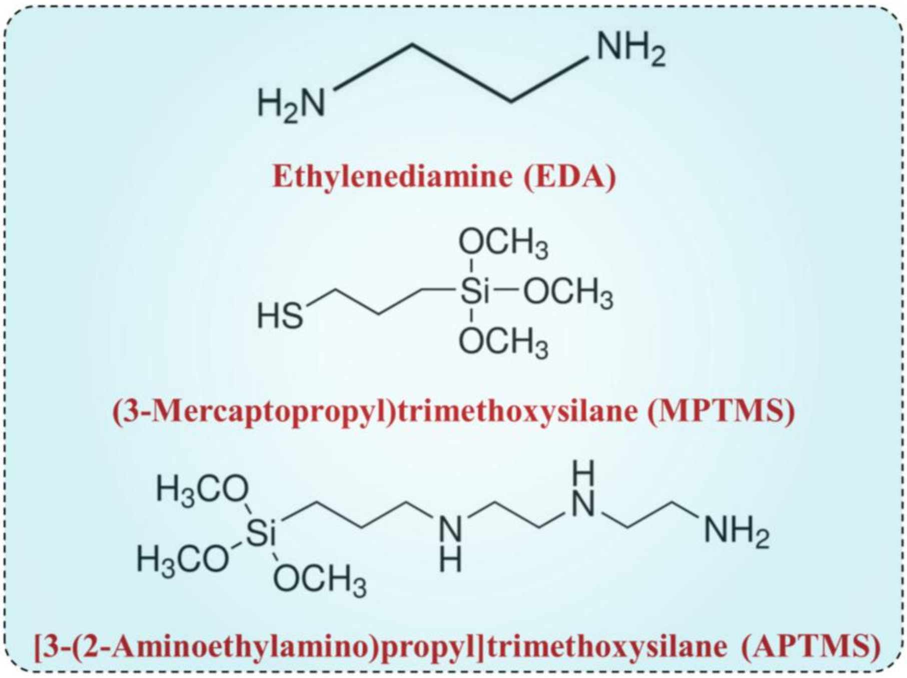

Figure S2. Chemical formula of EDA, MPTMS and APTMS.

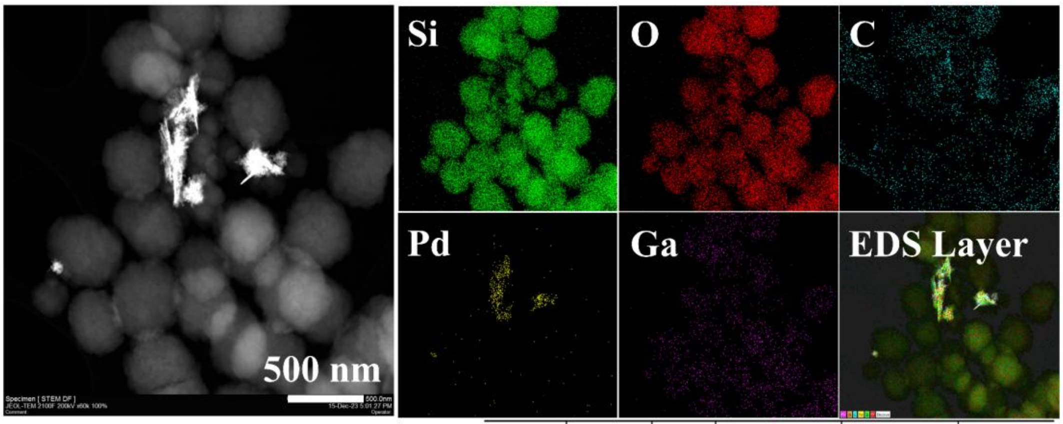

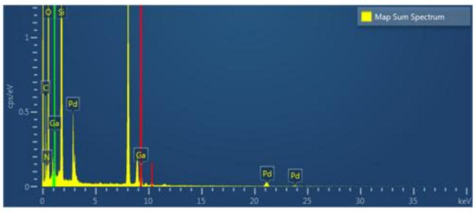

Element	Line Type	k Factor	k Factor type	Absorption Correction	Wt%
C	K series	2.769	Theoretical	1	5.15
O	K series	2.02	Theoretical	1	40.7
Si	K series	1	Theoretical	1	46.5
Ga	K series	1.369	Theoretical	1	2.4
Pd	K series	9.167	Theoretical	1	5.25
Total:					100

Figure S3. Pd-EDA method for the synthesis of PdGa@MFI (after synthesis).

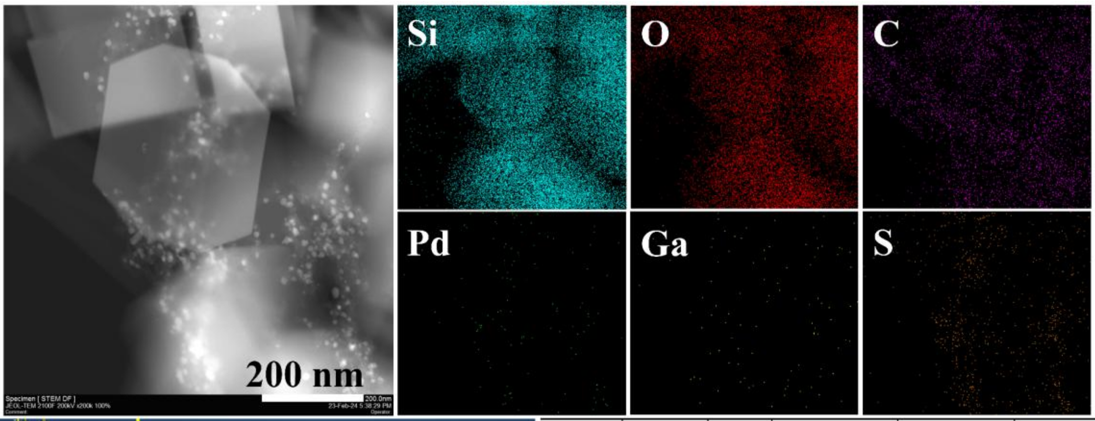

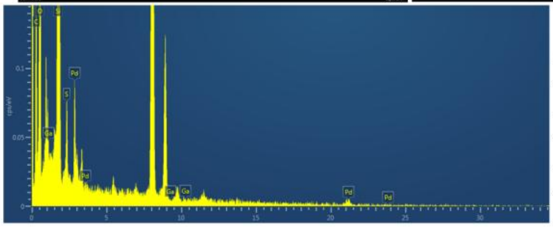

Element	Line Type	k Factor	k Factor type	Absorption Correction	Wt%
C	K series	2.769	Theoretical	1	23.34
O	K series	2.02	Theoretical	1	41.75
Si	K series	1	Theoretical	1	34.28
S	K series	1	Theoretical	1	0.24
Ga	K series	1.369	Theoretical	1	0.03
Pd	K series	9.167	Theoretical	1	0.37

Figure S4. Pd-MPTMS method for the synthesis of PdGa@MFI (after synthesis).

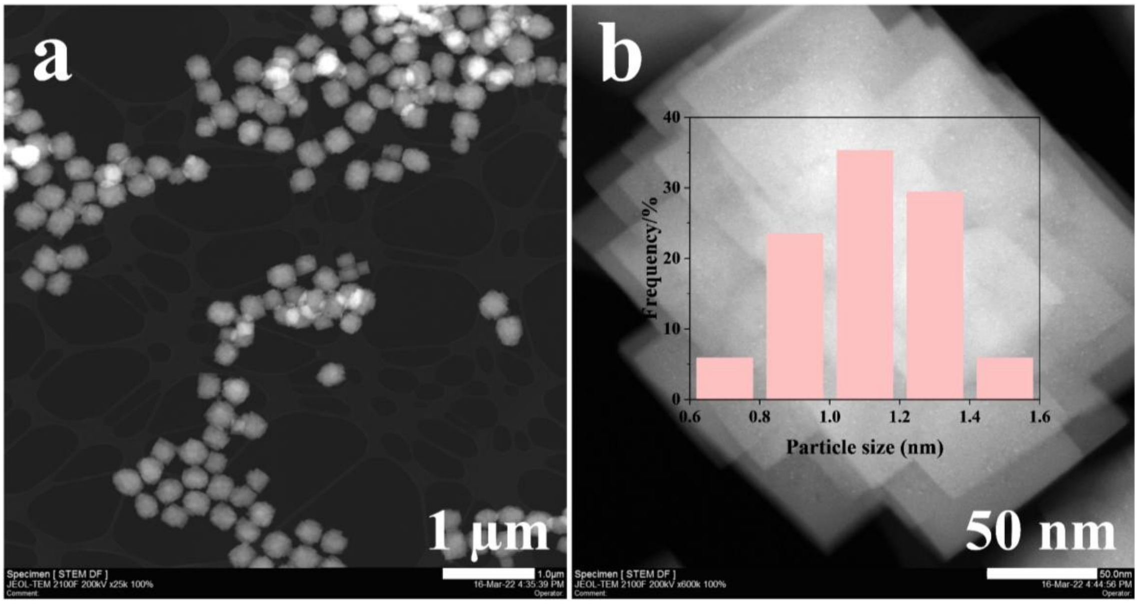

Figure S5. Pd-APTMS method for the synthesis of $PdGa_4$@MFI (after synthesis).

Comments:

For [3-(2-Aminoethylamino)propyl]trimethoxysilane (APTMS) as complexing agent, after synthesis a uniform zeolite precipitant is observed by keeping in a plastic bottle overnight, while in the case of failed PdGa@MFI (EDA and MPTMS method prepared catalysts) a thick black layer is formed at the bottom, which is due to the big bulk Pd precipitates formation. (Figure S6).

Besides, APTMS is susceptible to recrystallization or decomposition if it is stored in air for long time, as displayed in Figure S7. In this case the color of the $Pd(NH_3)_4Cl_2 \cdot H_2O$ complex with APTMS change from light yellow to deep yellow, leading to the formation of a layer of black floats on the liquid surface after the zeolite synthesis, which are carbon-related compounds. Such bad APTMS could also decrease the encapsulation efficiency of Pd inside zeolite. Thus, using fresh APTMS is necessary to get high quality PdGa@MFI catalyst.

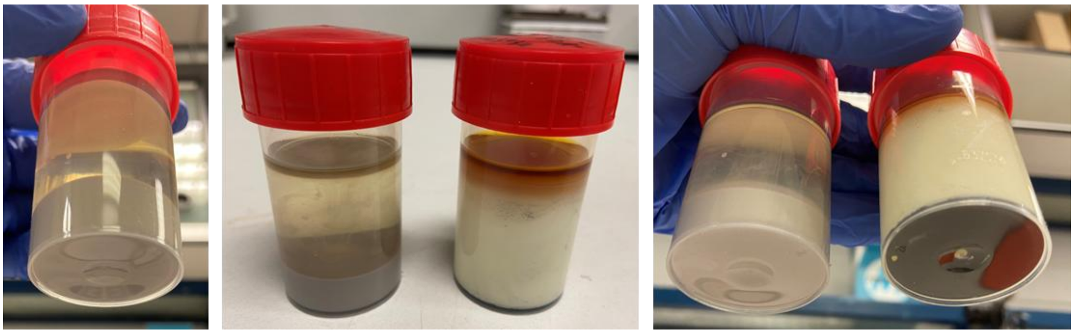

Figure S6. Photos of PdGa@MFI after synthesis and keep in plastic bottle overnight.

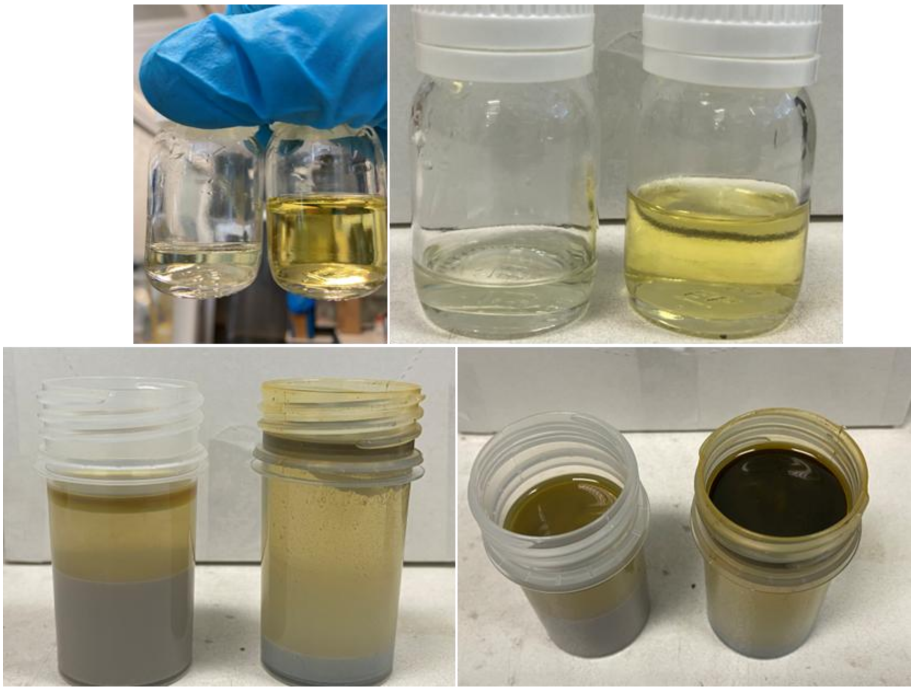

Figure S7. Photos of Pd-APTMS and PdGa@MFI after synthesis and keep in plastic bottle overnight (left glass or plastic bottle is good quality and right glass or plastic bottle is bad quality).

<u>Comments:</u>

The choice of the ligand plays a critical role in stabilizing Pd species during the hydrothermal synthesis of the PdGa@MFI samples. Compared with the conventional $PdCl_2$-ethylenediamine (EDA) system, the use of 3-[2-(2-25 aminoethylamino)ethylamino]propyltrimethoxysilane (silane-triamine) together with $Pd(NH_3)_4Cl_2$ affords a much higher quality of the PdGa@MFI materials. This improvement can be rationalized by several factors:

- **Covalent anchoring effect.**

The trimethoxysilyl groups of silane-triamine can hydrolyze and co-condense with TEOS under alkaline hydrothermal conditions, covalently incorporating the Pd-ligand complex into the growing silica framework. This prevents migration of Pd complexes into the bulk solution and suppresses uncontrolled precipitation. By contrast, ethylenediamine lacks such anchoring groups and therefore cannot integrate into the silica network.

- **Chelation stability.**
  Diethylenetriamine provides a stronger multidentate coordination environment than ethylenediamine. Together with the labile NH_3 ligands of $Pd(NH_3)_4Cl_2$, this allows for the rapid formation of stable Pd-triamine complexes that resist hydrolysis or ligand substitution under strongly basic, high-temperature synthesis conditions. In contrast, $PdCl_2$-EDA complexes may undergo partial hydrolysis or aggregation, leading to the precipitation of $Pd(OH)_2$ or PdO.

- **Spatial confinement during crystallization.**
  Once the silane-triamine is covalently bound to the silica framework, the Pd species are physically confined within the silanol nests or pore environment of MFI during nucleation and crystal growth. This restricts Pd diffusion and aggregation, thereby favoring the formation of highly dispersed Pd species within the zeolite matrix.

- **Controlled reduction and nucleation.**
  The stronger chelating environment and steric hindrance provided by the triamine ligand modulate the reduction kinetics of Pd(II), suppressing uncontrolled nucleation and particle growth. This further contributes to the uniform dispersion and confinement of Pd nanoparticles.

Taken together, these effects explain why the silane-triamine-$Pd(NH_3)_4Cl_2$ system effectively avoids Pd precipitation and enables the encapsulation of well-dispersed Pd species in MFI, while the conventional $PdCl_2$-EDA system often suffers from partially Pd loss and particle aggregation under identical hydrothermal conditions.

On the other hand, to support that this methodology allows the stabilization of Pd particles inside the zeolite channel, we performed phenylacetylene and diphenylacetylene semi-hydrogenation reaction on Pd@MFI-700RED sample as test reaction. It is reported

Pd@MFI catalyst is efficient for phenylacetylene semi-hydrogenation due to the appropriate molecular kinetic diameter that allows its diffusion through the MFI zeolite channels[13]. However, diphenylacetylene, possessing a considerably larger molecular dimension, cannot access the internal channels of the MFI framework. Figure S8 clearly demonstrates the successful encapsulation of Pd within the MFI zeolite framework, as reflected by the near-zero catalytic activity observed for diphenylacetylene semi-hydrogenation, which is consistent with the molecular size exclusion effect of the zeolite channels.

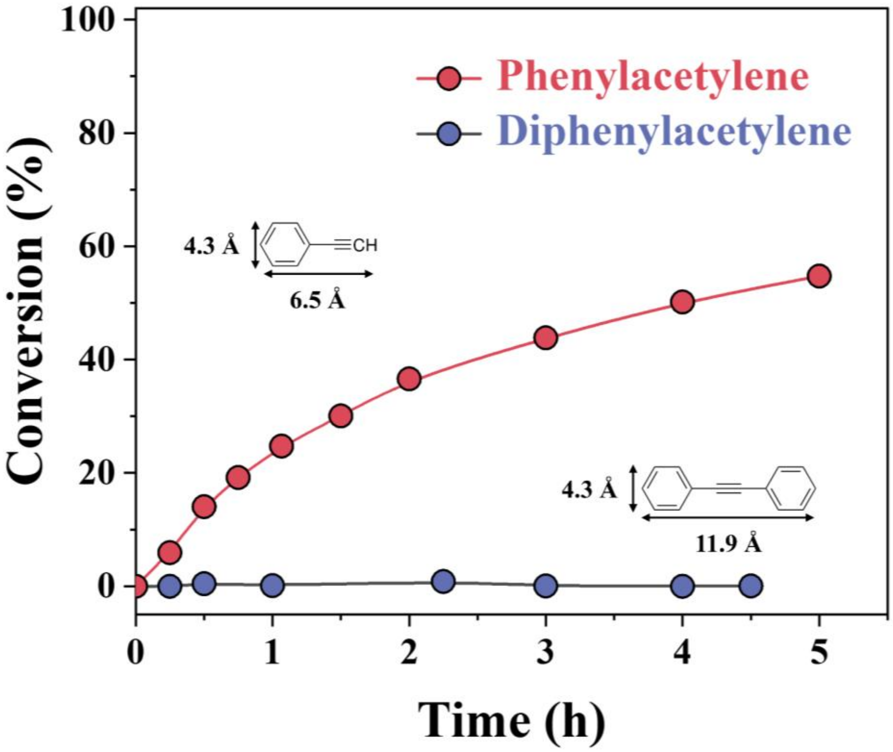

Figure S8. Phenylacetylene and diphenylacetylene hydrogenation on Pd@MFI-700RED sample. Reaction condition: 10 mg catalyst, 1 mmol phenylacetylene or diphenylacetylene, 2g hexane as solvent, 2 bar H_2, 65 °C and 800 rpm.

3. Physico-chemical characterization of $PdGa_x$@MFI samples

3.1. NMR

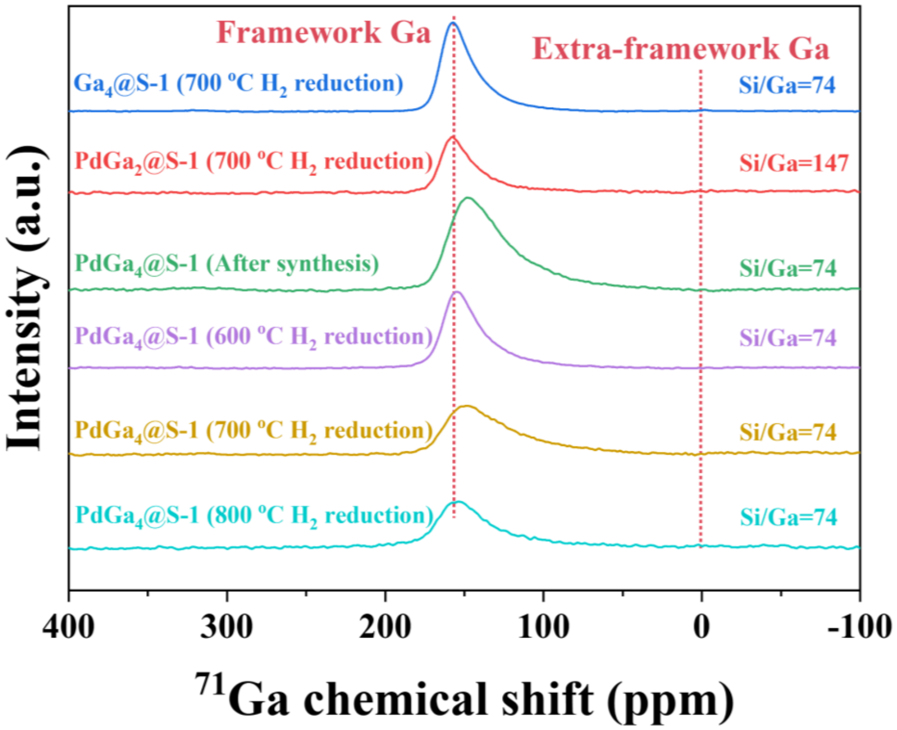

Figure S9. ^{71}Ga-NMR results of $PdGa_x$@MFI samples after synthesis and reduced at different temperatures.

^{17}Ga-NMR results indicate the absence of extra-framework Ga in the samples under study.

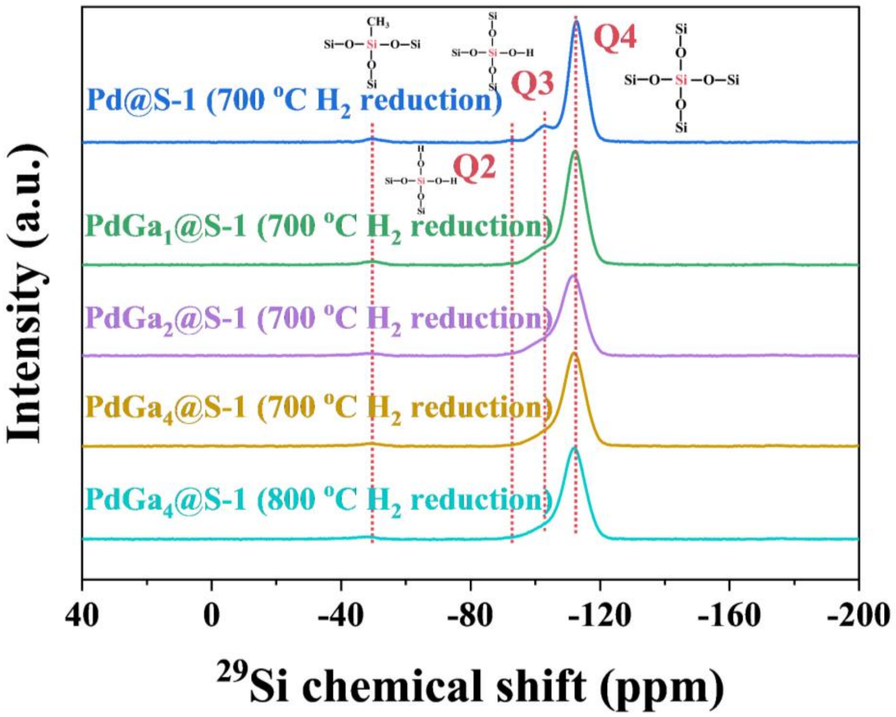

Figure S10. ^{29}Si-NMR results of $PdGa_x$@MFI reduced samples at different temperatures.

<u>Comments</u>:

The peak at -49 ppm is due to $-CH_3$ group coordinate with Si, originating from residual traces of the complexing agent APTMS. ^{29}Si-NMR results indicate that partially Ga occupies the silanol groups after reduction, based on the reduction of the peak at -102 ppm (Q3) at increasing Ga loading in the sample. This behaviour is also confirmed by Pyridine-IR results.

3.2. H_2-TPR

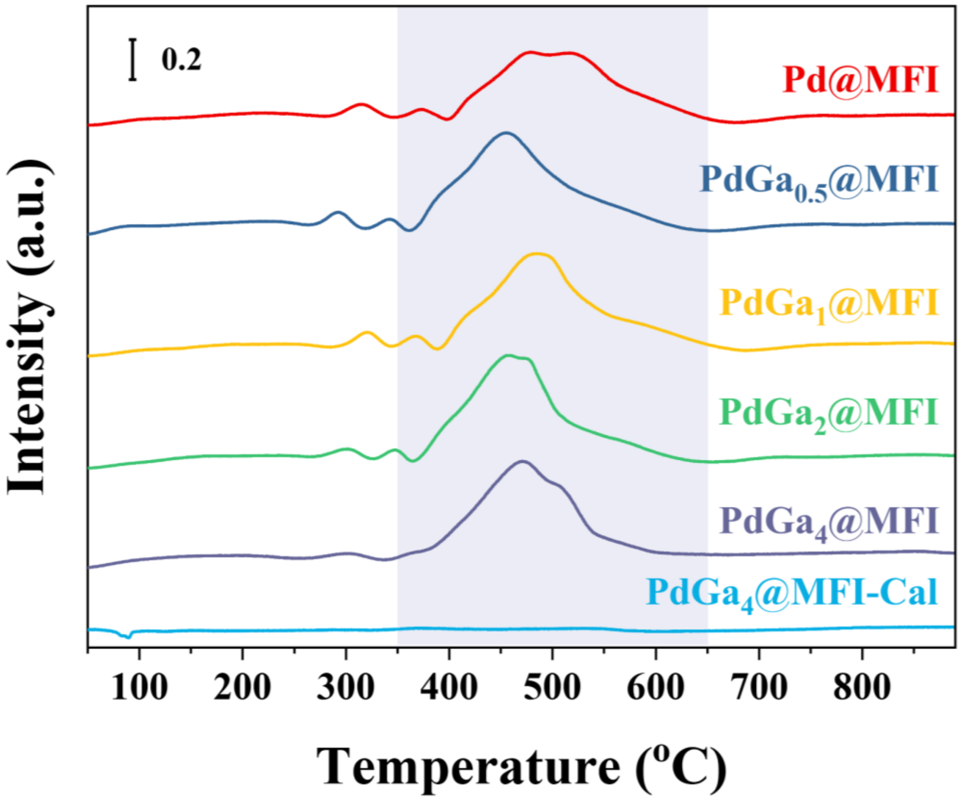

Figure S11. H_2-TPR of as synthesized $PdGa_x$@MFI samples.

<u>Comments</u>:

For all fresh PdGa@MFI catalysts, a prominent peak is observed within the temperature range of 350-700 °C. In contrast, the calcined $PdGa_4$@MFI catalyst exhibits only a single negative peak below 100 °C, corresponding to the reduction of PdO to Pd. Consequently, the significant peak observed above 350 °C in the H_2-TPR study of fresh (i.e., un-calcined) samples is attributed to the removal of the organic template.

3.3. Physical-chemical property of studied catalysts

Table S1. Physical-chemical property of $PdGa_x$@MFI samples.

Sample	Surface area (m^2/g)	V_{micro} (cm^3/g)	C[a]	N[a]	H[a]	Si/Ga[b]	Ga/Pd[b]	ICP Pd (wt%)[c]	ICP Ga (wt%)[c]	Actual Ga/Pd[d]
Ga_4@MFI-700RED	354.0	0.162	0.06	0	0.33	74	-	-	1.52	-
Pd@MFI-700RED	381.4	0.18	0.06	0	0.29	-	-	0.70	-	-
$PdGa_{0.5}$@MFI-700RED	395.4	0.19	0.03	0	0.32	592	0.5	0.68±0.10	0.19±0.01	0.4
$PdGa_1$@MFI-700RED	389.1	0.18	0.06	0	0.36	296	1	0.70±0.10	0.40±0.02	0.9
$PdGa_2$@MFI-700RED	388.9	0.18	0.06	0	0.44	148	2	0.64±0.05	0.76±0.05	1.8
$PdGa_4$@MFI-500RED	379.8	0.18	0.24	0	0.65	74	4	0.69	1.59	3.5
$PdGa_4$@MFI-600RED	371.7	0.15	0.04	0	0.68	74	4	0.62	1.63	4.0
$PdGa_4$@MFI-700RED	371.3	0.18	0.04	0	0.56	74	4	0.60±0.03	1.60±0.04	4.1
$PdGa_4$@MFI-700RED[e]	-	-	0.27	0	0.56	74	4	0.60	1.60	4.1
$PdGa_4$@MFI-800RED	375.2	0.17	0.07	0	0.82	74	4	0.59	1.50	3.9

[a] C, N and H is analysed by elemental analysis;
[b] Theoretical Si/Ga and Ga/Pd molar ratio;
[c] Actual Pd and Ga weight loading by ICP;
[d] Actual Si/Ga and Ga/Pd molar ratio;
[e] $PdGa_4$@MFI-700RED sample after >70h stability test.

4. Effect of the reduction temperature on the $PdGa_x$@MFI sample

The effect of the reduction temperature on the structural morphologic and chemical properties of the final material has been systematically investigated on the $PdGa_4$@MFI sample and discussed in this section using a variety of characterization methods.

4.1. Pyridine-IR on $PdGa_4$@MFI and Ga_4@MFI samples

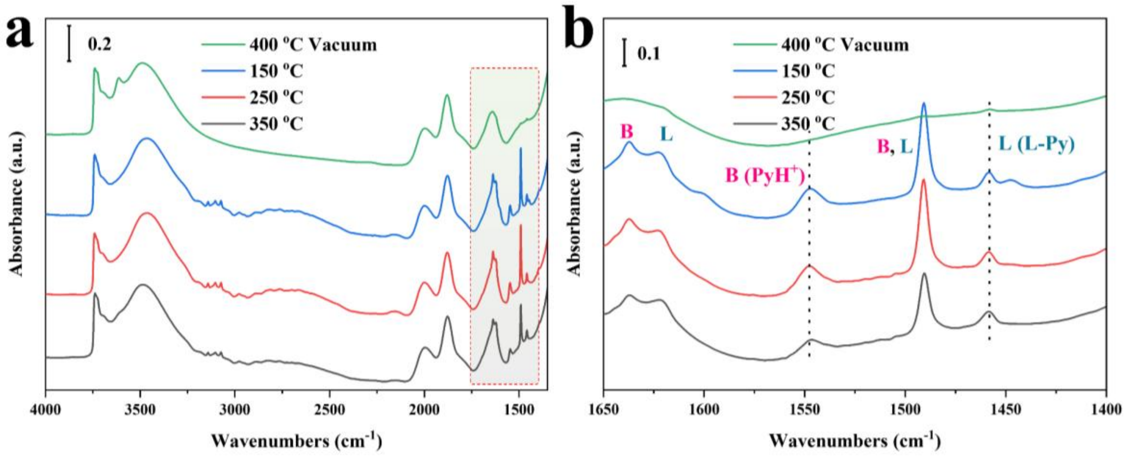

Figure S12. Pyridine-IR of Ga_4@MFI-Cal (a) full scale wavenumbers from 4000 to 1300 cm^{-1}, (b) enlarged pyridine adsorption region in (a).

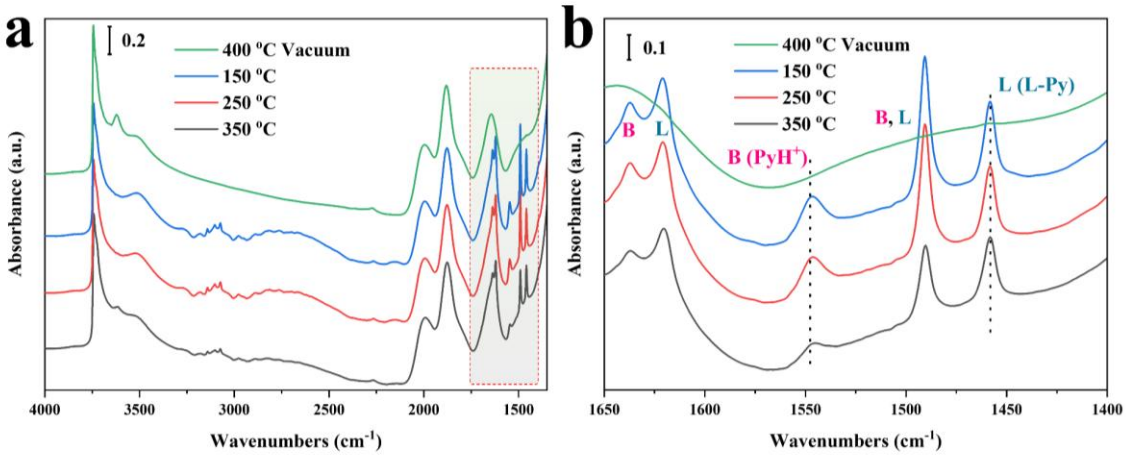

Figure S13. Pyridine-IR of Ga_4@MFI-700RED (a) full scale wavenumbers from 4000 to 1300 cm^{-1}, (b) enlarged pyridine adsorption region in (a).

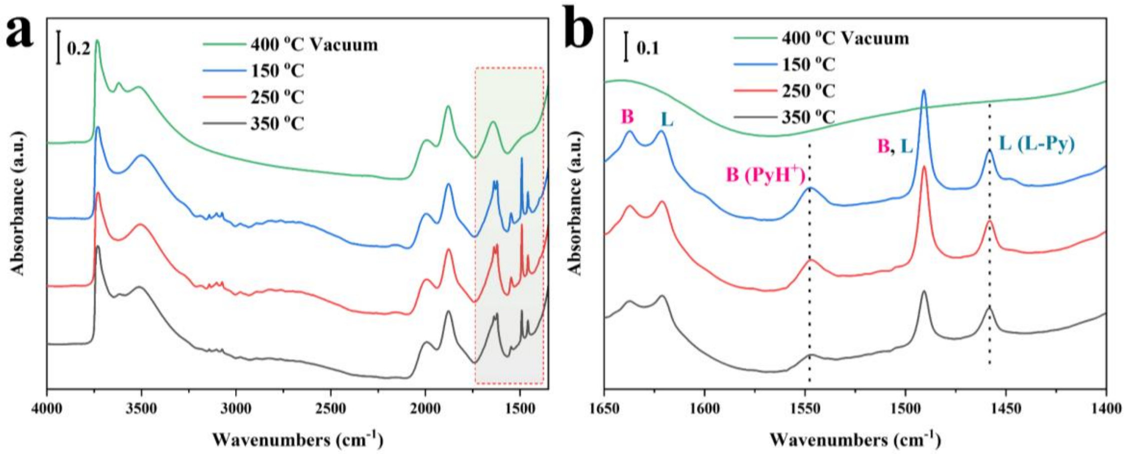

Figure S14. Pyridine-IR of $PdGa_4$@MFI-Cal (a) full scale wavenumbers from 4000 to 1300 cm^{-1}, (b) enlarged pyridine adsorption region in (a).

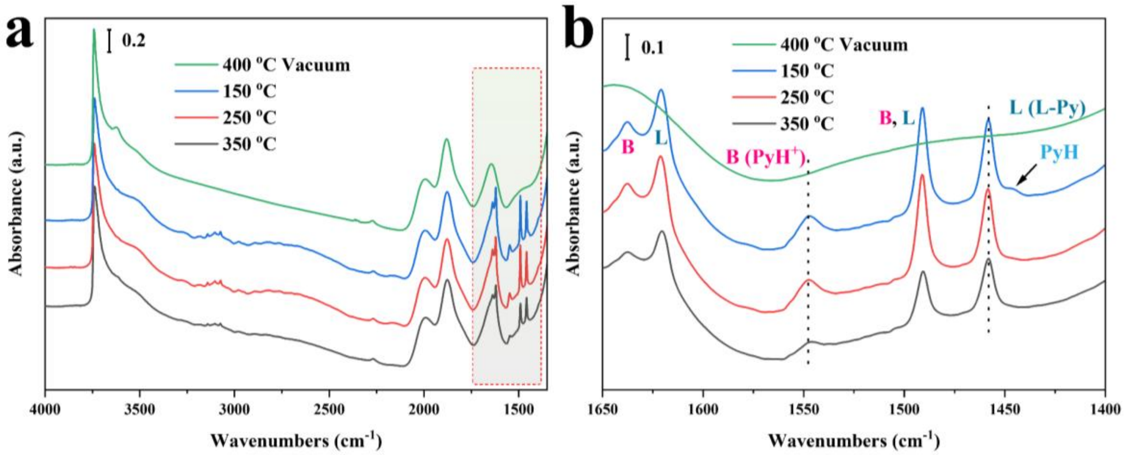

Figure S15. Pyridine-IR of $PdGa_4$@MFI-Cal700RED (a) full scale wavenumbers from 4000 to 1300 cm^{-1}, (b) enlarged pyridine adsorption region in (a).

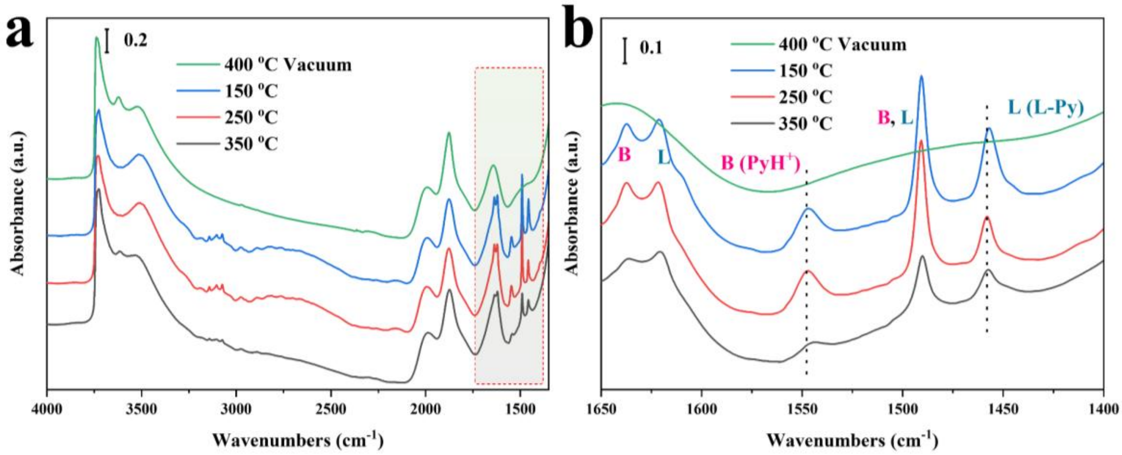

Figure S16. Pyridine-IR of $PdGa_4$@MFI-500RED (a) full scale wavenumbers from 4000 to 1300 cm^{-1}, (b) enlarged pyridine adsorption region in (a).

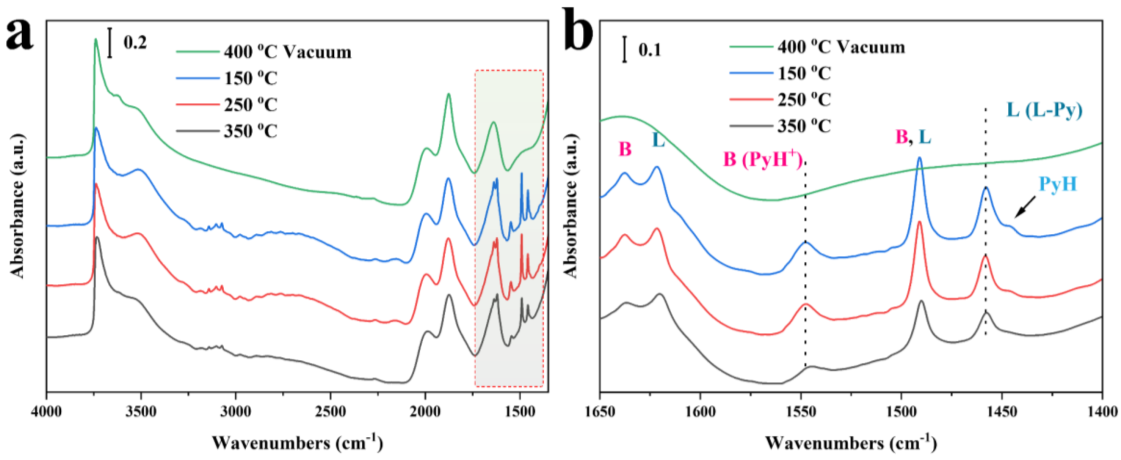

Figure S17. Pyridine-IR of $PdGa_4$@MFI-600RED (a) full scale wavenumbers from 4000 to 1300 cm^{-1}, (b) enlarged pyridine adsorption region in (a).

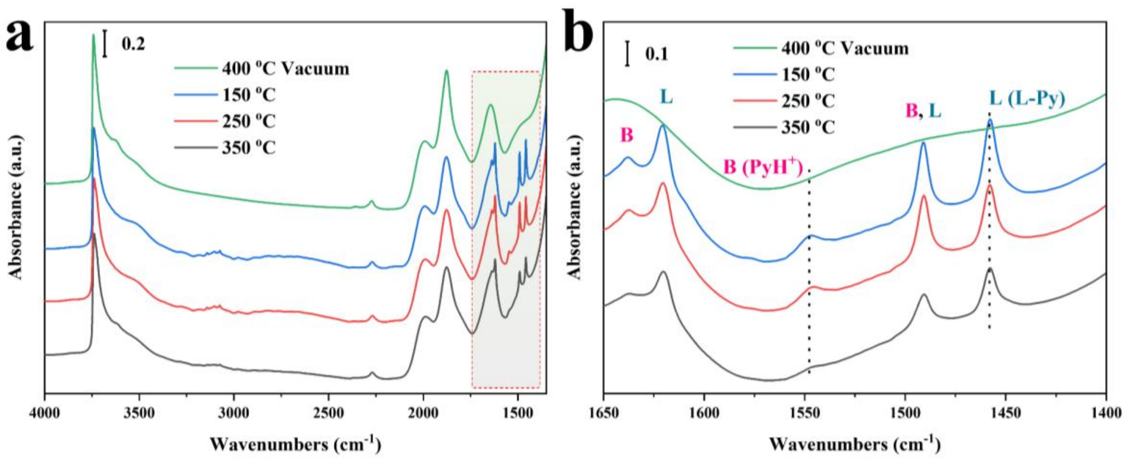

Figure S18. Pyridine-IR of $PdGa_4$@MFI-700RED (a) full scale wavenumbers from 4000 to 1300 cm^{-1}, (b) enlarged pyridine adsorption region in (a).

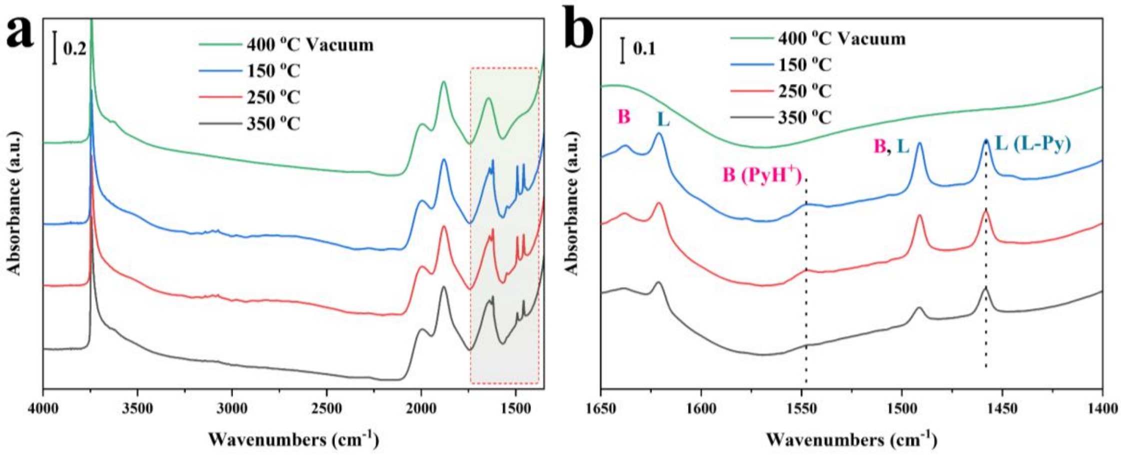

Figure S19. Pyridine-IR of $PdGa_4$@MFI-800RED (a) full scale wavenumbers from 4000 to 1300 cm^{-1}, (b) enlarged pyridine adsorption region in (a).

Table S2. Relative Brønsted and Lewis acidity on Ga_4@MFI and $PdGa_4$@MFI samples after different post-treatment conditions determined by IR of pyridine.[a]

Sample	Actual Ga loading	Overtone area[b]	Brønsted acid area	Lewis acid area	Relative B/O[c]	Relative L/O[c]	Framework Ga^{3+} [d]	Error
Ga_4@MFI-Cal	1.60 wt%	77.58	2.48	0.73	0.0320	0.0094	-	±3%
Ga_4@MFI-700RED	1.52 wt%	105.04	2.35	2.47	0.0223	0.0235	~70%	±7%
$PdGa_4$@MFI-Cal	1.70 wt%	47.99	1.45	0.95	0.030	0.0197	~94%	± 7%
$PdGa_4$@MFI-Cal700RED	1.70 wt%	102.32	1.61	2.50	0.01576	0.0244	~49%	± 7%
$PdGa_4$@MFI-500RED	1.59 wt%	39.53	1.18	0.60	0.0298	0.0151	~93%	±7%
$PdGa_4$@MFI-600RED	1.63 wt%	78.53	1.31	1.78	0.0167	0.0226	~52%	±7%
$PdGa_4$@MFI-700RED	1.60 wt%	81.05	0.81	1.67	0.00996	0.0206	~31%	± 7%
$PdGa_4$@MFI-800RED	1.50 wt%	66.72	0.63	1.48	0.00937	0.0221	~29%	± 7%

[a] Relative Brønsted acidity are based on 150 ^{o}C pyridine desorption.
[b] Zeolite overtone peak area.
[c] B/O = area of the Brønsted acid sites (B) normalized to the area corresponding to the overtone (O) of the sample, L/O = area of the Lewis acid sites (L) normalized to the area corresponding to the overtone (O) of the sample.
[d] Estimative analysis of the percent of Ga^{3+} species in framework positions calculated based on the Brønsted acidity of the corresponding sample, assuming that framework Ga^{3+} species are compensated by a proton (H^+). For this analysis we used Ga_4@MFI-Cal as reference sample for 100% incorporation of Ga^{3+}in the framework.

Comments:

The migration of Ga^{3+} species is supported by Pyridine-IR. The Pyridine-IR spectra reveal the presence of Brønsted acid sites, clearly indicating the location of Ga^{3+} ions at framework positions within the zeolite. Consequently, the loss of Brønsted acidity in the Pyridine-IR signal serves as a reliable indicator of Ga^{3+} detachment from these framework positions. Interestingly, while almost 70% of Ga^{3+} remains in framework positions in the Ga_4@MFI-700RED sample, it decreases to ~31% in the $PdGa_4$@MFI-700RED sample, behaving Pd as driving force for Ga^{3+} migration. (Table S2, Figure S12-19). Based on the data of Table S2, Ga^{3+} migration is promoted with the temperature, which is in line with

Energy-dispersive X-ray spectroscopy (EDX) data shown below. Moreover, the peak at ~3500 cm^{-1} which is associated to hydroxyl nests, decrease by increasing the reduction temperature (Figure S20). This indicates that, in addition to partially Ga being alloyed with Pd, a portion of Ga^{3+} are redistributed to occupy framework defects as isolated Ga^{+} Lewis acid sites (Figure S21).

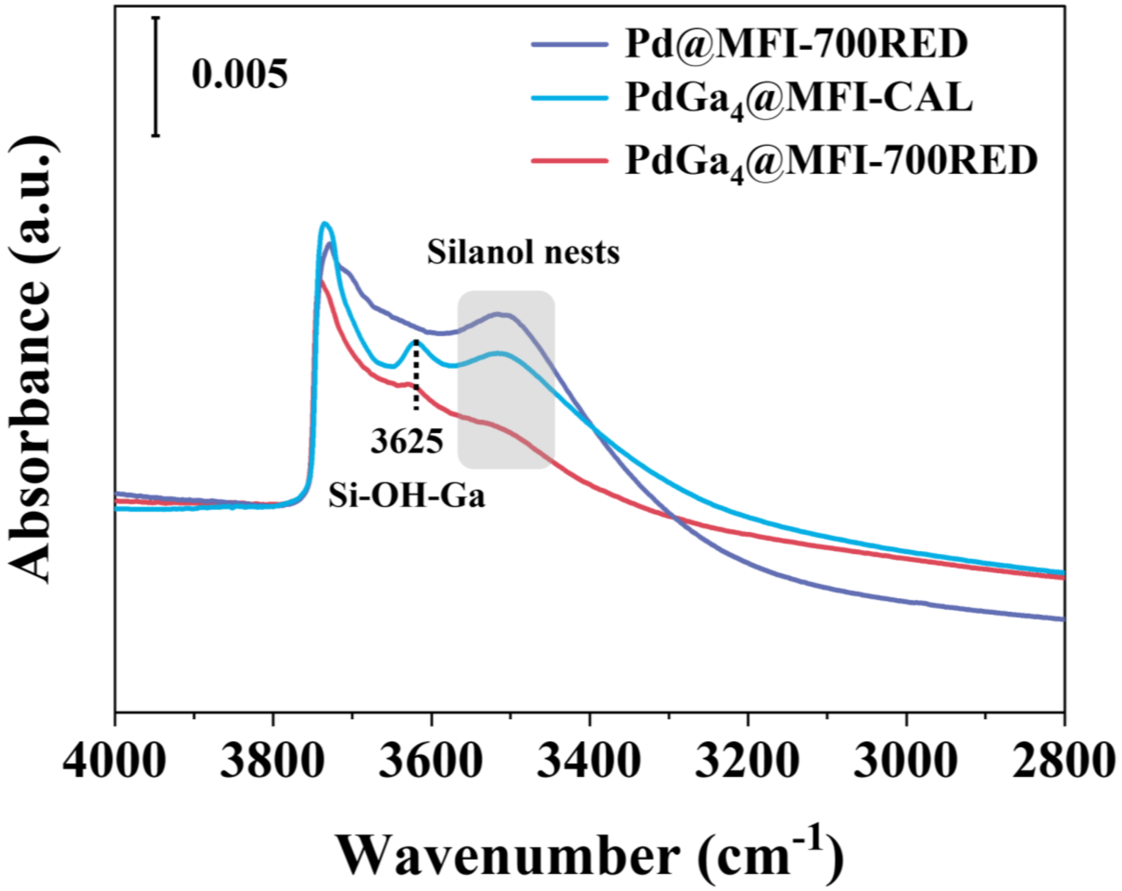

Figure S20. IR spectra in the silanol stretching region of Pd@MFI-700RED, $PdGa_4$@MFI-CAL, and $PdGa_4$@MFI-700RED.

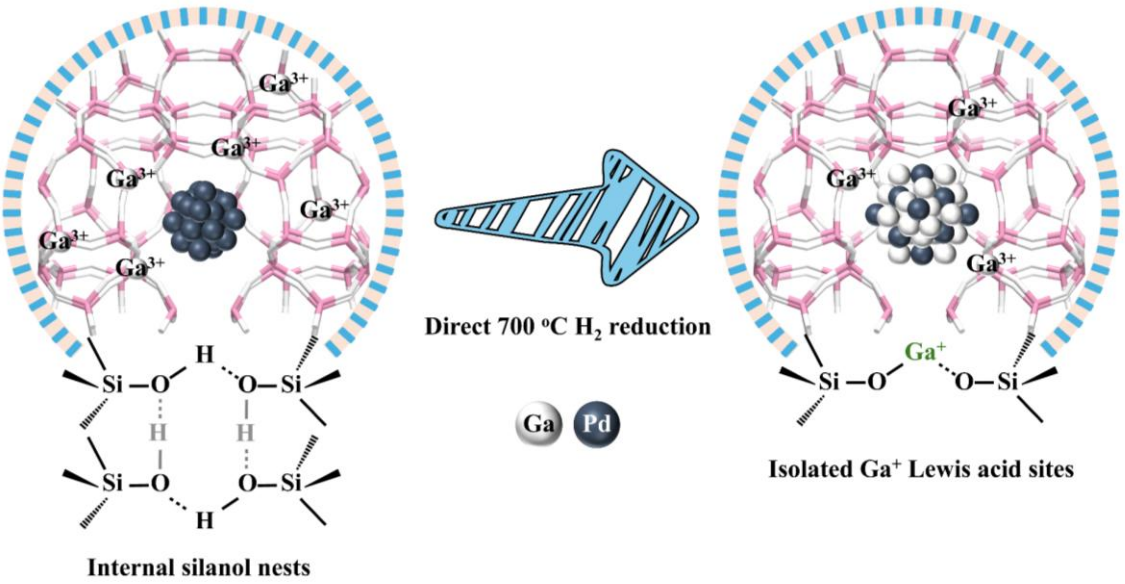

Figure S21. Illustration of H_2 induced framework Ga^{3+} migration stabilizing as isolated Ga^{+} Lewis acid site on internal silanol nests.

4.2. XRD

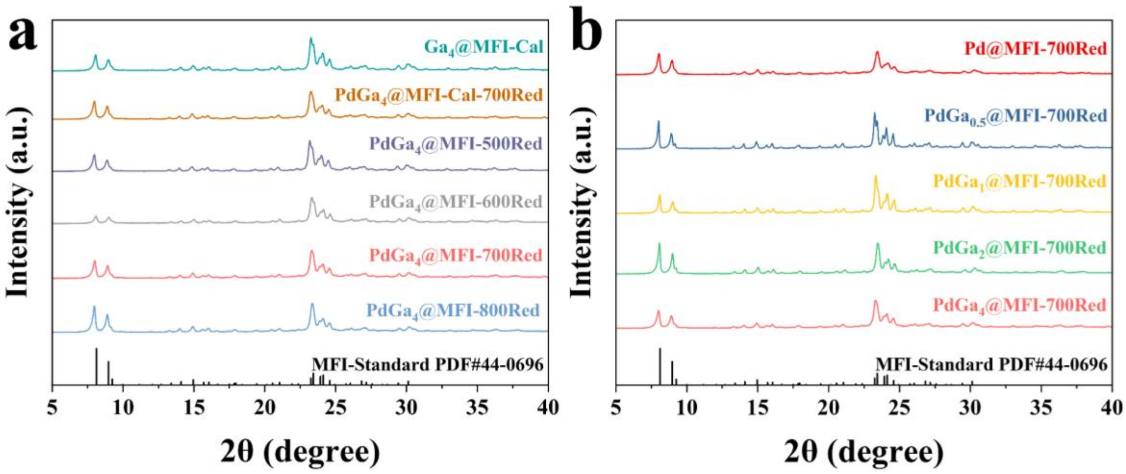

Figure S22. XRD results of (a) $PdGa_4$@MFI after different reduction temperatures and (b) $PdGa_x$@MFI-700RED with different Ga/Pd ratios.

XRD results indicate that all $PdGa_x$@MFI catalysts maintain MFI crystallinity, even after 800 ºC H_2 reduction.

4.3. CO chemisorption

Table S3. CO chemisorption data on the different $PdGa_x$@MFI samples[a]

Sample	Treatment	Dispersion (%)	Mono layer (μmol/g)
Pd@MFI-AS	*In-situ* 700 °C reduction	24.57	13.85
$PdGa_4$@MFI-AS	*In-situ* 500 °C reduction	26.19	14.76
$PdGa_4$@MFI-AS	*In-situ* 600 °C reduction	23.80	13.42
$PdGa_4$@MFI-AS[b]	*In-situ* 700 °C reduction	14.28 ± 1.5	7.89 ± 0.7
$PdGa_4$@MFI-700RED[b]	*In-situ* 350 °C reduction	11.1 ± 2.2	6.26 ± 1.2
$PdGa_4$@MFI-700RED	*In-situ* 700 °C reduction	12.31	6.94
$PdGa_4$@MFI-800RED	*In-situ* 350 °C reduction	2.37	1.34
$PdGa_4$@MFI-Cal700RED	*In-situ* 350 °C reduction	4.83	2.72

[a] Dispersion data are calculated assuming a stoichiometry Pd:CO=1:1.
[b] $PdGa_4$@MFI were tested 4 times to get error range.

Comments:

The dispersion and mono-layer CO adsorption capacity of Pd in PdGa@MFI catalysts are estimated using the double isotherm method on a Quantachrome Autosorb-1C instrument. Initially, $PdGa_4$@MFI are *in-situ* reduced at 500, 600 and 700 °C, respectively. $PdGa_4$@MFI-500 and 600RED exhibit similar CO adsorption capacities to Pd@MFI-700RED, indicating Ga is still not alloyed with Pd. Conversely, $PdGa_4$@MFI-700RED exhibits approximately half CO absorption capacity compared to Pd@MFI-700RED, giving strong evidence that Pd is diluted by Ga to form PdGa intermetallic structures.

$PdGa_4$@MFI-Cal-700RED exhibit lower CO adsorption capacity than the $PdGa_4$@MFI-700RED sample which, supported on HRTEM analysis, is explained due to Pd sintering on the zeolite surface after calcination and subsequent reduction (See Figure S40).

On the other hand, the reduced CO adsorption capacity observed on the $PdGa_4$@MFI-800RED sample may be related to the presence of gallium oxide on the surface of the PdGa intermetallic compound. This is in line with IR-CO studies where a low IR signal of adsorbed CO is observed probably due to blocking of surface sites by gallium oxide species.

Subsequently, studies on *ex-situ* reduced samples shows that a second reduction

temperature of 350 °C is sufficient to restore PdGa alloy surface structure in the *ex situ* reduced $PdGa_4$@MFI-700RED sample. This finding is collaborated by H_2-TPR analysis of $PdGa_4$@MFI-700RED (Figure S23).

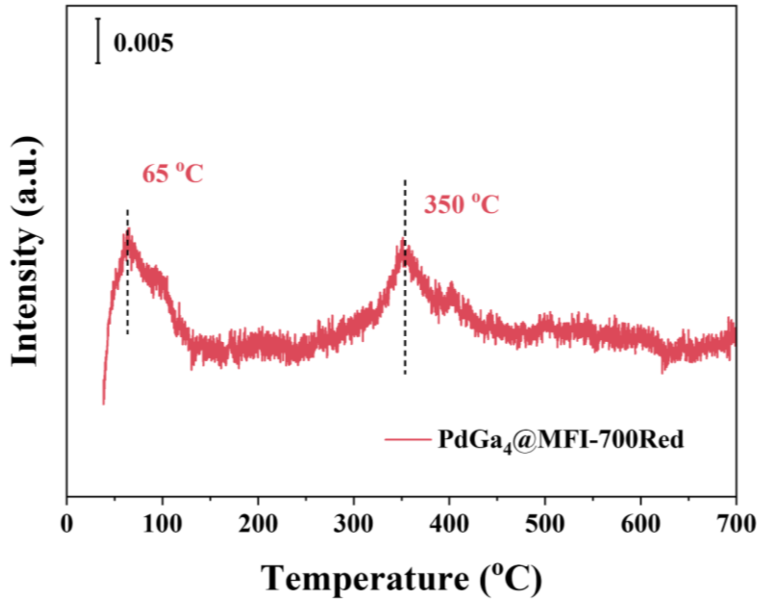

Figure S23. H_2-TPR of $PdGa_4$@MFI *ex-situ* 700 °C H_2 reduced sample and exposed to air for two weeks.

<u>Comments:</u>

As observed in Figure S23, two different reduction peaks are observed at 65 and 350°C in the H_2-TPR pattern of the *ex-situ* $PdGa_4$@MFI-700RED catalyst after air exposure, corresponding to the reduction of partially oxidized surface species. Nevertheless, the amount of H_2 is very low, indicating for a low fraction of oxidized species after air exposure. According to the H_2-TPR pattern and supported by CO chemisorption studies, it is possible to fully recover the pristine state of the PdGa alloy by submitting the sample to on an *in-situ* reduction treatment at 350 °C. This pre-treatment is used in the catalytic and spectroscopic studies.

4.4. IR-CO results of different post-treatment temperatures on $PdGa_4$@MFI.

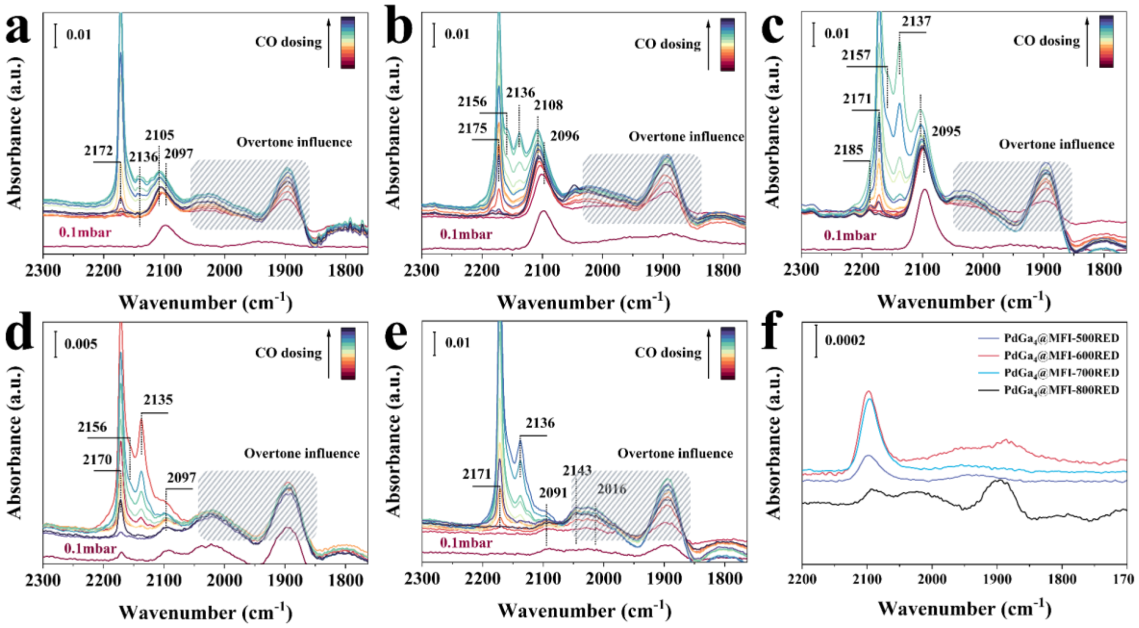

Figure S24. IR-CO at -100 ºC and at increasing CO dosing from 0.1 mbar to 3 mbar of $PdGa_4$@MFI under different pretreatment conditions: (a) 500RED, (b) 600RED, (c) 700RED, (d) 800RED, (e) Cal-700RED, and (f) summary of different pretreatments at low CO coverage (0.1 mbar CO). Spectra normalized to sample overtones. In all samples, IR bands in the 1970-1890 cm^{-1} IR region are observed at increasing CO coverages above 0.1 mbar which are due to a bad subtraction of the reference sample in the overtone region. The region is marked in shadow and labelled as overtone influence.

Comments:

➢ **Identification Pd environment under different reduction temperatures.**

The binding mode of CO used as probe molecule in IR spectroscopy (IR-CO), allows to determine the fraction of Pd species in different local configurations, differentiating between isolated sites and Pd domains. This can be obtained from the ratio of lineal coordinated CO (CO interacting with one site) and bridge CO (CO coordinated to two sites). Thus, one peak at 2095 cm^{-1} due to linear CO is observed in the $PdGa_4$@MFI-700RED sample (Figure S24c), whereas bridging CO (1955 cm^{-1}) peak coexist in the 500 and 600 reduced samples. This may indicate the stabilization of single-isolated Pd site in the $PdGa_4$@MFI-700RED sample, while the coexistence of Pd domains on the other samples. The stabilization of single Pd sites has been reported in the literature for Ga rich PdGa alloys[14-16].

In the sample reduced at 800 ºC, a lower IR intensity of adsorbed CO is observed, indicating a lower amount of surface Pd species, in line with EDX data, probably due to the blocking of surface Pd sites by surface segregated GaO_x species.

In line with the IR-CO data, the stabilization of isolated Pd species in the PdGa@MFI-700RED sample, versus more extended Pd domains is confirmed from CO chemisorption analysis (Table S3), where the CO surface adsorption, decrease half, from 13 µmol/g to 7 µmol/g, by increasing the reduction temperature from 500 to 700 ºC respectively. Moreover, over the $PdGa_4$@MFI-800RED sample, the chemisorbed CO decrease markedly to a value of ~2 µmol/g in line with the IR-CO data.

- **Identification of Ga^+ species in PdGa@MFI-RED samples.**

According to the literature, Ga species may exist in zeolite-based materials in different configurations (see Figure S25), resulting in different CO IR peaks, which are summarized in Table S4. Thus, CO adsorbed on coordinatively unsaturated Ga^+ sites has been reported at 2144-2150 cm^{-1} (Ref [17]), while also at 2174 cm^{-1} (Ref [18]) and 2188 cm^{-1} (Ref [19]). In the last case Hensen et al, prepared a gallium-modified HZSM-5 zeolite by impregnation. According to these authors, reduction of gallium species at high temperature (500 ºC) results in the formation of low-coordinated Ga^+ ions, characterized by an IR-CO band at 2188 cm^{-1}. Other authors report a band at 2189-2195 cm^{-1} to isolated low coordinated $Ga^{\delta+}$ species that were formed on a Ga-containing ZSM-5 zeolite under reductive treatment (Ref [20]). Controversially, Otero Arean assign a band at 2190 cm^{-1} to CO adsorbed on Ga^{3+} in mesoporous Ga_2O_3 (Ref [21]), while Ga^{3+} in most studies of CO adsorbed on Ga^{3+} zeolite supported samples are characterized by IR Bands in the 2214-2223 cm^{-1} region (Ref [17,19,22,23]).

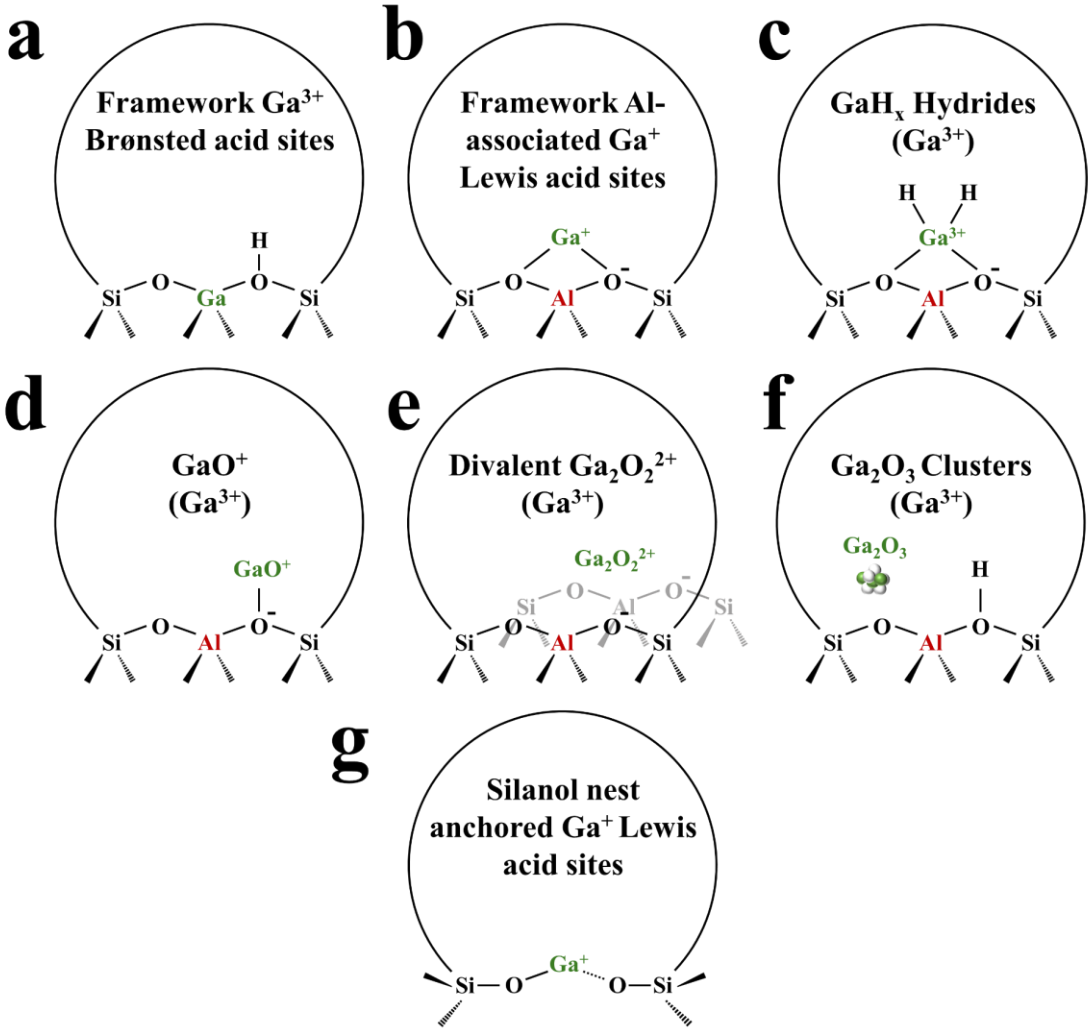

Figure S25. Literature reported Ga species on MFI zeolite: (a) Ga^{3+} isomorphous substitution of MFI zeolite; (b) Framework Al associated Ga^{+} Lewis acid sites; (c) GaH_x hydrides under H_2 atmosphere; (d) GaO^{+} species on HZSM-5; (e) Divalent $Ga_2O_2^{2+}$ on HZSM-5; (f) Ga_2O_3 clusters on HZSM-5; (g) Silanol nests stabilized isolated Ga^{+} Lewis acid sites on MFI zeolite (our case).

Table S4. Literature reported different Ga species inside zeolite.

Ga-zeolite structure	XANES edge energy (eV)		Peak location of IR-CO (cm^{-1})			Ref
	Ga^+	Ga^{3+}	Lewis Ga^+	Lewis Ga^{3+}	Brønsted ≡ Si-Ga^{3+}-OH	
Ga-MFI					2173 (Figure S25 a)	24
Ga/HZSM-5	10371.5		2147 (Figure S25 b)	2235 (Ga_2O_3)		17
Ga-MOR Ga-MFI			2150 (Ga-MOR) 2147 (Ga-MFI)	2218		22
Ga/HZSM-5			2155 (Figure S25 b)	2235 (Ga_2O_3)		23
Ga-HZSM-5			2188 (Figure S25 b)	2218 (GaO^+) (Figure S25 d)		19
Ga/HZSM-5	10369.9					25
Ga/SiO_2			2174			18
Ga/CHA				2034 (GaH_2^+) (Figure S25 c)		26,27
$Ga(CH_3)_3$/HZSM-5		10373	2189-2195			20
Ga/HZSM-5	10368	10372				28
Ga/HBEA	10371.4	10375.2				29
Ga/HZSM-5	10371.5	10374.9				30

For a more detailed analysis of the IR band at 2185 cm^{-1}, in our study, IR-CO experiments were acquired at increasing CO coverage in order to follow the evolution of the IR bands, which help in the peak assignment. At low dosing, CO preferentially binds to the strongest and most relevant sites, minimizing CO-CO interactions and avoiding artificial peak shifts or asymmetries due to other peaks contribution. Thus, in Figure S26, it is clearly seen that the band at 2185 cm^{-1} appears as an independent IR band, separated from the IR band at 2172 cm^{-1}. In deed it is observed that at low CO dosing (light purple line), two bands appear at 2213 and 2185 cm^{-1} associated to Lewis acid sites, since no shift in the OH region is observed. At increasing CO dosing, a new component at 2172 cm^{-1} appears in parallel to a shift in the OH band. This allows assigning the IR band at 2172 cm^{-1} to Brønsted acid sites (Ref [24]). The band at 2213 cm^{-1} can be associated to Ga^{3+} species in Ga_2O_3 (Ref [21]) but can also be assigned with cationic GaO^+ oxo-ions (Ref [19]). Notice that

extraframework Ga species are not observed by ^{71}Ga-NMR, however, its presence cannot be completely ruled out being below the detection limit of the technique (<1wt% Ga).

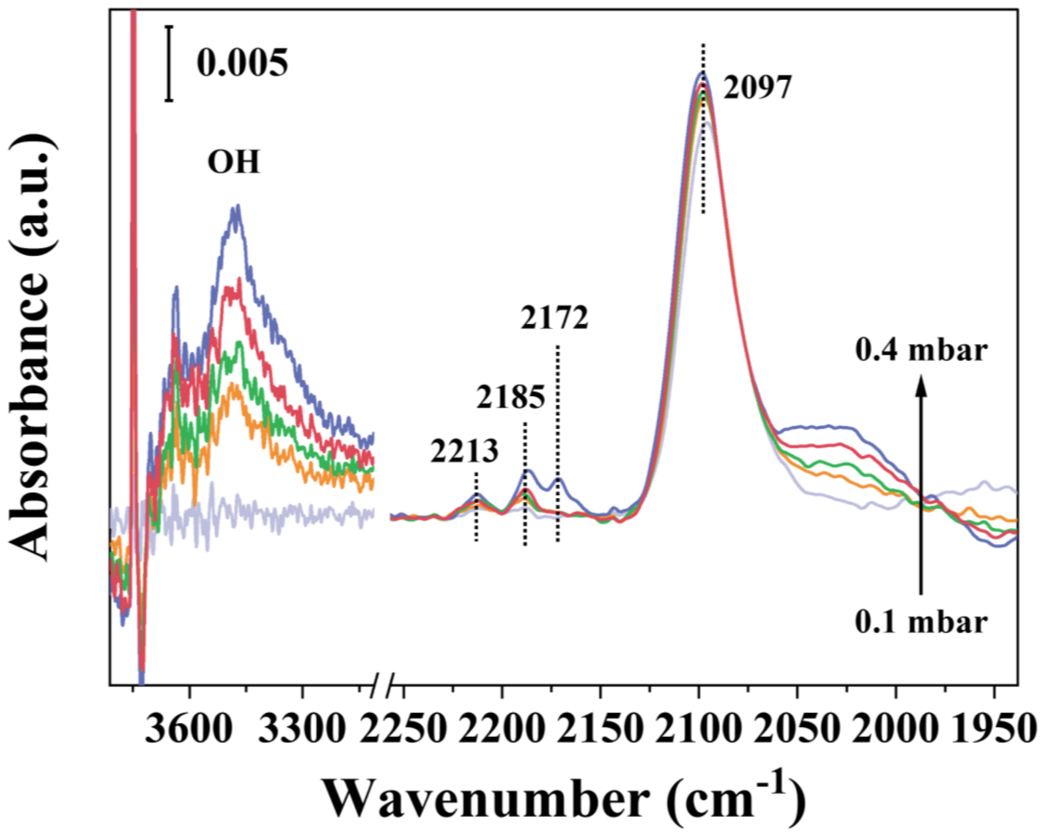

Figure S26. IR-CO spectra on the $PdGa_4$@MFI-700 RED sample at -100 ºC and low CO dosing (0.1 to 0.4mbar)

The IR band at 2185 cm^{-1} is also observed in the $PdGa_4$@MFI-600 RED sample (Figure S27), whereas in this case the IR band at 2213 cm^{-1} is absent due to a less severe reduction treatment.

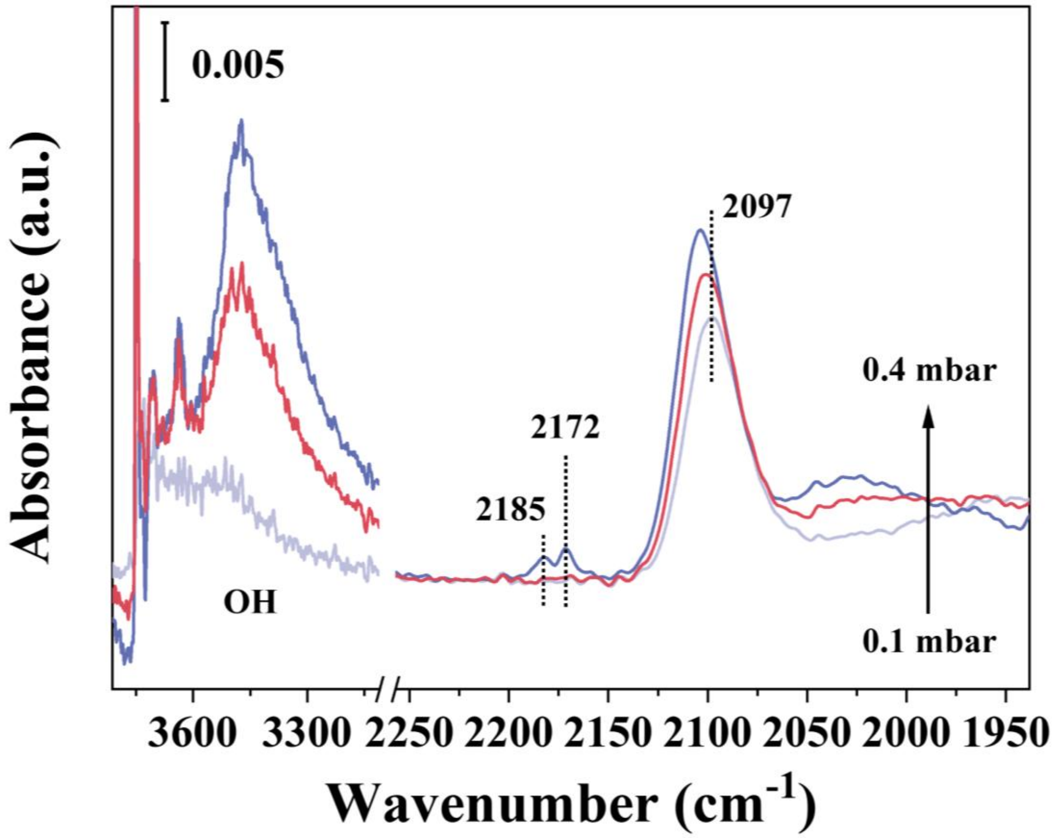

Figure S27. IR-CO spectra on the $PdGa_4$@MFI-600 RED sample at -100ºC and low CO dosing (0.1 to 0.4mbar)

Thus, the presence of isolated Ga^+ Lewis acid sites on $PdGa_4$@MFI-700RED is clearly observed by low temperature CO dosing experiments.

4.5. HAADF-STEM and iDPC results of $PdGa_4$@MFI after different post-treatment temperatures (500, 600, 700 and 800 °C).

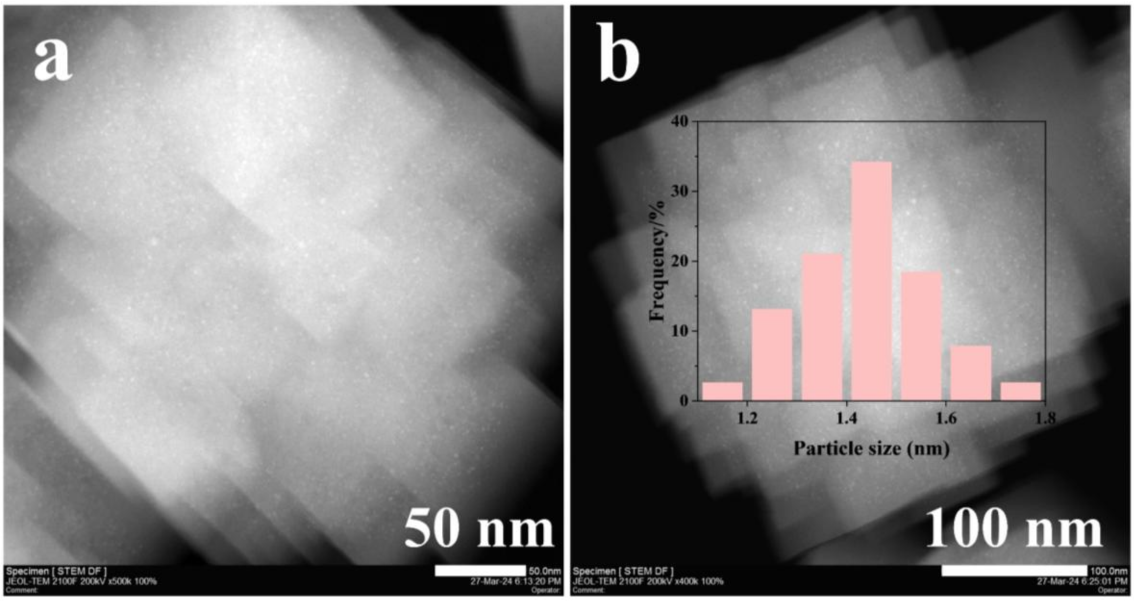

Figure S28. $PdGa_4$@MFI-500RED. Particle size distribution on the right side. The average particle sizes of each sample were calculated by the following equation within a statistical analysis of 50 particles: $d_{TEM} = \Sigma n_i d_i^3 / \Sigma n_i d_i^2$.

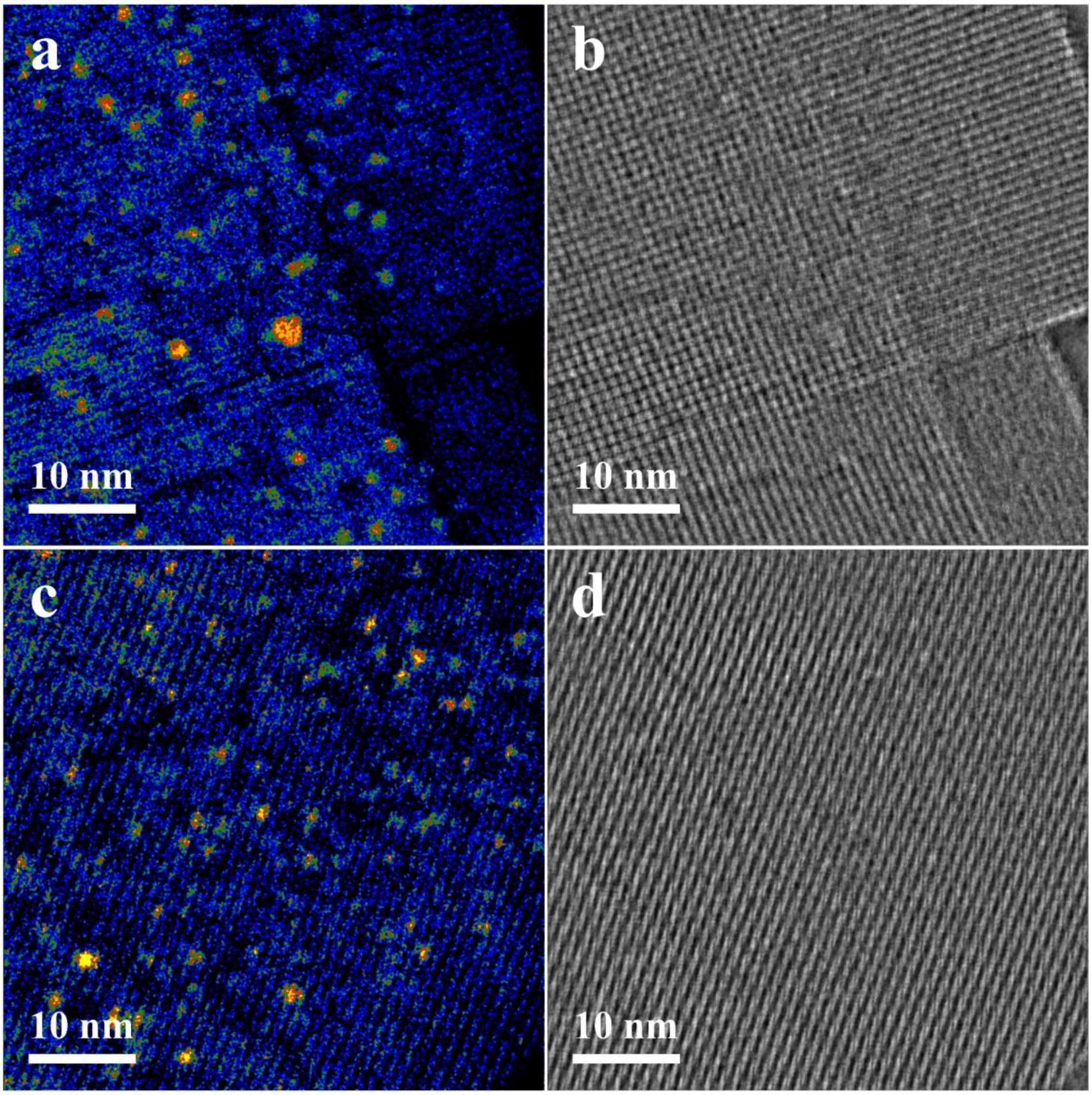

Figure S29. HR-HAADF-STEM and i-DPC of $PdGa_4$@MFI-500RED.

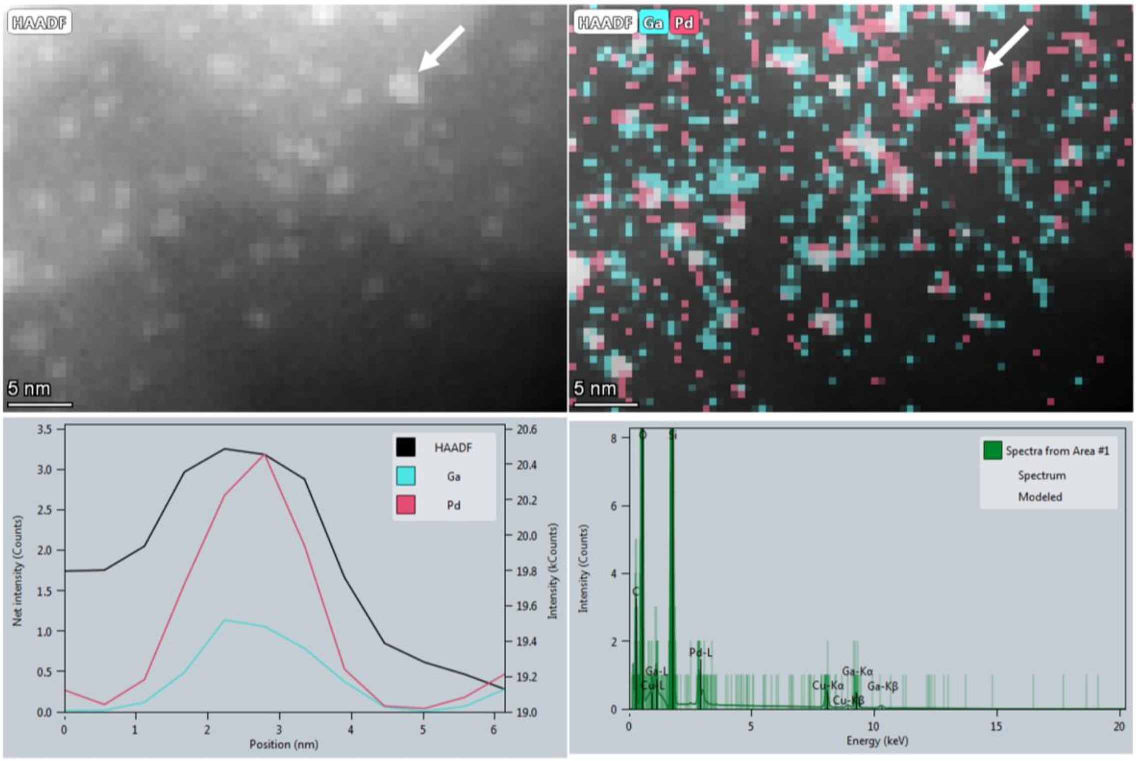

Z	Element	Family	Atomic Fraction (%)	Atomic Error (%)	Mass Fraction (%)	Mass Error (%)	Fit error (%)
31	Ga	K	41.21	16.52	31.48	10.55	29.31
46	Pd	L	58.79	18.64	68.52	15.67	12.83

Figure S30. Point analysis of $PdGa_4$@MFI-500RED with zoomed area in one selected area. Selecting most representative one point among all analyzed points. (On the one hand, we can observe clear separation of Pd and Ga on $PdGa_4$@MFI-500RED, on the other hand, the particle is Pd-rich structure, which is related to not well alloyed structure).

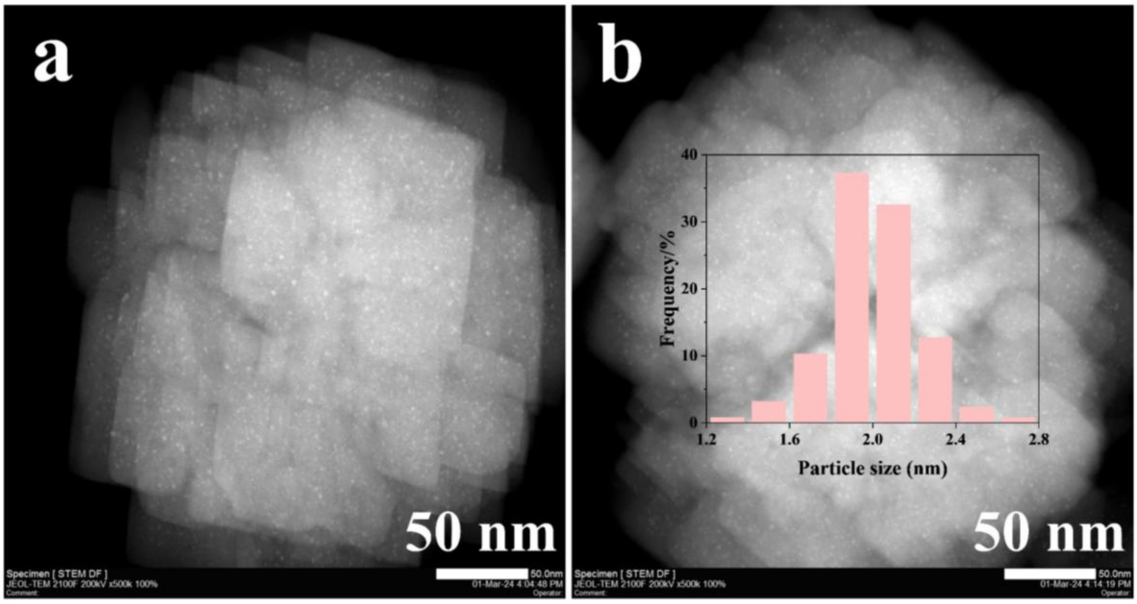

Figure S31. $PdGa_4$@MFI-600RED. Particle size distribution on the right side. The

average particle sizes of each sample were calculated by the following equation within a statistical analysis of 50 particles: $d_{TEM} = \Sigma n_i d_i^3 / \Sigma n_i d_i^2$.

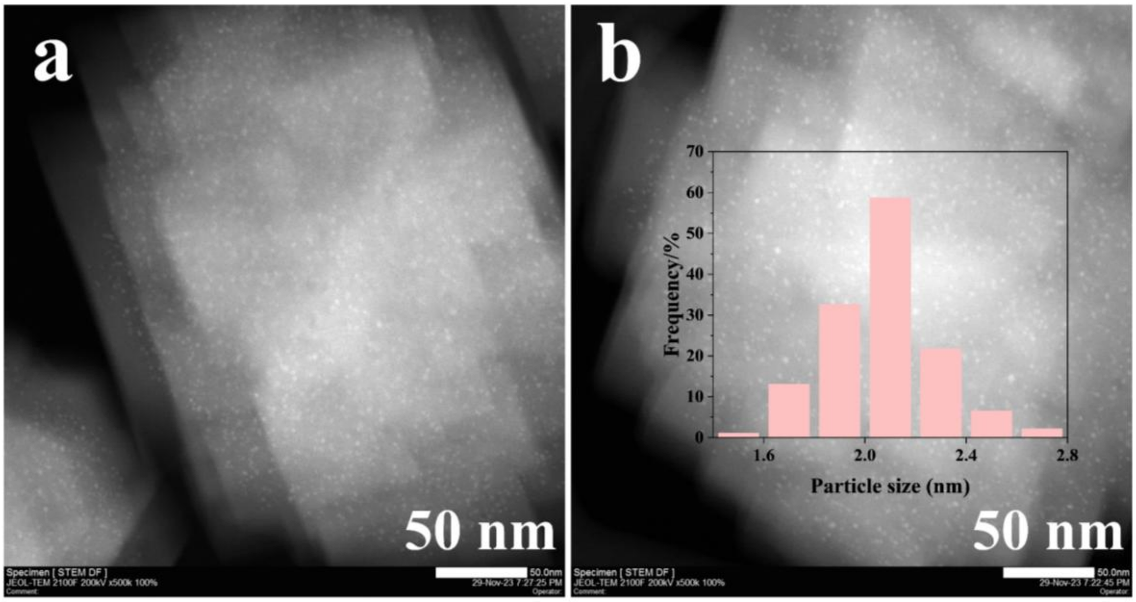

Figure S32. $PdGa_4$@MFI-700RED. Particle size distribution on the right side. The average particle sizes of each sample were calculated by the following equation within a statistical analysis of 50 particles: $d_{TEM} = \Sigma n_i d_i^3 / \Sigma n_i d_i^2$.

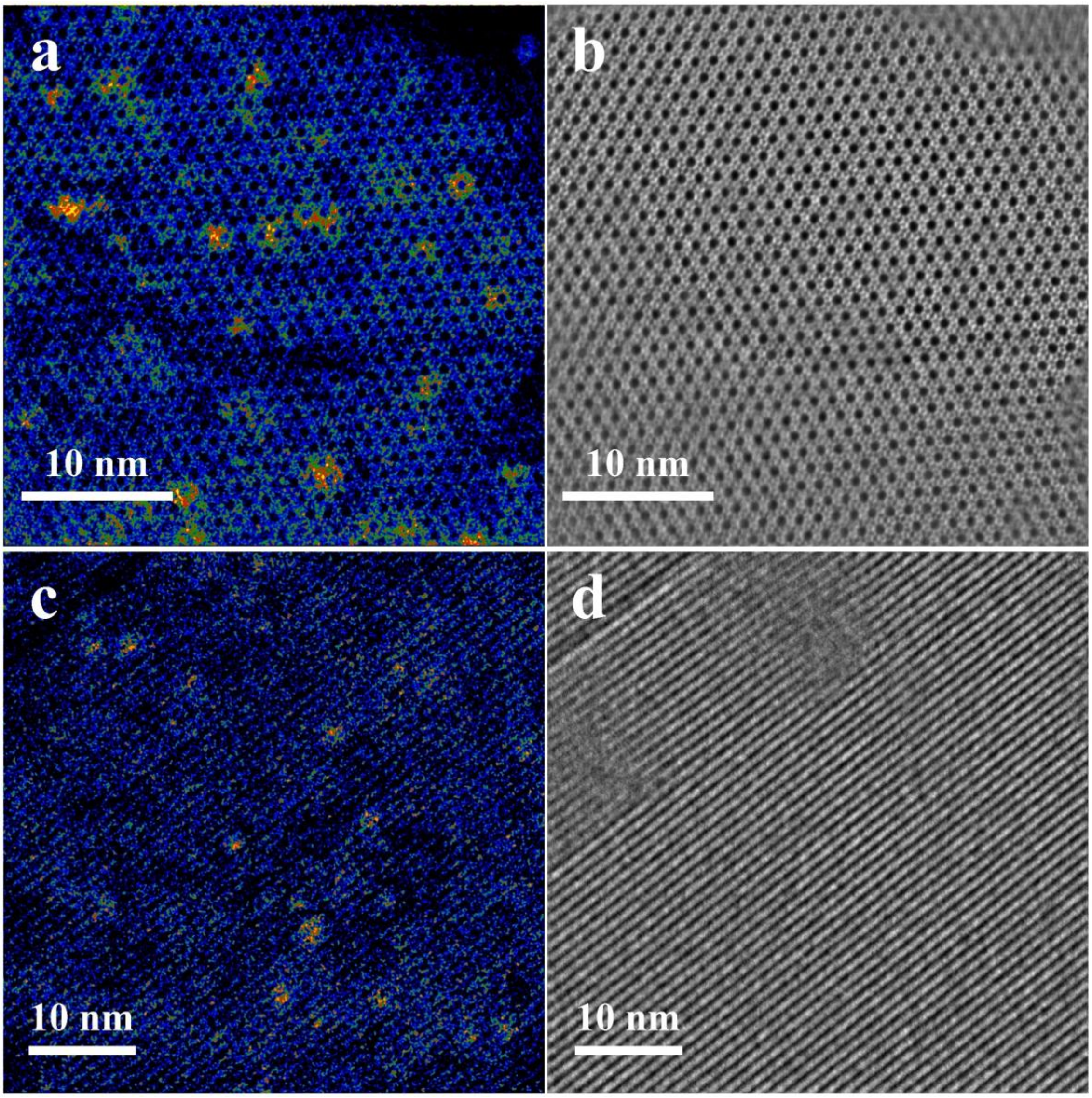

Figure S33. HR-HAADF-STEM and i-DPC of $PdGa_4$@MFI-700RED.

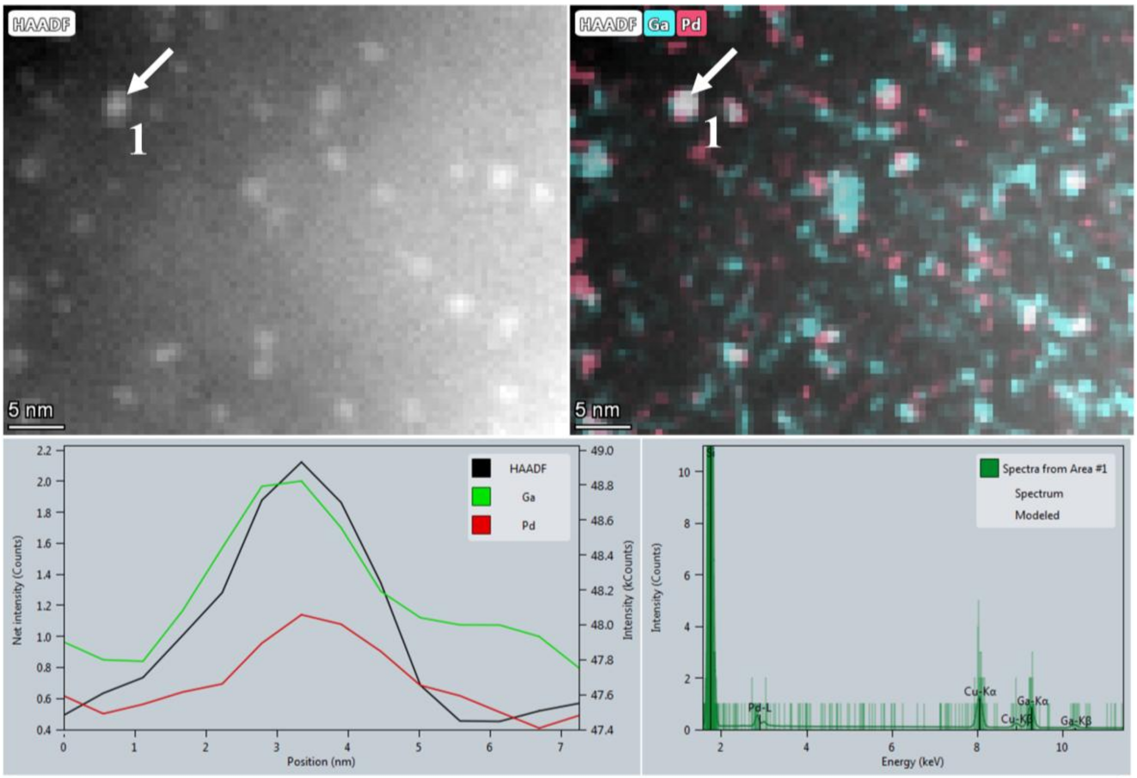

Z	Element	Family	Atomic Fraction (%)	Atomic Error (%)	Mass Fraction (%)	Mass Error (%)	Fit error (%)
31	Ga	K	65.15	15.15	55.05	9.58	12.04
46	Pd	L	34.85	9.1	44.95	9.48	13.92

Figure S34. Point analysis of $PdGa_4$@MFI-700RED with zoomed area in one selected bright point (Point 1). Selecting most representative one point among all analyzed points. (On the one hand, it indicates Pd and Ga are uniform distributed on $PdGa_4$@MFI-700RED, on the other hand, the particle is Ga-rich structure and Ga is clear observed surrounded PdGa alloy).

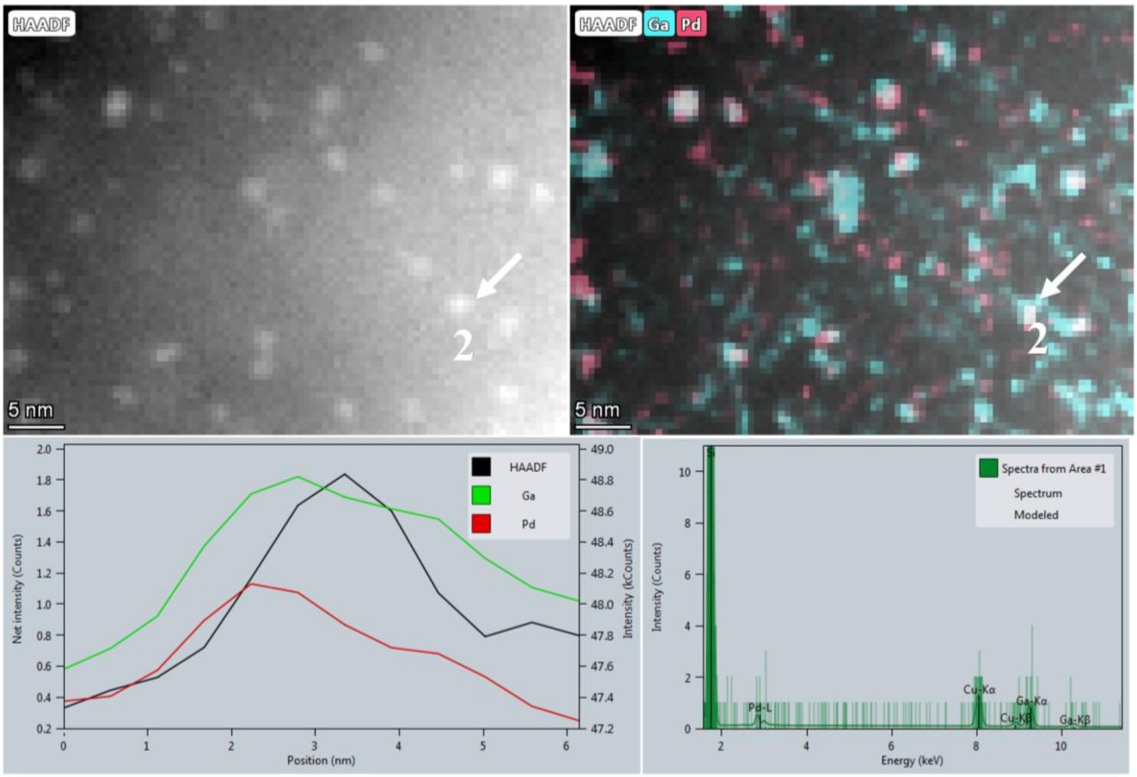

Z	Element	Family	Atomic Fraction (%)	Atomic Error (%)	Mass Fraction (%)	Mass Error (%)	Fit error (%)
31	Ga	K	75.67	25.71	67.08	16.6	17.16
46	Pd	L	24.33	10.96	32.92	12.7	33.3

Figure S35. Point analysis of $PdGa_4$@MFI-700RED with zoomed area in other selected bright point (Point 2). Selecting most representative one point among all analyzed points. (On the one hand, it indicates Pd and Ga are uniform distributed on $PdGa_4$@MFI-700RED, on the other hand, the particle is Ga-rich structure and Ga is clear observed surrounded PdGa alloy).

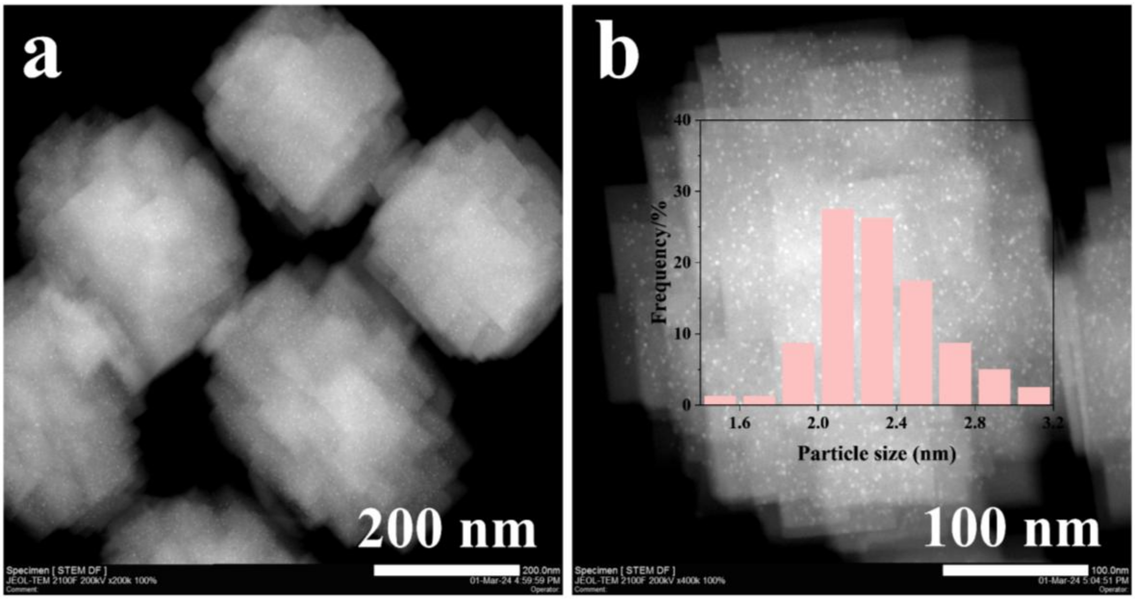

Figure S36. $PdGa_4$@MFI-800RED. Particle size distribution on the right side. The average particle sizes of each sample were calculated by the following equation within a statistical analysis of 50 particles: $d_{TEM} = \Sigma n_i d_i^3/\Sigma n_i d_i^2$.

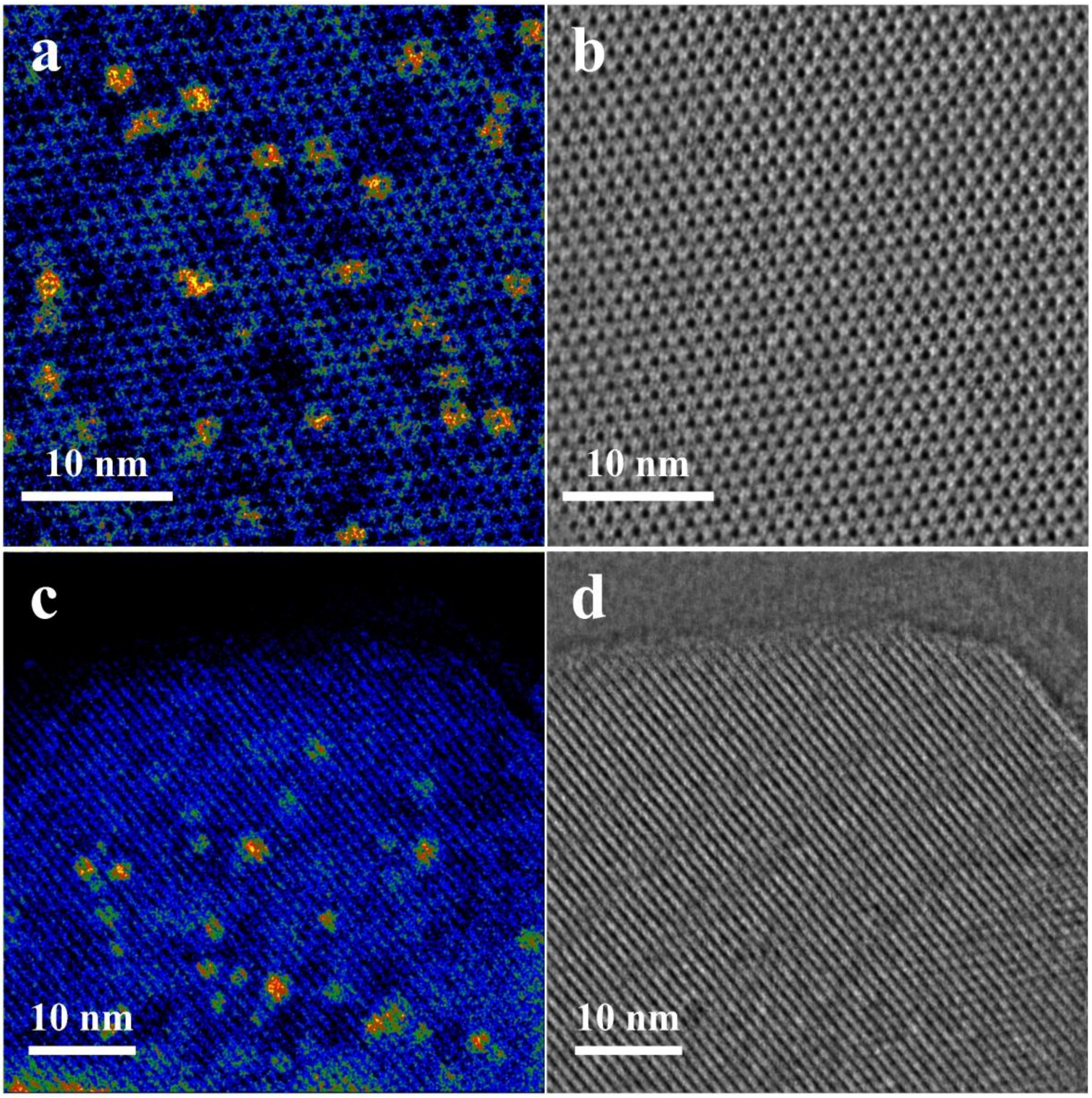

Figure S37. HR-HAADF-STEM and i-DPC of $PdGa_4$@MFI-800RED.

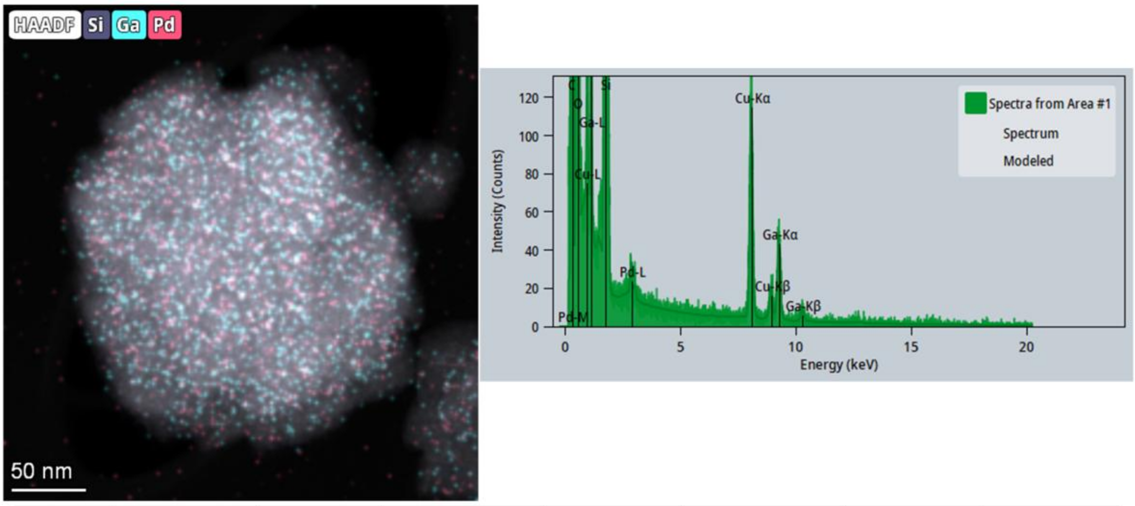

Z	Element	Family	Atomic Fraction (%)	Atomic Error (%)	Mass Fraction (%)	Mass Error (%)	Fit error (%)
31	Ga	K	82.09	9.27	75.02	6.17	0.65
46	Pd	L	17.91	2.7	24.98	3.24	1.41

Figure S38. EDX mapping analysis of $PdGa_4$@MFI-800RED with one zeolite crystal. (It indicates Pd and Ga are uniform distributed on $PdGa_4$@MFI-800RED, the Pd/Ga molar ratio is close to theoretical ratio).

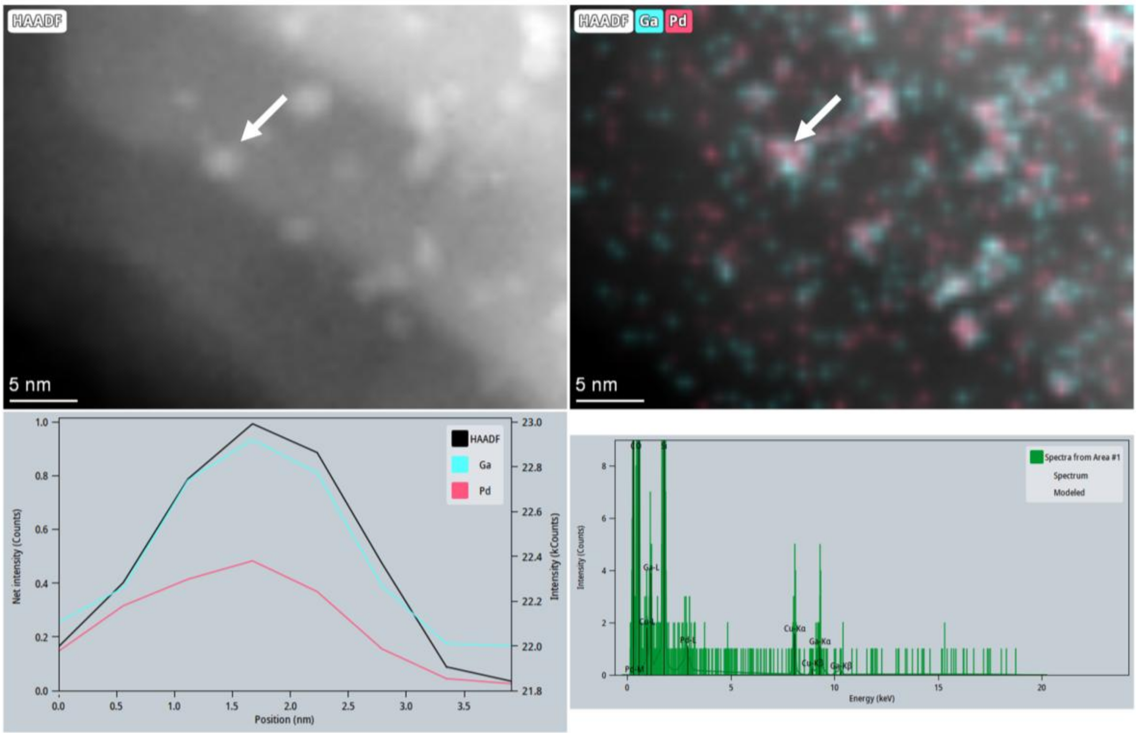

Z	Element	Family	Atomic Fraction (%)	Atomic Error (%)	Mass Fraction (%)	Mass Error (%)	Fit error (%)
31	Ga	K	69.8	17.92	60.22	11.54	12.77
46	Pd	L	30.2	8.84	39.78	9.44	17.05

Figure S39. Point analysis of $PdGa_4$@MFI-800RED with zoomed area in one selected

bright point. Selecting most representative one point among all analyzed points. (One the one hand, it indicates Pd and Ga are uniform distributed on $PdGa_4$@MFI-800RED, one the other hand, the particle is Ga-rich structure and Ga is clear observed surrounded PdGa alloy).

<u>Comments:</u>

The structures of ex situ reduced $PdGa_4$@MFI samples at 500, 600, 700, 800 ºC, were characterized by STEM, including high-resolution high-angle annular dark-field scanning transmission electron microscopy (HR-HAADF-STEM) and integrated differential phase contrast (iDPC) imaging. STEM images show a good dispersion of metal species in the $PdGa_4$@MFI reduced samples, with particle size slightly increasing from ~1.4 nm to ~2.6 nm, at increasing reduction temperatures from 500 to 800 ºC respectively (Figure S28-39). Based on paired HR-HAADF-STEM and iDPC images (also in Appendix 1), Pd containing nanoparticles are located inside MFI zeolite, even after 800 ºC reduction, without destroying the microporous structure. The preservation of the crystalline structure of the zeolite, even after high temperature reduction, is confirmed by X-ray diffraction (XRD) (Figure S20), and N_2 sorption isotherms exhibiting surface area and pore volume at around 320 m^2/g and 0.15 cm^3/g in all samples (Table S1), similar to literature reported MFI zeolites[31]. EDX mapping (Figure S30, 34, 35, 38, 39, 52, 53, 56, 57) shows homogeneous distribution of Pd and Ga in the zeolite crystallites, however due to resolution limitation of elemental mapping, it is not possible to resolve spatially the interaction between both elements. Interestingly, a higher fraction of Ga versus Pd are observed at increasing reduction temperature, related to a promoted migration of Ga species and detachment from framework positions and preferential interaction with Pd. Interestingly, if the $PdGa_4$@MFI sample is calcined at 550 ºC before reduction, Pd is easily migrated to zeolite surface due to the lack of organic template protection, leading after further reduction to ~5 nm particles on zeolite surface (Figure S40).

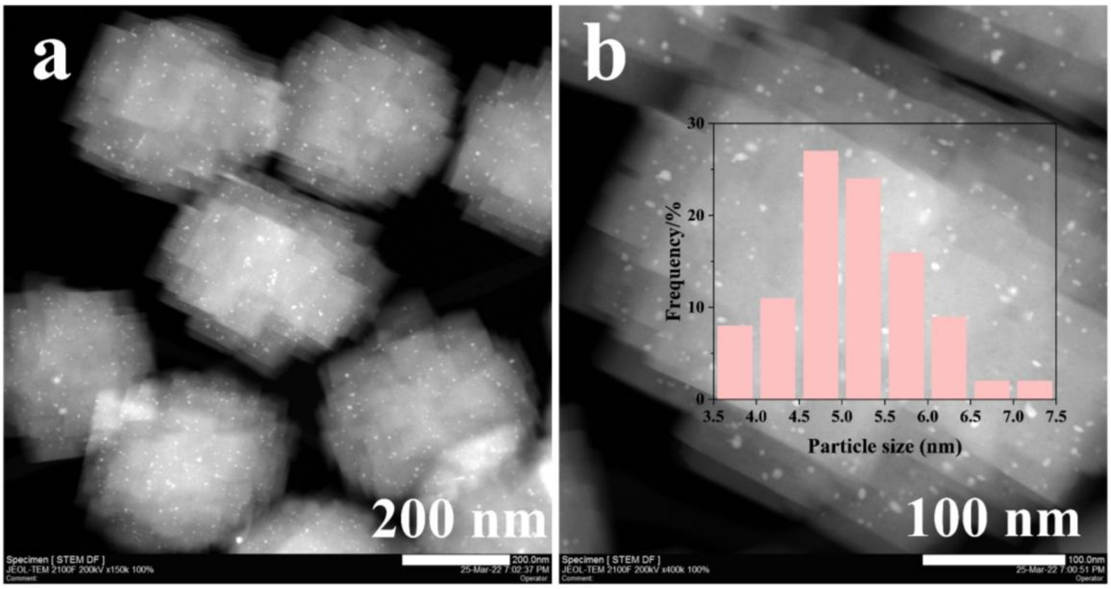

Figure S40. PdGa$_4$@MFI-Cal700RED. Particle size distribution on the right side. The average particle sizes of each sample were calculated by the following equation within a statistical analysis of 50 particles: $d_{TEM} = \Sigma n_i d_i^3 / \Sigma n_i d_i^2$.

5. Influence of the Ga/Pd ratio in PdGa$_x$@MFI samples

5.1. IR of pyridine on PdGa$_x$@MFI-700RED samples with different Ga/Pd ratios

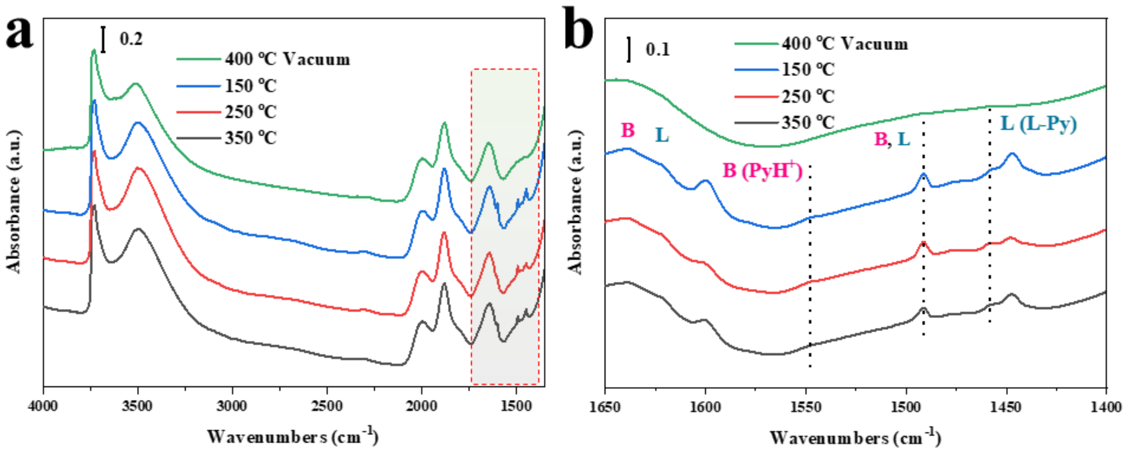

Figure S41. Pyridine-IR of PdGa$_{0.5}$@MFI-CAL (a) full scale wavenumbers from 4000 to 1300 cm^{-1}, (b) enlarged pyridine adsorption region in (a).

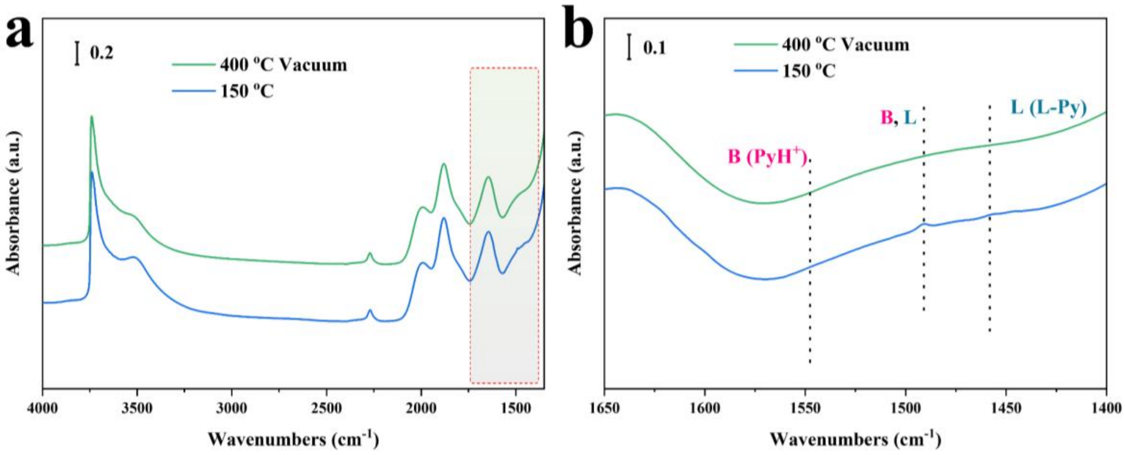

Figure S42. Pyridine-IR of $PdGa_{0.5}$@MFI-700RED (a) full scale wavenumbers from 4000 to 1300 cm^{-1}, (b) enlarged pyridine adsorption region in (a).

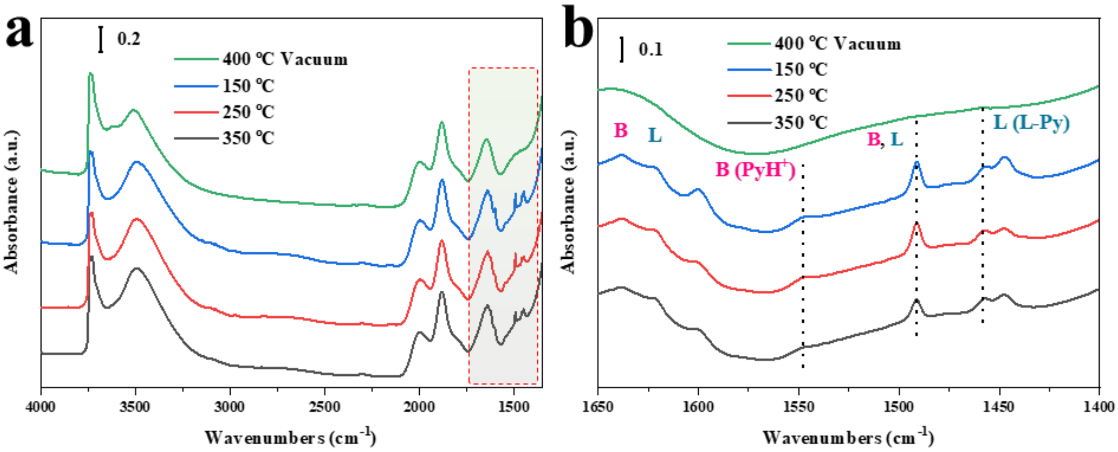

Figure S43. Pyridine-IR of $PdGa_{1}$@MFI-Cal (a) full scale wavenumbers from 4000 to 1300 cm^{-1}, (b) enlarged pyridine adsorption region in (a).

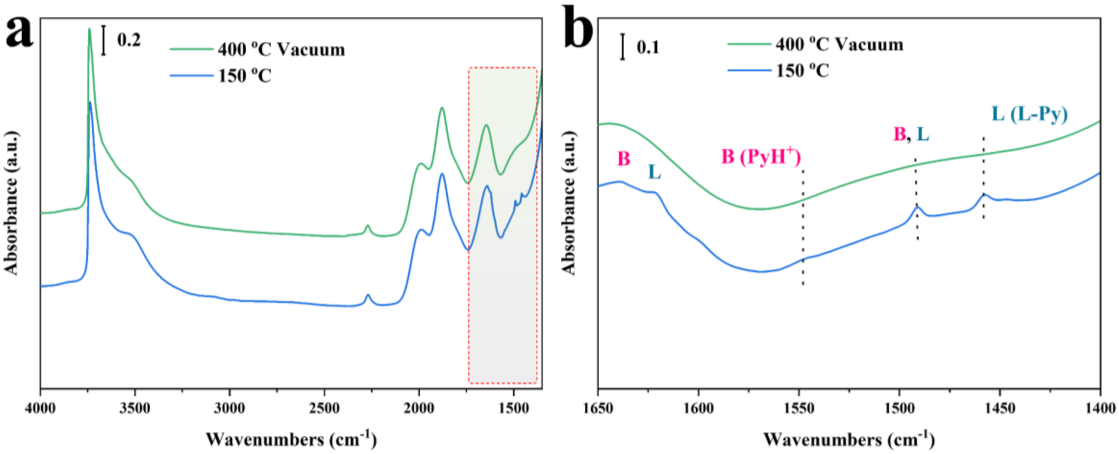

Figure S44. Pyridine-IR of $PdGa_{1}$@MFI-700RED (a) full scale wavenumbers from 4000 to 1300 cm^{-1}, (b) enlarged pyridine adsorption region in (a).

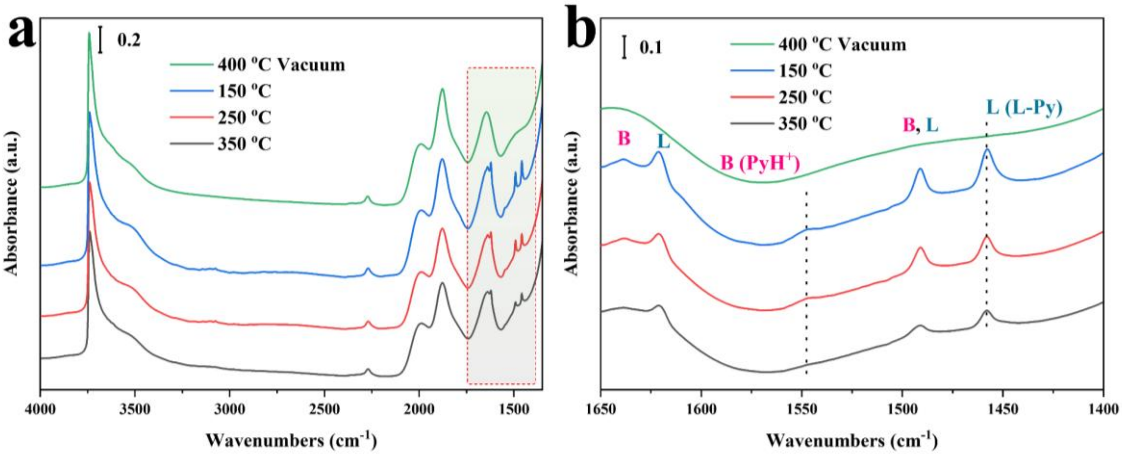

Figure S45. Pyridine-IR of $PdGa_2$@MFI-700RED (a) full scale wavenumbers from 4000 to 1300 cm^{-1}, (b) enlarged pyridine adsorption region in (a).

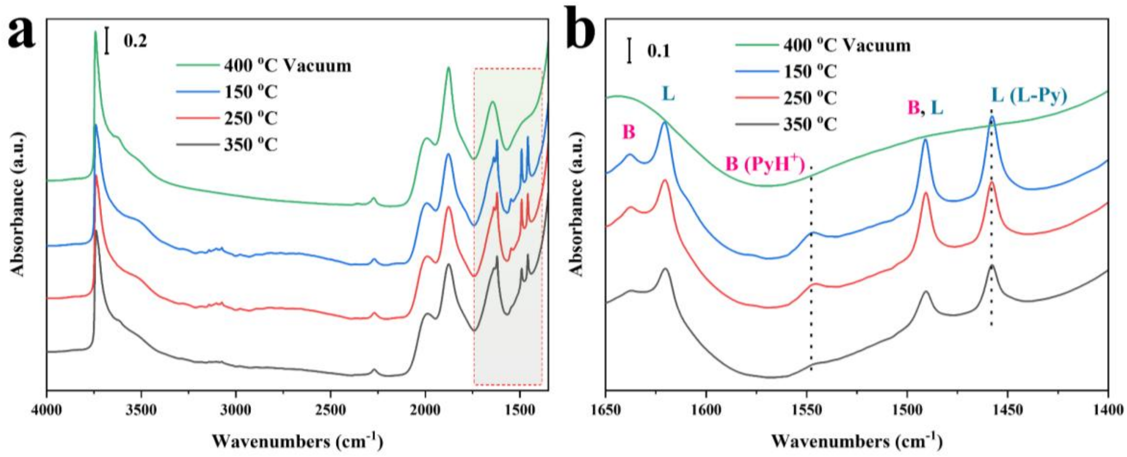

Figure S46. Pyridine-IR of $PdGa_4$@MFI-700RED (a) full scale wavenumbers from 4000 to 1300 cm^{-1}, (b) enlarged pyridine adsorption region in (a).

Table S5. Relative Brønsted and Lewis acidity on $PdGa_x$@MFI-700RED samples with different Ga/Pd ratios, determined by IR of pyridine.[a]

Sample	Actual Ga loading	Overtone area[b]	Brønsted acid area	Lewis acid area	Relative B/O[c]	Relative L/O[c]	Framework Ga^{3+} (%)[d]	Error
Ga_4@MFI-Cal	1.60 wt%	77.58	2.48	0.73	0.0320	0.0094	-	±3%
$PdGa_{0.5}$@MFI-Cal	0.19 wt%	80.96	0.088	0.037	0.001	0.00045	~25%	±7%
$PdGa_{0.5}$@MFI-700RED	0.19 wt%	106.42	0.07	0.028	0.00065	0.00026	~16%	±7%
$PdGa_1$@MFI-Cal	0.40 wt%	75.74	0.27	0.13	0.0035	0.0017	~44%	±7%
$PdGa_1$@MFI-700RED	0.40 wt%	105.52	0.14	0.40	0.00137	0.0037	~17%	±7%
$PdGa_2$@MFI-700RED	0.76 wt%	105.55	0.44	1.31	0.00414	0.0124	~26%	±7%
$PdGa_4$@MFI-700RED	1.60 wt%	81.05	0.81	1.67	0.00996	0.0206	~31%	± 7%

[a] Relative Brønsted acidity are based on 150 °C pyridine desorption.
[b] Zeolite overtone peak area.
[c] B=O area of the Brønsted acid sites (B) normalized to the area corresponding to the overtone of the sample (O), L/O = area of the Lewis acid sites (L) normalized to the area corresponding to the overtone (O) of the sample.
[d] Estimative analysis of the percent of Ga^{3+} species in framework positions calculated based on the Brønsted acidity of the corresponding sample, assuming that all Ga^{3+} is compensated by a proton (H^+). For this analysis we used Ga_4@MFI-Cal as reference sample for 100% incorporation of Ga^{3+} in the framework.

5.2. CO Chemisorption

Table S6. CO chemisorption data on the different $PdGa_x$@MFI samples [a].

Sample	Treatment	Dispersion (%)	Mono layer (µmol/g)
Pd@MFI-AS	*In-situ* 700 °C reduction	24.57	13.85
$PdGa_{0.5}$@MFI-AS	*In-situ* 700 °C reduction	20.06	11.31
$PdGa_1$@MFI-AS	*In-situ* 700 °C reduction	22.70	12.80
$PdGa_2$@MFI-AS	*In-situ* 700 °C reduction	19.66	11.08
$PdGa_4$@MFI-AS [b]	*In-situ* 700 °C reduction	14.28 ± 1.5	7.89 ± 0.7

[a] Dispersion data are calculated assuming a stoichiometry Pd:CO=1:1.
[b] $PdGa_4$@MFI were tested 4 times to get error range.

5.3. IR-CO results of PdGa$_x$@MFI-700RED with different Ga/Pd ratio

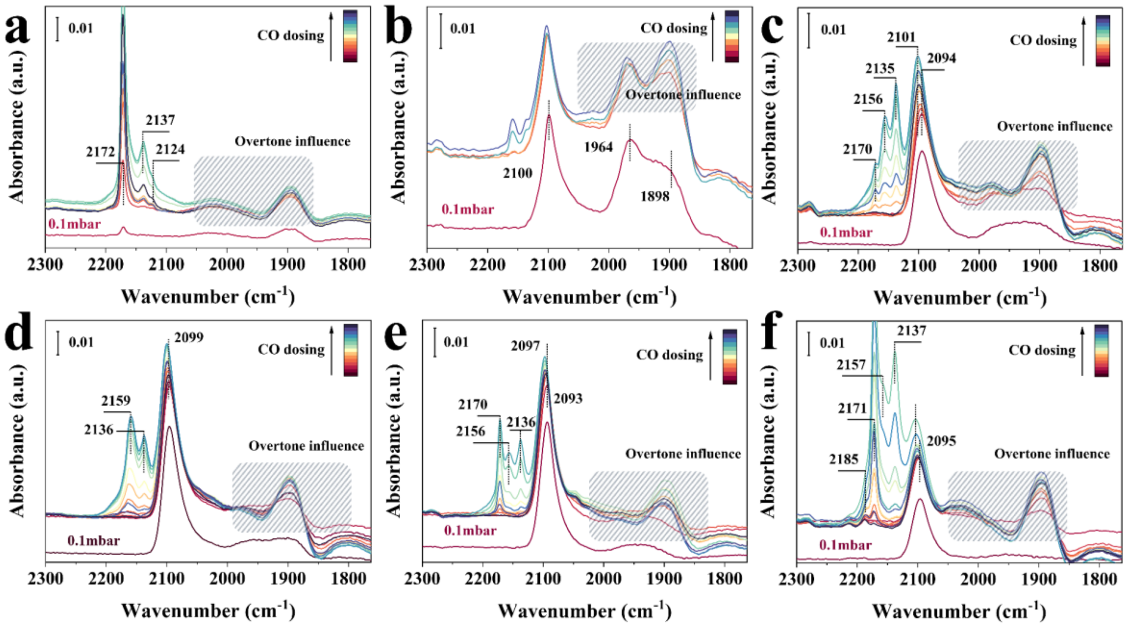

Figure S47. IR-CO at -100 ºC and at increasing CO dosing from 0.1 mbar to 3 mbar on PdGa$_x$@MFI-700RED samples with different Ga/Pd molar ratio: (a) Ga$_4$@MFI-700RED, (b) Pd@MFI-700RED, (c) PdGa$_{0.5}$@MFI-700RED, (d) PdGa$_1$@MFI-700RED, (e) PdGa$_2$@MFI-700RED, (f) PdGa$_4$@MFI-700RED. In all samples, IR bands in the 1970-1890 cm^{-1} IR region are observed at increasing CO coverages above 0.1mbar which are due to a bad subtraction of the reference sample in the overtone region. The region is marked in shadow and labelled as overtone influence. IR bands at 2170, 2156 and 2136 cm^{-1} are associated to Brönsted acid sites, silanol groups and physisorbed CO, respectively[24]. The IR band at 2185 cm^{-1} is due to Ga^{+} (Ref [19,20]) and the ones between 2097 and 1898 cm^{-1} are associated to Pd species[14].

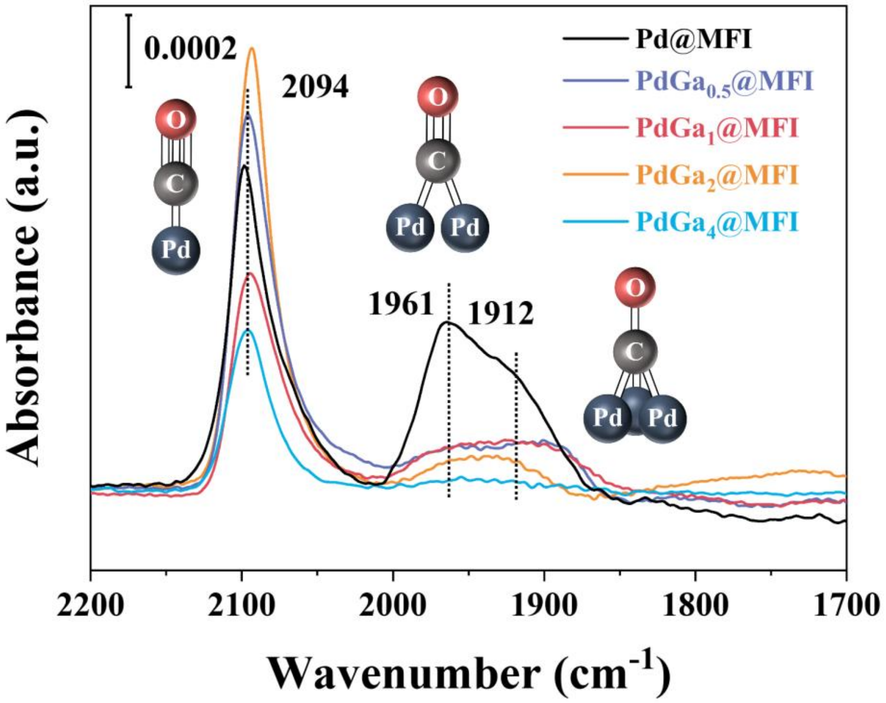

Figure S48. IR-CO at -100 ºC and low CO coverage (0.1 mbar CO) on Pd@MFI-700RED and $PdGa_x$@MFI-700RED samples. Spectra normalized to sample overtones.

Comments:

The binding mode of CO used as probe molecule in IR spectroscopy (IR-CO), allows to determine the fraction of Pd species in different local configurations, differentiating between isolated sites and Pd domains. This can be obtained from the ratio of linear coordinated CO (CO interacting with one site) and bridge CO (CO coordinated to two sites). Thus, one peak at 2094 cm^{-1} due to linear CO is observed in the $PdGa_4$@MFI-700RED sample (Figure S48), whereas bridging CO (1961 cm^{-1}) peak coexist in other different Ga/Pd ratio samples. This may indicate the stabilization of single-isolated Pd site in the $PdGa_4$@MFI-700RED sample, while the coexistence of Pd domains on the other samples. On the other hand, the lower intensity of the linear Pd-CO peak in the $PdGa_4$@MFI-700RED sample is due to the dilution of Pd by Ga.

5.4. HAADF-STEM and iDPC results of $PdGa_x$@MFI-700RED with different Ga/Pd ratio (x=0, 1 and 2)

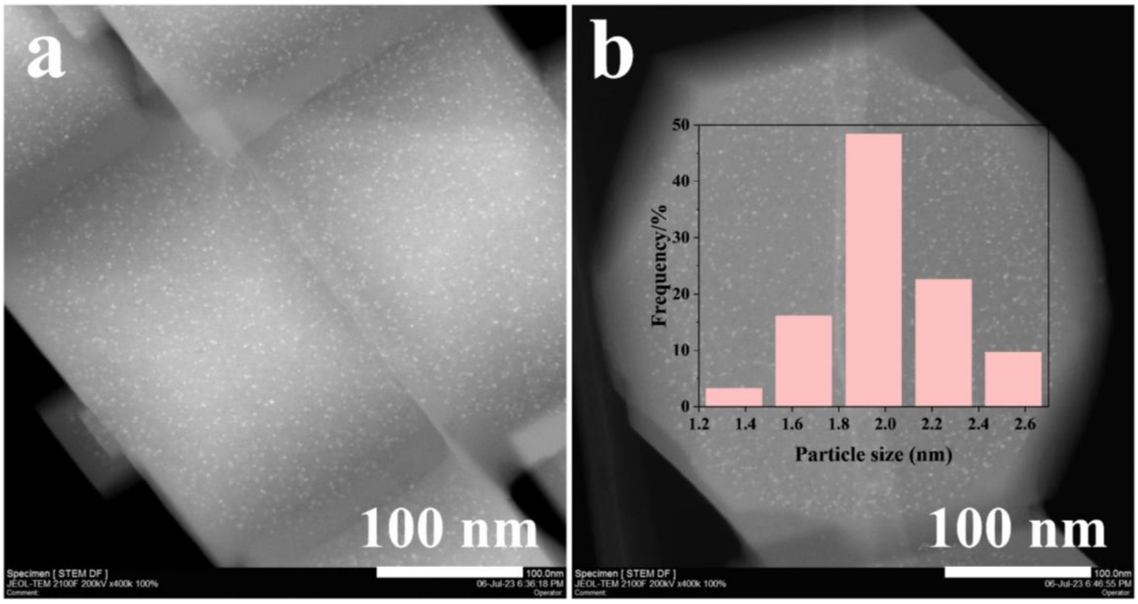

Figure S49. Pd@MFI-700RED. Particle size distribution on the right side. The average particle sizes of each sample were calculated by the following equation within a statistical analysis of 50 particles: $d_{TEM} = \Sigma n_i d_i^3/\Sigma n_i d_i^2$.

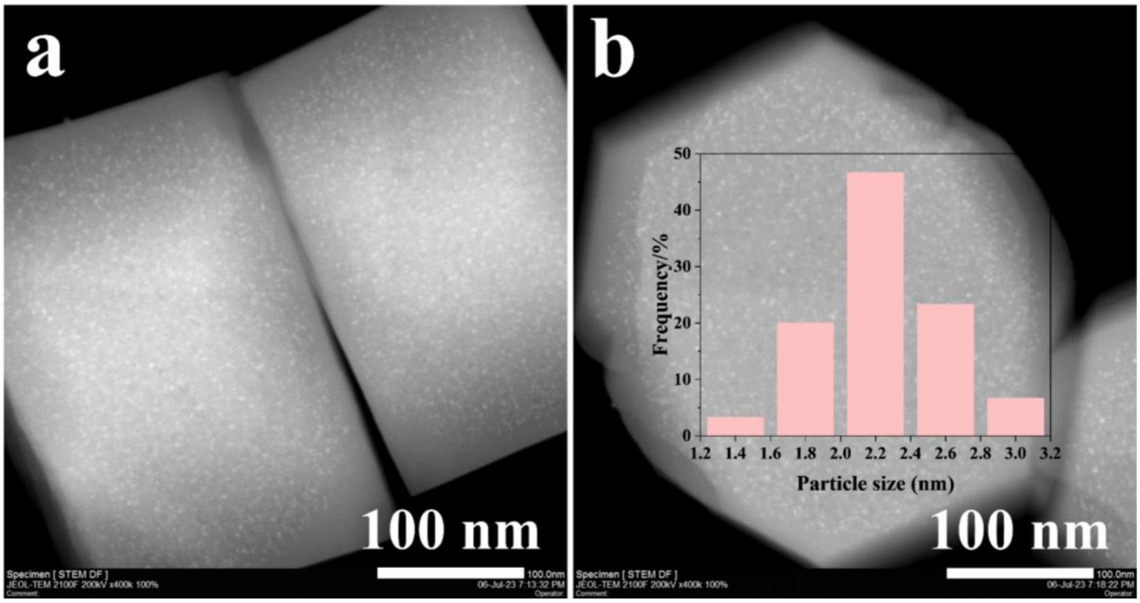

Figure S50. $PdGa_1$@MFI-700RED. Particle size distribution on the right side. The average particle sizes of each sample were calculated by the following equation within a statistical analysis of 50 particles: $d_{TEM} = \Sigma n_i d_i^3/\Sigma n_i d_i^2$.

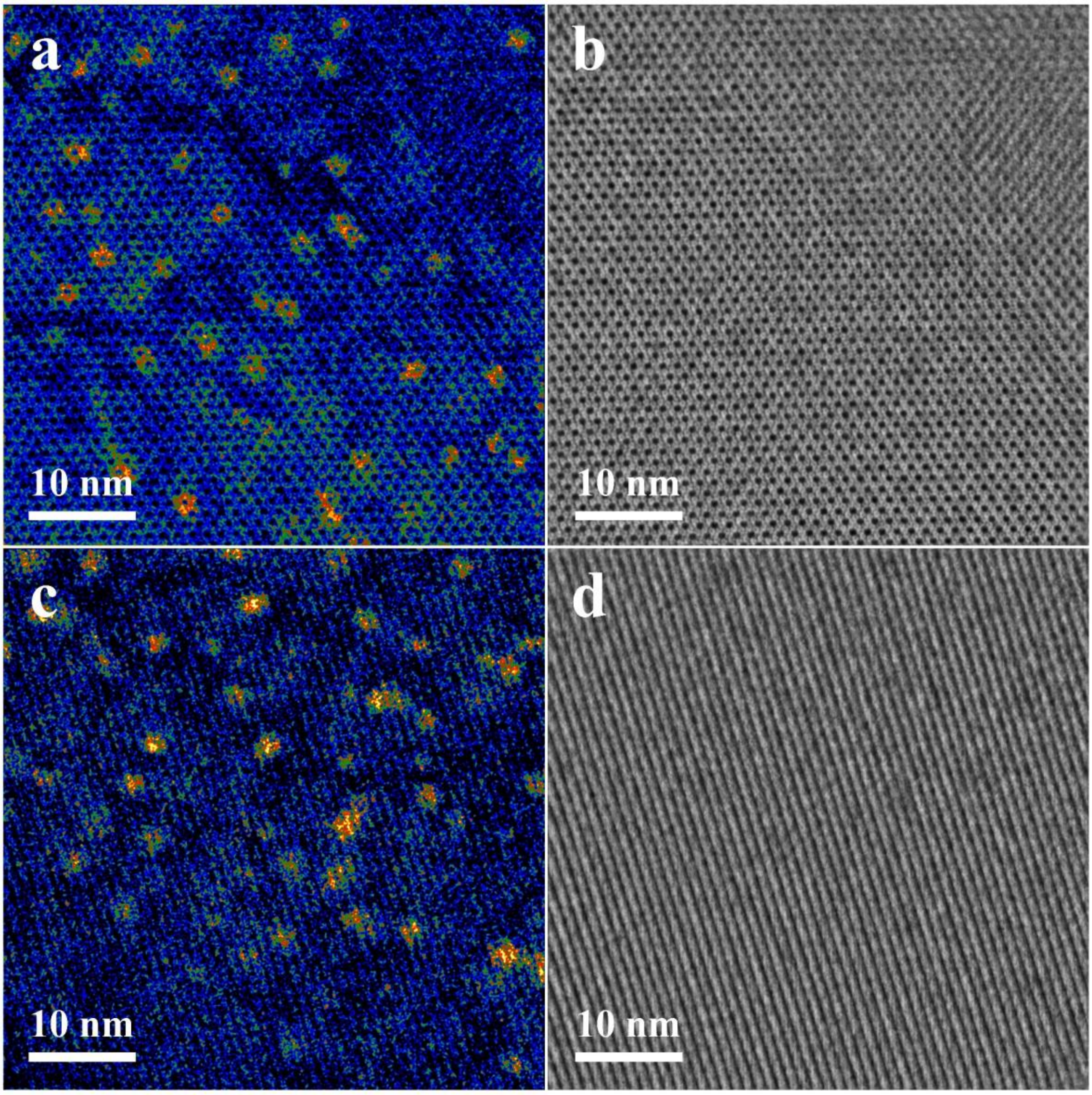

Figure S51. HR-HAADF-STEM and i-DPC of $PdGa_1$@MFI-700RED.

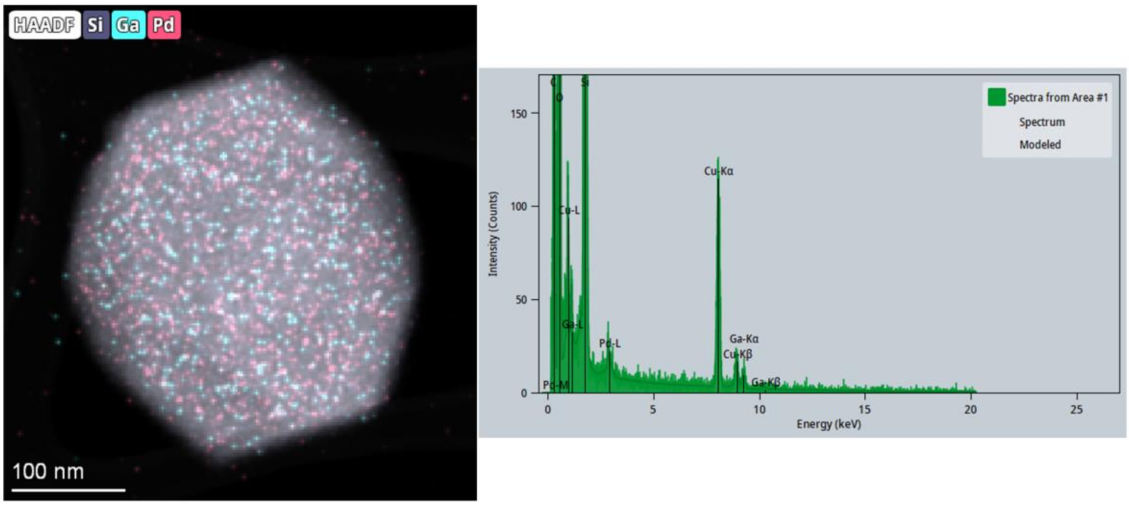

Z	Element	Family	Atomic Fraction (%)	Atomic Error (%)	Mass Fraction (%)	Mass Error (%)	Fit error (%)
31	Ga	K	49.6	7.98	39.2	4.62	4.01
46	Pd	L	50.4	9.14	60.8	8.8	2.94

Figure S52. EDX mapping of PdGa$_1$@MFI-700RED with one zeolite crystal. (It indicates Pd and Ga are uniform distributed on PdGa1@MFI-700RED, the Pd/Ga molar ratio is close to theoretical ratio)

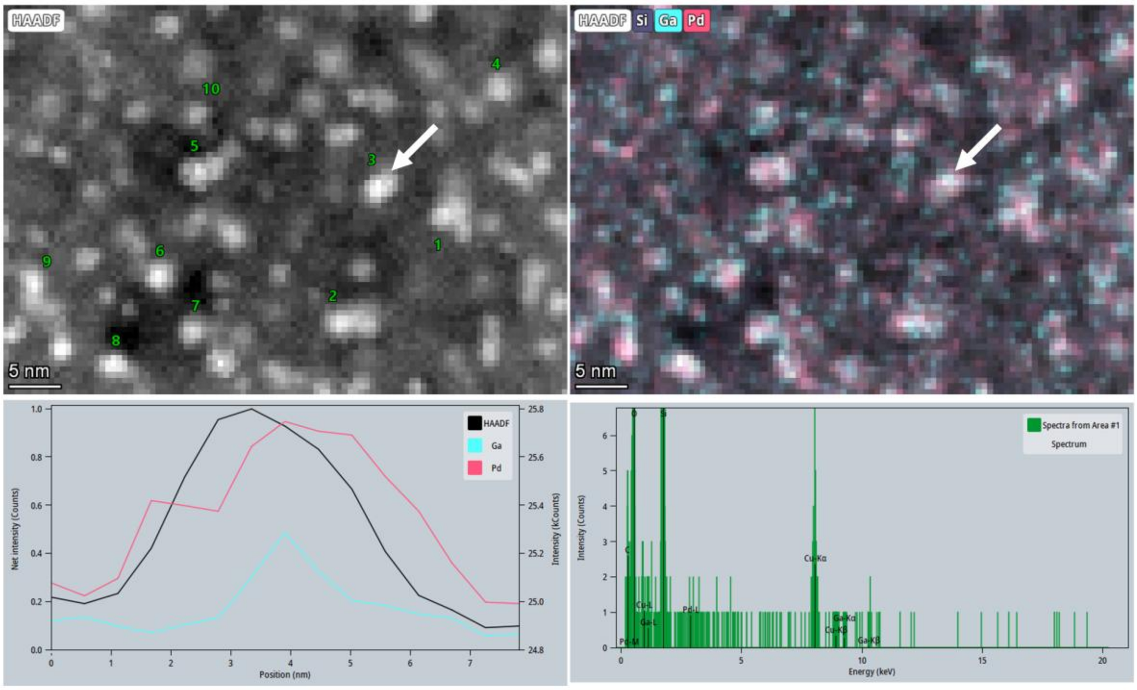

Z	Element	Family	Atomic Fraction (%)	Atomic Error (%)	Mass Fraction (%)	Mass Error (%)	Fit error (%)
31	Ga	K	39.58	24.68	30.03	16	47.97
46	Pd	L	60.42	27.5	69.97	22.39	20.55

Figure S53. Point analysis of PdGa$_1$@MFI-700RED with zoomed area in one selected area. Selecting most representative one point among all analyzed points. (On the one hand,

it indicates Pd and Ga are uniform distributed on $PdGa_1$@MFI-700RED, on the other hand, the particle is Pd-rich structure).

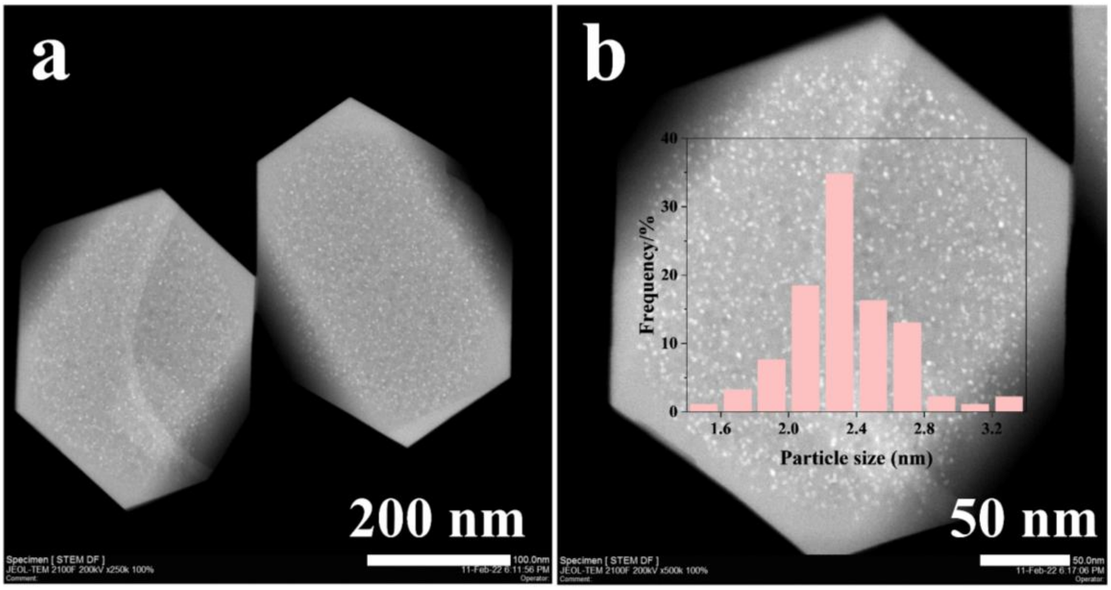

Figure S54. $PdGa_2$@MFI-700RED. Particle size distribution on the right side. The average particle sizes of each sample were calculated by the following equation within a statistical analysis of 50 particles: $d_{TEM} = \Sigma n_i d_i^3 / \Sigma n_i d_i^2$.

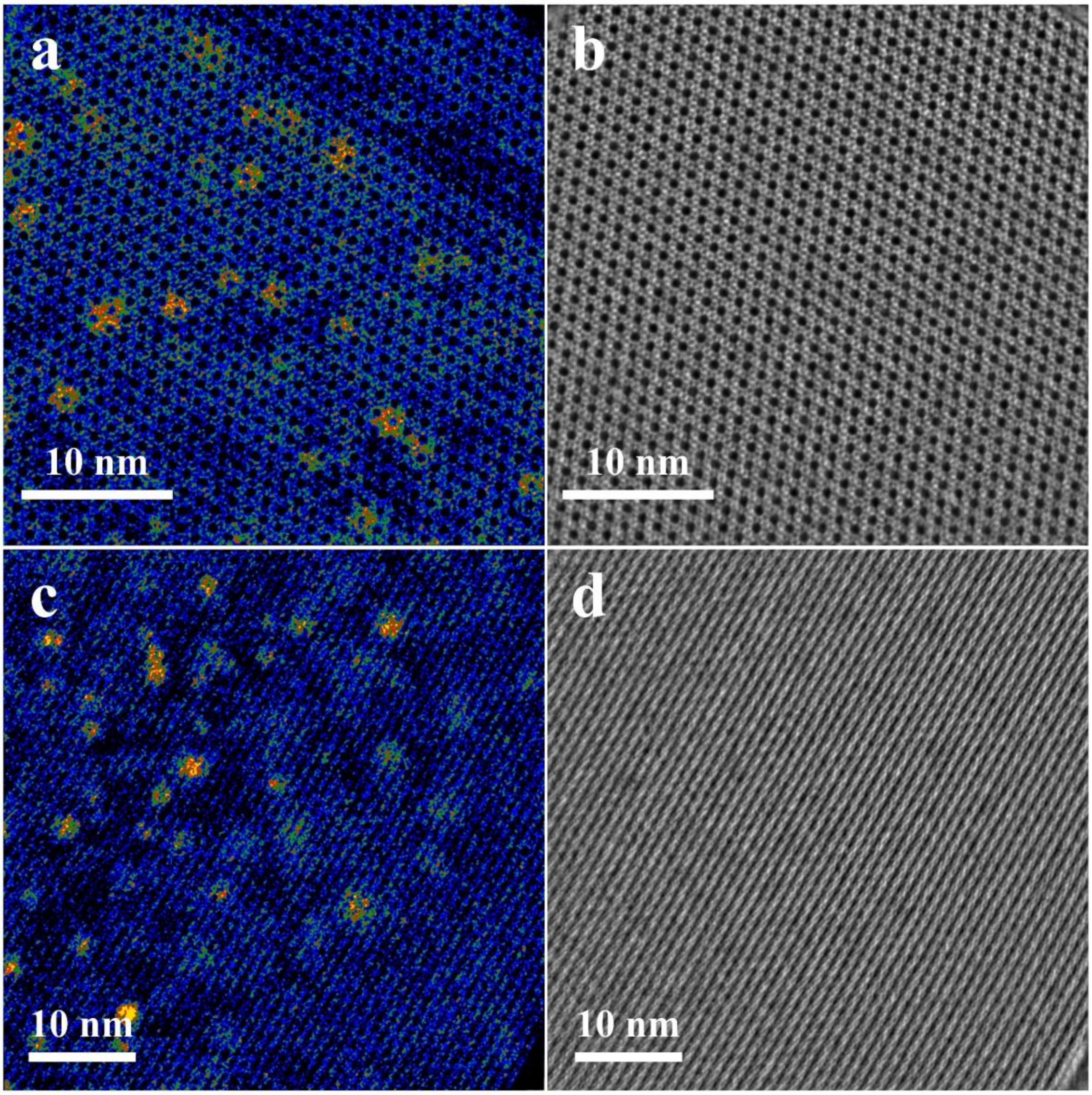

Figure S55. HR-HAADF-STEM and i-DPC of $PdGa_2$@MFI-700RED.

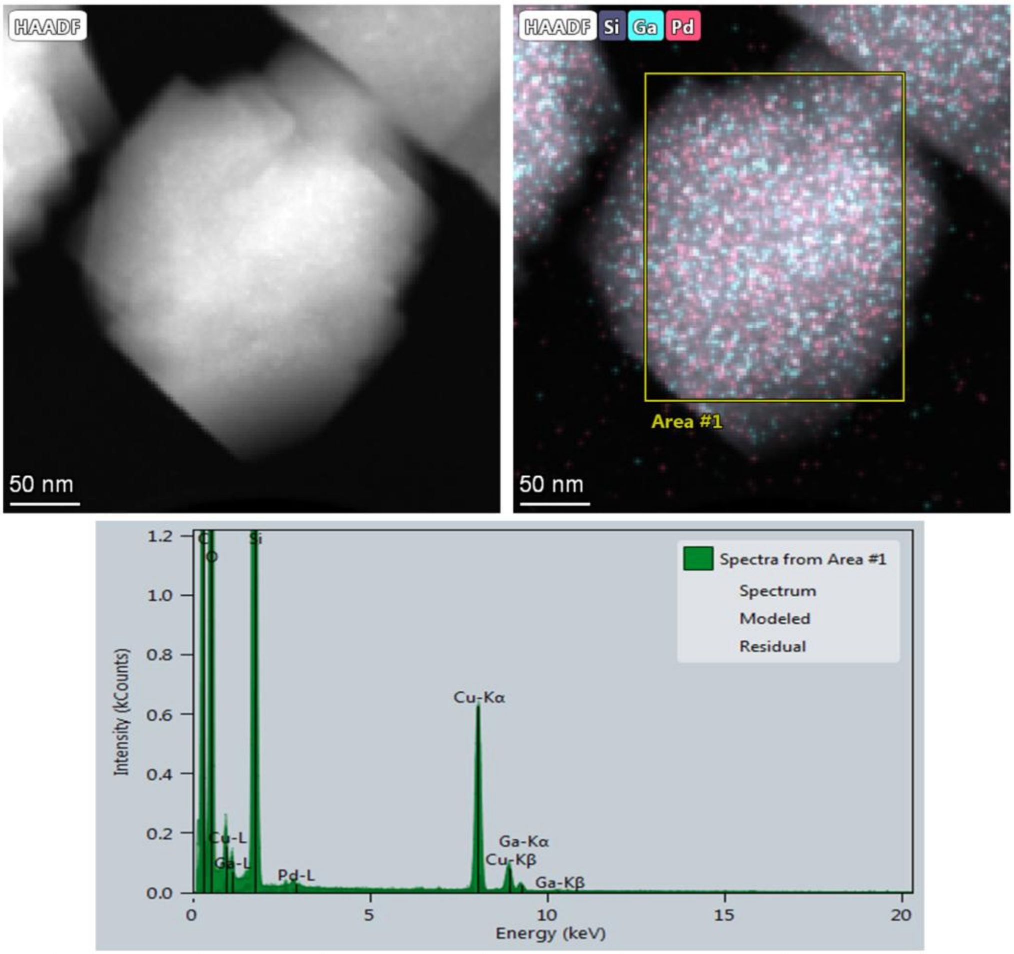

Z	Element	Family	Atomic Fraction (%)	Atomic Error (%)	Mass Fraction (%)	Mass Error (%)	Fit error (%)
31	Ga	K	64.42	7.96	54.26	4.82	1.15
46	Pd	L	35.58	5.65	45.74	6.11	1.34

Figure S56. EDX mapping of $PdGa_2$@MFI-700RED with one zeolite crystal. (It indicates Pd and Ga are uniform distributed on $PdGa_2$@MFI-700RED, the Pd/Ga molar ratio is close to theoretical ratio)

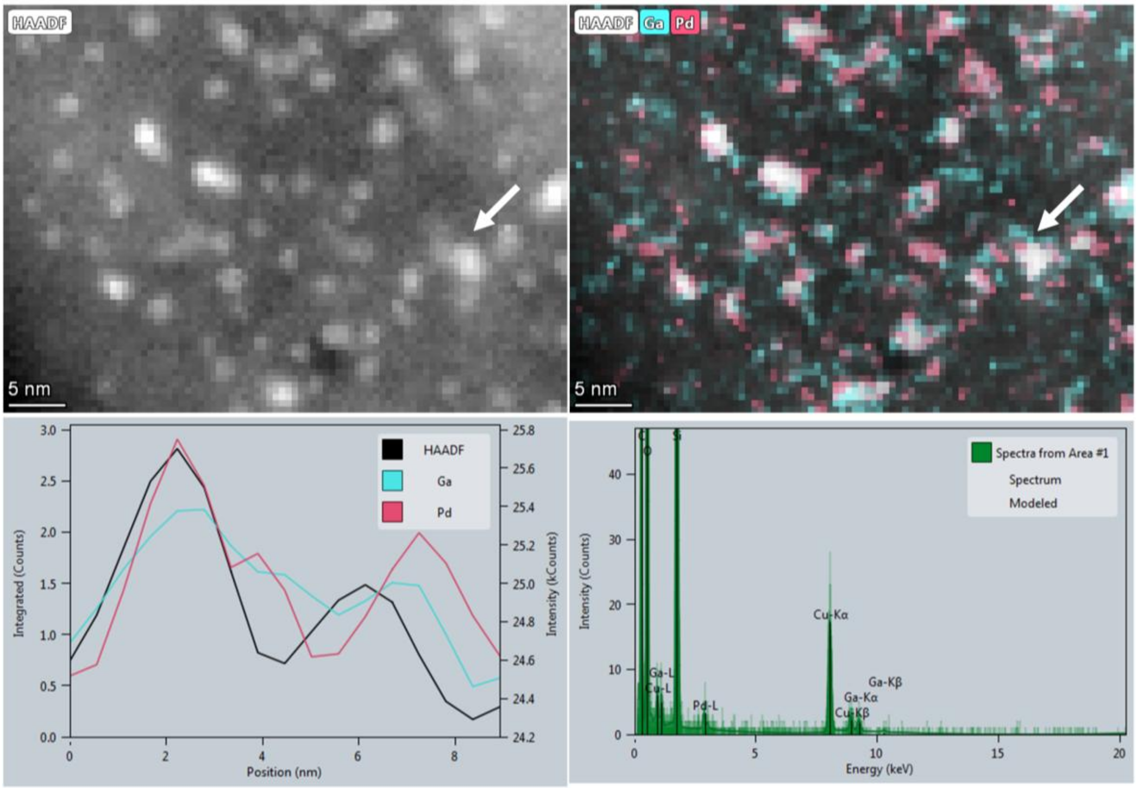

Z	Element	Family	Atomic Fraction (%)	Atomic Error (%)	Mass Fraction (%)	Mass Error (%)	Fit error (%)
31	Ga	K	52.84	9.69	42.33	5.8	8.46
46	Pd	L	47.16	9.5	57.67	9.25	6.59

Figure S57. Point analysis of $PdGa_2$@MFI-700RED with zoomed area in one selected area. Selecting most representative one point among all analyzed points. (On the one hand, it indicates Pd and Ga are uniform distributed on $PdGa_2$@MFI-700RED, on the other hand, the particle is slightly Ga-rich structure and some Ga is surrounded PdGa alloy).

5.5. H_2-D_2 isotopic exchange

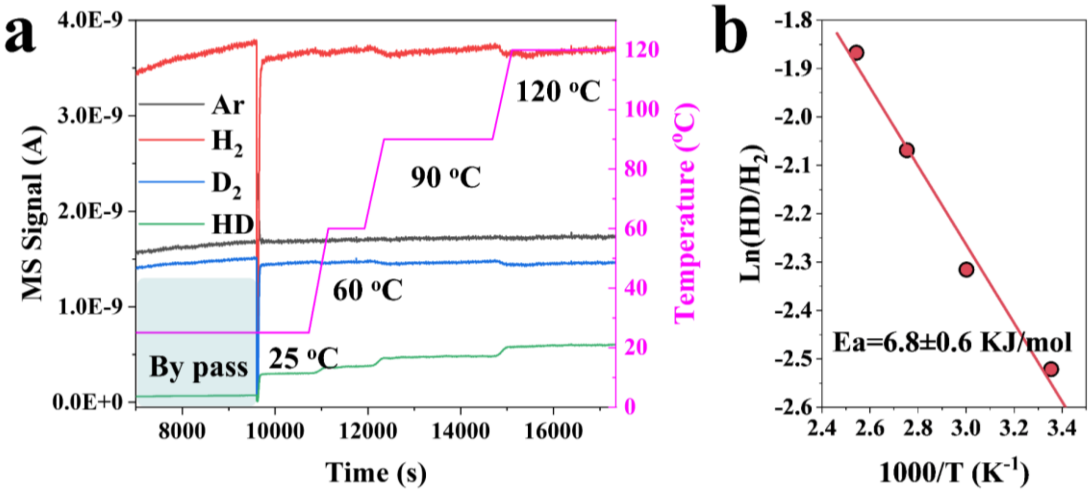

Figure S58. (a) H_2-D_2 isotopic exchange of Pd@MFI-700RED from 25 to 120 °C and (b) Apparent activation energy of HD recombination and desorption.

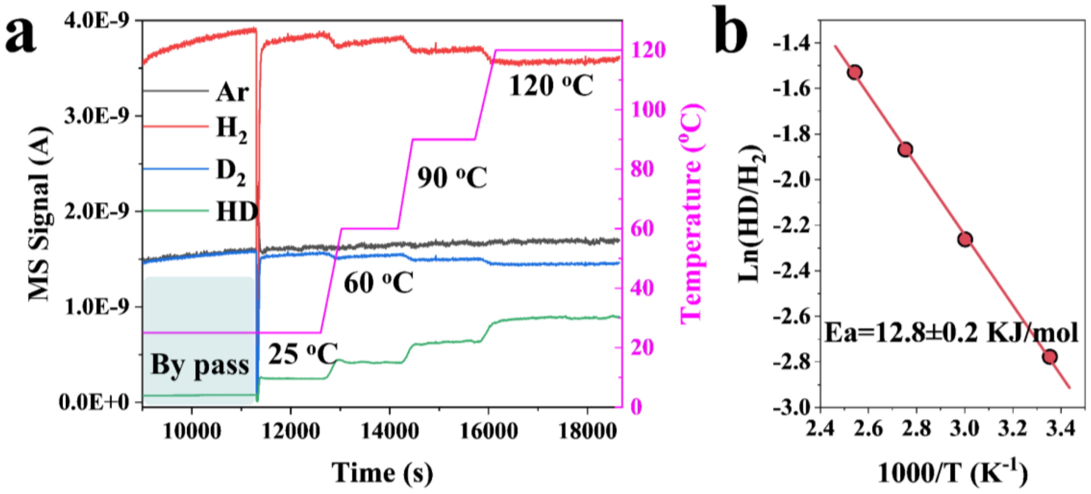

Figure S59. (a) H_2-D_2 isotopic exchange of PdGa1@MFI-700RED from 25 to 120 °C and (b) Apparent activation energy of HD recombination and desorption.

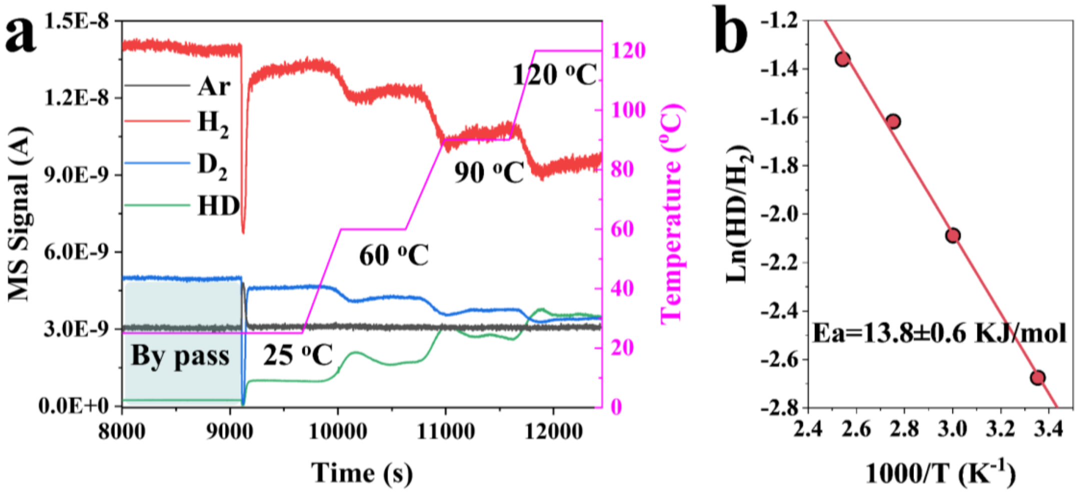

Figure S60. (a) H_2-D_2 isotopic exchange of PdGa2@MFI-700RED from 25 to 120 °C and (b) Apparent activation energy of HD recombination and desorption.

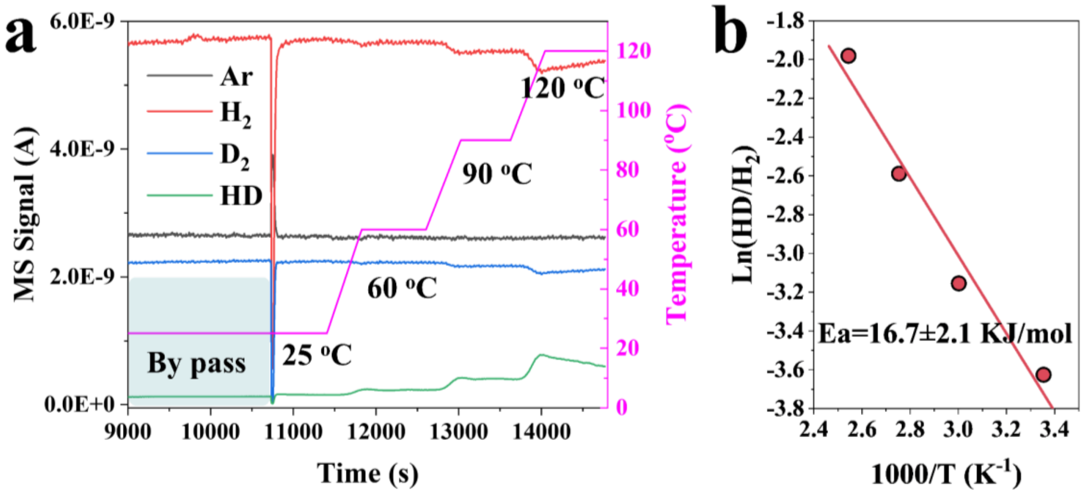

Figure S61. H_2-D_2 isotopic exchange of $PdGa_4$@MFI-700RED from 25 to 120 °C and (b) Apparent activation energy of HD recombination and desorption.

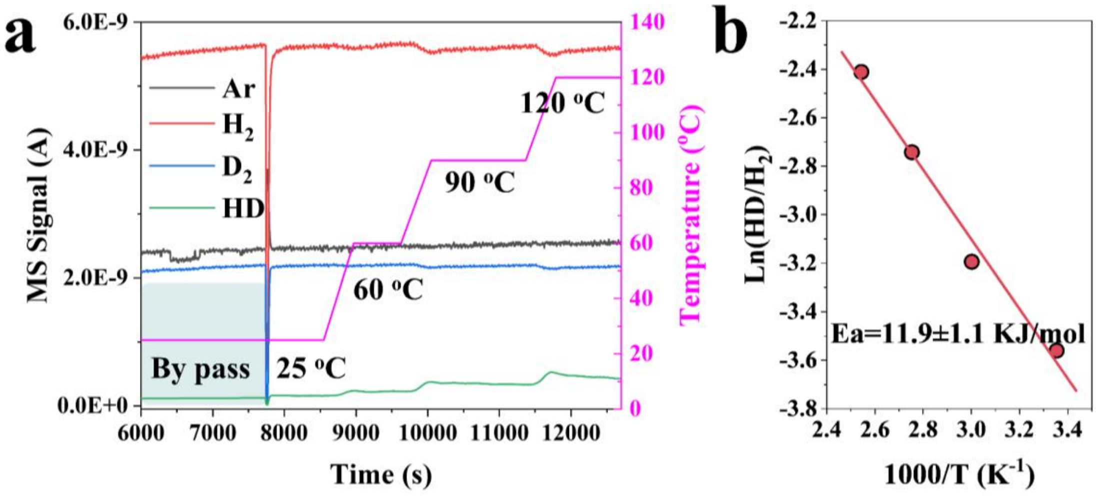

Figure S62. (a) H_2-D_2 isotopic exchange of supported 2nm $PdGa_1/SiO_2$ from 25 to 120 °C and (b) Apparent activation energy of HD recombination and desorption.

Table S7. H_2-D_2 isotopic exchange results obtained from $PdGa_x$@MFI-700RED samples.

Sample	Signal (A)	25 °C	60 °C	90 °C	120 °C	Ea (KJ/mol)
Pd@MFI-700RED	HD/H_2	8.03E-02	9.87E-02	1.26E-01	1.55E-01	6.8 ± 0.6
$PdGa_1$@MFI-700RED		6.22E-02	1.04E-01	1.54E-01	2.17E-01	12.8 ± 0.2
$PdGa_2$@MFI-700RED		1.75E-02	2.02E-02	2.74E-02	4.32E-02	13.8 ± 0.6
$PdGa_4$@MFI-700RED		2.66E-02	4.27E-02	7.51E-02	1.38E-01	16.7 ± 2.1
$PdGa_1/SiO_2$-700RED		2.84E-02	4.10E-02	6.44E-02	8.97E-02	11.9 ± 1.1

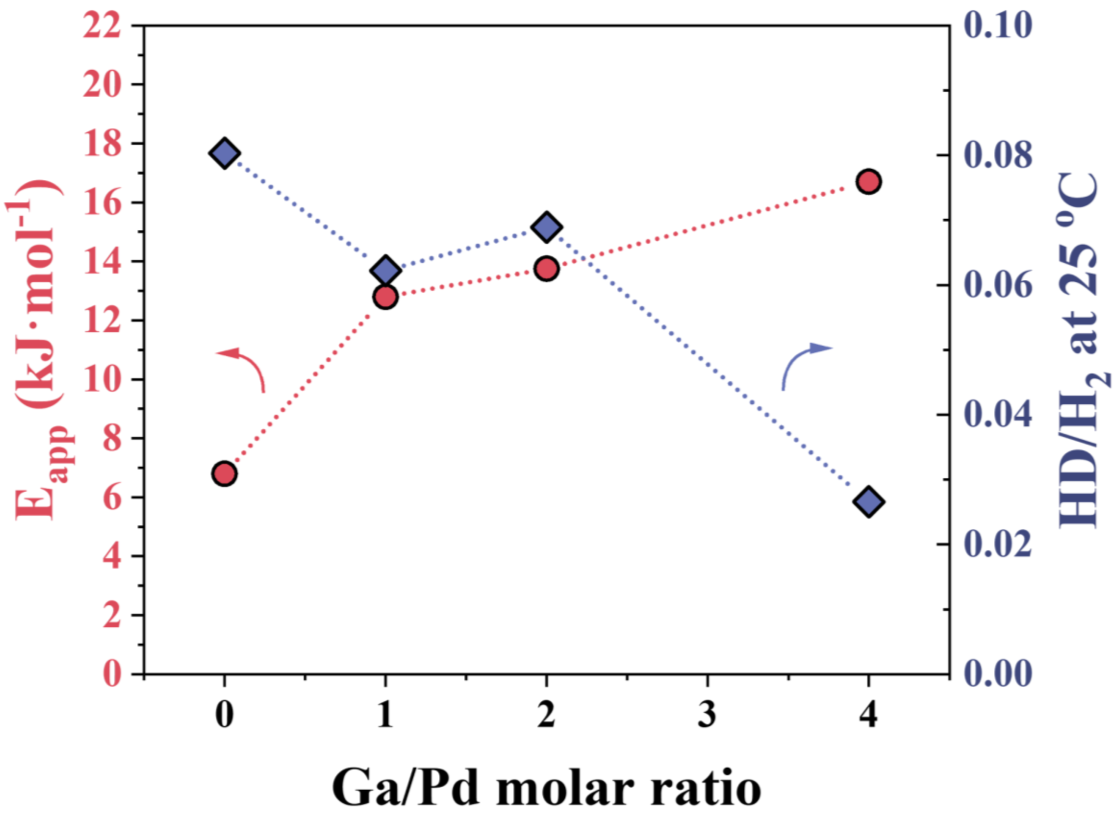

Figure S63. Variation of the HD/H_2 exchange level at 25 °C (right axe) and of the apparent activation energy of HD formation and desorption in the H_2-D_2 isotopic exchange experiments (left axe) over $PdGa_x$@MFI-700RED samples with the Ga/Pd molar ratio from 0 to 4.

6. Catalytic performance of $PdGa_x$@MFI in CO_2 hydrogenation

6.1. Activity evaluation of $PdGa_4$@MFI

The catalytic performance of the $PdGa_4$@MFI catalysts reduced at different temperatures is shown in Table S8. In all cases methanol, DME and CO are formed. As shown in Figure S64, there is a volcanic-type trend for different temperatures.

Table S8. Catalytic performance of $PdGa_4$@MFI catalysts activated at different reduction temperatures (500, 600, 700 and 800 °C).

Sample	Temp. (°C)	Conver. (%)	Selec. (%) MeOH+DME (MeOH/DME/CO)	Yield for MeOH and DME formation		
				Per mass of catalyst ($g_{MeOH+DME} \cdot kg_{cat}^{-1} \cdot h^{-1}$)	Per mass of Pd ($g_{MeOH+DME} \cdot kg_{Pd}^{-1} \cdot h^{-1}$)	TOF Per mole of surface exposed Pd sites ($mol_{MeOH+DME} \cdot mol_{Pd\ sur}^{-1} \cdot h^{-1}$)
$PdGa_4$@MFI -500RED	220	0.2	21 (9/12/79)	2.11	306.49	3.57
	240	0.3	30 (10/20/68)	6.29	911.21	10.32
	260	0.7	29 (10/19/69)	11.36	1645.80	18.67
	280	1.0	25 (10/15/73)	14.80	2144.68	24.75
	300	1.6	19 (9/10/78)	17.30	2507.58	29.63
$PdGa_4$@MFI -600RED	220	0.4	58 (17/41/41)	13.97	2253.38	24.76
	240	0.9	66 (16/50/33)	38.79	6256.32	67.63
	260	1.6	63 (16/47/36)	61.07	9850.48	107.04
	280	2.4	56 (16/40/43)	82.04	13232.41	145.36
	300	3.4	47 (16/31/51)	96.25	15524.98	173.22
$PdGa_4$@MFI -700RED	220	1.1	78 (19/59/22)	53.07	8844.55	198.81
	240	2.0	78 (18/60/22)	97.48	16247.28	363.45
	260	3.3	75 (18/57/25)	153.95	25658.51	575.27
	260 [a]	5.2	80 (19/61/20)	257.19	42864.47	959.91
	280	5.1	70 (18/52/30)	218.77	36461.47	822.70
	300	7.0	62 (18/44/38)	264.72	44120.55	1006.01
$PdGa_4$@MFI -800RED	220	0.5	79 (18/61/21)	23.88	3980.29	415.80
	240	0.9	76 (17/59/23)	42.24	7040.20	734.87
	260	1.6	74 (18/56/26)	69.17	11527.69	1210.01
	280	2.9	70 (18/52/30)	115.94	19323.00	2040.92
	300	4.8	65 (19/46/36)	177.36	29559.92	3151.76

Reaction conditions: $CO_2/H_2 = 1/3$, WHSV = 15000 $mL \cdot g_{cat}^{-1} \cdot h^{-1}$, Flow = 50 mL/min, T = 220-300 °C and P = 20 bar. All $PdGa_x$@MFI catalysts are *in-situ* reduced at 350 °C under 20 mL/min H_2 for 2h.

[a] Reaction conditions similar as before but at 45 bar.

Table S9. Catalytic performance of $PdGa_x$@MFI-700RED catalysts with different Ga/Pd ratio at different reaction temperatures.

Sample	Temp. (ºC)	Conver. (%)	Selec. (%) MeOH+DME (MeOH/DME/CO)	Yield to MeOH and DME		
				Per mass of catalyst ($g_{MeOH+DME}\cdot kg_{cat}^{-1}\cdot h^{-1}$)	Per mass of Pd ($g_{MeOH+DME}\cdot kg_{Pd}^{-1}\cdot h^{-1}$)	TOF Per mole of surface exposed Pd sites ($mol_{MeOH+DME}\cdot mol_{Pd\ sur}^{-1}\cdot h^{-1}$)
Pd@MFI-700RED	220	0.1	4 (4/0/92)	0.11	15.16	0.24
	240	0.1	9 (9/0/86)	0.42	60.58	0.96
	260	0.2	9 (9/0/82)	0.73	104.83	1.65
	280	0.3	8 (8/0/82)	0.99	140.78	2.22
	300	0.4	6 (6/0/80)	1.10	156.98	2.48
$PdGa_{0.5}$@MFI-700RED	220	0.1	3 (3/0/93)	0.09	13.87	0.26
	240	0.2	10 (8/2/87)	0.88	129.29	2.19
	260	0.4	9 (7/2/89)	1.74	255.49	4.29
	280	0.7	9 (6/3/90)	2.91	427.29	7.09
	300	1.1	7 (5/2/92)	3.71	545.40	9.16
$PdGa_1$@MFI-700RED	220	0.4	65 (49/16/35)	23.10	3300.04	50.90
	240	1.3	64 (41/23/36)	43.71	6244.92	92.22
	260	2.1	60 (32/28/40)	70.29	10040.88	142.13
	280	3.2	54 (24/30/46)	97.69	13956.32	191.52
	300	4.5	44 (18/26/55)	115.75	16535.64	224.51
$PdGa_2$@MFI-700RED	220	1.0	76 (31/45/24)	44.33	6926.76	120.66
	240	1.8	75 (25/50/25)	79.00	12344.18	208.93
	260	2.8	72 (21/51/28)	121.81	19032.27	317.80
	280	4.2	67 (19/48/32)	170.46	26634.51	443.95
	300	5.9	59 (18/41/40)	211.55	33055.42	555.18
$PdGa_4$@MFI-700RED	220	1.1	78 (19/59/22)	53.07	8844.55	198.81
	240	2.0	78 (18/60/22)	97.48	16247.28	363.45
	260	3.3	75 (18/57/25)	153.95	25658.51	575.27
	260 [a]	5.2	80 (19/61/20)	257.19	42864.47	959.91
	280	5.1	70 (18/52/30)	218.77	36461.47	822.70
	300	7.0	62 (18/44/38)	264.72	44120.55	1006.01
$PdGa_4$@MFI-800RED	220	0.5	79 (18/61/21)	23.88	3980.29	415.80
	240	0.9	76 (17/59/23)	42.24	7040.20	734.87
	260	1.6	74 (18/56/26)	69.17	11527.69	1210.01
	280	2.9	70 (18/52/30)	115.94	19323.00	2040.92
	300	4.8	65 (19/46/36)	177.36	29559.92	3151.76
$PdGa_4$@MFI-Cal700RED	220	0.1	13 (8/5/85)	0.90	231.57	8.83
	240	0.3	50 (13/37/49)	9.59	2458.13	83.15

	260	0.5	49 (14/35/50)	15.27	3914.87	133.58
	280	0.8	42 (14/28/57)	21.05	5398.52	186.82
	300	1.4	37 (14/23/62)	30.83	7905.82	277.02

Reaction conditions: CO_2/H_2 = 1/3, WHSV = 15000 mL·g_{cat}^{-1}·h^{-1}, Flow = 50 mL/min, T = 220-300 ºC and P = 20 bar. All $PdGa_x$@MFI catalysts are *in-situ* reduced at 350 ºC under 20 mL/min H_2 for 2h.

[a] Reaction conditions similar as before but at 45 bar.

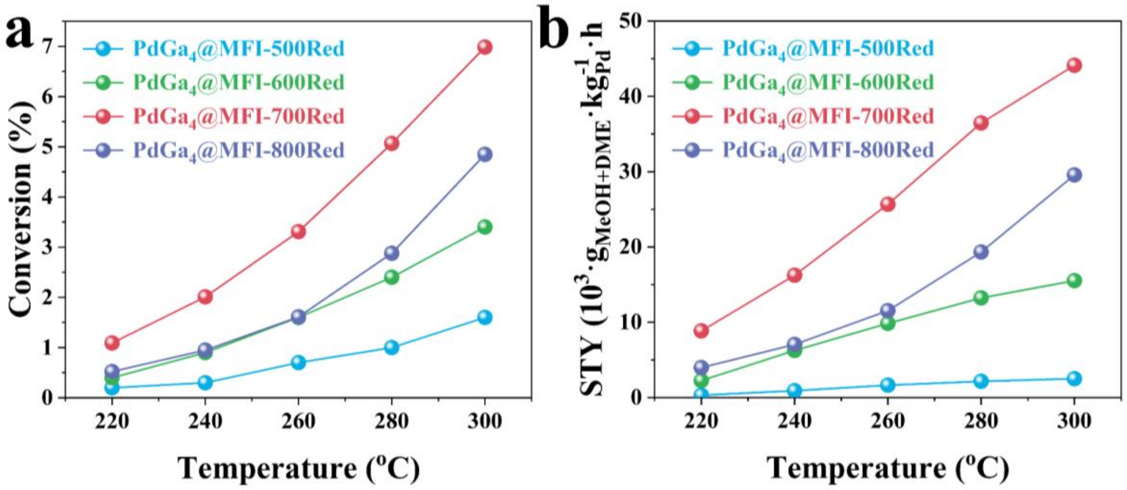

Figure S64. CO_2 hydrogenation activity on $PdGa_4$@MFI catalysts reduced at different temperatures. (a) Temperature versus CO_2 conversion, (b) Temperature versus space time yield (STY) to oxygenates. Reaction conditions: WHSV = 15000 mL·g_{cat}^{-1}·h^{-1}, Flow = 50 mL/min, T = 220-300 ºC, P = 20 bar.

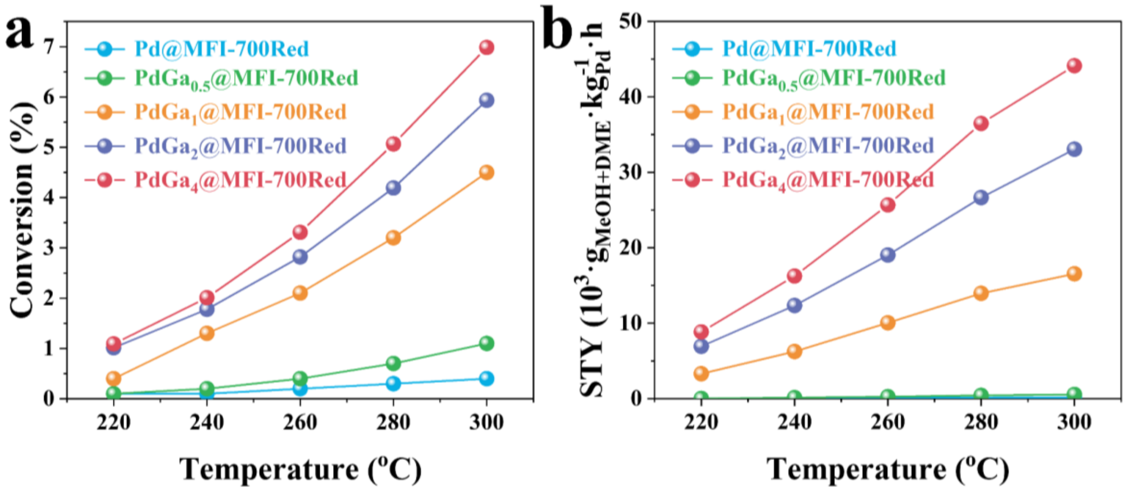

Figure S65. CO_2 hydrogenation activity on $PdGa_x$@MFI-700RED catalysts with different Ga/Pd ratio (a) Temperature versus CO_2 conversion, (b) Temperature versus STY of oxygenates. Reaction conditions: WHSV = 15000 mL·g_{cat}^{-1}·h^{-1}, Flow = 50 mL/min, T = 220-300 ºC, P = 20 bar.

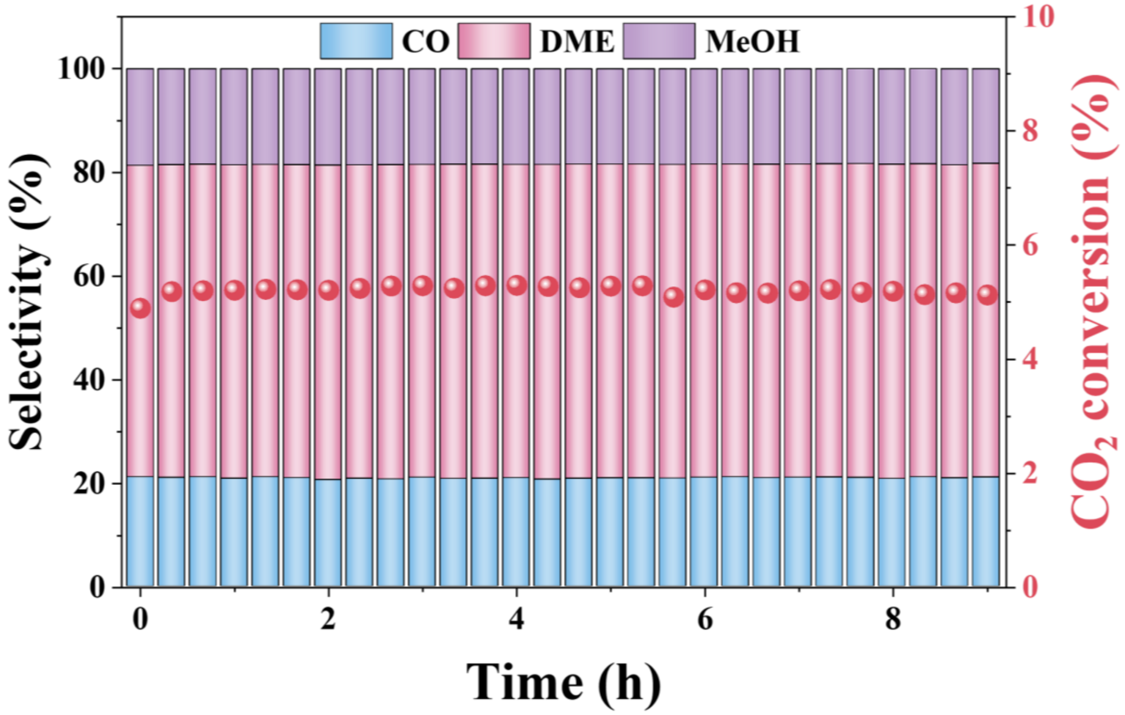

Figure S66. CO_2 hydrogenation activity on $PdGa_4$@MFI-700RED catalyst at 260 ºC and 45 bar. Reaction conditions: WHSV = 15000 mL·g_{cat}^{-1}·h^{-1}, Flow=50 mL/min, T=260 ºC, P = 45 bar.

6.2. Characterization of used catalyst after long-term stability test.

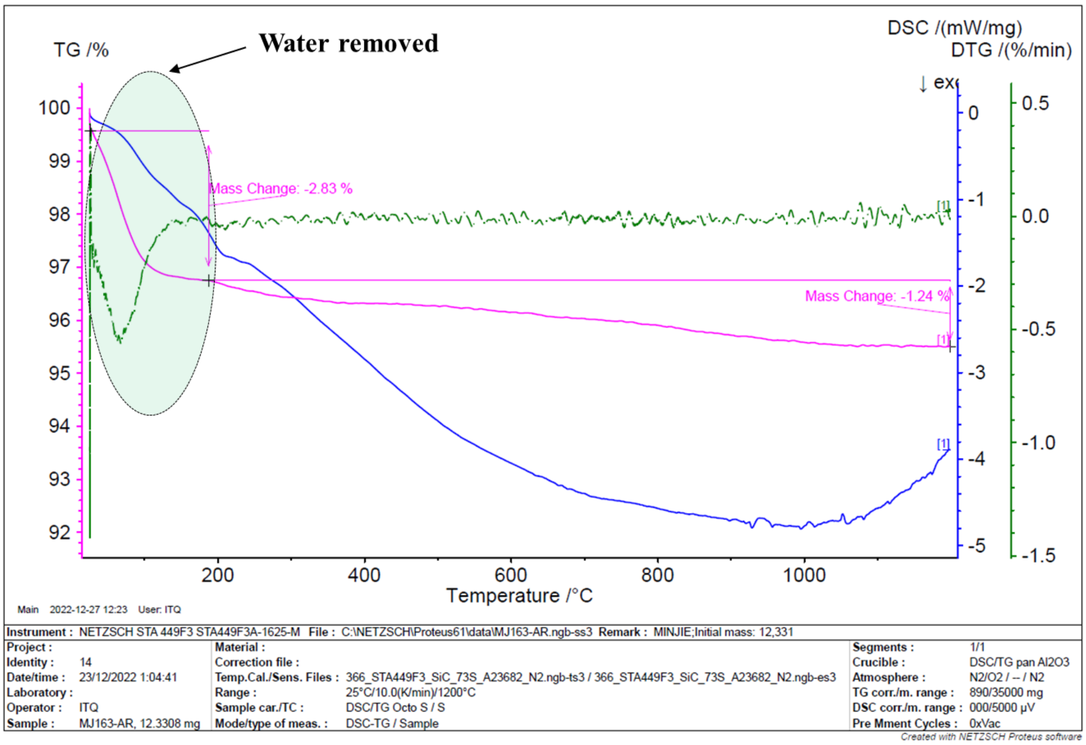

Figure S67. TG-DSC result of 0.6wt% $PdGa_4$@MFI-700RED after long-term stability test.

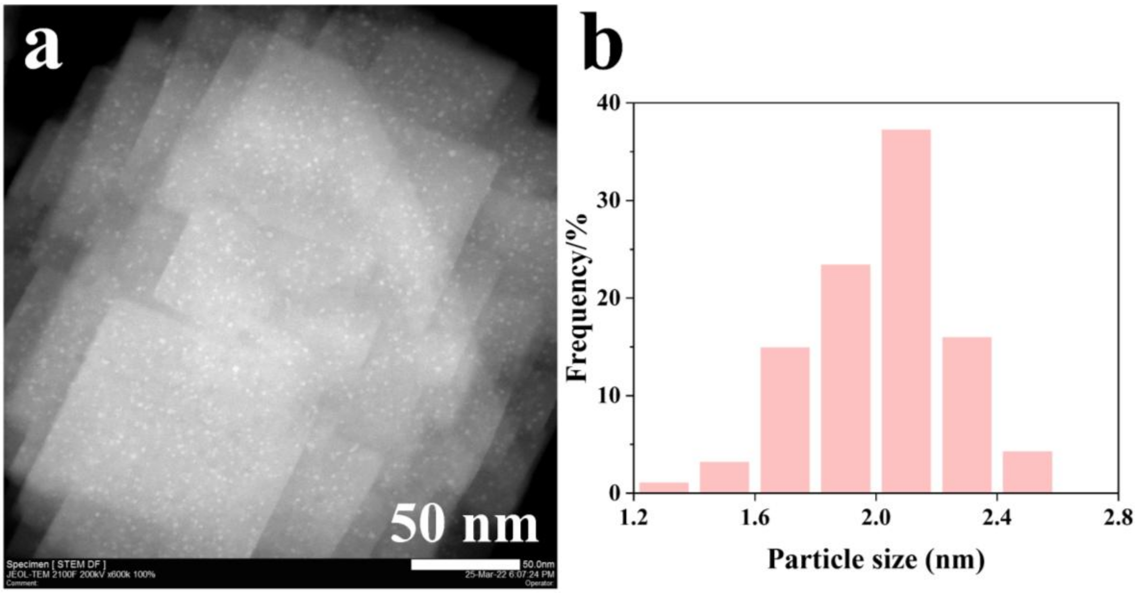

Figure S68. STEM result of $PdGa_4$@MFI-700RED catalyst after 60h long-term stability test. Particle size distribution on the right side. The average particle sizes of each sample were calculated by the following equation within a statistical analysis of 50 particles: $d_{TEM} = \Sigma n_i d_i^3 / \Sigma n_i d_i^2$.

6.3. State-of-the-art catalysts for CO_2 hydrogenation

Table S10. Comparison of CO_2 hydrogenation performances of PdGa catalysts with literature.

Entry	Catalyst	Preparation	Pd loading (wt%)	Particle size (nm)	GHSV (h^{-1})	WHSV ($mL\cdot g_{cat}^{-1}\cdot h^{-1}$)	T (^oC)	P (bar)	Conver. (%)	Selec. (%)[a]	Yield (%)[b]	Yield to MeOH and DME		reference
												$g_{MeOH+DME}\cdot kg_{cat}^{-1}\cdot h^{-1}$	$g_{MeOH+DME}\cdot kg_{Pd}^{-1}\cdot h^{-1}$	
1	$PdGa_4$@MFI	One-pot	0.6	2.2	6000	15000	260	20	3.3	75	2.5	154	25659	This work
2	$PdGa_4$@MFI	One-pot	0.6	2.2	6000	15000	260	45	5.2	80	4.2	257	42864	This work
3	$PdGa_4$@MFI	One-pot	0.6	2.2	6000	15000	280	20	5.1	70	3.6	219	36461	This work
4	$PdGa_4$@MFI	One-pot	0.6	2.2	6000	15000	300	20	7.0	62	4.3	265	44120	This work
	$PdGa_1/SiO_2$	Impregnation	0.6	2.5	6000	15000	260	20	2.9	71	2.1	93	14349	This work
5	Pd@8cGa_2O_3	ALD[c]	1	4.1±0.9		15000	250	45	7.1	70.4	5.0	270	26960	[14]
6	Pd@8cGa_2O_3	ALD	1	4.1±0.9		6000	250	45	11.6	69.5	8.1	174	17400	[14]
7	Pd/Ga_2O_3	ALD	1	4.5±1.2		15000	250	45	2.4	45.7	1.1	57	5670	[14]
8	Pd/Al_2O_3	ALD	1			15000	250	45	0.9	24.5	<0.1	12	1200	[14]
9	Pd/SiO_2	SOMC[d]	1.6	1.6±0.4		30000	230	25	~1	20	<0.1		~30	[15]
	PdGa/SiO_2	SOMC	1.1	1.6±0.4		30000	230	25	~1	81	<0.1			[15]
10	PdGa/SiO_2	SOMC	1.1	1.6±0.4		30000	230	25	<5	81	<3.8		~5619	[15]
11	Pd/Plate Ga_2O_3	Impregnation	5	1-5		6000	250	50	17.3	52	9.0	NA	~3674	[32]
12	Pd/Rod Ga_2O_3	Impregnation	5			6000	250	50	11.0	41.3	4.5		~1870	[32]
13	Pd/Plate Ga_2O_3	Impregnation	1			6000	250	50	11.0	29.5	3.2		6700	[32]
14	Pd/Rod Ga_2O_3	Impregnation	1			6000	250	50	8.7	45.2	3.9		8120	[32]
15	Pd/Ga_2O_3		10			18000	250	50	19.6	51.5	10.1		6253	[33]
16	Pd_2Ga/α-Ga_2O_3		1			NA	230	30	~3.0	~57	~1.7		~8640	[34]

17	$Pd_2Ga/\alpha\text{-}Ga_2O_3$		1			NA	250	30	~6.0	~50	~3.0		~13440	[34]
18	$Pd/\beta\text{-}Ga_2O_3$	Impregnation	1	2.8		80000	250	30	<1	52	<1		~25300	[35]
19	$Pd/\beta\text{-}Ga_2O_3$	Impregnation	1			82000	250	30	0.9	52	<1		~25300	[36]
20	$PdGa(1.0)/SiO_2$	Impregnation	4.5	15±9		4800	280	8	21.7	57	12.4		~4540	[37]
21	$PdGa(80)/SiO_2$	Impregnation	2	2.8		7800	250	30	1.6	52.2	0.8		~1050	[38]
22	$PdGa(80)/SiO_2$	Impregnation	2	2.8		78000	250	30	0.7	60.5	0.4		~6350	[38]
23	Pd@Zn-(26.4CdSe)		5			18000	250	20	6.7	67.3	4.5		6100	[39]
24	Pd@Zn-(26.4CdSe)		5			18000	250	45	~7.9	77.6	~6.1		8300	[39]
25	Pd/ZnO	ALD	0.4	2.5		15000	250	45	~2.0	~77	~1.5		18000	[40]
26	Pd/ZnO	ALD	5.2	7.9		15000	250	45	~3.5	~80	~2.8		~3000	[40]
27	Pd/ZnO	Impregnation	2	2±0.2		60000	260	50	2.8	66.5	1.9	384	19200	[41]
28	$PdZn/ZnO/SiO_2$	Impregnation	3.7	2-20		60000	260	50	3.3	65.3	2.2	443	11973	[41]
29	$Cu/ZnO/Al_2O_3$		~50 Cu			21600	250	45	~20	~55	~11		~1700	[42]

[a] Selectivity of methanol and DME.
[b] Yield of methanol and DME.
[c] ALD: atomic layer deposition.
[d] SOMC: surface organometallic chemistry.

Table S11. Tandem CO_2 hydrogenation to DME performance.

Entry	Hybrid Catalyst	Preparation	H_2/CO_2 ratio	GHSV (h^{-1})	WHSV ($mL\cdot g_{cat}^{-1}\cdot h^{-1}$)	T (°C)	P (bar)	Conver. (%)	DME Selec. (%)	DME Yield (%)	Yield to DME		reference
											$g_{DME}\cdot kg_{cat}^{-1}\cdot h^{-1}$	$g_{DME}\cdot kg_{metal}^{-1}\cdot h^{-1}$	
1	$PdGa_4$@MFI	One-pot	3	6000	15000	260	20	3.3	57	1.9	127	21107	This work
2	$PdGa_4$@MFI	One-pot	3	6000	15000	260	45	5.2	61	3.2	212	35383	This work
3	$PdGa_4$@MFI	One-pot	3	6000	15000	280	20	5.1	52	2.7	177	29420	This work
4	$PdGa_4$@MFI	One-pot	3	6000	15000	300	20	7.0	44	3.1	207	34448	This work
	$PdGa_4$@MFI	One-pot	3	1000	2100	260	20	11.1	56.2	6.2	56	9259	This work
	GaN[a]	Thermal polymerization	2		3000	360	20	6.3	32	2.0	39	-	[43]
	$CaCO_3$-GaN[a]	Thermal polymerization	2		3000	360	20	10.7	21	2.2	134	-	[43]
	$GaZrO_x$[b]	EISA[c]	3		24000	330	30	9.0	21	1.9	213	-	[44]
30	5wt% PdZn/HZSM-5	Chemical vapor impregnation	3		3600	270	20	13.7	30.4	4.2	25	501	[45]
31	5wt% PdZn/TiO_2/HZSM-5	Chemical vapor impregnation	3		3600	270	20	11	32.2	3.6	31	615	[45]
32	5wt% PdZn/TiO_2/γ-Al_2O_3	Chemical vapor impregnation	3		3600	270	20	14	29.1	4.1	24	486	[45]
33	PdZn@meso-HZSM-5	SOMC	3		6000	300	20	13	17	2.2	62	207	[46]
34	PdZn@ZnO/HZSM-5	Physical mixture	3		6000	300	20	20	35	7.0	197	309	[46]
35	Pd/ZnO/Hierarchically H-ZSM-5	Physical mixture	3		12000	300	30	10.8	31	3.3	189	3150	[47]
36	CuO-ZnO-ZrO_2/HZSM-5	Impregnation	3		3600	250	30	22.2	67.6	15.0	507	1491[d]	[48]
37	CuO-ZnO-ZrO_2/WO_x-ZrO_2	Physical mixture	3		13000	260	20	15.3	28.1	4.3	141	549[d]	[49]

	CuO-ZnO-ZrO_2/H-Ga-silicate	Physical mixture	3		3 (1g catalyst; W/F=20 $g_{cat}\cdot h\cdot mL^{-1}$)	250	28	19.0	45.3	8.6	NA	NA	50
38	CuO-ZnO-ZrO_2/Ferrierite	Impregnation	3		8800	260	50	26.0	55.7	14.5	~600	1326[d]	51
39	CuO-ZnO-ZrO_2/MOR	Impregnation	3		8800	260	50	23.2	50.8	11.8	~500	1113[d]	51
	CuO-ZnO-Al_2O_3/HZSM-5	Co-precipitation	3		750	275	40	~35	~67	23.5	83	NA	52
	CuO-ZnO-Al_2O_3/γ-Al_2O_3	Co-precipitation	3		750	275	40	~27	~30	8.1	29	NA	52
40	CuO-ZnO-Al_2O_3/HZSM-5	Physical mixture	3		3000	260	50	31	65	20.2	284	613[d]	53
41	CuO-ZnO-Al_2O_3/La-ZSM-5	Physical mixture	3		3000	250	30	43.8	71.2	31.2	440	950[d]	54
42	CuO-ZnO-Al_2O_3/H-ZSM-5 (Commercial)	Physical mixture	3		48000	225	30	2.2	73.5	1.6	382	864[d]	55
43	CuO-ZnO-Al_2O_3/H-ZSM-5 (Nano)	Physical mixture	3		48000	225	30	2.9	75.6	2.2	510	1154[d]	55
44	CuO-ZnO-Al_2O_3/H-ZSM-5 (Hollow)	Physical mixture	3		48000	225	30	2.3	72.3	1.7	390	882[d]	55
45	CuO-ZnO-Al_2O_3/$H_3PW_{12}O_{40}$/MCM-41	Physical mixture	3		40000	250	45	8.9	23.1	2.1	1552	2989[d]	56
46	CuO-In_2O_3-ZrO_2/SAPO-34	Physical mixture	3		6000	250	30	4.3	60.6	2.6	81	1319[d]	57
47	HZSM-5/CuO-ZnO-ZrO_2	*In-situ* growing MFI on CZZ	3		2400	250	30	8.6	60	5.2	58	318[d]	58
48	CuGa/SiO_2-Hydrophilic	Hydrophobic treatment	3		6000	240	30	6.2	45	2.8	79	132[d]	59
49	CuO-ZnO-Al_2O_3/Ferrierite	Physical coating	3		5000	260	50	24.6	54.4	13.4	400.8	902[d]	60
50	Pd/In_2O_3/H-ZSM-5	Physical mixture	3		15360	290	30	9.0	44.1	4.0	80	7729	61

[a] One step DME synthesis.
[b] DME is by-product, not sure process.
[c] EISA: Evaporation-induced self-assembly.
[d] Based on catalyst Cu weight loading, unit: $g_{DME}\cdot kg_{Cu}^{-1}\cdot h^{-1}$.

For the literature not reported DME formation rate, the calculation is based on:

$$STY\ (g_{DME}\cdot kg_{cat}^{-1}\cdot h^{-1}) = \frac{WHSV}{V_m\ at\ 25\ ℃} * V\%_{CO_2} * X_{CO_2} * S_{DME} * M_{DME}$$

Where $V\%_{CO2}$ is the volume fraction of CO_2, X_{CO2} is the conversion, S_{DME} is the selectivity of DME and M_{DME} is the molar mass of DME.

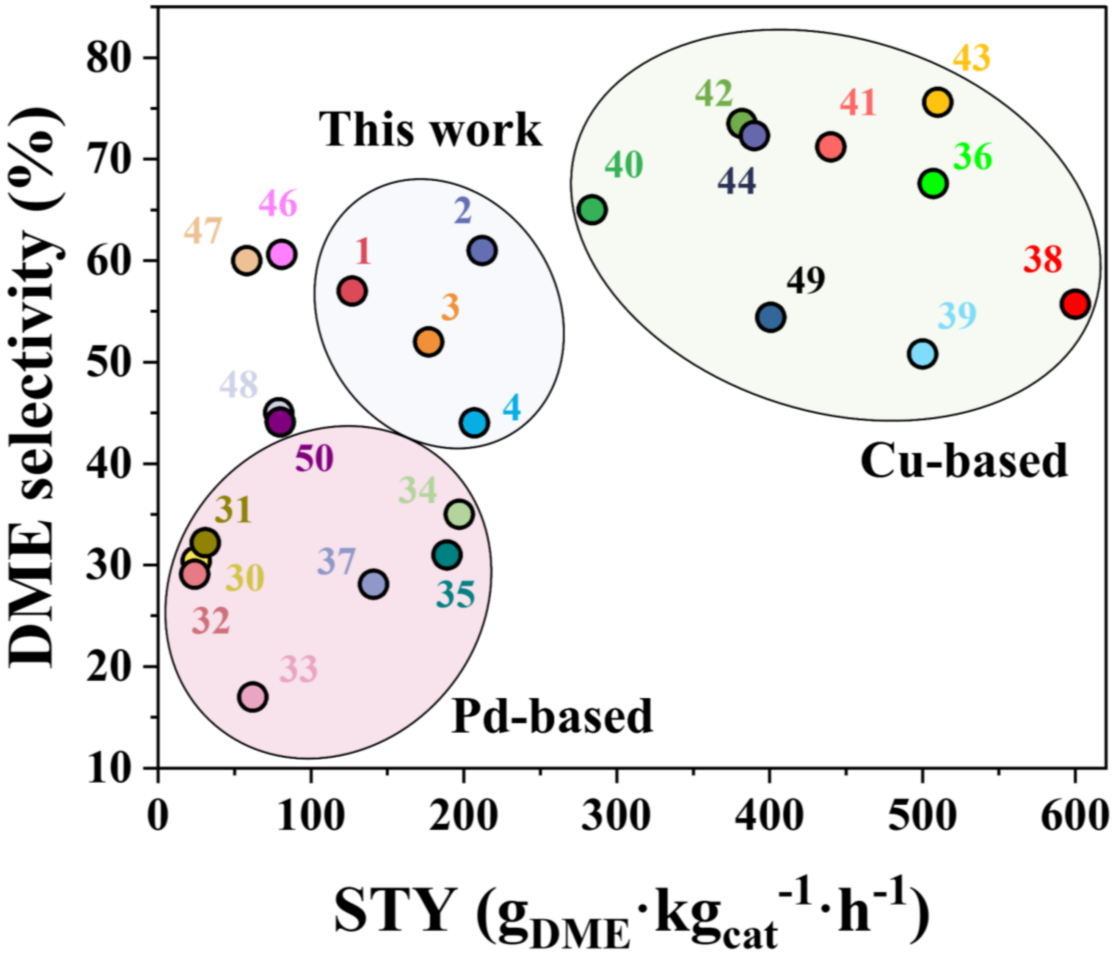

Figure S69. Compared with state-of-the-art CO_2 hydrogenation to DME catalysts (X axis is STY with $g_{DME}\cdot kg_{cat}^{-1}\cdot h^{-1}$ unit).

6.5. Kinetics studies

6.5.1. Initial reaction rates to each product on PdGa$_x$@MFI-700 (x=1 and 4) and on supported PdGa$_1$/SiO$_2$ catalysts.

In order to get initial selectivity at zero CO_2 conversion and initial reaction rate, experiments at various contact time are conducted on PdGa$_x$@MFI-700RED and supported PdGa$_1$/SiO$_2$ catalysts. The experimental conditions are outlined in Figure S70. Second-order polynomial fits are used to extrapolate selectivity to zero CO_2 conversion. The initial reaction rate is calculated at low contact time with CO_2 conversion below 5%, ensuring the measurements under kinetic region.

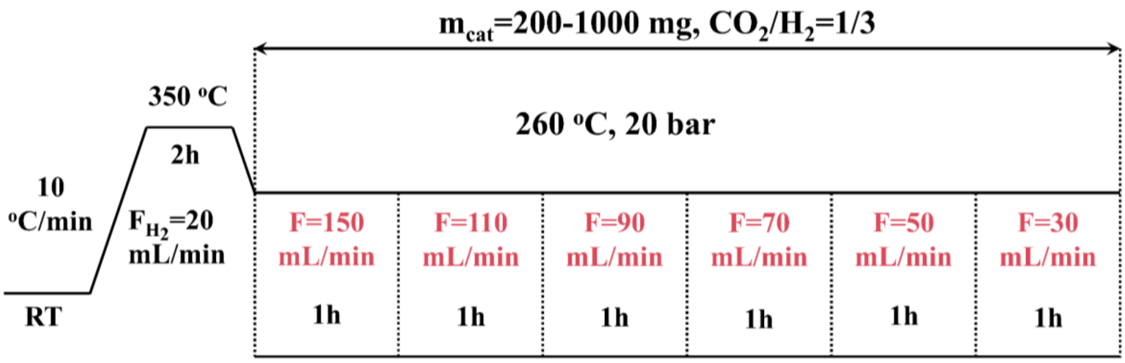

Figure S70. Schema of various contact time experiment details.

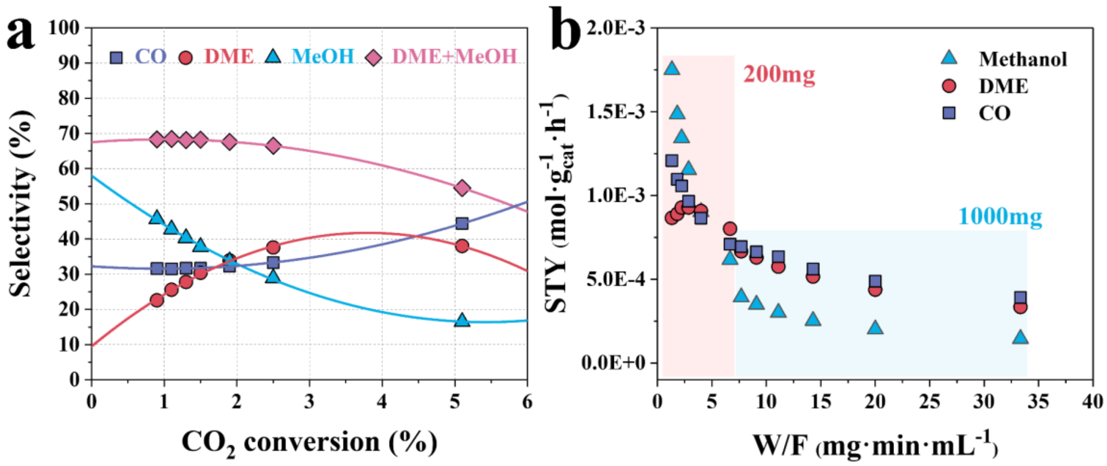

Figure S71. (a) CO, DME and methanol selectivity as a function of CO_2 conversion (Second-order polynomial fits is used to extrapolate selectivity to zero CO_2 conversion). (b) STY to each product at different contact time on $PdGa_1$@MFI-700RED catalyst. Reaction condition: 200-1000 mg catalyst, T=260 ºC, 20 bar, Flow=150-30 mL/min.

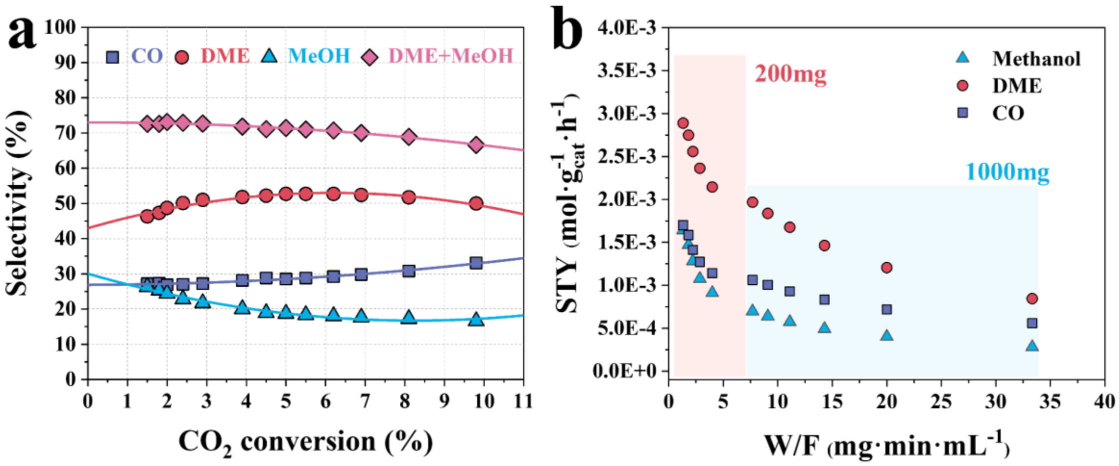

Figure S72. (a) CO, DME and methanol selectivity as a function of CO_2 conversion (Second-order polynomial fits is used to extrapolate selectivity to zero CO_2 conversion). (b) STY to each product at different contact time on $PdGa_2$@MFI-700RED catalyst. Reaction condition: 200-1000 mg catalyst, T=260 ºC, 20 bar, Flow=150-30 mL/min.

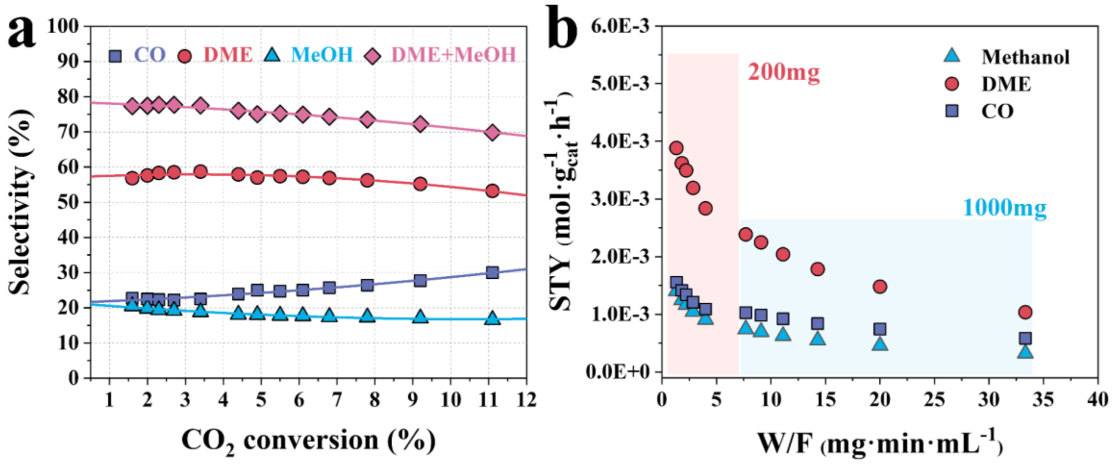

Figure S73. (a) CO, DME and methanol selectivity as a function of CO_2 conversion (Second-order polynomial fits is used to extrapolate selectivity to zero CO_2 conversion). (b) STY to each product at different contact time on $PdGa_4$@MFI-700RED catalyst. Reaction condition: 200-1000 mg catalyst, T=260 ºC, 20 bar, Flow=150-30 mL/min.

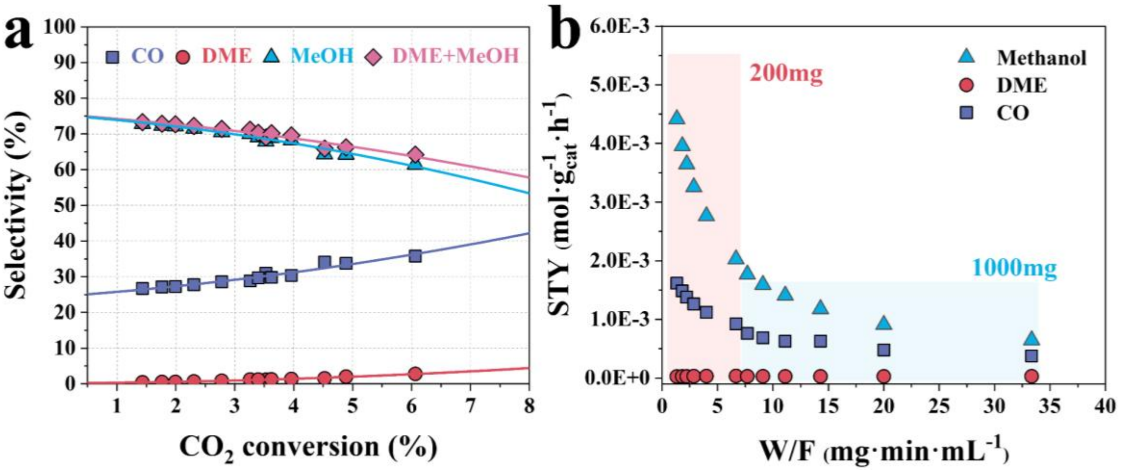

Figure S74. (a) CO, DME and methanol selectivity as a function of CO_2 conversion (Second-order polynomial fits is used to extrapolate selectivity to zero CO_2 conversion). (b) STY to each product at different contact time on $PdGa_1/SiO_2$-700RED catalyst. Reaction condition: 200-1000 mg catalyst, T=260 ºC, 20 bar, Flow=150-30 mL/min.

Table S12. Catalytic data of $PdGa_x$@MFI-700RED catalysts at different contact time, keeping the reaction temperature at 260 ºC and 20 bar during experiments.

Sample	Contact time ($mg·min·mL^{-1}$)	Conversion (%)	Selectivity (%)			STY ($mmol·g_{cat}^{-1}h^{-1}$)		
			MeOH	DME	CO	MeOH	DME	CO
$PdGa_1$@MFI-700RED	1.33	0.9	45.7	22.6	31.6	1.75	0.87	1.21
	1.82	1.1	42.8	25.6	31.5	1.49	0.89	1.10
	2.22	1.3	40.3	27.8	31.7	1.34	0.93	1.06
	2.86	1.5	37.8	30.4	31.7	1.15	0.93	0.97
	4	1.9	33.7	33.9	32.3	0.90	0.91	0.86
	33.33	5.1	16.5	38.0	44.4	0.15	0.33	0.39
$PdGa_2$@MFI-700RED	1.33	1.5	26.3	46.3	27.2	1.64	2.89	1.70
	1.82	1.8	25.3	47.3	27.3	1.47	2.75	1.58
	2.22	2	24.4	48.7	26.9	1.28	2.56	1.41
	2.86	2.4	22.8	50.1	27	1.08	2.36	1.27
	4	2.9	21.7	51	27.2	0.91	2.14	1.14
	9.09	5.5	18.3	52.7	28.8	0.64	1.84	1.00
	11.11	6.2	18	52.7	29.2	0.57	1.68	0.93
	14.29	6.9	17.6	52.4	29.8	0.49	1.46	0.83
	20	8.1	17.2	51.7	30.8	0.40	1.21	0.72
	33.33	9.8	16.6	50	33.1	0.28	0.84	0.56
$PdGa_4$@MFI-700RED	1.33	1.6	20.5	56.8	22.7	1.40	3.88	1.56
	1.82	2	19.8	57.6	22.5	1.25	3.62	1.41
	2.22	2.3	19.4	58.3	22.3	1.16	3.49	1.34
	2.86	2.7	19.2	58.5	22.2	1.04	3.19	1.21
	4	3.4	18.8	58.7	22.5	0.91	2.84	1.09
	9.09	6.1	17.7	57.2	25	0.69	2.25	0.98
	11.11	6.8	17.4	56.9	25.7	0.62	2.04	0.92
	14.29	7.8	17.3	56.2	26.4	0.55	1.78	0.84
	20	9.2	17	55.2	27.7	0.46	1.48	0.74
	33.33	11.1	16.6	53.2	30	0.32	1.03	0.58
$PdGa_1/SiO_2$-700RED	1.33	1.4	72.9	0.4	26.7	4.42	0.03	1.62
	1.82	1.8	72.4	0.5	27.1	3.96	0.03	1.48
	2.22	2.0	72.2	0.5	27.3	3.65	0.03	1.38
	2.86	2.3	71.6	0.7	27.8	3.26	0.03	1.26
	4	2.8	70.6	0.8	28.6	2.76	0.03	1.12
	6.67	3.5	67.9	1.1	31.0	2.03	0.03	0.92
	7.69	3.4	69.1	1.2	29.7	1.77	0.03	0.76
	9.09	3.6	69.0	1.3	29.8	1.59	0.03	0.69
	11.11	4.0	68.2	1.4	30.4	1.41	0.03	0.63

	14.29	4.5	64.4	1.5	34.1	1.18	0.03	0.63
	20	4.9	64.2	2.0	33.7	0.91	0.03	0.48
	33.33	6.1	61.5	2.8	35.8	0.65	0.03	0.38

Table S13. Initial selectivity and reaction rate by extrapolating to zero CO_2 conversion at 260 °C and 20 bar with second-order polynomial fits.

**Sample**	**Extrapolating initial selectivity**				**Initial rate ($mmol_{CO2}\cdot g_{cat}^{-1}h^{-1}$)**	**Initial rates for different products ($mmol\cdot g_{cat}^{-1}h^{-1}$)**				**r_3/r_1**
	**MeOH**	**DME**	**CO**	**MeOH+ DME**		**MeOH (r_1)**	**DME (r_3)**	**CO (r_4)**	**MtD (r_2)**	
$PdGa_1$@MFI	58.0	9.5	32.3	67.5	3.89	2.26	0.37	1.26	-	0.16
$PdGa_2$@MFI	30.0	43.0	26.9	73.0	6.44	1.93	2.77	1.74	-	1.44
$PdGa_4$@MFI	21.4	57.1	21.5	78.5	6.83	1.46	3.90	1.47	-	2.67
$PdGa_1/SiO_2$	75.3	0.2	24.5	75.5	6.06	4.56	0.01	1.48	-	0.002

6.5.2. Delplot analysis.

For more detailed analysis of reaction order in DME formation a Delplot- type analysis have been done[62].

Under these conditions, an apparent order extracted from a Delplot-type analysis can help in the identification of pathways, specifically when they coexists. For $PdGa_2$@MFI-700RED and $PdGa_4$@MFI-700RED, the Delplots for DME are consistent with predominantly first-order behavior, indicating that a direct DME-formation route from CO_2 and H_2 dominates for these samples (Figure S75), namely on Ga^+ Lewis acid sites in close proximity to Brønsted acid sites. In contrast, for $PdGa_1$@MFI-700RED, the DME data at low conversion (X_{CO2} < 0.9%) has greater uncertainty and show features compatible with both first- and second-order trends, representative of the coexistence of PdGa alloy sites adjacent to Brønsted acid sites and a low amount of Ga^+ Lewis acid sites. These results can be attributed to dual DME formation path operating simultaneously: (i) A direct DME-formation pathway (r_3, formally first order) on Ga^+ Lewis acid sites + Brønsted acid sites; and (ii) Methanol dehydration on acid sites ($r_1 + r_2$, formally second order) with methanol formed on PdGa alloy sites + Brønsted acid sites.

Finally, the 2nm $PdGa_1/SiO_2$-700RED reference catalyst clearly shows second-order behavior for DME, while due to the absence of Brønsted acid sites, DME formation is extremely low in this sample.

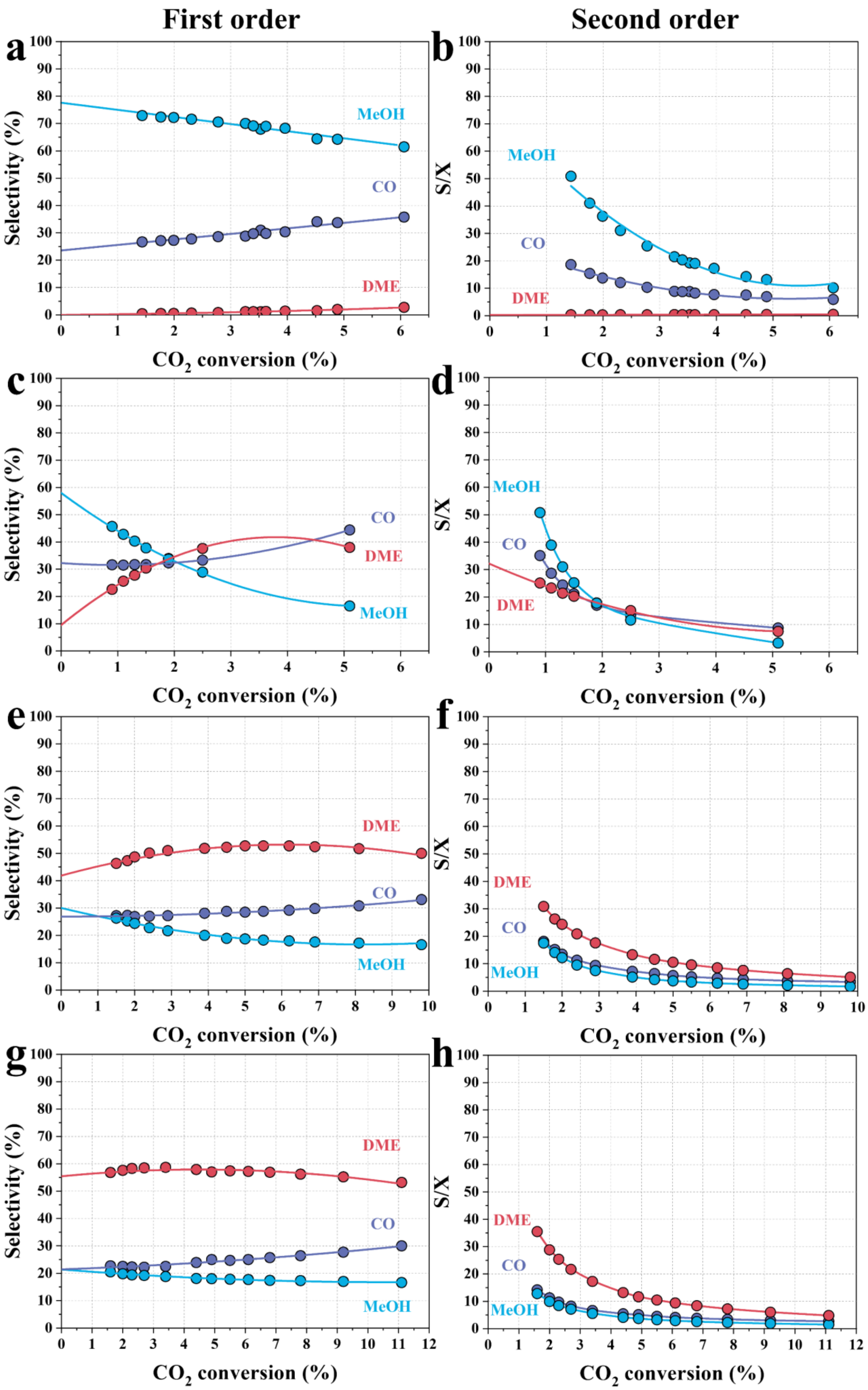

Figure S75. First- and second-order Delplot analyses of CO, methanol (MeOH), and

dimethyl ether (DME) over (a,b) 2nm $PdGa_1/SiO_2$-700RED, (c,d) $PdGa_1$@MFI-700RED, (e,f) $PdGa_2$@MFI-700RED, and (g,h) $PdGa_4$@MFI-700RED at 260 °C and 20 bar. Left panels: first-order Delplot; right panels: second-order Delplot. Symbols are experimental data; curves represent the corresponding fits.

6.5.3. Apparent activation energy for CO formation on $PdGa_x$@MFI catalysts.

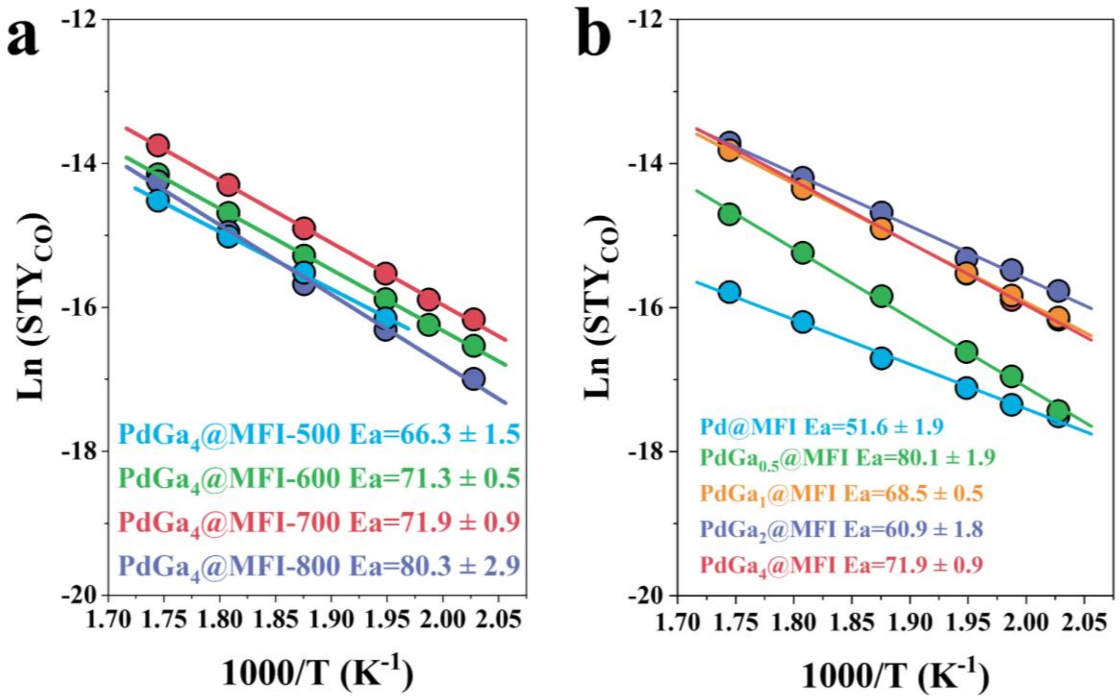

Figure S76. Apparent activation energy for CO formation on (a) $PdGa_4$@MFI with different reduction temperatures, and (b) $PdGa_x$@MFI-700RED with different Ga/Pd ratio.

6.5.4. Reaction order of PdGa@MFI and supported $PdGa_1/SiO_2$ catalysts

The measured rates were fitted by a power law kinetic model following the expression: $r_i = k_i * P_{H2}^{\alpha} * P_{CO2}^{\beta}$, where r_i corresponds to the rate of methanol+DME or CO formation, α and β correspond to the apparent reaction orders with respect to H_2 and CO_2 partial pressures, respectively.

$$r_{MeOH+DME} = k_{MeOH+DME} {H_2}^{\alpha} {CO_2}^{\beta}$$

$$r_{CO} = k_{CO} {H_2}^{\alpha} {CO_2}^{\beta}$$

Note that the apparent order of methanol and DME is very difficult to determine exactly because DME is formed as a primary and secondary product from methanol, therefore, the orders determined here is a global apparent order of the reaction of oxygenated products.

Table S14. Summarize data of reaction order.

Sample	Partial pressure (Bar)			STY ($mmol \cdot g_{cat}^{-1} \cdot h^{-1}$)			
	CO_2	H_2	N_2	MeOH	DME	CO	MeOH+DME
$PdGa_1$@MFI-700RED	4.74	2.37	12.89	0.30	0.19	0.74	0.49
	4.74	4.74	10.52	0.48	0.33	0.83	0.80
	4.74	9.5	5.76	0.84	0.58	0.95	1.43
	4.74	14.26	1	1.22	0.81	1.05	2.03
	1.9	9.5	8.6	1.52	1.11	1.35	2.63
	3.166	9.5	7.334	1.19	0.83	1.14	2.02
	4.74	9.5	5.76	0.94	0.63	0.97	1.57
	9.5	9.5	1	0.61	0.38	0.79	0.99
$PdGa_4$@MFI-700RED	4.74	2.37	12.89	0.38	0.91	1.21	1.29
	4.74	4.74	10.52	0.61	1.63	1.40	2.23
	4.74	9.5	5.76	1.04	2.92	1.58	3.96
	4.74	14.26	1	1.45	4.12	1.74	5.57
	1.9	9.5	8.6	1.85	5.41	2.48	7.26
	3.166	9.5	7.334	1.45	4.17	2.03	5.61
	4.74	9.5	5.76	1.18	3.30	1.72	4.47
	9.5	9.5	1	0.77	2.09	1.29	2.86
$PdGa_1/SiO_2$-700RED	4.74	2.37	12.89	0.90	0.01	1.32	0.91
	4.74	4.74	10.52	1.62	0.02	1.38	1.64
	4.74	9.5	5.76	2.86	0.02	1.51	2.88
	4.74	14.26	1	3.90	0.03	1.58	3.92
	1.9	9.5	8.6	4.52	0.04	2.19	4.56
	3.166	9.5	7.334	3.59	0.03	1.80	3.62
	4.74	9.5	5.76	2.83	0.02	1.49	2.85
	9.5	9.5	1	1.86	0.01	1.08	1.87

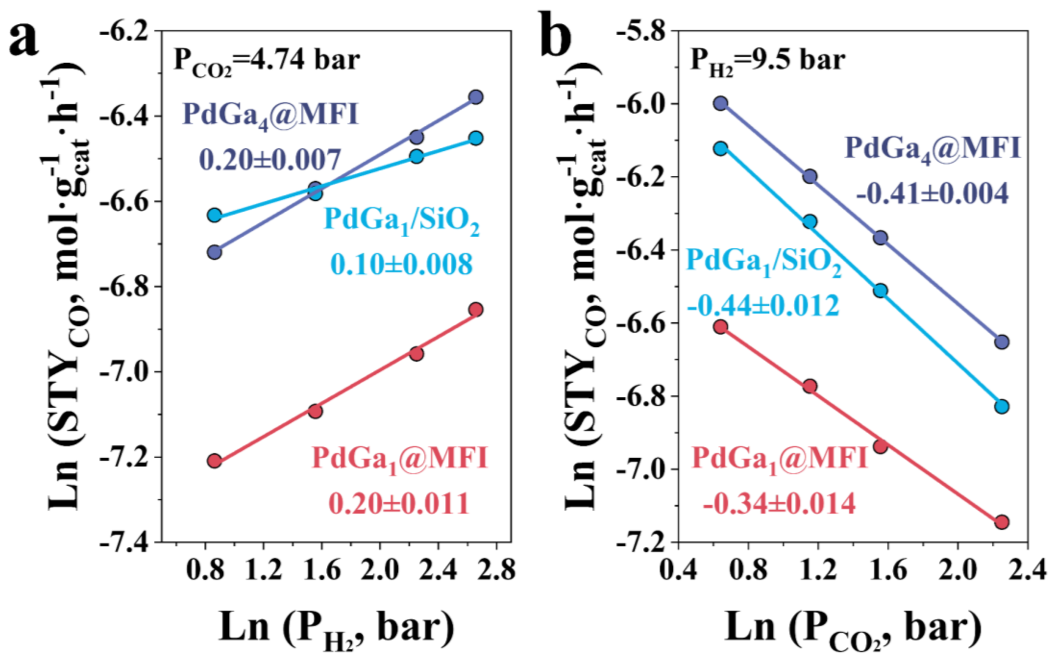

Figure S77. Reaction order of H_2 and CO_2 for CO formation on PdGa@MFI and supported $PdGa_1/SiO_2$.

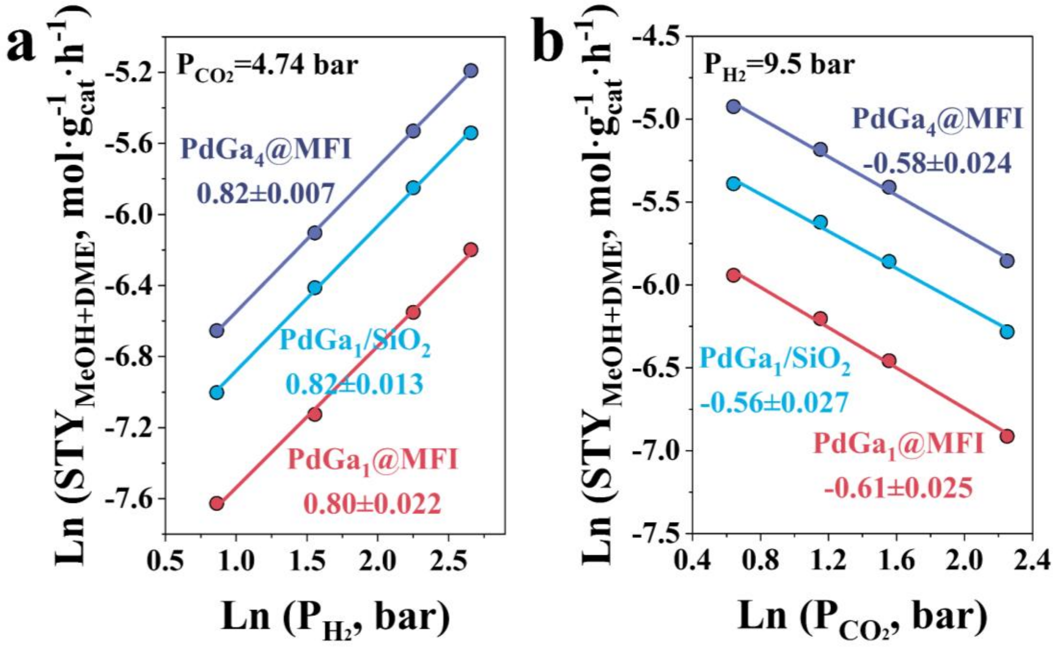

Figure S78. Reaction order of H_2 and CO_2 for DME+MeOH formation on PdGa@MFI and supported $PdGa_1/SiO_2$.

Table S15. Reaction order with respect to reactants CO_2 and H_2.

Sample	Reactant	Product			
		**MeOH**	**DME**	**CO**	**MeOH+DME**
$PdGa_1$@MFI-700RED	CO_2	-0.5670	-0.6686	-0.3362	-0.6079
	H_2	0.7841	0.8154	0.1954	0.7964
$PdGa_4$@MFI-700RED	CO_2	-0.5441	-0.5930	-0.4066	-0.5802
	H_2	0.7460	0.8427	0.1990	0.8159
$PdGa_1/SiO_2$-700RED	CO_2	-0.5583	-0.7563	-0.4416	-0.5598
	H_2	0.8183	0.4941	0.1030	0.8155

Comments:

CO_2 has a negative contribution on the rate of CO, methanol and DME formation as indicated by its negative apparent order, being quite similar in both $PdGa_4$@MFI-700RED and $PdGa_1$@MFI-700RED samples with apparent orders of -0.3, -0.5 and -0.6 respectively. From a kinetic point of view this can be explained as a preferential CO_2 adsorption on the catalyst surface reducing the availability of active H_2. On the other hand, there is a clear positive effect of H_2 partial pressure on the products in all materials, indicating that H_2 is involved in the rate determining step of the reaction, in particular for methanol and DME formation. The apparent reaction order of H_2 respect to CO, methanol and DME are close to 0.2, 0.7 and 0.8 respectively.

7. *In-situ* spectroscopic studies on PdGa$_x$@MFI-700RED samples

7.1. *In-situ* IR.

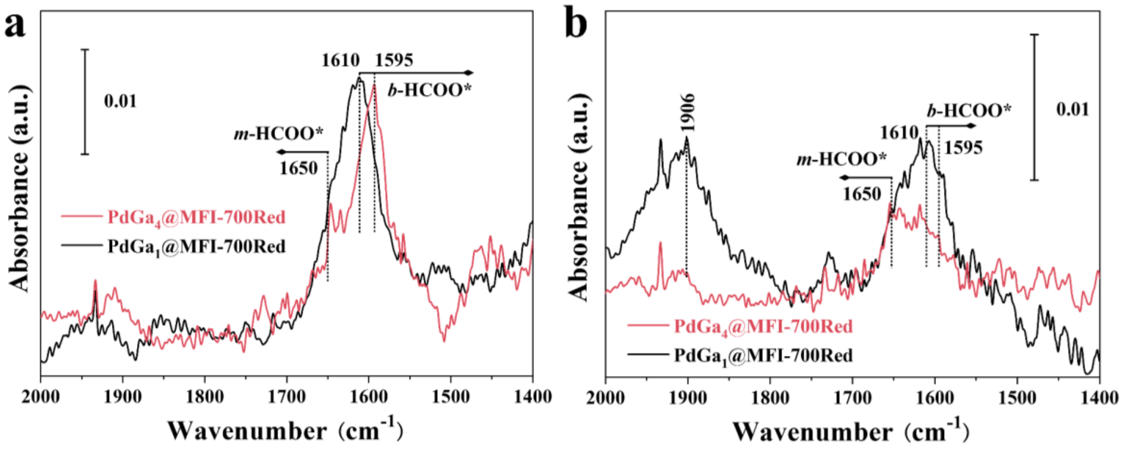

Figure S79. IR spectra in the 2000-1400 cm^{-1} region of PdGa$_1$@MFI-700RED and PdGa$_4$@MFI-700RED catalysts under H_2/CO_2 reaction mixture at (a) 200 °C and (b) 260 °C.

7.1.1. Transient IR studies.

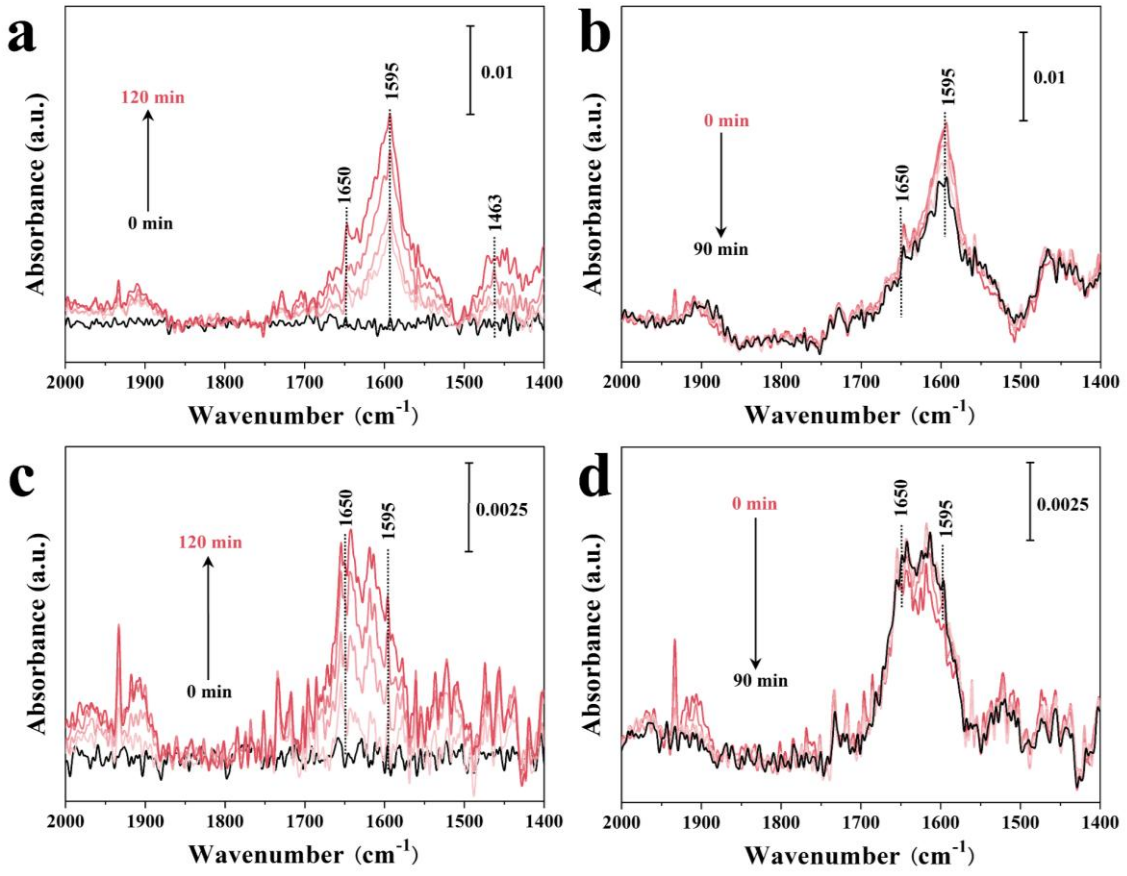

Figure S80. IR spectra in the 2000-1400 cm^{-1} region under different chemical atmospheres over PdGa$_4$@MFI-700RED catalyst at 200 and 260 °C. Time evolution of

spectra at 200 °C in (a) reaction mixture and the subsequent shift to (b) H_2/He. Time evolution of spectra at 260 °C in (c) reaction mixture and the subsequent shift to (d) H_2/He. Reaction conditions: H_2/He = H_2/CO_2 = 3, 10 bar, 20 mL/min.

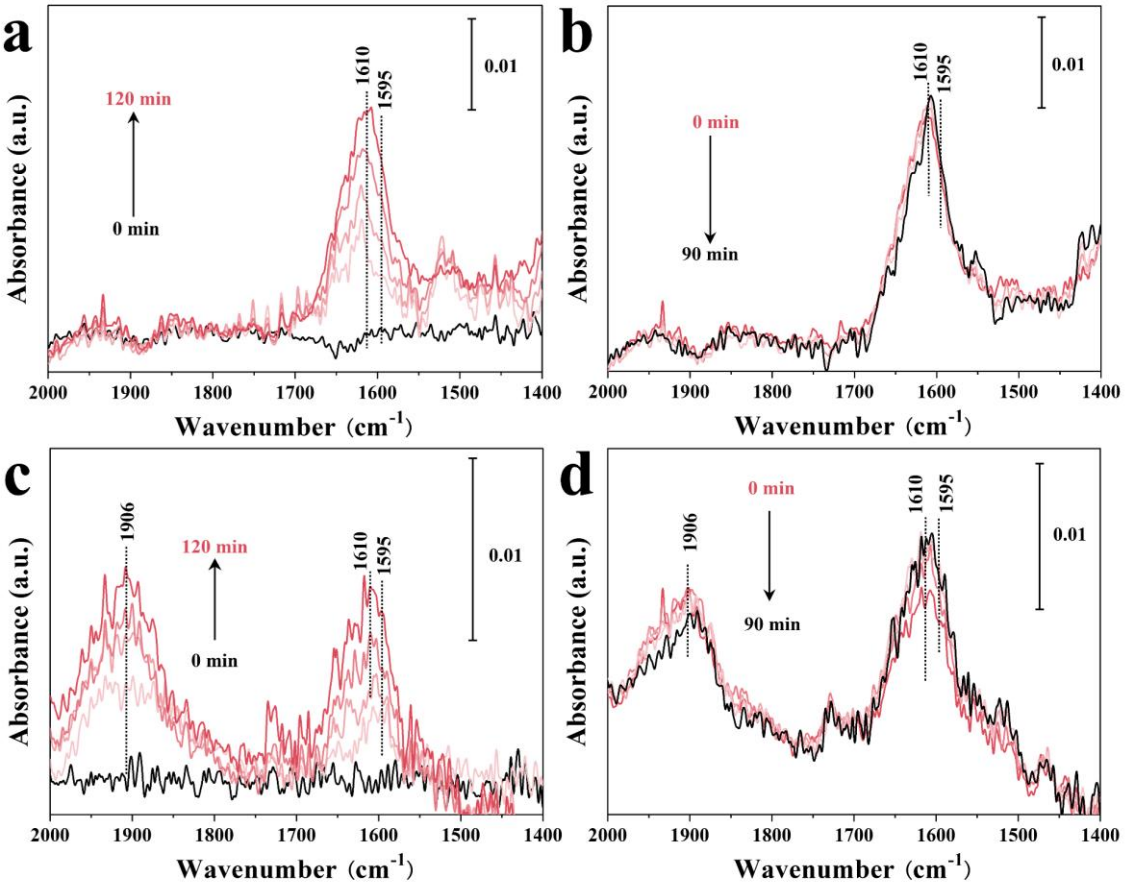

Figure S81. IR spectra in the 2000-1400 cm^{-1} region under different chemical atmospheres over $PdGa_1$@MFI-700RED catalyst at 200 and 260 °C. Time evolution of spectra at 200 °C in (a) reaction mixture and the subsequent shift to (b) H_2/He. Time evolution of spectra at 260 °C in (c) reaction mixture and the subsequent shift to (d) H_2/He. Reaction conditions: H_2/He = H_2/CO_2 = 3, 10 bar, 20 mL/min.

7.1.2. Temperature resolved IR studies

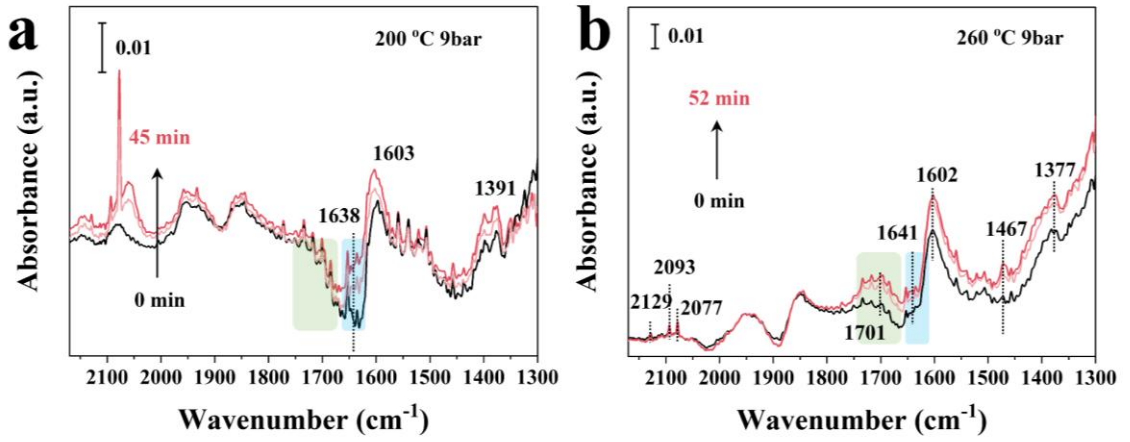

Figure S82. IR spectra of PdGa$_4$@MFI-700RED catalyst under reaction mixture at increasing reaction time at (a) 200 ºC and (b) 260 °C. Reaction conditions: $H_2/CO_2 = 3$, 10 bar, 20 mL/min.

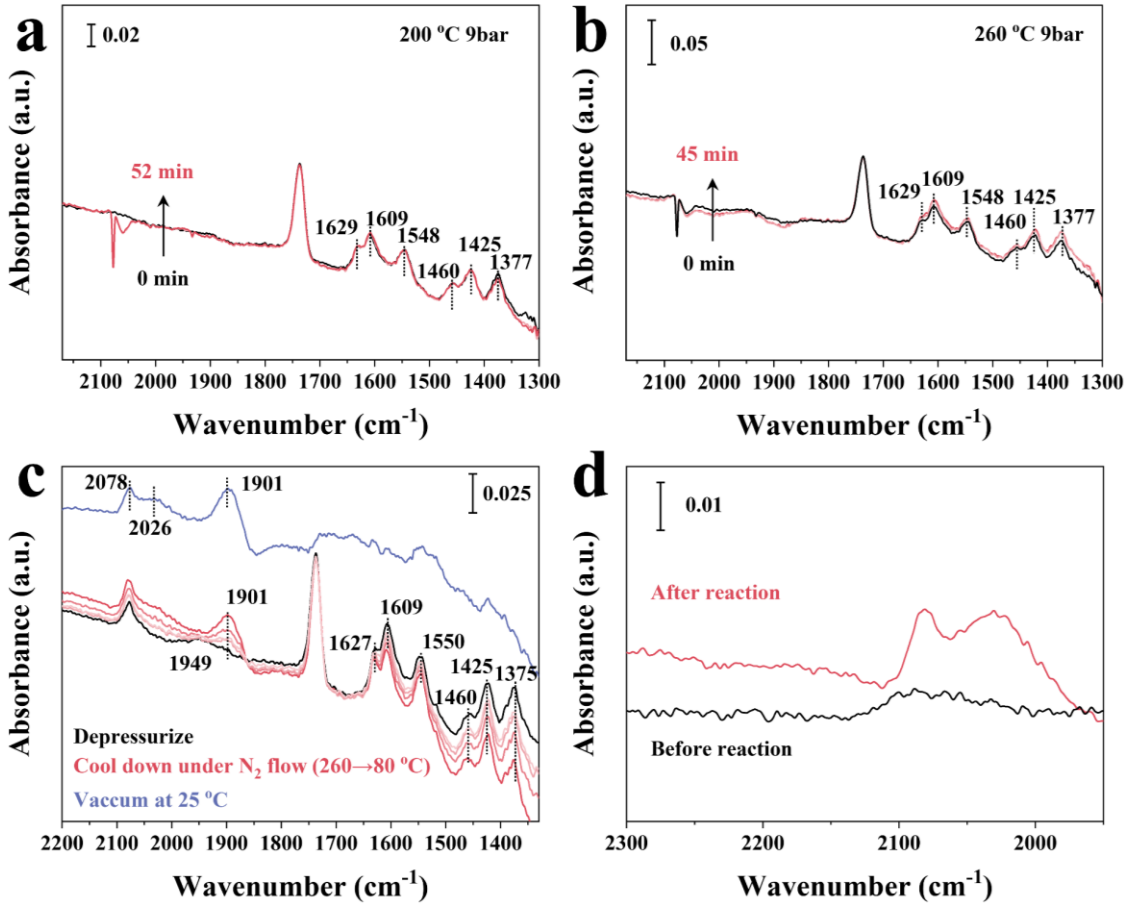

Figure S83. IR spectra of PdGa$_1$@MFI-700RED catalyst under reaction mixture at increasing reaction time at (a) 200 ºC, (b) 260 °C, (c) IR cell depressurization at 260 ºC: in black the first spectra and subsequent spectra under cooling down the temperature in N_2 flow in red. After vacuum at 25 ºC in purple (d) IR spectra of the sample before and

after reaction. Reaction conditions: H_2/CO_2 = 3, 10 bar, 20 mL/min. CO is clearly observed and formed during the reaction

7.1.3. IR studies of the adsorption of formic acid for discrimination of formate species adsorbed on Pd, PdGa alloy, Ga^{3+} and Ga^{+}.

Formic acid has been adsorbed on the following samples: Ga_4@MFI-CAL (containing predominately Ga^{3+}, with some Ga^{+}), Ga_4@MFI-RED after 400 ºC H_2 reduction (containing a higher proportion of Ga^{+} and a lower amount of Ga^{3+} compared to the calcined sample, based on XAS, Figure S94), Pd@MFI-700RED (containing Pd NP), PdG_4@MFI-700RED (Containing PdGa alloy and Ga^{+}, predominately).

The IR spectra are included in Figure S84-S88.

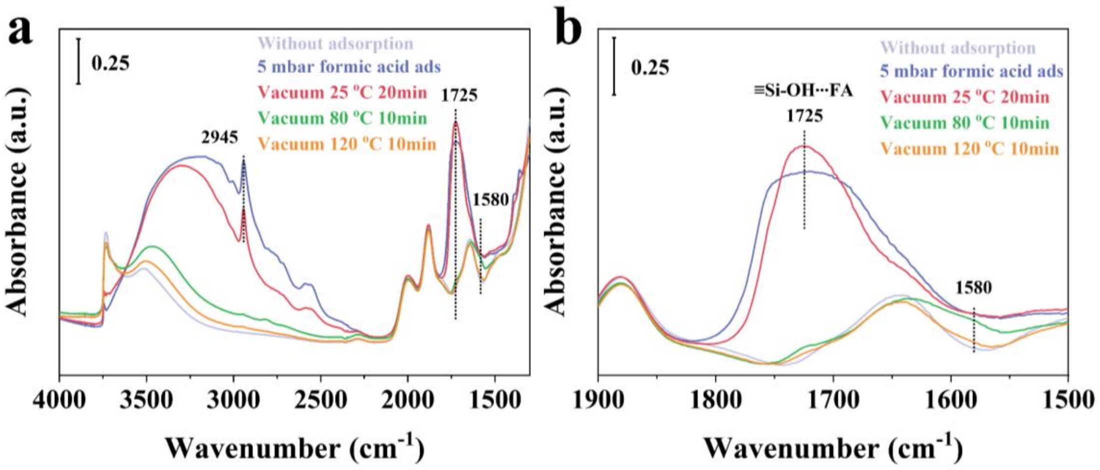

Figure S84. IR spectra of formic acid on the Pd@MFI-700RED sample. IR spectra have been collected after the adsorption of 5 mbar formic acid (purple line), followed by evacuation at increasing temperature: 25 ºC (red line), 80 ºC (green line) and 120 ºC (yellow line). The IR spectra of the sample before adsorption is displayed in grey.

In Figure S84, the IR band at 1725 cm^{-1} corresponds to the carbonyl group of formic acid interacting with hydroxyl groups (OH) of the zeolite. This is supported by the corresponding shift in the OH region and its fast desorption at increasing temperatures. On the other hand, the IR band at 1580 cm^{-1}, observed at 80 ºC, can be ascribed to the ν_{asym}(OCO) of bidentate formiate species (Ref [14,63,64]), interacting with the Pd NP.

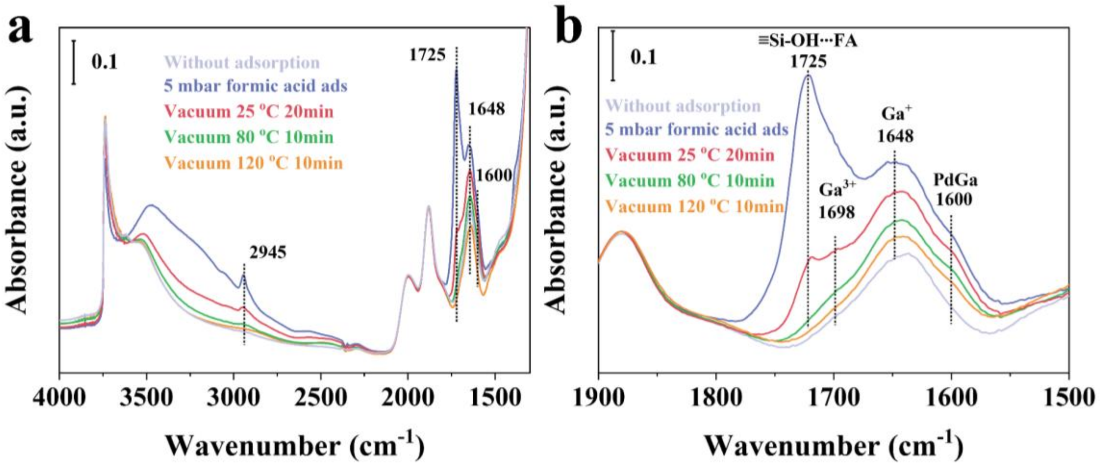

Figure S85. IR spectra of formic acid on the $PdGa_4$@MFI-700RED sample. IR spectra have been collected after the adsorption of 5 mbar formic acid (purple line), followed by evacuation at increasing temperature: 25 ºC (red line), 80 ºC (green line) and 120 ºC (yellow line). The IR spectra of the sample before adsorption is displayed in grey.

As before the IR band at 1720 cm^{-1} is due to a weak interaction of formic acid with OH groups, and the IR band at 1600 cm^{-1} can be ascribed to the ν_{asym}(OCO) of bidentate formate species. The slight higher frequency of the last band compared to that of the Pd@MFI sample could be related to Pd species in a different local environments, as is the case of the PdGa alloy. On the other hand the IR bands at 1648 cm^{-1} can be ascribed to the ν_{asym}(OCO) of monoformate species, which usually appears around 1650-1630 cm^{-1} (Ref [14,63,64]). Notice also a small shoulder at 1698 cm^{-1}. In order to distinguish between monoformate species interacting with Ga^{3+} or Ga^{+} sites, Ga_4@MFI sample has been used as reference sample, where the concentration of Ga^{3+} and Ga^{+} species have been altered based on XAS analysis. MCR analysis of the Ga_4@MFI-XAS spectra (Figure S94), shows an increase in the amount of Ga^{+} species at the expense of Ga^{3+} when the sample is reduced in H_2 flow at increasing temperature. Consequently, to figure out the contribution of Ga^{+} and Ga^{3+} species in the stabilization of monoformate species, a comparative IR analysis have been done on the Ga_4@MFI-CAL sample and the same sample *in-situ* reduced at the maxima available temperature (i.e., 400 ºC) of the IR cell. The corresponding IR spectra are included in Figure S86-S87. The IR spectra of formic acid on pure MFI zeolite is given in Figure S88.

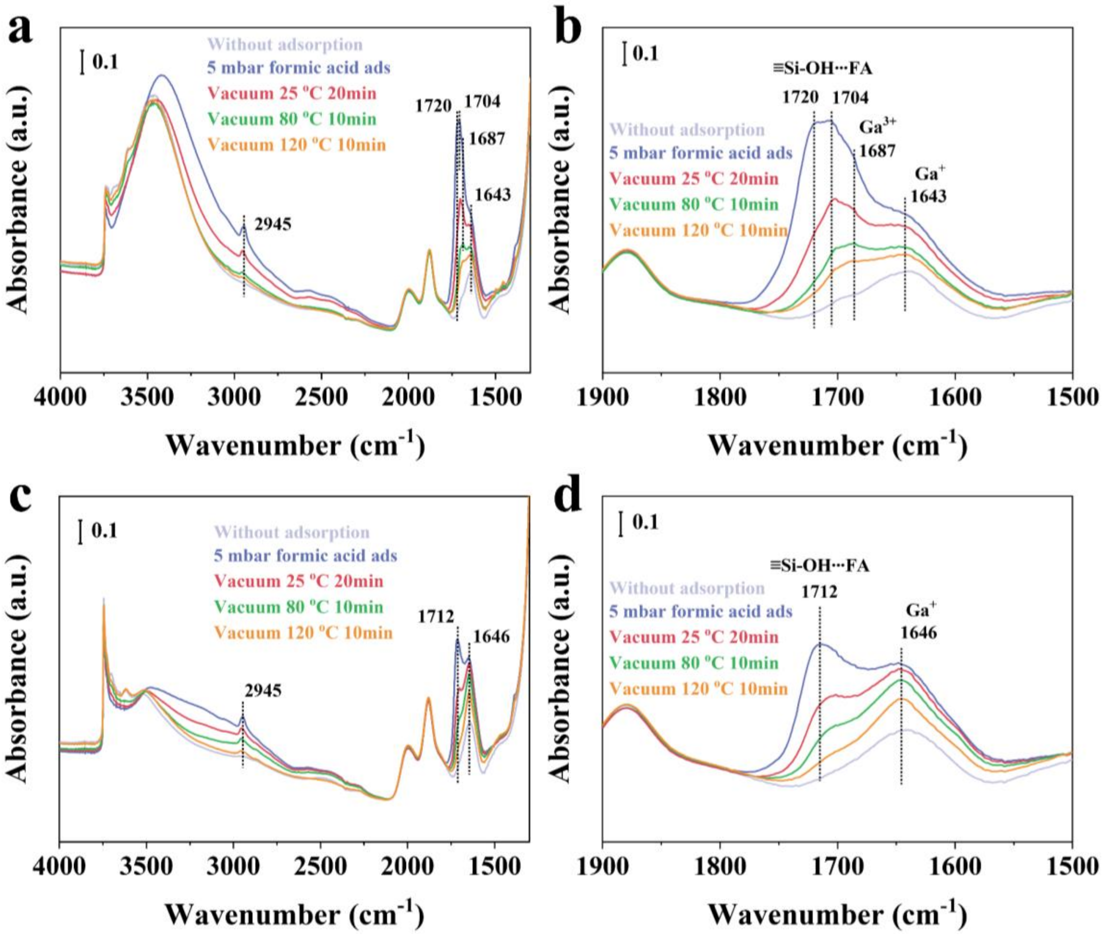

Figure S86. IR spectra of formic acid on the (a, b) Ga_4@MFI-CAL and (c, d) Ga_4@MFI-RED samples. The spectra have been collected, prior to adsorption, after the adsorption of 5 mbar formic acid (purple line), and under evacuation at increasing temperature: 25 ºC (red line), 80 ºC (green line) and 120 ºC (yellow line).

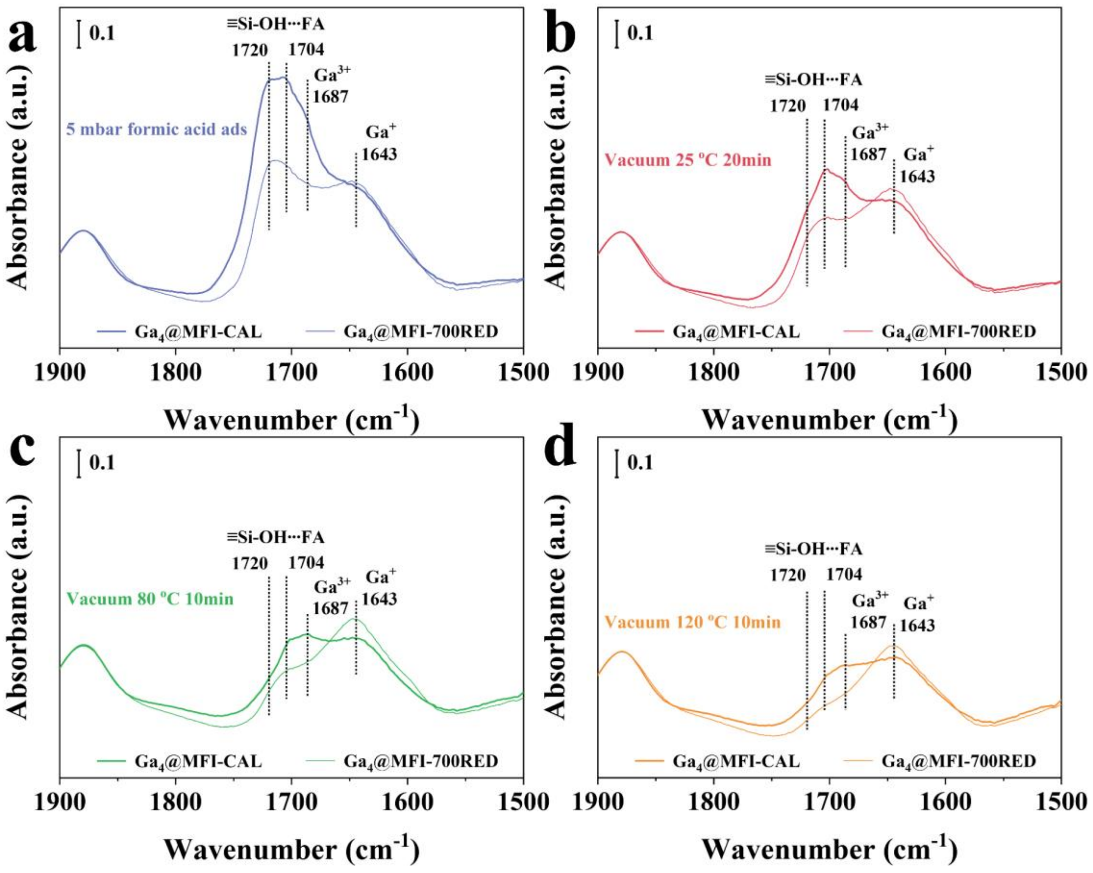

Figure S87. Comparative IR spectra on the Ga_4@MFI-CAL (thick line) and Ga_4@MFI-RED (thin line) samples under similar conditions as in Figure S86.

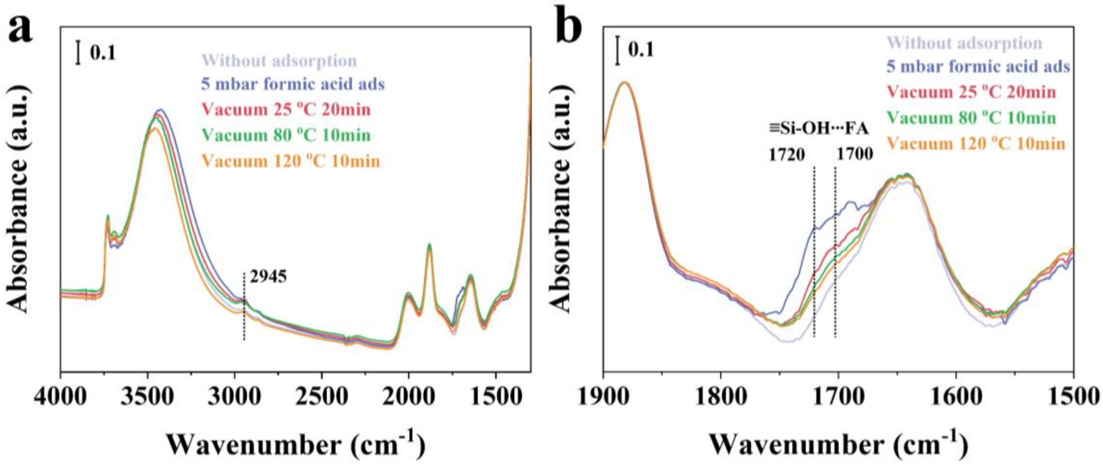

Figure S88. IR spectra of formic acid on the pure MFI silica sample. IR spectra have been collected after the adsorption of 5 mbar formic acid (purple line), followed by evacuation at increasing temperature: 25 ºC (red line), 80 ºC (green line) and 120 ºC (yellow line). The IR spectra of the sample before adsorption is displayed in grey.

<u>**Comments**</u>:

From Figure S88 it is seen that both IR bands at 1720 and 1700 cm^{-1} are due to the C=O

bond of weakly interacting formic acid on pure zeolite.

From the comparative study done in Figure S87, it is shown that the intensity of the IR bands at 1704 and 1687 cm^{-1} decreases on the Ga_4@MFI-RED sample, in parallel to an increase of the intensity of the IR band at 1643 cm^{-1}. The IR band at 1704 cm^{-1} can be associated, as mentioned above, to weakly interacting formic acid with the zeolite, while both 1687 and 1643 cm^{-1} IR bands should be related to Ga species. Supported on XAS data, we can assume that the first band at higher frequencies are associated to monoformate species stabilized on Ga^{3+} species, which decrease after *in-situ* reduction of the sample, whereas the IR band at 1643 cm^{-1} can be ascribed to monoformate species stabilized on Ga^{+}, increasing after *in-situ* reduction.

Based on these results and the operando IR studies shown in Figure 7 and Figure S79-83, we can affirm that Ga^{+} species participate in the reaction, instead of Ga^{3+}, and play a direct role in the production of DME.

7.2. *In-situ* XAS studies

The Ga K-edge XANES spectra were subjected to Multivariate Curve Resolution-Alternating Least Squares (MCR-ALS) unmixing, using the Multivariate Curve Resolution in Python pyMCR toolbox running under Python 3.7.4[65-68]. In detail, the initial estimate for the spectra of the pure components have been done considering the spectral variability and expectations, and then the ALS algorithm was operated using as constraints non-negativity for spectra and concentration profiles, together with the normalization constraint, which ensures that after each iteration the summation of all concentrations for each data point sums-to-one. Five components were found to be the best model complexity to deconvolute the different *in-situ* data sets obtained on $PdGa_{0.5}$@MFI-700RED, $PdGa_4$@MFI-700RED, Ga_4@MFI-700RED, and IM-Ga_4/Pd@ZSM-5-700RED systems.

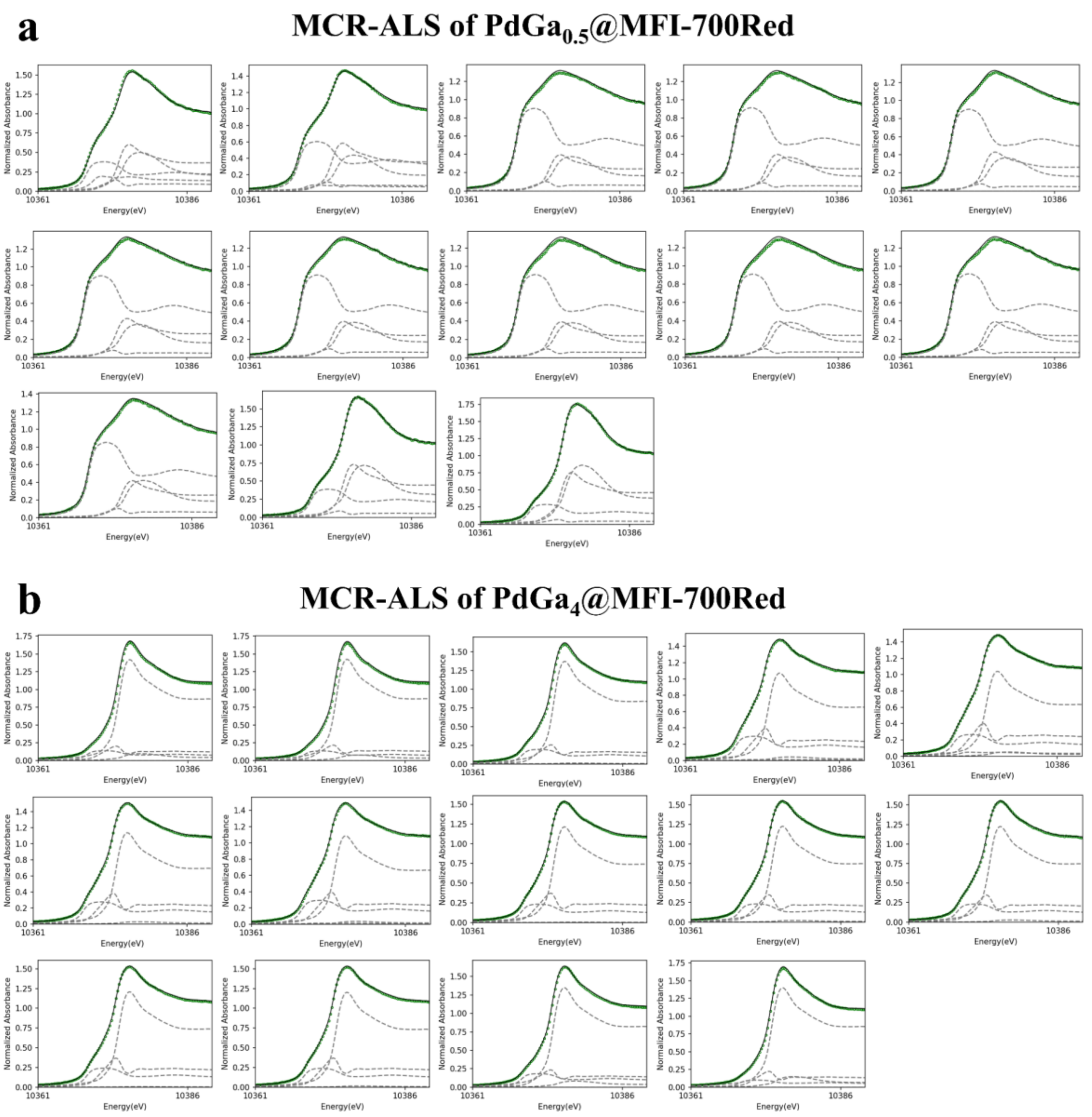

Figure S89. Ga K-edge spectra deconvolution with the identified MCR-ALS components for the (a) $PdGa_{0.5}$@MFI-700RED and (b) $PdGa_4$@MFI-700RED systems with every 5 spectra showing here. The green symbols represent the experimental data, the dashed curves the MCR-ALS components, while the continuous black curve the reconstructed spectra considering the MCR-ALS components weight reported on Figure 2 of the main manuscripts.

<u>Comments</u>:

The Pd K-edge EXAFS spectra acquired on the $PdGa_4$@MFI-700RED catalyst where fitted accordingly to the standard equation in single scattering approximation. Multi (k^1, k^2, k^3)-weighted fits carried out in r-space (1-3 Å) over a k-range of 3-12 $Å^{-1}$ using a Hannings window (dk 1), and $S_0^2 = 0.9$. Bond distances and disorder parameters (Δr_{eff} and σ^2) were allowed to float having initial values of 0.0 Å and 0.003 $Å^2$ respectively, with a universal E_0 and $\Delta E_0 = 0$ eV.

The Pd K-edge EXAFS collected before reaction is reminiscent of Pd^0, but the FT of the EXAFS region shows the presence of both Pd-Ga and Pd-Pd scattering paths at 2.55 Å and 2.75 Å, respectively. The fact that the Pd-Ga formation is not detectable by Ga K-edge EXAFS is due to its relatively small amount once compared to the total Ga (< 10%), and its highly disordered nature. Instead, it is detectable by MCR over the Ga K-edge XANES region while analysing all the data sets.

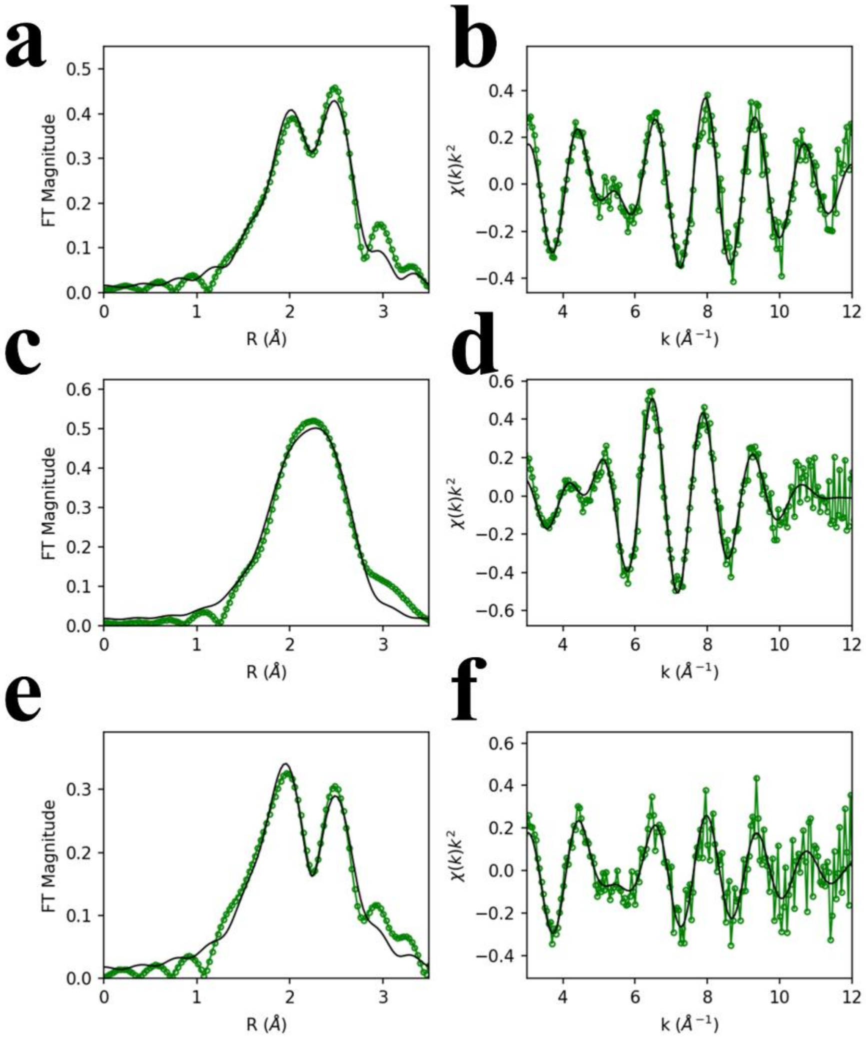

Figure S90. Pd K-edge k^2 weighted EXAFS oscillation and corresponding Fourier transform obtained for the $PdGa_4$@MFI-700-RED sample: fresh (a, b), after reduction and then reaction (c, d), and after exposure to air (e, f).

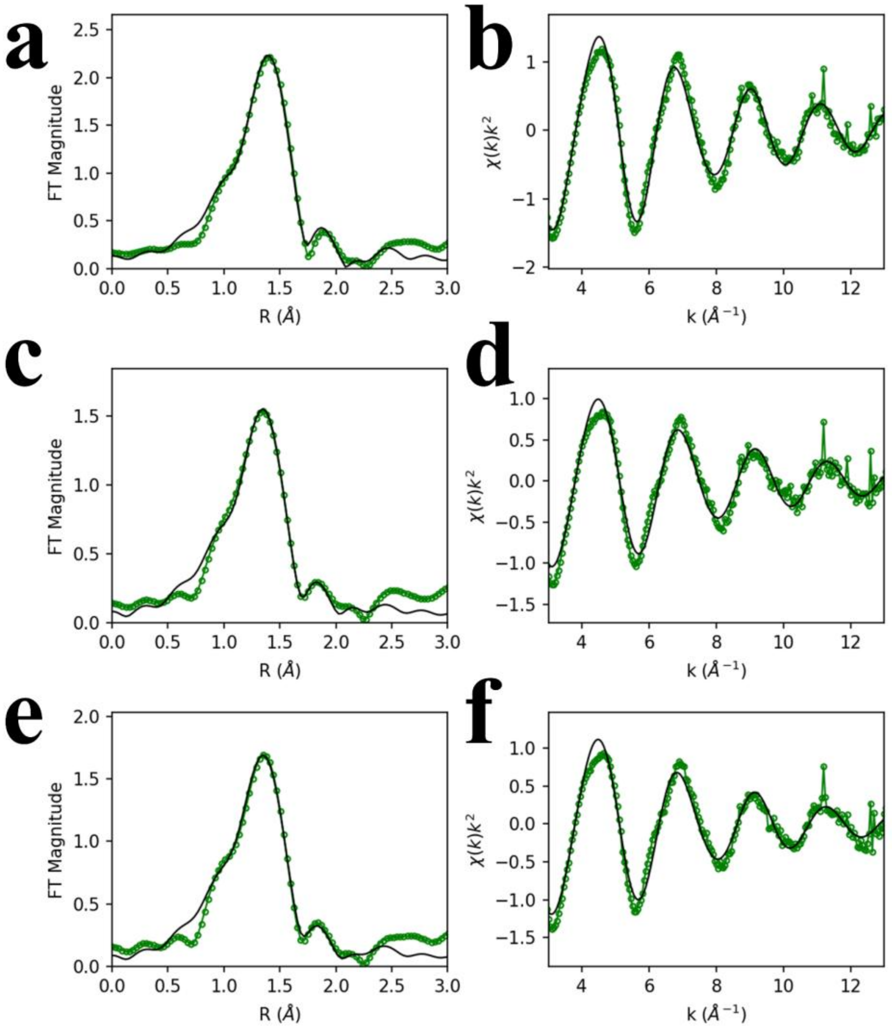

Figure S91. Ga K-edge k^2 weighted EXAFS oscillation and corresponding Fourier transform obtained for the Ga_4@MFI-700-RED sample: fresh (a, b), after reduction (c, d), and after exposure to air (e, f).

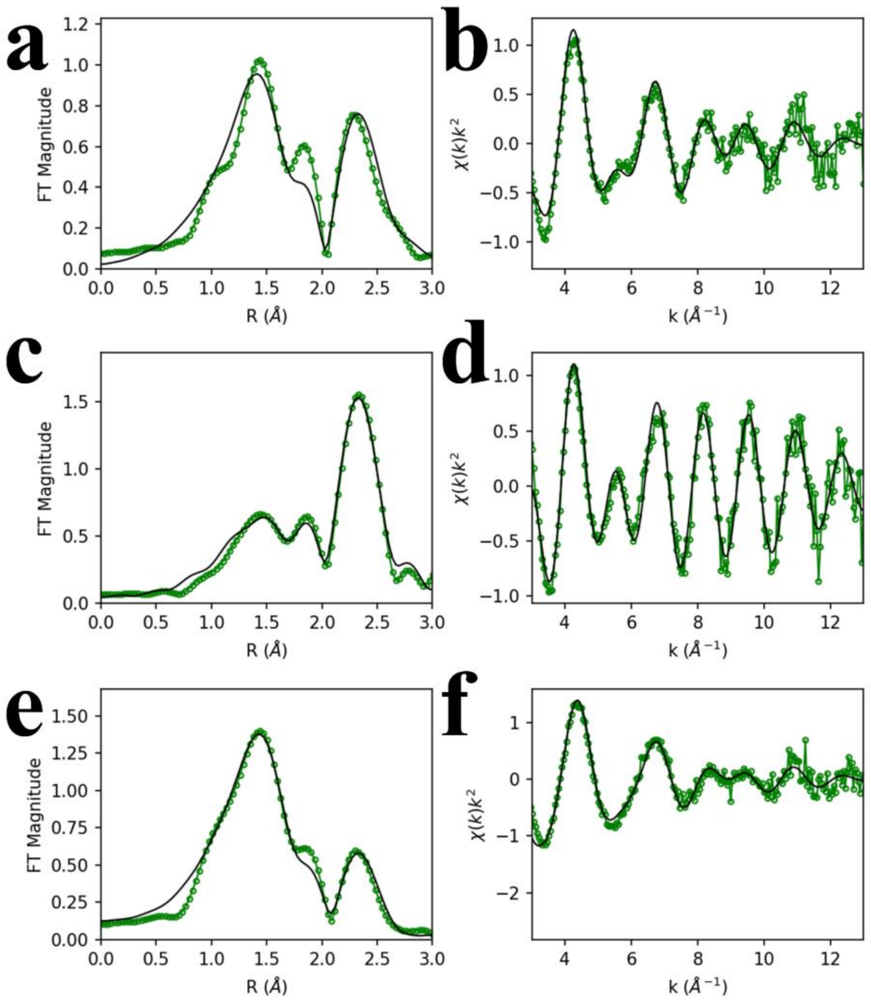

Figure S92. Ga K-edge k^2 weighted EXAFS oscillation and corresponding Fourier transform obtained for the $PdGa_{0.5}$@MFI-700-RED sample: fresh (a, b), after reduction and then reaction (c, d), and after exposure to air (e, f).

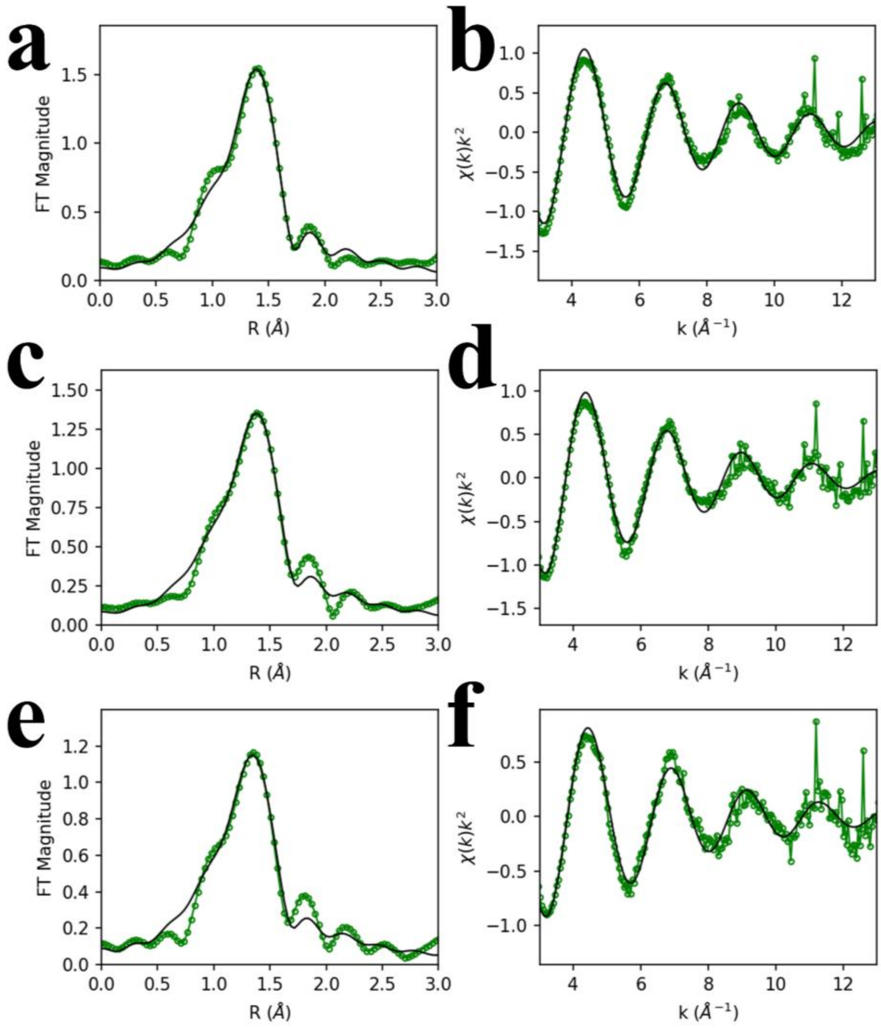

Figure S93. Ga K-edge k^2 weighted EXAFS oscillation and corresponding Fourier transform obtained for the $PdGa_4$@MFI-700-RED sample: fresh (a, b), after reduction and then reaction (c, d), and after exposure to air (e, f).

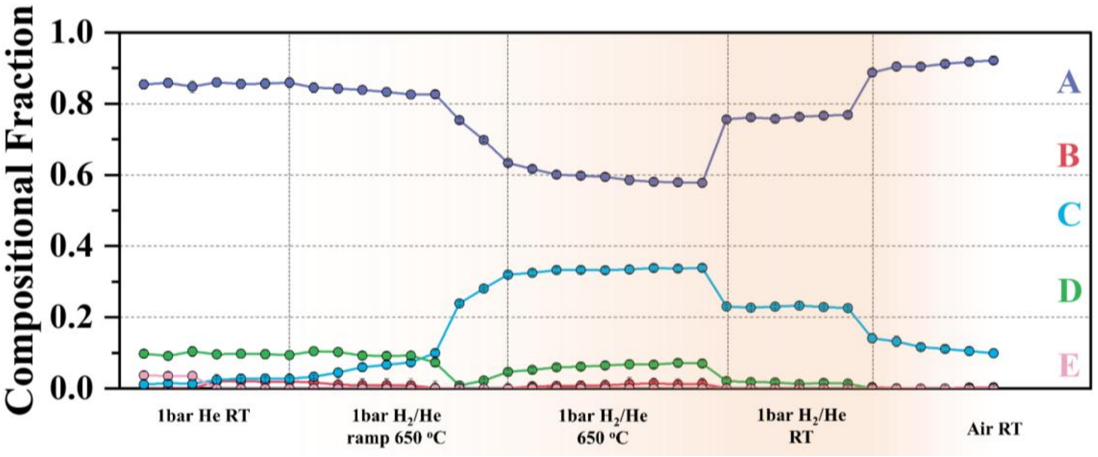

Figure 94. XAS-MCR components for the Ga@MFI-RED sample under H_2 flow at increasing temperatures. Component code: A = tetrahedral Ga^{3+}, B = PdGa alloy, C = isolated Ga^{+}, D = octahedral Ga(III) oxide phase, and E = a reduced Ga species, compatible with a disordered Ga-OPd/Ga cluster.

Table S16. Pd K-edge EXAFS fitting results obtained for the $PdGa_4$@MFI-700RED sample: fresh, after reduction and then reaction, and after exposure to air. Multi (k^1, k^2, k^3)-weighted fits carried out in r-space (1-3 Å) over a k-range of 3-12 $Å^{-1}$ using a Hannings window (dk 1), and S_0^2 = 0.9. Bond distances and disorder parameters (Δr_{eff} and σ^2) were allowed to float having initial values of 0.0 Å and 0.003 $Å^2$ respectively, with a universal E_0 and ΔE_0 = 0 eV. Data was acquired at room temperature.

Sample	R_{FACTOR}	X^2_v	Var. No.	k-range ($Å^{-1}$)	r-range (Å)	ΔE_0 (eV)	Pd-Ga			Pd-Pd		
							CN	r (Å)	σ^2 (x10^3 $Å^2$)	CN	r (Å)	σ^2 (x10^3 $Å^2$)
**$PdGa_4$@MFI-700RED-Fresh**	0.020	5	4	3-12	1-3	-3.6 (0.9)	2	2.55 (0.01)	11.0 (0.4)	3	2.72 (0.01)	11.0 (0.4)
**$PdGa_4$@MFI-700RED-Reaction**	0.016	4	4	3-12	1-3	-4.5 (1.6)	3	2.52(0.01)	11.0 (0.9)	2.5	2.79 (0.01)	11.0 (0.9)
**$PdGa_4$@MFI-700RED-Air**	0.019	1	4	3-12	1-3	-5.2 (0.8)	2.4	2.55 (0.01)	14.1 (0.5)	3.2	2.69 (0.01)	14.1 (0.5)

Table S17. Ga K-edge EXAFS fitting results obtained for the Ga_4@MFI-700RED, $PdGa_{0.5}$@MFI-700RED and $PdGa_4$@MFI-700RED samples: fresh, after reduction and then reaction, and after exposure to air. Multi (k^1, k^2, k^3)-weighted fits carried out in r-space (1-3 Å) over a k-range of 3-13 $Å^{-1}$ using a Hannings window (dk 1), and S_0^2 = 0.9. Bond distances and disorder parameters (Δr_{eff} and σ^2) were allowed to float having initial values of 0.0 Å and 0.003 $Å^2$ respectively, with a universal E_0 and ΔE_0 = 0 eV. Data was acquired at room temperature.

Sample	R_{FACTOR}	X^2_v	Var. No.	k-range ($Å^{-1}$)	r-range (Å)	ΔE_0 (eV)	Ga-O			Ga-Si			Pd-Ga		
							CN	r (Å)	σ^2 (x10^3 $Å^2$)	CN	r (Å)	σ^2 (x10^3 $Å^2$)	CN	r (Å)	σ^2 (x10^3 $Å^2$)
**Ga_4@MFI-700RED Fresh**	0.012	41	5	3-13	1-3	0.7 (1.2)	4	1.80 (0.01)	2.4 (0.4)	2	2.67 (0.02)	14.2 (4)	-	-	-
**Ga_4@MFI-700RED Reduction**	0.018	29	5	3-13	1-3	-3.0 (1.8)	3	1.77 (0.01)	3.0 (0.6)	1	2.64 (0.04)	15.7 (8)	-	-	-
**Ga_4@MFI-700RED Air**	0.016	43	5	3-13	1-3	-2.8 (1.6)	3.5	1.77 (0.01)	3.6 (0.6)	1	2.63 (0.04)	11.5 (6)	-	-	-
**$PdGa_{0.5}$@MFI-700RED-Fresh**	0.023	11	5	3-13	1-3	-2.8 (1.3)	2.5	1.83 (0.01)	7.0 (1.2)	-	-	-	3	2.54 (0.01)	11.1 (1.1)

**$PdGa_{0.5}$@MFI-700RED-Reaction**	0.012	7	5	3-13	1-3	-2.9 (0.6)	1.5	1.81 (0.01)	8.7 (1.8)	-	-	-	4	2.53 (0.01)	7.2 (0.3)
**$PdGa_{0.5}$@MFI-700RED-Air**	0.009	7	5	3-13	1-3	3.8 (1.14)	4	1.85 (0.01)	8.3 (0.1)	-	-	-	1.5	2.56 (0.01)	8.9 (1.2)
**$PdGa_4$@MFI-700RED-Fresh**	0.007	8	3	3-13	1-3	0.5 (0.8)	3	1.81 (0.01)	3.1 (0.4)	-	-	-	-	-	-
**$PdGa_4$@MFI-700RED-Reaction**	0.008	7	3	3-13	1-3	-0.1 (0.9)	3	1.81 (0.01)	4.5 (0.1)	-	-	-	-	-	-
**$PdGa_4$@MFI-700RED-Air**	0.005	24	5	3-13	1-3	2.2 (1.1)	3	1.83 (0.01)	3.5 (0.5)	1	2.69 (0.05)	11.2 (8.5)	-	-	-

Comments: The Pd-Ga coordination number extracted from the EXAFS fits agrees in the error bar with the optically characterized particle size, which do not differ much in between samples.

To estimate the particle size from the EXAFS obtained coordination number, several methods have been applied, all providing similar results. In particular, we exploited the theoretical formula proposed by Calvin et al., directly linking the measured coordination number with the particle size, assuming a spherical particle shape[69].

$$N_{exafs} = \left[1 - \frac{3}{4}\left(\frac{r_{eff}}{R_{nano}}\right) + \frac{1}{16}\left(\frac{r_{eff}}{R_{nano}}\right)^3\right] N_{bulk} \quad (11)$$

Formula 11: Relationship between coordination number and particle radius from Calvin et. al. (where N_{bluk} is the coordination in the bulk, N_{exafs} is the EXAFS determined coordination number, R_{nano} is the particle radius, and r_{eff} is the scattering radius.

This approach has been found to well describe the particles size below few nanometers, which looks to better apply to the present case. By this approach we can estimate the PdGa particle size around 1 nm for $PdGa_{0.5}$@MFI-700RED and 0.5 nm for $PdGa_4$@MFI-700RED, assuming Ga in a Pd matrix. Such calculations are expected to slightly under estimate the particle size. Despite the relatively high error bare due to the performed assumptions, the results suggest that higher Pd/Ga molar ratio corresponds to larger PdGa particles size.

8. $PdGa_x/SiO_2$ supported samples

To confirm the key role of zeolite confinement, a PdGa alloy supported on SiO_2 of similar particle sized (i.e., 2nm) is prepared. According to Raydel et al.[37] larger particle size in $PdGa/SiO_2$ catalysts are active for CO_2 hydrogenation to methanol. Therefore, for comparison, a 15nm PdGa supported on mesoporous SiO_2 is also prepared.

8.1. Synthesis.

The synthesis of 2 nm PdGa/Aerosil 200 is achieved through an amino-protective strategy combined with the incipient wetness impregnation method. Initially, $-NH_2$ groups are anchored onto amorphous SiO_2 following the procedure described by Luo et al.[70] Specifically, 2 g of SiO_2 is dried under vacuum at 120 °C for 2 h. Then, 100 mL of toluene is added under continuous stirring in a nitrogen atmosphere. Subsequently, 4 mL of (3-aminopropyl)triethoxysilane (APTES) is introduced dropwise, and the mixture is stirred for 24 h under N_2 protection. The resulting NH_2-SiO_2 solid is obtained by filtration, washed with ethanol, and dried under vacuum overnight at 120 °C.

For Pd loading onto NH_2-SiO_2, 0.02 g of $PdCl_2$ is dissolved in a mixture of 20 mL H_2O and 4 mL ethanol, followed by the addition of 240 µL of 25 wt% $NH_3·H_2O$. The solution is stirred in an ultrasonic bath for 1 h to ensure complete dissolution. Next, 1 g of NH_2-SiO_2 is added and dispersed ultrasonically for 30 min, followed by stirring at 80 °C under reflux for 4 h. The resulting solid, designated as Pd-NH_2-SiO_2, serves as a precursor for further Ga incorporation. Ga is introduced using the incipient wetness impregnation method at Ga/Pd molar ratios of 1 and 4. Specifically, 0.019 g or 0.076 g of $Ga(NO_3)_3·xH_2O$ is dissolved in 1 g of H_2O and then added dropwise to 1 g of Pd-NH_2-SiO_2 under stirring. The mixture is dried under vacuum at 120 °C overnight and subsequently reduced at 700 °C for 4 h, yielding the final catalysts: 2 nm $PdGa_1/SiO_2$-700RED and 2 nm $PdGa_4/SiO_2$-700RED.

The synthesis of 15 nm $PdGa/SiO_2$ follows a two-step incipient wetness impregnation method. Initially, 0.0148 g of $Pd(NH_3)_4Cl_2·H_2O$ is dissolved in 1.54 g of H_2O and added dropwise to 1 g of High-Surface-Area mesoporous SiO_2 under stirring. The obtained solid is dried under vacuum at 120 °C overnight, then calcined in a tubular oven under an air flow of 75 mL/min, with a heating rate of 1.5 °C/min to 350 °C, and held at this temperature for 2 h, forming Pd/SiO_2. For Ga impregnation, 0.019 g or 0.076 g of $Ga(NO_3)_3·xH_2O$ (corresponding to Ga/Pd molar ratios of 1 and 4, respectively) is

dissolved in 1.54 g of H_2O and added dropwise to 1 g of above Pd/SiO_2 under stirring. The resulting solid is dried under vacuum at 120 °C overnight and then calcined in a tubular oven under air flow (75 mL/min) with a heating rate of 1.5 °C/min to 350 °C, followed by a 2 h hold. Finally, the samples are reduced at 700 °C for 4 h to obtain the catalysts: 15 nm $PdGa_1/SiO_2$-700RED and 15 nm $PdGa_4/SiO_2$-700RED.

8.2. Physico-chemical characterization

8.2.1 Physical-chemical property of studied catalysts

Table S18. Physical-chemical property of supported $PdGa_x/SiO_2$ samples.

Sample	Surface area (m^2/g)	V_{micro} (cm^3/g)	C[a]	N[a]	H[a]	Si/Ga[b]	Ga/Pd[b]	ICP Pd (wt%)[c]	ICP Ga (wt%)[c]
2nm $PdGa_1/SiO_2$-700RED	184.7	0.019	0.59	0.07	0.27	296	1	0.65	0.46
2nm $PdGa_4/SiO_2$-700RED	186.5	0.012	0.44	0	0.19	74	4	0.62	1.61
15 nm $PdGa_1/SiO_2$-700RED	224.0	-	0.21	0	0.18	296	1	0.58	0.42
15 nm $PdGa_4/SiO_2$-700RED	225.7	-	0.14	0	0.21	74	4	0.52	1.57

[a] C, N and H is analysed by elemental analysis;
[b] Theoretical Si/Ga and Ga/Pd ratio;
[c] Actual Pd and Ga weight loading by ICP.

8.2.2. STEM

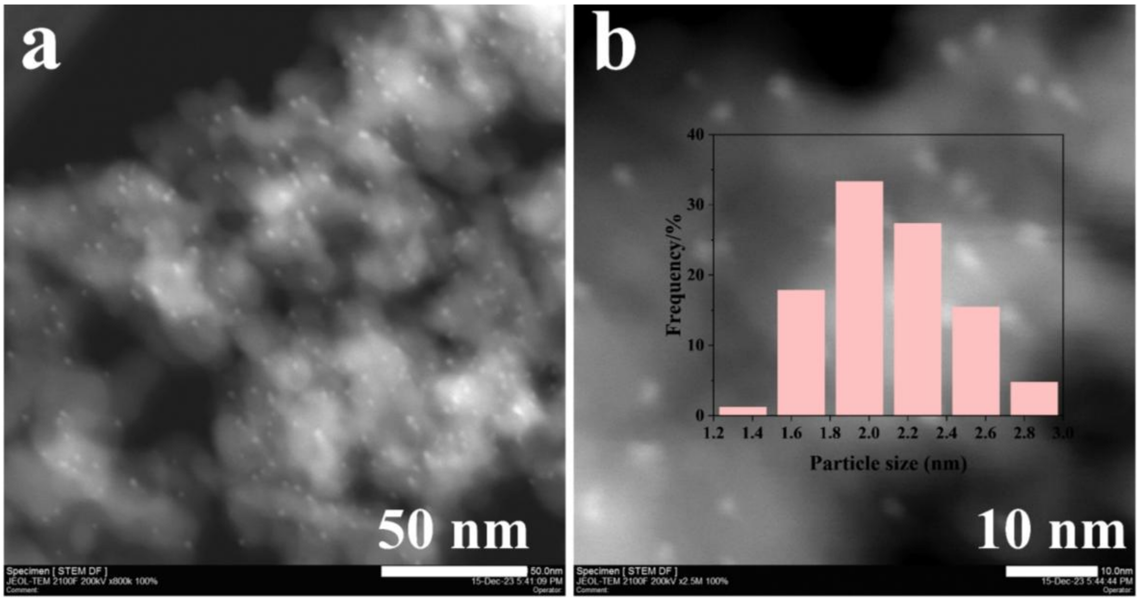

Figure S95. 2nm $PdGa_1/SiO_2$-700RED with Aerosil-200 as support. Particle size distribution on the right side. The average particle sizes of each sample were calculated by the following equation within a statistical analysis of 50 particles: $d_{TEM} = \Sigma n_i d_i^3 / \Sigma n_i d_i^2$.

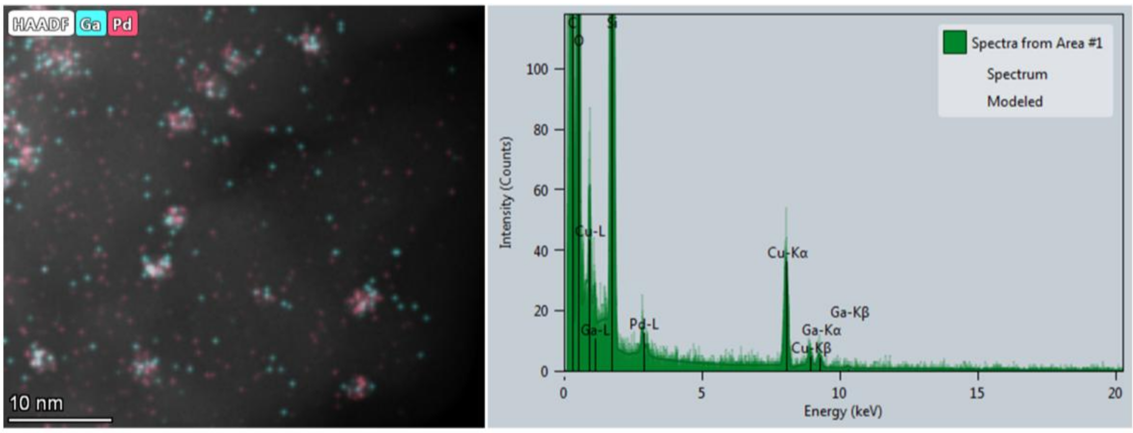

Z	Element	Family	Atomic Fraction (%)	Atomic Error (%)	Mass Fraction (%)	Mass Error (%)	Fit error (%)
31	Ga	K	45.94	7.06	35.77	3.89	3.98
46	Pd	L	54.06	9.73	64.23	9.23	2.31

Figure S96. EDX mapping of 2nm $PdGa_1/SiO_2$-700RED. (the Pd/Ga molar ratio is close to theoretical ratio).

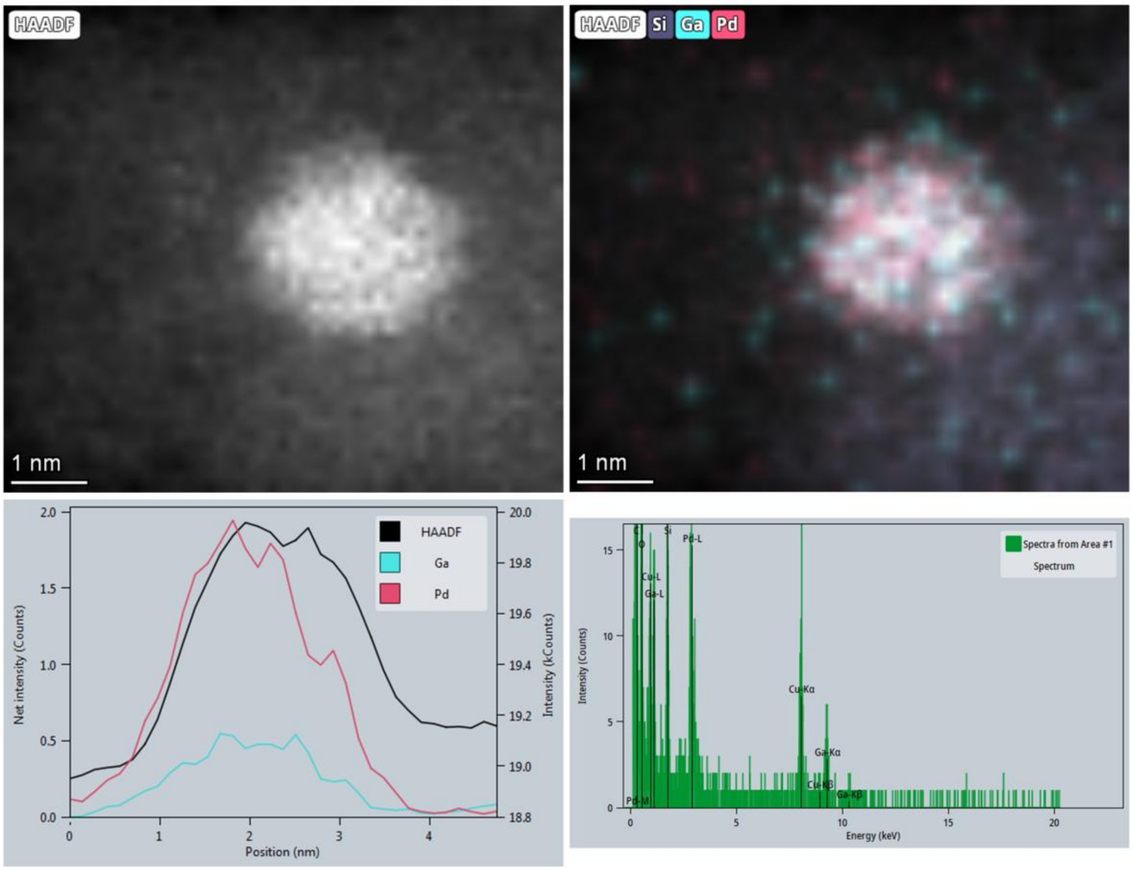

Z	Element	Family	Atomic Fraction (%)	Atomic Error (%)	Mass Fraction (%)	Mass Error (%)	Fit error (%)
31	Ga	K	31.07	5.69	22.8	2.99	5.13
46	Pd	L	68.93	13.63	77.2	11.66	2.03

Figure S97. EDX mapping of 2nm $PdGa_1/SiO_2$-700RED with zoomed area. (It indicates

the nanoparticle is PdGa alloy with Pd-rich structure)

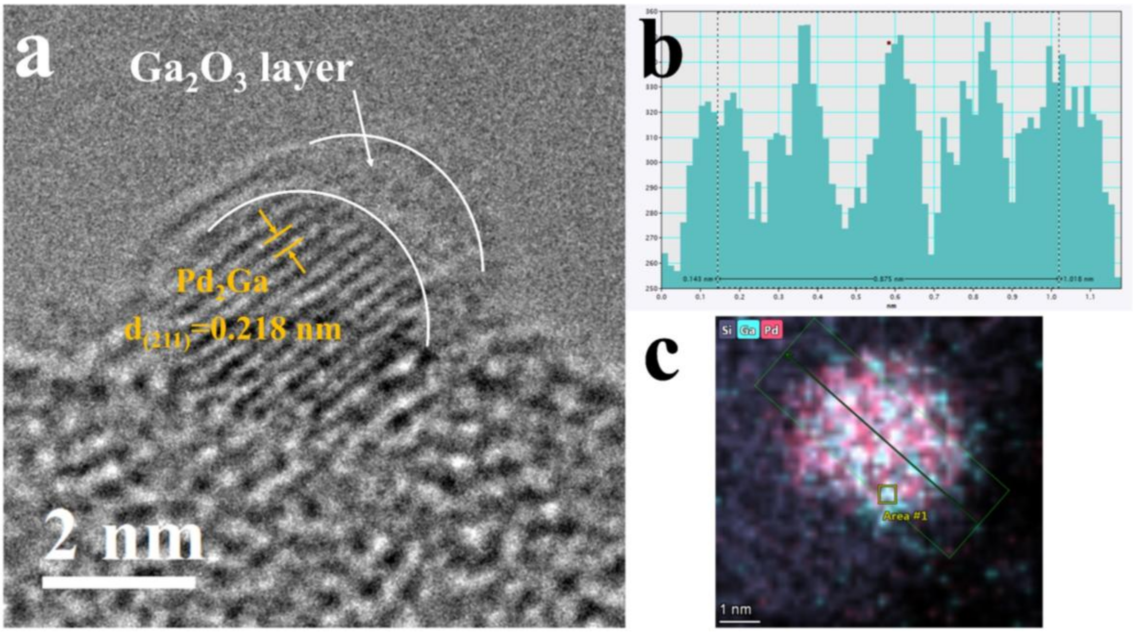

Figure S98. (a) TEM images of *ex situ* reduced 2nm $PdGa_1/SiO_2$-700RED sample and air exposure. (b)The planar distances 2.18 Å, corresponding to the lattice distance of {211} plane of Pd_2Ga. (c) EDX map indicate GaO_x layers on PdGa alloy surface, Pd (red), Ga (cyan).

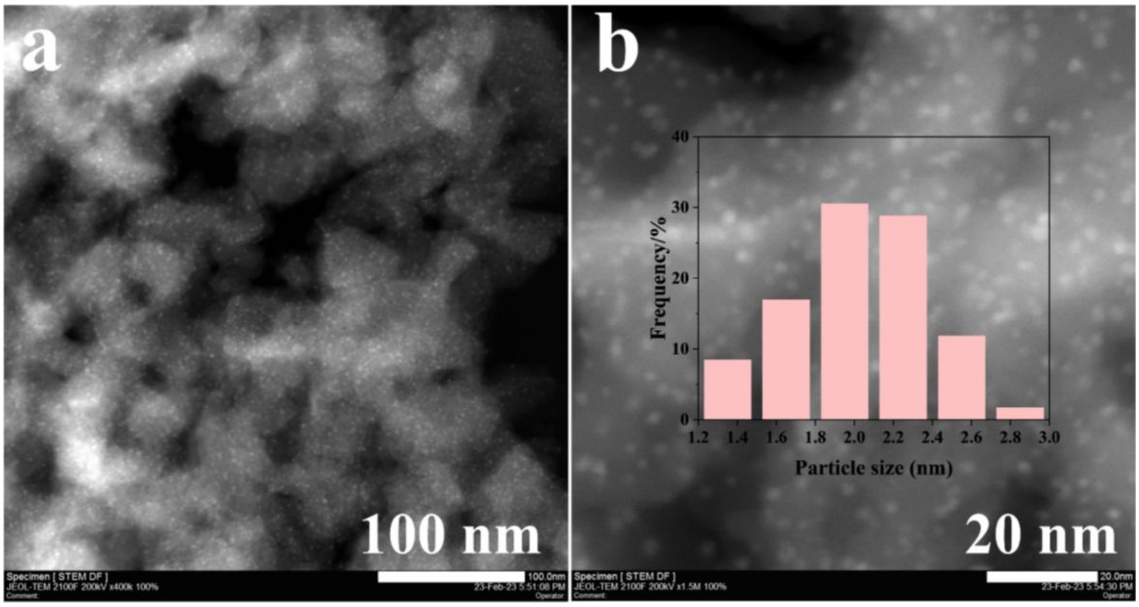

Figure S99. 2nm $PdGa_4/SiO_2$-700RED with Aerosil-200 as support. Particle size distribution on the right side. The average particle sizes of each sample were calculated by the following equation within a statistical analysis of 50 particles: $d_{TEM} = \Sigma n_i d_i^3/\Sigma n_i d_i^2$.

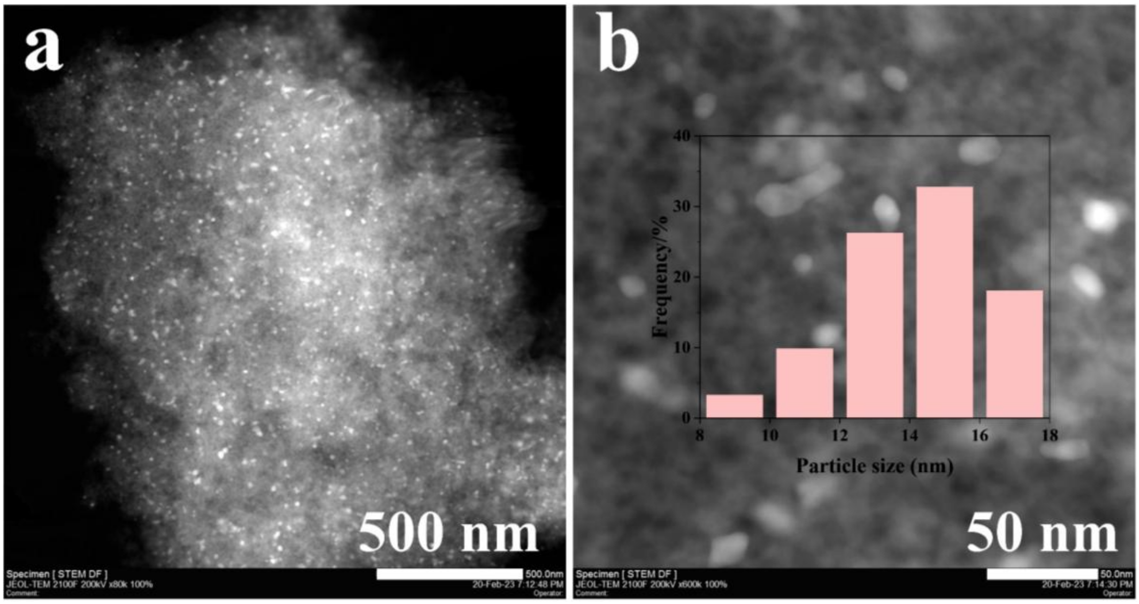

Figure S100. 15nm $PdGa_4/SiO_2$-700RED with high-surface-area SiO_2 as support. Particle size distribution on the right side. The average particle sizes of each sample were calculated by the following equation within a statistical analysis of 50 particles: $d_{TEM} = \Sigma n_i d_i^3 / \Sigma n_i d_i^2$.

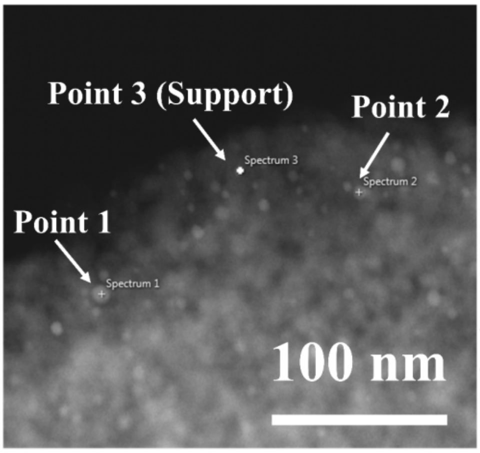

Spectrum 1

Element	Line Type	k Factor	k Factor type	Absorption Correction	Wt%	Pd/Ga molar ratio
O	K series	2.02	Theoretical	1	12.23	1.43
Si	K series	1	Theoretical	1	76.1	
Ga	K series	1.369	Theoretical	1	3.67	
Pd	K series	9.167	Theoretical	1	8	
Total:					100	

Spectrum 2

Element	Line Type	k Factor	k Factor type	Absorption Correction	Wt%	Pd/Ga molar ratio
O	K series	2.02	Theoretical	1	12.86	2.75
Si	K series	1	Theoretical	1	74.82	
Ga	K series	1.369	Theoretical	1	2.37	
Pd	K series	9.167	Theoretical	1	9.96	
Total:					100	

Spectrum 3

Element	Line Type	k Factor	k Factor type	Absorption Correction	Wt%	Pd/Ga molar ratio
O	K series	2.02	Theoretical	1	13.01	0.11
Si	K series	1	Theoretical	1	85.4	
Ga	K series	1.369	Theoretical	1	1.37	
Pd	K series	9.167	Theoretical	1	0.22	
Total:					100	

Figure S101. Point analysis of 15nm $PdGa_4/SiO_2$-700RED with high-surface-area SiO_2 as support. (It indicates formed PdGa alloy is Pd rich structure. Besides, except partially alloyed Ga, some un-alloyed GaO_x still located on SiO_2 surface, which is drawn from the comparison between Point 1, 2 and SiO_2 support of Point 3.)

8.2.3. XRD

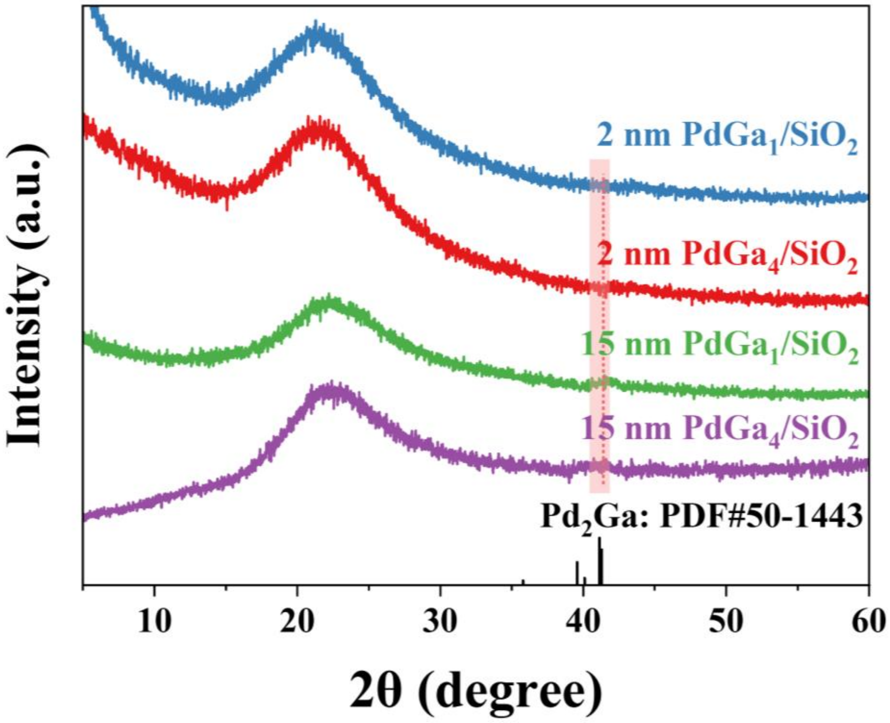

Figure S102. XRD result of supported $PdGa/SiO_2$ after ex situ 700 ºC H_2 reduction and finally air exposed.

Due to small size of PdGa, which is under limitation of XRD detection, there is no indication of Pd_2Ga formation on 2nm $PdGa_x/SiO_2$. However, a clear peak at around 42º indicate the formation Pd_2Ga in the bigger 15nm $PdGa_x/SiO_2$ catalysts.

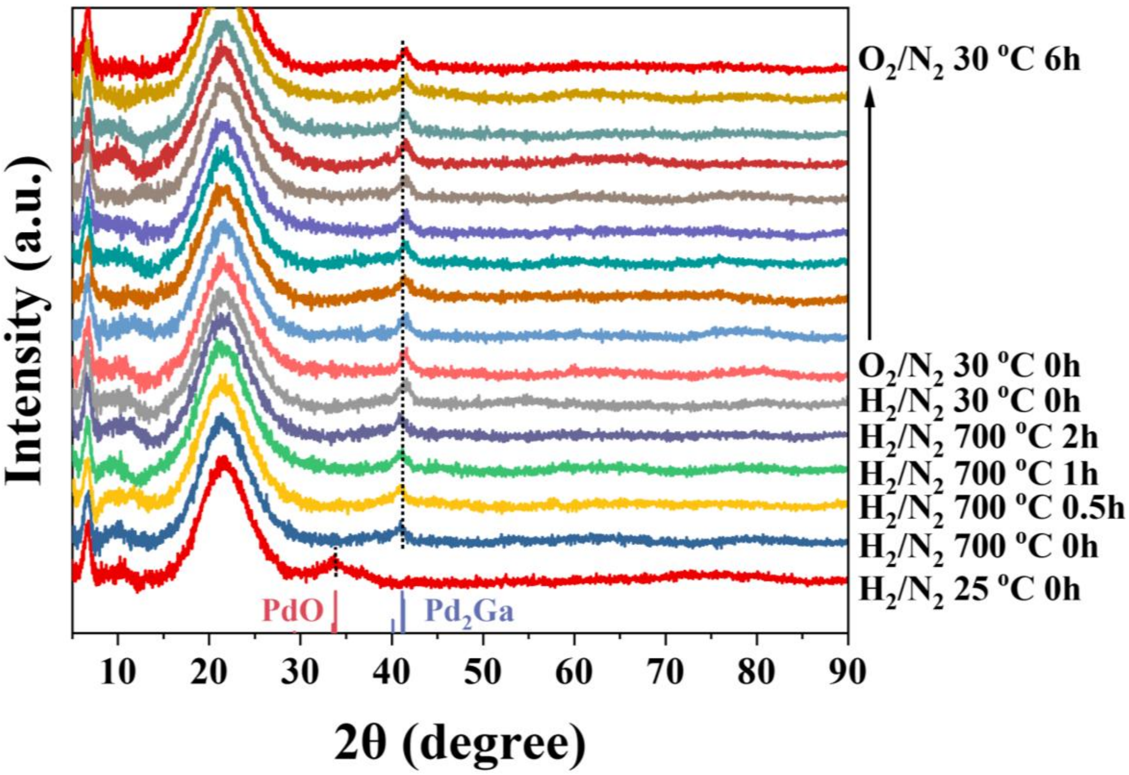

Figure S103. *In-situ* XRD result of 15nm $PdGa_4/SiO_2$ under H_2 reduction and finally air exposed 6h.

A sharp peak below 10° and broad peak at around 23° is related the mesoporous and amorphous SiO_2 structure. There is one peak at 34° before reduction is assigned to PdO, and a new peak at 42° occurs after 700 °C reduction 2h, which is ascribed to the formation of PdGa alloy (Pd_2Ga) (https://www.crystallography.net/cod/result.php).

8.2.4. XAS

XAS analysis at the Ga K-edge in the 2nm $PdGa_1/SiO_2$ sample shows that Ga is mainly as tetrahedral Ga^{3+} in the fresh sample with minor octahedral Ga(III) oxide phase impurities. Rising the temperature under H_2 conditions the tetrahedral Ga^{3+} species partially transform (from 90-95% to 30%) into GaPd alloy (from 0 to 40-45%), and Ga^{+}, octahedral Ga(III) oxide phase, and a reduced Ga species, compatible with a disordered Ga-OPd/Ga cluster (called E) (the latter three Ga species, from 0 to 10%). The subsequent exposure to air at room temperature suppress the Ga-Pd alloying in favor of the tetrahedral Ga^{3+}, octahedral Ga(III) oxide phase, and E species, while the amount of Ga^{+} is kept constant to 10%. Rising again the temperature under H_2 condition the initial composition under H_2 atmosphere is restored and maintain constant under CO_2/H_2 reaction conditions.

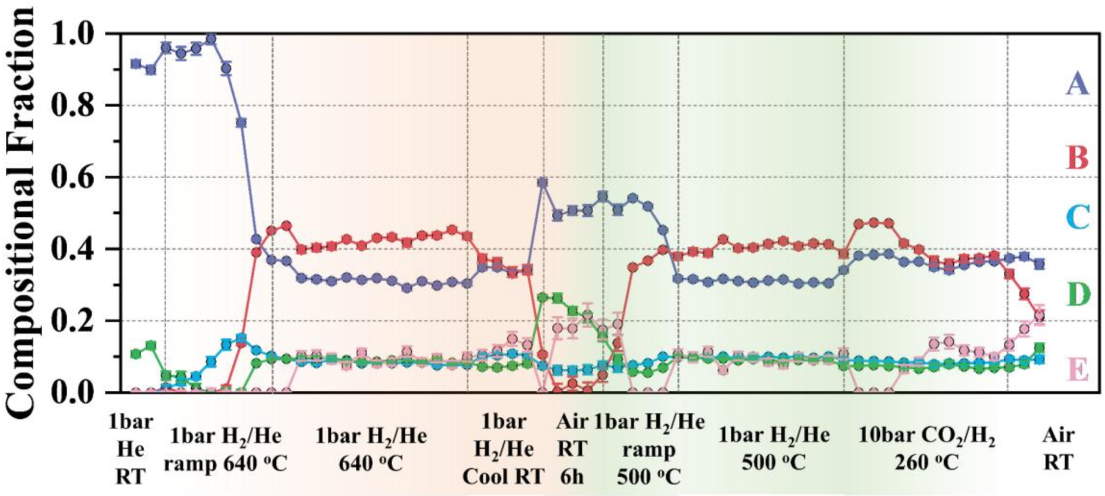

Figure S104. Quantitative evolution of the MCR components for the 2nm $PdGa_1/SiO_2$ dried sample under *in-situ* H_2 reduction followed by CO_2/H_2 reaction conditions. Component code: A = tetrahedral Ga^{3+}, B = PdGa alloy, C = isolated Ga^{+}, D = octahedral Ga(III) oxide phase, and E= a reduced Ga species, compatible with a disordered Ga-OPd/Ga cluster.

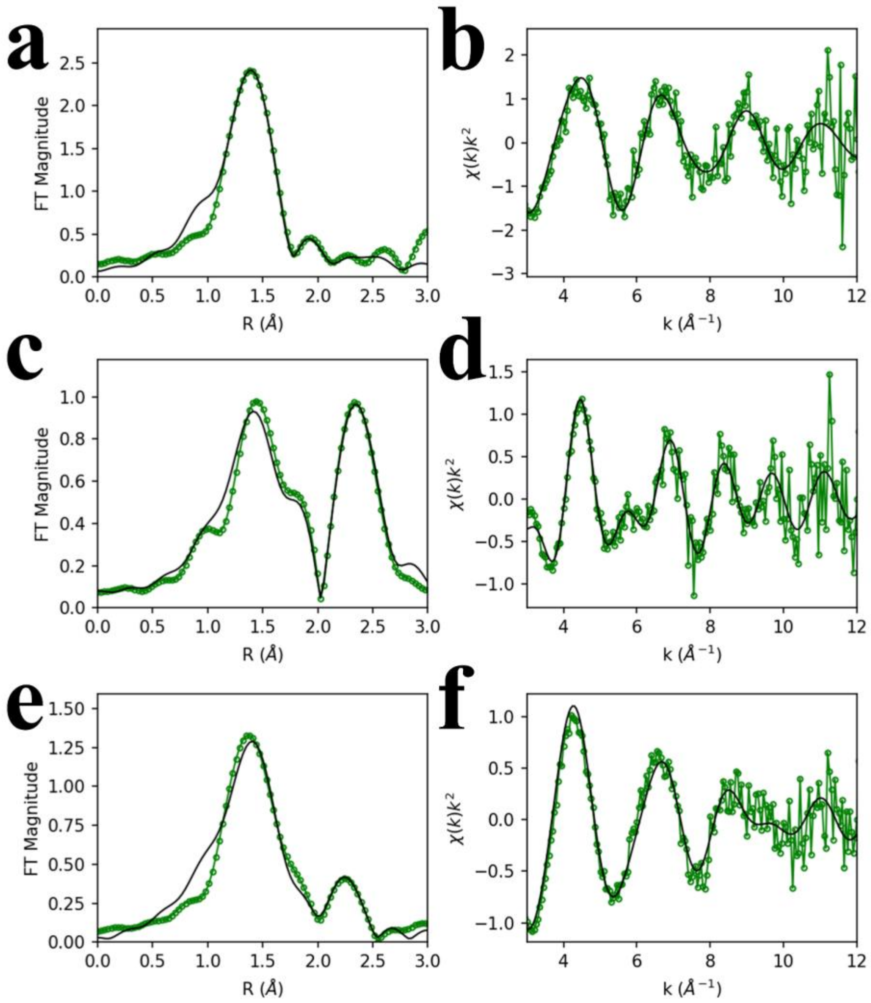

Figure S105. Ga K-edge k^2 weighted EXAFS oscillation and corresponding Fourier transform obtained for the 2nm $PdGa_1/SiO_2$-700RED sample: fresh (a, b), after reduction and then reaction (c, d), and after exposure to air (e, f).

Table S19. Ga K-edge EXAFS fitting results obtained for the 2nm $PdGa_1/SiO_2$-700RED sample: fresh, after reduction and then reaction, and after exposure to air. Multi (k^1, k^2, k^3)-weighted fits carried out in r-space (1-3 Å) over a k-range of 3-12 $Å^{-1}$ using a Hannings window (dk 1), and S_0^2 = 0.9. Bond distances and disorder parameters (Δr_{eff} and σ^2) were allowed to float having initial values of 0.0 Å and 0.003 $Å^2$ respectively, with a universal E_0 and ΔE_0 = 0 eV. Data was acquired at room temperature.

**Sample**	**R_{FACTOR}**	**X^2_v**	**Var. No.**	**k-range ($Å^{-1}$)**	**r-range (Å)**	**ΔE_0 (eV)**	**Ga-O**			**Ga-Si**			**Pd-Ga**		
							**CN**	**r (Å)**	**σ^2 (x10^3 $Å^2$)**	**CN**	**r (Å)**	**σ^2 (x10^3 $Å^2$)**	**CN**	**r (Å)**	**σ^2 (x10^3 $Å^2$)**
**2nm $PdGa_1/SiO_2$-700RED-Fresh**	0.014	1.5	5	3-12	1-3	0.3 (1.5)	4.5	1.81 (0.01)	2.0 (0.5)	2	2.67 (0.02)	8.5 (3.4)	-	-	-
**2nm $PdGa_1/SiO_2$-700RED-Reaction**	0.013	1.5	5	3-12	1-3	2.6 (0.9)	2	1.82 (0.01)	5.9 (0.001)	-	-	-	3	2.51 (0.01)	8.6 (0.6)
**2nm $PdGa_1/SiO_2$-700RED-Air**	0.016	6	5	3-12	1-3	0.0 (1.7)	3.5	1.85 (0.01)	6.3 (0.8)	-	-	-	0.5	2.50 (0.02)	4.3 (0.2)

8.2.5. IR-CO

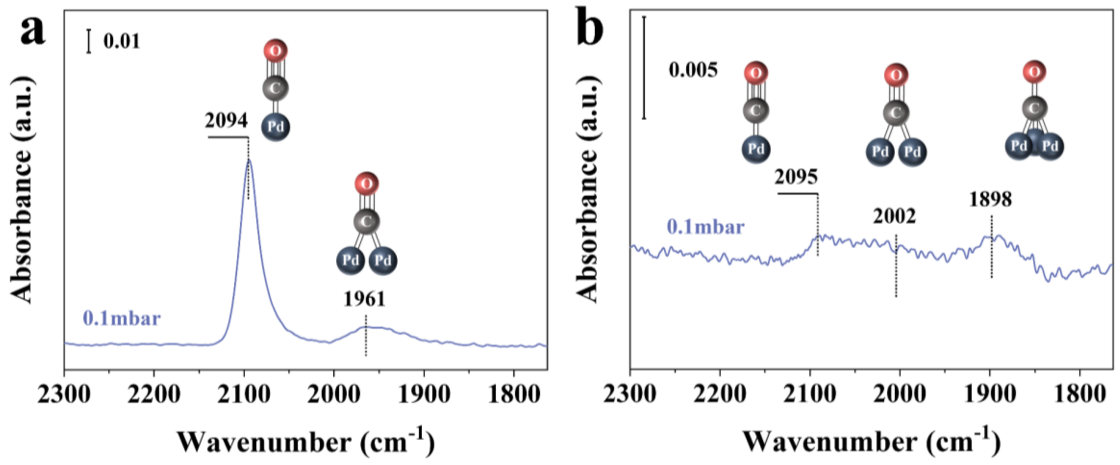

Figure S106. IR-CO at -100 ºC at 0.1 mbar CO dosing on (a) 2nm $PdGa_1/SiO_2$-700RED and (b) 2nm $PdGa_4/SiO_2$-700RED catalysts.

On the 2nm $PdGa_1/SiO_2$ sample (Figure S106a), the IR peak at 2094 cm^{-1} is due to CO on isolated Pd sites, whereas the IR peak at 1961 cm^{-1} is due to bridge coordinated CO species stabilized on Pd domains. This result confirms the co-existence of both isolated Pd sites and Pd domains on this sample.

On the 2nm $PdGa_4/SiO_2$ sample the IR bands are of very weak intensity (Figure S106b), which can be related to the blocking of metal sites by the formation of a GaO_x shell. The formation of the shell is confirmed by CO chemisorption analysis (Table S20) and the STEM images of Figure S98.

8.2.6. CO chemisorption

Table S20. CO chemisorption[a]

Sample	Treatment	Dispersion (%)	Mono layer (μmol/g)
$PdGa_4$@MFI-700RED[b]	*In-situ* 350 °C reduction	11.1 ± 2.2	6.26 ± 1.2
2nm $PdGa_1/SiO_2$-AS	*In-situ* 700 °C reduction	11.07	6.24
2nm $PdGa_1/SiO_2$-700RED	*In-situ* 350 °C reduction	1.11	1.05
2nm $PdGa_1/SiO_2$-700RED	*In-situ* 500 °C reduction	7.23	4.08
2nm $PdGa_4/SiO_2$-AS	*In-situ* 700 °C reduction	4.71	2.66
2nm $PdGa_4/SiO_2$-700RED	*In-situ* 500 °C reduction	0.52	0.29

[a] Pd:CO=1:1.
[b] $PdGa_4$@MFI were tested 4 times to get error range.

When the 2nm $PdGa_1/SiO_2$ sample is *in-situ* reduced at 700 ºC, the CO adsorption capacity is in the order of 6 μmol/g . However, when $PdGa_1/SiO_2$-700RED is exposed to air, the formation of GaO_x shell is observed from STEM images (Figure S98). A subsequent reduction of at least 500 °C is essential to restore surface PdGa alloy structure. For $PdGa_4/SiO_2$ sample, such GaO_x shell is thicker, making it more challenging to restore the pristine surface alloy structure.

8.3. Catalytic performance of supported $PdGa_x/SiO_2$

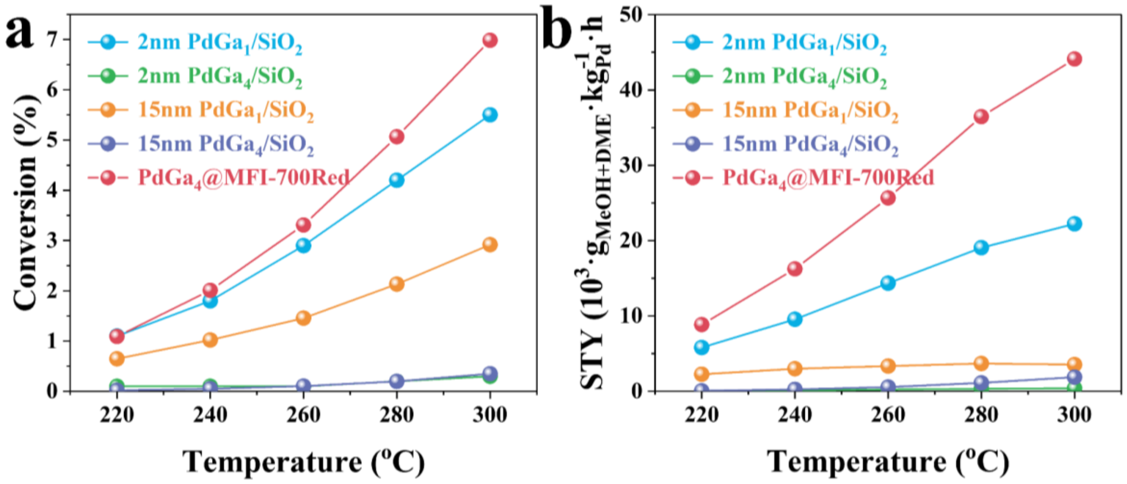

Figure S107. CO_2 hydrogenation activity on supported $PdGa_x/SiO_2$-700RED catalyst with different Ga/Pd ratio (a) Temperature versus CO_2 conversion, (b) Temperature versus STY. Reaction conditions: WHSV = 15000 mL·g_{cat}^{-1}·h^{-1}, Flow = 50 mL/min, T = 220-300 ºC, P = 20 bar. Before reaction, *in-situ* reduction at 500 ºC with 20 mL/min H_2 2h.

Table S21. Catalytic performance of the $PdGa_x/SiO_2$-700RED catalysts at different reaction temperatures compared to the $PdGa_4$@MFI-700RED sample.

Sample	Temp. (°C)	Conver. (%)	Selec. (%) MeOH+DME (MeOH/DME/CO)	Yield to MeOH and DME		
				Per mass of catalyst ($g_{MeOH+DME}$ ·kg_{cat}^{-1}·h^{-1})	Per mass of Pd ($g_{MeOH+DME}$ ·kg_{Pd}^{-1}·h^{-1})	TOF Per mole of surface exposed Pd sites ($mol_{MeOH+DME}$·$mol_{Pd\ sur}^{-1}$·h^{-1})
$PdGa_4$@MFI-700RED	220	1.1	78 (19/59/22)	53.07	8844.55	198.81
	240	2.0	78 (18/60/22)	97.48	16247.28	363.45
	260	3.3	75 (18/57/25)	153.95	25658.51	575.27
	260 [a]	5.2	80 (19/61/20)	257.19	42864.47	959.91
	280	5.1	70 (18/52/30)	218.77	36461.47	822.70
	300	7.0	62 (18/44/38)	264.72	44120.55	1006.01
2nm $PdGa_1/SiO_2$-	220	1.1	79 (79/0/21)	37.72	5802.50	288.11
	240	1.8	75 (74/1/25)	62.03	9543.49	473.20

700RED	260	2.9	71 (70/1/29)	93.27	14349.31	710.07
	280	4.2	65 (64/1/35)	123.89	19060.29	939.55
	300	5.5	58 (56/2/43)	144.51	22231.59	1089.27
2nm $PdGa_4/SiO_2$-700RED	220	0.1	8 (7/1/78)	0.17	24.69	18.03
	240	0.1	19 (16/3/69)	0.71	101.29	71.26
	260	0.1	24 (15/9/65)	1.62	230.77	149.76
	280	0.2	20 (12/8/70)	2.27	324.53	208.03
	300	0.3	16 (10/6/75)	2.81	401.04	259.24
15nm $PdGa_1/SiO_2$-700RED	220	0.6	45 (45/0/55)	13.46	2243.29	-
	240	1.0	38 (38/0/62)	17.87	2978.57	-
	260	1.5	30 (30/0/70)	20.05	3340.93	-
	280	2.1	22 (22/0/78)	22.08	3679.26	-
	300	2.9	16 (16/0/84)	21.22	3535.99	-
15nm $PdGa_4/SiO_2$-700RED	220	0	35 (35/0/65)	0.30	57.82	-
	240	0.1	51 (47/4/48)	1.26	242.93	-
	260	0.1	54 (41/13/46)	2.88	554.77	-
	280	0.2	55 (36/19/45)	5.72	1099.41	-
	300	0.3	51 (30/21/49)	9.70	1866.27	-

Reaction conditions: CO_2/H_2 = 1/3, WHSV=15000 mL·g_{cat}^{-1}·h^{-1}, Flow = 50 mL/min, T = 220-300 °C and P = 20 bar. The $PdGa_4$@MFI-700RED catalyst is *in-situ* reduced at 350 °C 20 mL/min H_2 for 2h, while all supported $PdGa_x/SiO_2$-700RED catalysts are *in-situ* reduced at 500 °C 20 mL/min H_2 for 2h.

[a] Reaction is done at 45 bar.

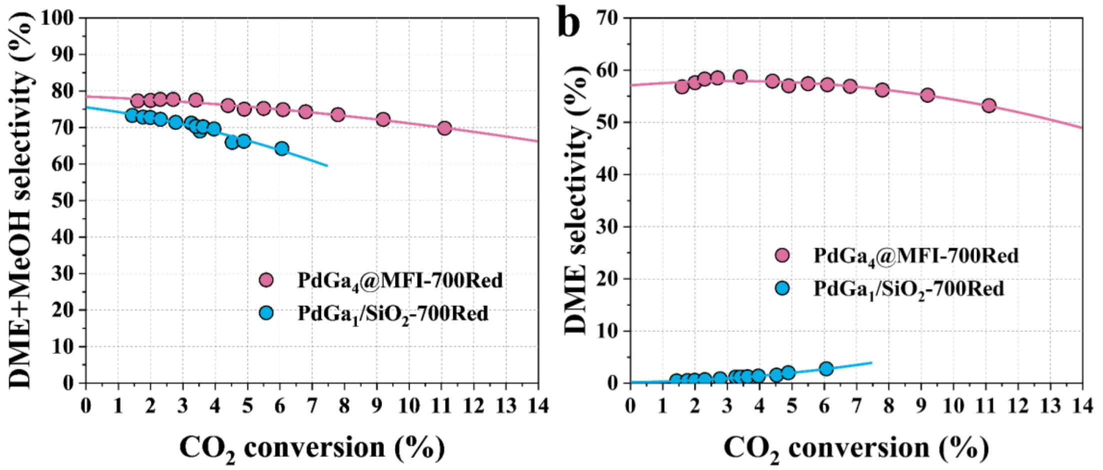

Figure S108. (a) DME+MeOH and (b) DME selectivity as a function of CO_2 conversion on $PdGa_4$@MFI-700RED and supported 2nm $PdGa_1/SiO_2$ catalysts, at 260 °C and 20 bar, WHSV=1800-45000 mL·g_{cat}^{-1}·h^{-1}.

8.4. Water effect on $PdGa_4$@MFI-700RED and $PdGa_1/SiO_2$ samples

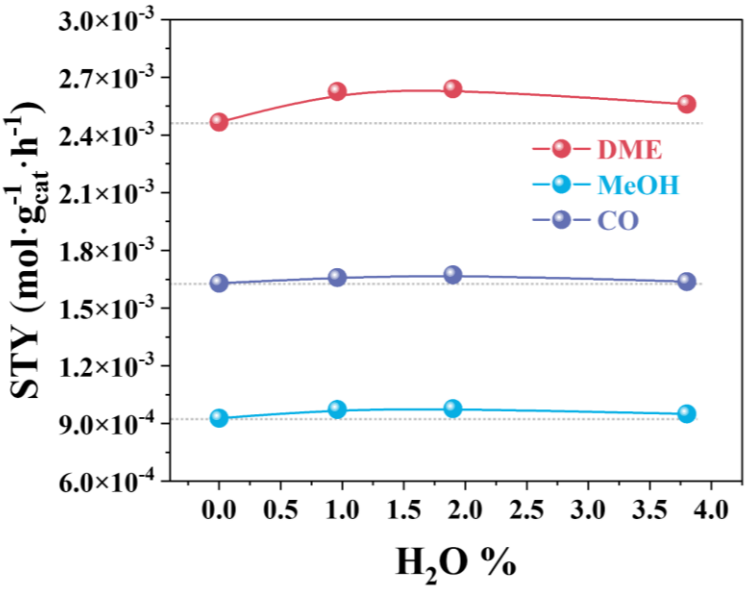

Figure S109. CO_2 hydrogenation activity with different molar ratio H_2O co-feeding for $PdGa_4$@MFI-700RED. Reaction conditions: WHSV = 30000 mL·g_{cat}^{-1}·h^{-1}, Flow = 50 mL/min, T = 260 ºC, P = 20 bar.

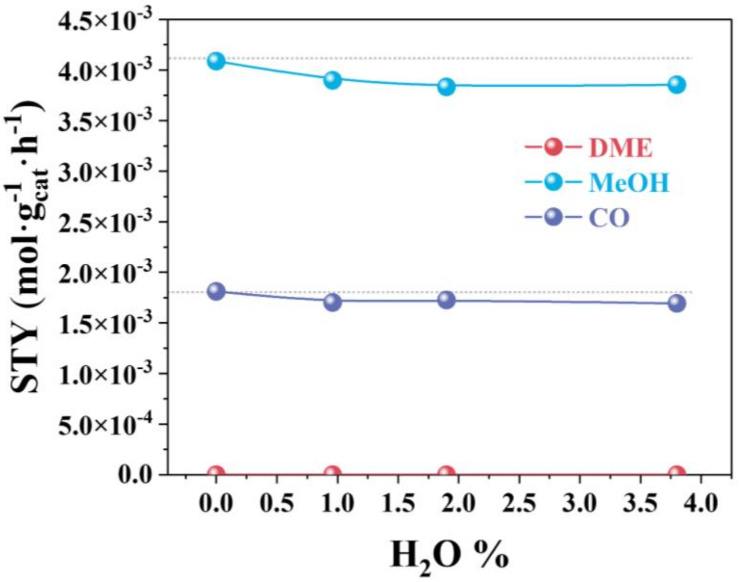

Figure S110. CO_2 hydrogenation activity with different molar ratio H_2O co-feeding for $PdGa_1/SiO_2$-700RED. Reaction conditions: WHSV = 30000 mL·g_{cat}^{-1}·h^{-1}, Flow = 50 mL/min, T = 260 ºC, P = 20 bar.

9. Alternative approaches for the preparation of PdGa@MFI

9.1. Synthesis method

In addition to the one-pot hydrothermal synthesis method, alternative approaches such as impregnation, ionic-exchange or physical mixing have been explored for the preparation of PdGa@MFI samples. These methods help overcome potential limitations of maximum metal loading in the one-pot synthesis process, which is approximately 0.6wt% for Pd and 1.6% for Ga. In this alternative approximation, Ga_4@S-1 (1.6 wt% Ga), Pd@S-1 (0.6wt% Pd), and Pd@ZSM-5 (Si/Al=200, 0.6wt% Pd) are prepared and used as supports.

For Pd impregnation on Ga_4@S-1, 0.0171g $Pd(acac)_2$ is first dissolved in 20 mL acetone solution, followed by the addition of 1g fresh Ga_4@S-1.The mixture undergoes ultrasonic treatment for 30min, then stirred at 80 ºC until acetone is fully evaporated. The resulting solid is dried in air at 100 °C and subsequently directly reduced in a tubular oven in pure H_2 (50 mL/min) with a heating rate of 10 °C/min to 700 °C, maintaining for 6 hours. The final solid is named as IM-Pd/Ga_4@S-1-700RED.

For Ga impregnation on Pd@S-1 or Pd@ZSM-5, 0.0759g $Ga(NO_3)\cdot xH_2O$ is first dissolved in 20 mL ethanol solution, then 1g fresh Pd@S-1 or Pd@ZSM-5 is added to aforementioned solution and subjected to ultrasonic treatment for 30min, followed by stirring at 80 ºC until ethanol is fully evaporated. The resulting solid is dried in air at 100 °C and then directly reduced in a tubular oven in pure H_2 (50 mL/min) with a heating rate of 10 °C/min to 700 °C, maintaining for 6 hours. The final solid is named as IM-Ga_4/Pd@S-1-700RED or IM-Ga_4/Pd@ZSM-5-700RED.

For ionic exchange, Pd@ZSM-5 is initially reduced at 500 ºC for 4h to remove organic template and generate acid sites for Ga^{3+} exchange. Subsequently, 0.0759g $Ga(NO_3)\cdot xH_2O$ is dissolved in 50 mL H_2O. Following this, 1g Pd@ZSM-5-500RED is added to aforementioned solution and subjected to ultrasonic treatment for 30min, followed by stirring at 80 ºC for 6h. The resulting solid product is collected by filtration and washed thoroughly with distilled water and dried in air at 100 °C. The dried sample is then directly reduced in a tubular oven in pure H_2 (50 mL/min) with a heating rate of 10 °C/min to 700 °C and maintain 10 hours. The final solid is named as IE-Ga_4/Pd@ZSM-5-700RED.

For physical mixing, 0.0211g commercial β-Ga_2O_3 is uniformly mixed with fresh Pd@ZSM-5 using a mortar and pestle for 10min, then directly reduced in a tubular oven in pure H_2 (50 mL/min) with a heating rate of 10 °C/min to 700 °C and maintain 10 hours.

The final solid is named as PM-Ga_2O_3/Pd@ZSM-5-700RED.

9.2. Physico-chemical properties

9.2.1 ICP analysis

Table S22. Physical-chemical property of alternative prepared PdGa @MFI samples.

Sample	Mean particle size (nm)	Si/Ga[a]	Ga/Pd[a]	Si/Al[a]	ICP Pd (wt%)	ICP Ga (wt%)
IM-Pd/Ga_4@S-1-700RED	2.2 (Some 25nm on zeolite surface)	74	4	-	0.68	1.65
IM-Ga_4/Pd@S-1-700RED	2.2	74	4	-	0.62	1.70
IM-Ga_4/Pd@ZSM-5-700RED	2.4	74	4	200	0.59	1.68
IE-Ga_4/Pd@ZSM-5-700RED	2.5	74	4	200	0.62	1.63
PM-Ga_2O_3/Pd@ZSM-5-700RED	2-7	74	4	200	0.60	1.58

[a] Theoretical Si/Ga, Ga/Pd and Si/Al ratio.

9.2.2. STEM

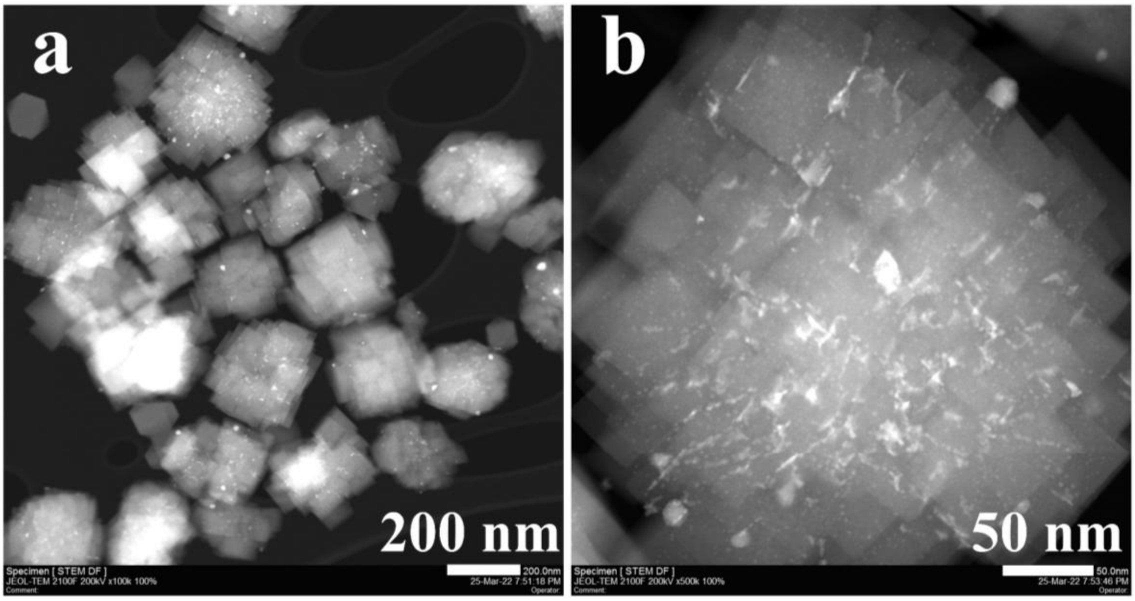

Figure S111. STEM result of impregnation method prepared IM-Pd/Ga_4@S-1-700RED.

Above STEM results indicate only partially Pd is confined inside MFI channel, but mostly are exist as big Pd nanoparticles on zeolite surface.

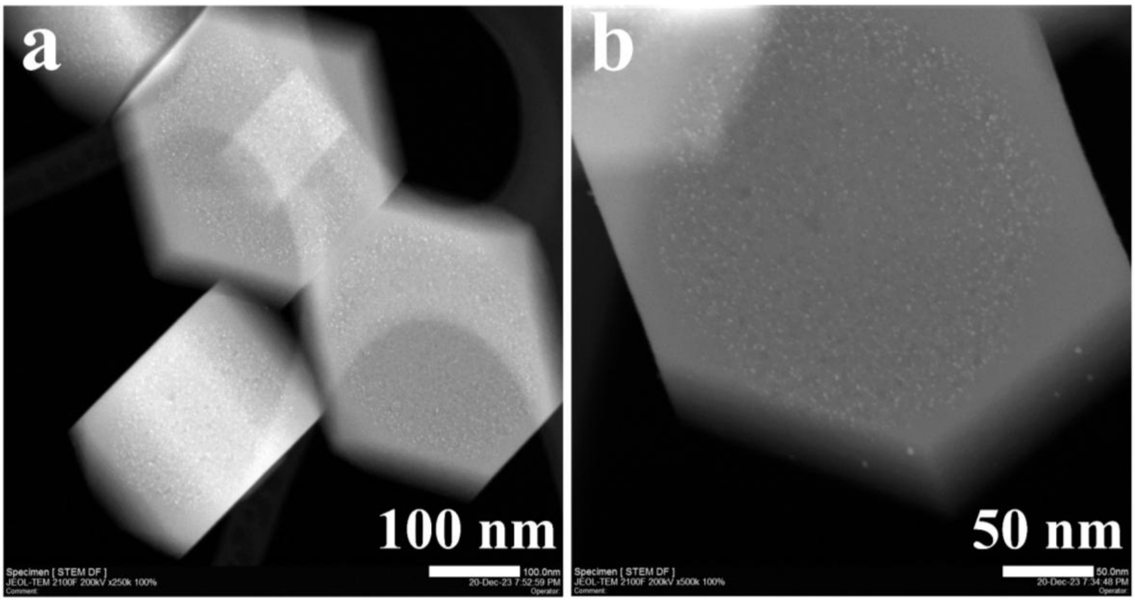

Figure S112. STEM result of impregnation method prepared IM-Ga_4/Pd@S-1-700RED.

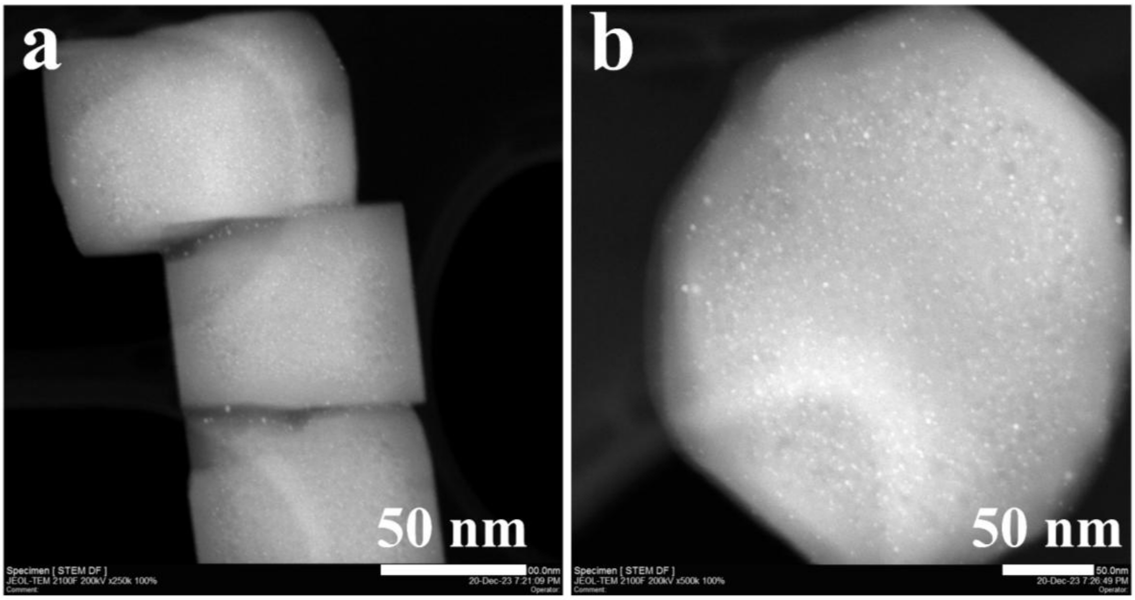

Figure S113. STEM result of impregnation method prepared IM-Ga_4/Pd@ZSM-5-700RED.

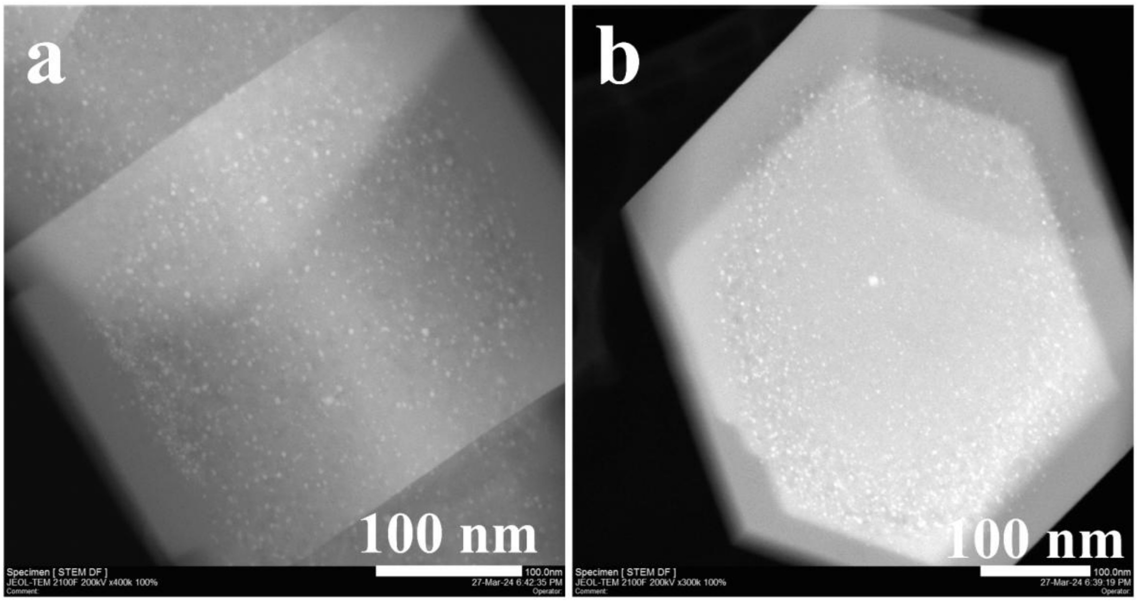

Figure S114. STEM result of ionic exchange method prepared IE-Ga_4/Pd@ZSM-5-700RED.

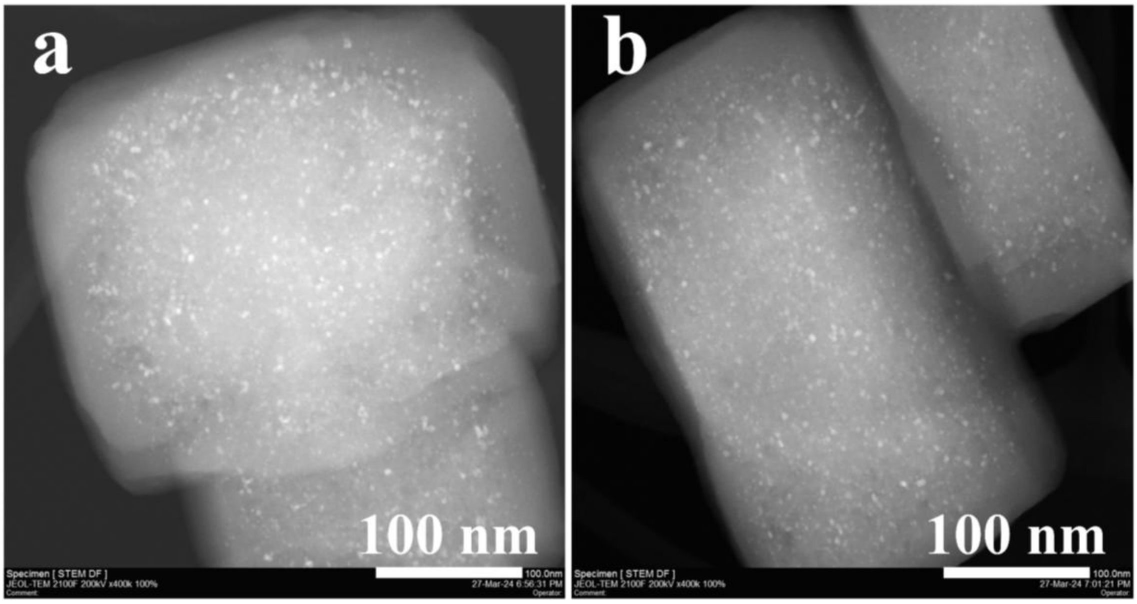

Figure S115. STEM result of power mixing method prepared PM-Ga_2O_3/Pd@ZSM-5-700RED.

Above results on IM-Ga_4/Pd@ZSM-5-700RED, IE-Ga_4/Pd@ZSM-5-700RED and PM-Ga_2O_3/Pd@ZSM-5-700RED indicate that impregnate or ionic exchange $Ga(NO_3)_3$ on Pd@ZSM-5 are able to get well metal confined inside zeolite structure, but the metal states should be characterized by further techniques.

9.2.3. IR-CO of alternative approach prepared PdGa@MFI

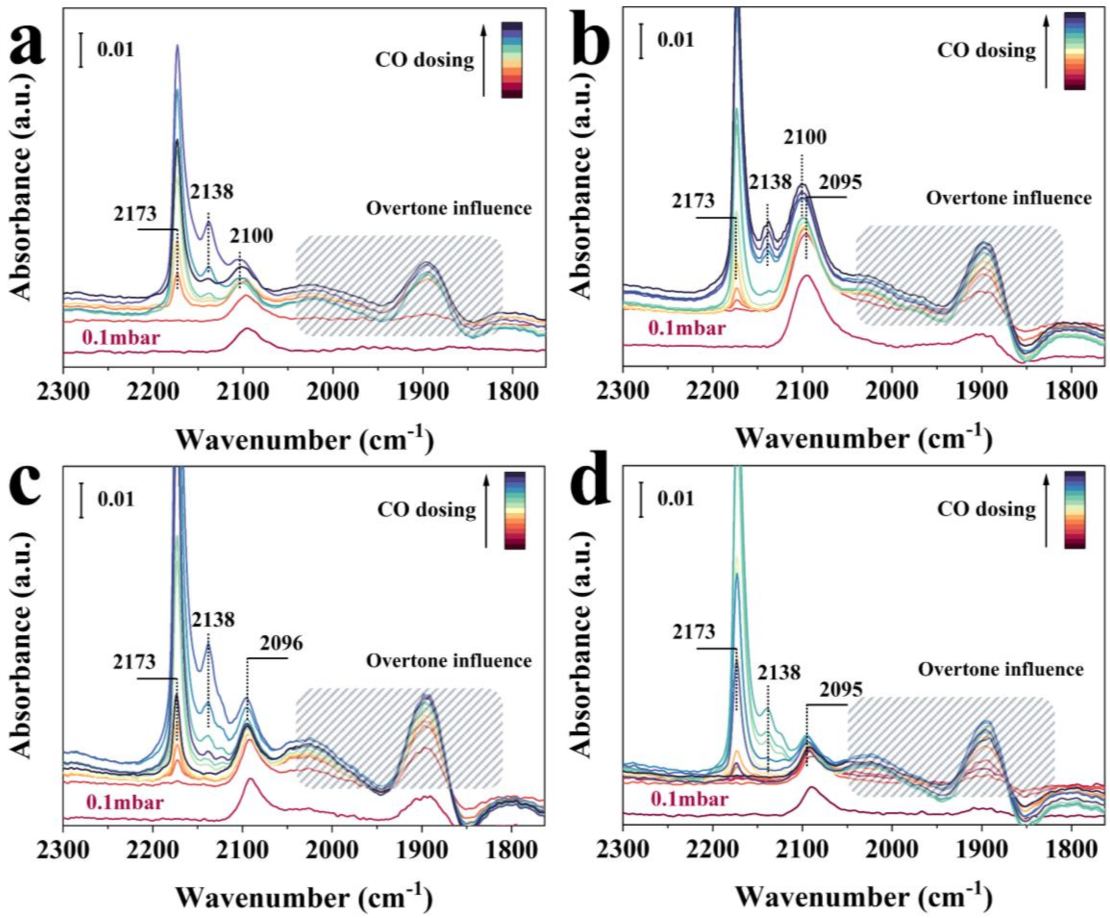

Figure S116. IR-CO at -100 ºC and at increasing CO dosing from 0.1 mbar to 3 mbar on alternative prepared PdGa@MFI-700RED catalysts: (a) IM-Ga_4/Pd@S-1-700RED, (b) IM-Ga_4/Pd@ZSM-5-700RED, (c) IE-Ga_4/Pd@ZSM-5-700RED, and (d) PM-Ga_2O_3/Pd@ZSM-5-700RED. In all samples, IR bands in the 1970-1890 cm^{-1} IR region are observed at increasing CO coverages above 0.1mbar which are due to a bad substraction of the reference sample in the overtone region. The region is marked in shadow and labelled as overtone influence.

9.2.4. XAS of alternative method prepared PdGa@MFI-700RED catalysts

XAS analysis at the Ga K-edge reveals that in the fresh sample Ga is in large extent oxidized, which is reasonable due to fresh IM-Ga_4/Pd@ZSM-5 is prepared by $Ga(NO_3)_3$ impregnate on Pd@ZSM-5 without any pretreatment. Indeed, the XANES spectra can be deconvoluted with the 96% of the octahedral Ga(III) oxide phase and the 4% of tetrahedral Ga^{3+} species. Rising the temperature under H_2 conditions a progressive transformation of the octahedral Ga(III) oxide species, into tetrahedral Ga^{3+} and isolated Ga^{+} species (Figure S117) is observed. The strong increase of the MCR component E associated to a reduced Ga species, compatible with a disordered Ga-OPd/Ga cluster and the concomitant tiny increase of the GaPd alloy at the expenses of the tetrahedral Ga^{3+}, octahedral Ga(III) oxide, and Ga^{+} species is visible by keeping the temperature to 650 °C under reaction conditions. Cooling down to room temperature the PdGa phase is again suppressed, and the E MCR component lose weight from 80 to 50% in favor of tetrahedral Ga^{3+} (from 5 to 30%) and Ga^{+} (from 0 to 18%). Unfortunately data under reaction conditions are not obtained.

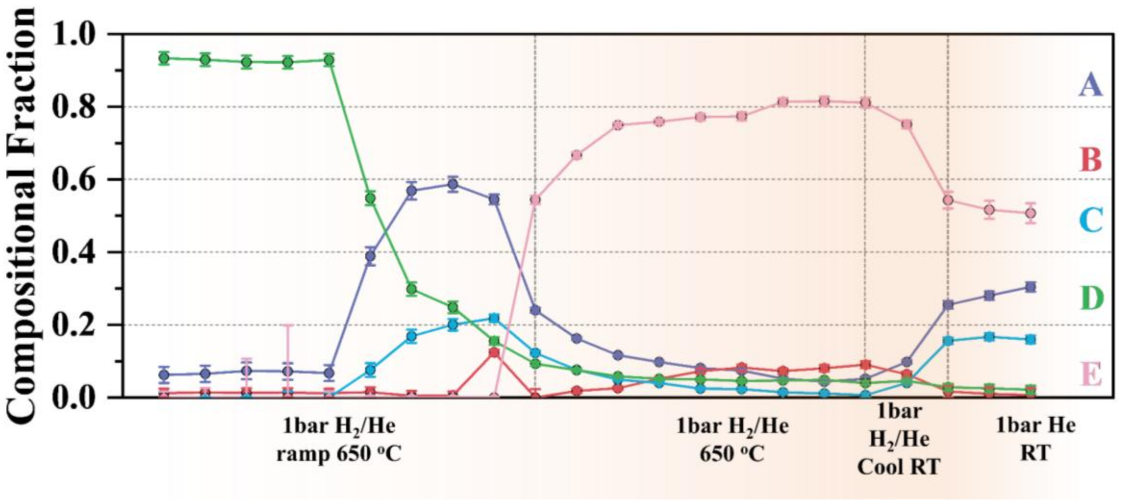

Figure S117. Quantitative evolution of the MCR components for fresh IM-Ga_4/Pd@ZSM-5 sample under *in-situ* H_2 reduction. Component code: A = tetrahedral Ga^{3+}, B = PdGa alloy, C= isolated Ga^{+}, D = octahedral Ga(III) oxide phase, and E = a reduced Ga species, compatible with a disordered Ga-OPd/Ga cluster.

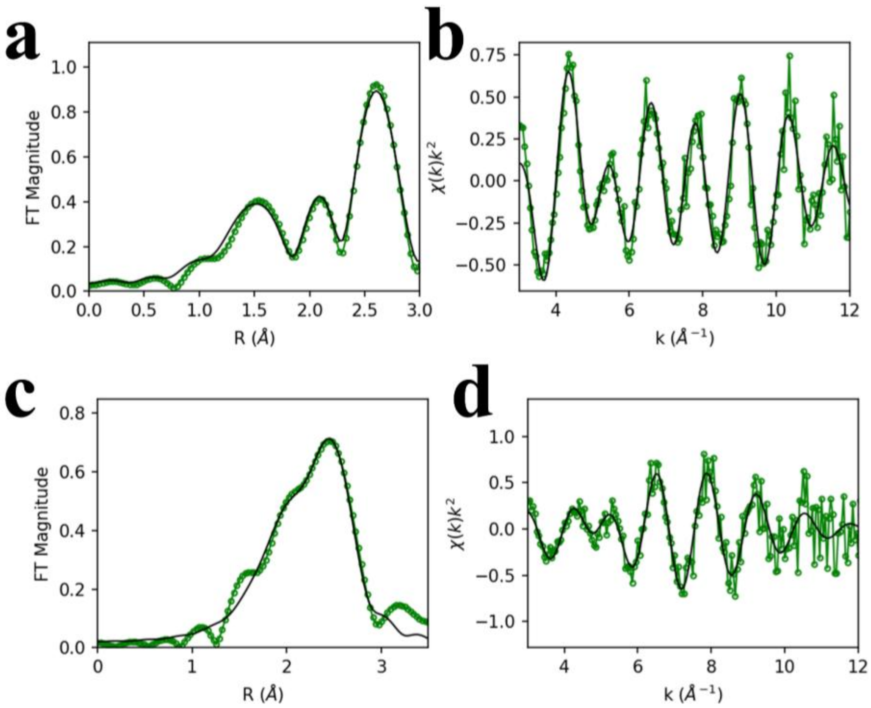

Figure S118. Pd K-edge k^2 weighted EXAFS oscillation and corresponding Fourier transform obtained for the IM-Ga_4/Pd@ZSM-5 sample: fresh (a, b), after reduction (c, d).

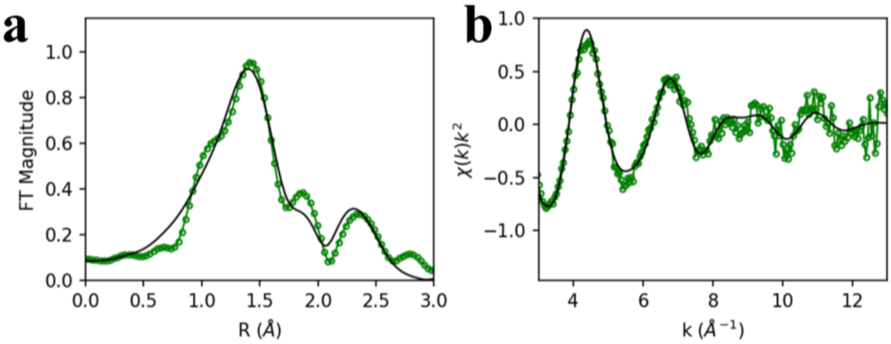

Figure S119. Ga K-edge k^2 weighted EXAFS oscillation and corresponding Fourier transform obtained for the IM-Ga_4/Pd@ZSM-5 sample: after reduction (a, b).

Table S23. Pd K-edge EXAFS fitting results obtained for the IM-Ga_4/Pd@ZSM-5 sample: fresh, after reduction. Multi (k^1, k^2, k^3)-weighted fits carried out in r-space (1-3 Å) over a k-range of 3-12 $Å^{-1}$ using a Hannings window (dk 1), and S_0^2 = 0.9. Bond distances and disorder parameters (Δr_{eff} and σ^2) were allowed to float having initial values of 0.0 Å and 0.003 $Å^2$ respectively, with a universal E_0 and ΔE_0 = 0 eV. Data was acquired at room temperature.

Sample	R_{FACTOR}	X^2_ν	Var. No.	k-range ($Å^{-1}$)	r-range (Å)	ΔE_0 (eV)	Pd-O			Pd-Ga			Pd-Pd		
							CN	r (Å)	σ^2 (x10^3 $Å^2$)	CN	r (Å)	σ^2 (x10^3 $Å^2$)	CN	r (Å)	σ^2 (x10^3 $Å^2$)
**IM-Ga_4/Pd@ZSM-5-Fresh**	0.008	3.3	5	3-12	1-3	1.9 (0.8)	1	1.99 (0.01)	3 (1)	-	-	-	4.5	2.81 (0.01)	7.7 (0.4)
**IM-Ga_4/Pd@ZSM-5-Reduction**	0.013	0.9	4	3-12	1-3	-3.1 (0.9)	-	-	-	3	2.52 (0.01)	10.1 (0.4)	4	2.78 (0.01)	10.1 (0.4)

Table S24. Ga K-edge EXAFS fitting results obtained for the IM-Ga_4/Pd@ZSM-5 sample: after reduction. Multi (k^1, k^2, k^3)-weighted fits carried out in r-space (1-3 Å) over a k-range of 3-13 $Å^{-1}$ using a Hannings window (dk 1), and S_0^2 = 0.9. Bond distances and disorder parameters (Δr_{eff} and σ^2) were allowed to float having initial values of 0.0 Å and 0.003 $Å^2$ respectively, with a universal E_0 and ΔE_0 = 0 eV. Data was acquired at room temperature.

Sample	R_{FACTOR}	X^2_ν	Var. No.	k-range ($Å^{-1}$)	r-range (Å)	ΔE_0 (eV)	Ga-O			Ga-Si			Pd-Ga		
							CN	r (Å)	σ^2 (x10^3 $Å^2$)	CN	r (Å)	σ^2 (x10^3 $Å^2$)	CN	r (Å)	σ^2 (x10^3 $Å^2$)
**IM-Ga_4/Pd@ZSM-5-Reduction**	0.011	17	7	3-13	1-3	-4.9 (1.3)	2	1.81 (0.01)	7.1 (1.4)	1.5	2.72 (0.03)	16.6 (9.7)	3	2.51 (0.01)	8.1 (0.7)

9.3. Catalytic performance of alternative prepared PdGa@MFI-700RED catalysts

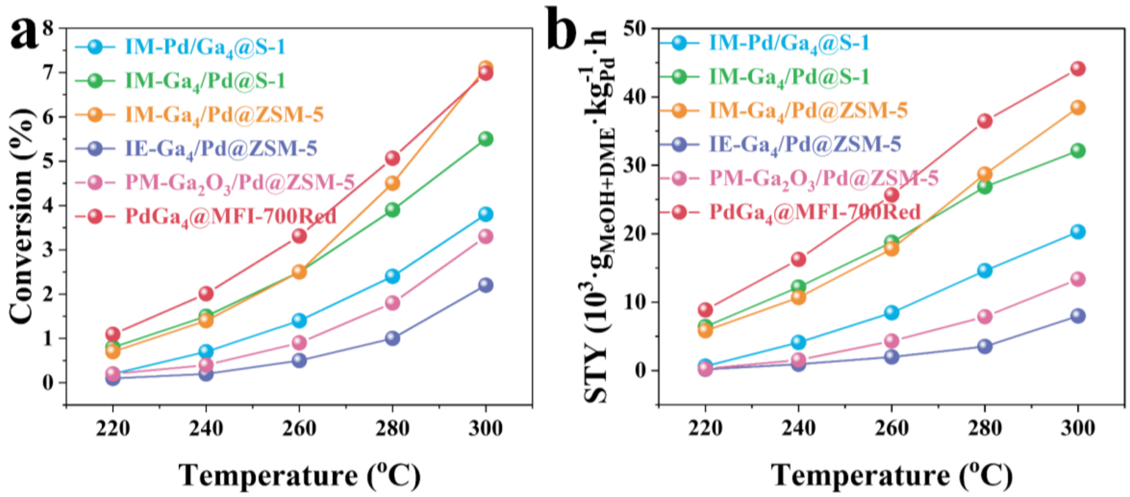

Figure S120. CO_2 hydrogenation activity on impregnation, ionic exchange and physical mixing methods prepared PdGa@MFI catalysts (a) Temperature versus CO_2 conversion, (b) Temperature versus STY. Reaction conditions: WHSV=15000 $mL\cdot g_{cat}^{-1}\cdot h^{-1}$, Flow=50 mL/min, T=220-300 °C, P=20bar. Before reaction, the samples were *in-situ* reduced at 350 °C with 20 mL/min H_2 2h.

10. Appendix

Appendix 1.

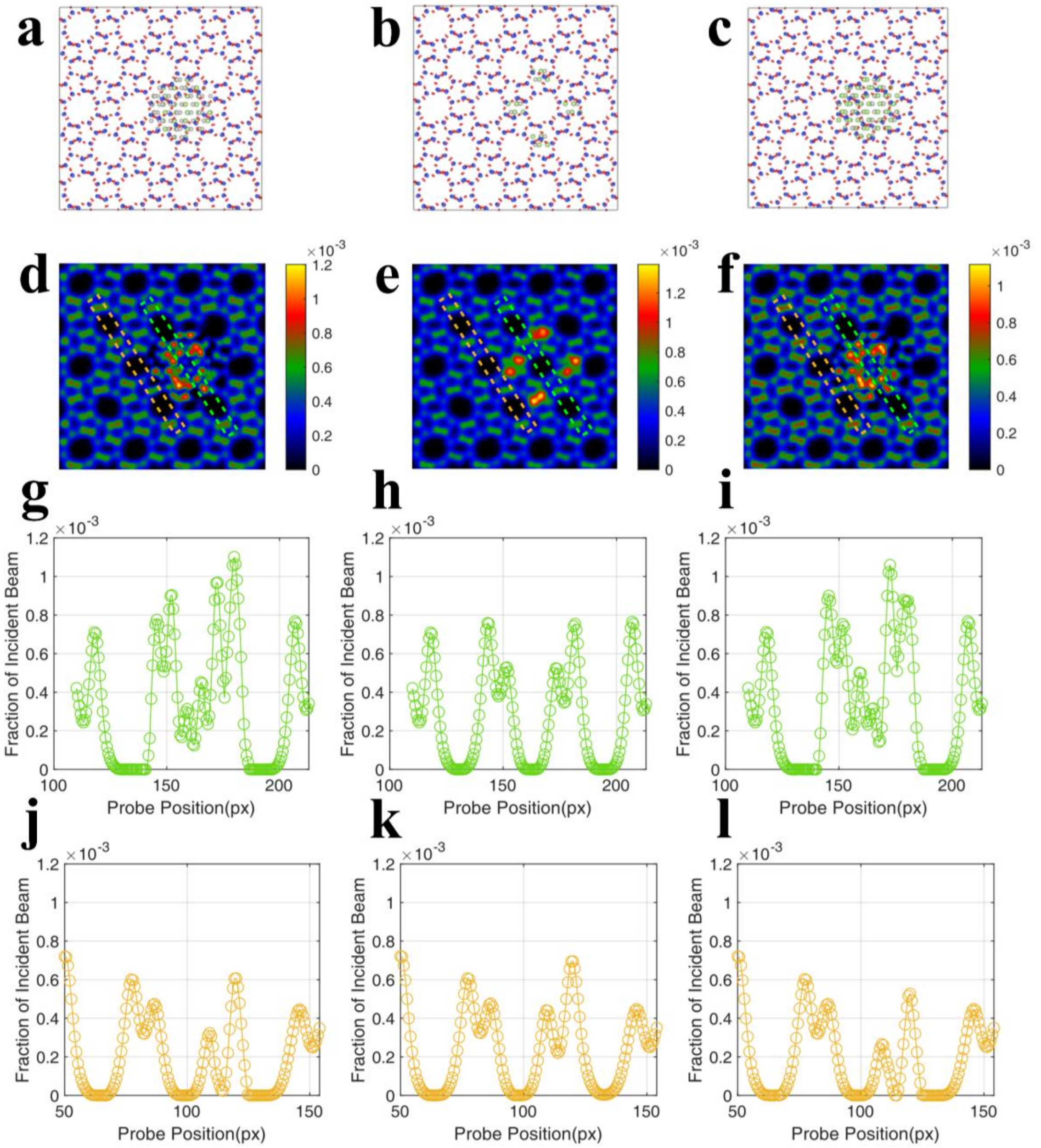

Figure S121. Structural models of: (a) Pd_2Ga nanoparticle, (b) Pd_2Ga clusters, and (c) $PdGa_5$ nanoparticle within MFI zeolite channels (straight and sinusoidal), viewed along the [010] direction. (d-f) Simulated HR-HAADF images based on these models. (g-i) Intensity profiles extracted from the nanoparticle/cluster positions (green markers). (j-l) Intensity profiles from particle-free regions (yellow markers).

Comments:

To determine the location of PdGa nanoparticles within the MFI zeolite, three distinct models were constructed (Figure S121): (i) a 2nm Pd_2Ga nanoparticle in the both straight and sinusoidal channel (a), (ii) Pd_2Ga clusters in the sinusoidal channels (b), and (iii) a 2nm $PdGa_5$ nanoparticle in in the both straight and sinusoidal channel (c). HR-HAADF image simulations (d-f) of these models exhibited similar contrast, with maximum intensities observed at the sinusoidal channel positions. To accurately compare intensities, profiles were extracted from the marked regions in the simulations. Specifically, intensity profiles were generated at the nanoparticle or cluster locations (g, h, i) and, as a control, through straight channels devoid of particles. Notably, the profiles from models with nanoparticles showed non-zero intensities specifically at the straight channel locations, while those from the cluster model exhibited zero intensities, consistent with the control. This indicates that nanoparticles within the straight channels produce a distinct intensity signature which can be used to distinguish the exact location of the nanoparticles/clusters. Significantly, experimental HR-HAADF intensity profiles also revealed non-zero intensities at the straight channel locations, confirming that PdGa nanoparticles are predominantly located within these channels, while the PdGa clusters are mainly at the sinusoidal channels (Figure S122).

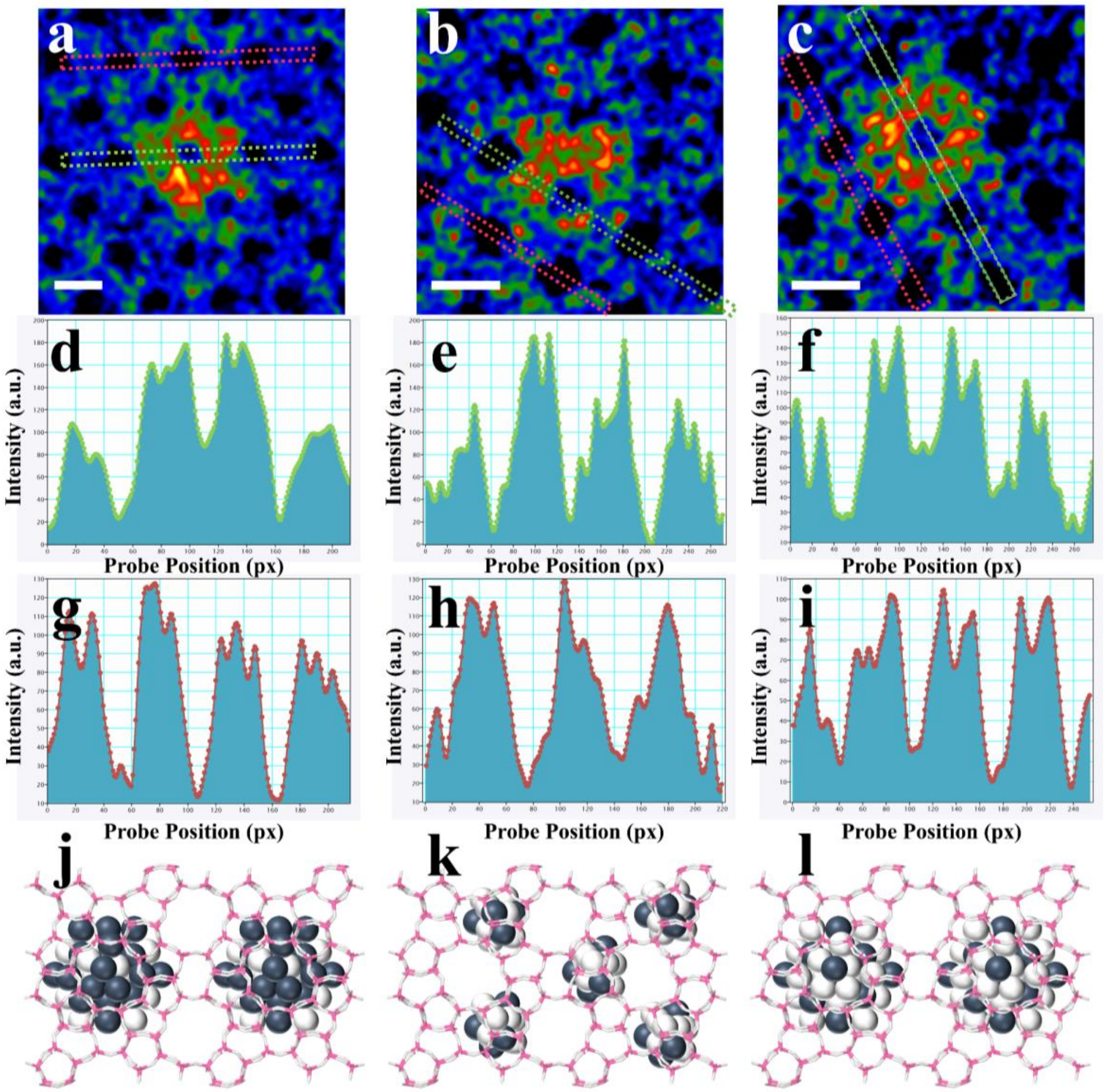

Figure S122. Experimental HR-HAADF-STEM images of $PdGa_1$@MFI-700RED (a, d, g), $PdGa_4$@MFI-700RED (b, e, h, c, f, i). An intensity profile recorded on the linear path marked on the image of a, b, c, crossing several consecutive straight channels in the presence of PdGa or not (d-i). (j, k, l) Illustration of different PdGa nanoparticles or clusters inside MFI zeolite along the [010] direction.

Combined with Figure S121 simulation results, the existence of PdGa nanoparticles (both in straight and sinusoidal pore channel) with some zeolite structure break down, clusters (only in sinusoidal pore channel) can be identified in both $PdGa_1$@MFI-700RED and $PdGa_4$@MFI-700RED samples, while the exactly Pd-rich or Ga-rich structure is hard to distinguish based on currently results.

Appendix 2.

Table S25. Peak assignments of surface species in the *in-situ* DRIFT study.

Peak (cm^{-1})	Assignment	Species
1575-1595	v_{as} (CO_2)	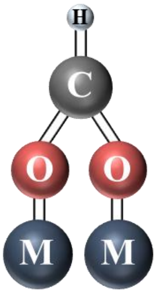 Bidentate formate
1360-1370	v_s (CO_2)	
1379-1383	δ (CH)	
2880-2890	v (CH)	
2970-2980	δ (CH) + v_{as} (CO_2)	
2731-2738	δ (CH) + v_s (CO_2)	
1640	v_{as} (CO_2)	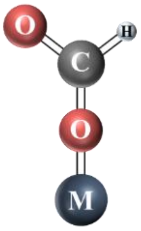 Monodentate formate
1300	v_s (CO_2)	
1350	δ (CH)	
2800	v (CH)	
1050-1060	v_s (CO)	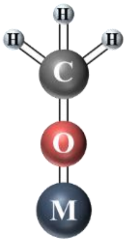 Methoxy
2930-2940	v_{as} (CH)	
2820-2850	v_s (CH)	

11. References

1. Simonelli, L. *et al.* CLÆSS: The hard X-ray absorption beamline of the ALBA CELLS synchrotron. *Cogent Phys.* **3**, 1231987 (2016).

2. Ravel, B. & Newville, M. ATHENA, ARTEMIS, HEPHAESTUS: data analysis for X-ray absorption spectroscopy using IFEFFIT. *J. Synchrotron Radiat.* **12**, 537-541 (2005).

3. Jaumot, J., de Juan, A. & Tauler, R. MCR-ALS GUI 2.0: New features and applications. *Chemom. Intell. Lab. Syst.* **140**, 1-12 (2015).

4. de Juan, A. & Tauler, R. Chapter 2 - Multivariate Curve Resolution-Alternating Least Squares for Spectroscopic Data. *Data Handl. Sci. technol.* **30**, 5-51 (2016).

5. Newville, M. EXAFS analysis using FEFF and FEFFIT. *J. Synchrotron Radiat.* **8**, 96-100 (2001).

6. Rehr, J. J. & Albers, R. C. Theoretical approaches to x-ray absorption fine structure. *Rev. Mod. Phys.* **72**, 621-654 (2000).

7. Newville, M. Larch: An Analysis Package for XAFS and Related Spectroscopies. *J. Phys. Conf. Ser.* **430**, 012007 (2013).

8. Coker, A. K. & Sotudeh-Gharebagh, R. *Chemical Process Engineering, Volume 2: Design, Analysis, Simulation, Integration, and Problem Solving with Microsoft Excel-UniSim Software for Chemical Engineers, Heat Transfer and Integration, Process Safety, and Chemical Kinetics*. (John Wiley & Sons, 2022).

9. Hill, C. G. & Root, T. W. *Introduction to chemical engineering kinetics and reactor design*. (John Wiley & Sons, 2014).

10. Simons, J. F. M. *et al.* Structure Sensitivity of CO_2 Hydrogenation on Ni Revisited. *J. Am. Chem. Soc.* **145**, 20289-20301 (2023).

11. Wang, N. *et al.* In Situ Confinement of Ultrasmall Pd Clusters within Nanosized Silicalite-1 Zeolite for Highly Efficient Catalysis of Hydrogen Generation. *J. Am. Chem. Soc.* **138**, 7484-7487 (2016).

12. Choi, M., Wu, Z. & Iglesia, E. Mercaptosilane-assisted synthesis of metal clusters within zeolites and catalytic consequences of encapsulation. *J. Am. Chem. Soc.* **132**, 9129-9137 (2010).

13. Liu, H. *et al.* Encapsulation of Pd Single-Atom Sites in Zeolite for Highly Efficient Semihydrogenation of Alkynes. *J. Am. Chem. Soc.* **146**, 24033-24041 (2024).

14. Liu, X. *et al.* Atomically Thick Oxide Overcoating Stimulates Low-Temperature Reactive Metal-Support Interactions for Enhanced Catalysis. *J. Am. Chem. Soc.* **145**, 6702-6709 (2023).

15. Docherty, S. R. *et al.* Silica-Supported PdGa Nanoparticles: Metal Synergy for Highly Active and Selective CO_2-to-CH_3OH Hydrogenation. *JACS Au* **1**, 450-458 (2021).

16. Baumgärtner, J. F. *et al.* Metadynamics simulations reveal alloying-dealloying processes for bimetallic PdGa nanoparticles under CO_2 hydrogenation. *Chem. Sci.* **15**, 4871-4880 (2024).

17. Rane, N., Overweg, A. R., Kazansky, V. B., van Santen, R. A. & Hensen, E. J. M. Characterization and reactivity of Ga^+ and GaO^+ cations in zeolite ZSM-5. *J. Catal.* **239**, 478-485 (2006).

18. Rebenstorf, B. *et al.* IR Studies of Coordinatively Unsaturated Surface Compounds on Silica Gel. III. Adsorption of CO on Main Group Metal Ions. *Acta Chem. Scand.* **34a**, 239-248 (1980).

19. Kazansky, V. B., Subbotina, I. R., van Santen, R. A. & Hensen, E. J. M. DRIFTS study of the chemical state of modifying gallium ions in reduced Ga/ZSM-5 prepared by impregnation: I. Observation of gallium hydrides and application of CO adsorption as a molecular probe for reduced gallium ions. *J. Catal.* **227**, 263-269 (2004).

20. Garcia Sanchez, M. *Characterization of gallium-containing zeolites for catalytic applications* Phd Thesis 1 (Research TU/e / Graduation TU/e) thesis, Technische Universiteit Eindhoven, (2003).

21. Otero Areán, C., Bellan, A. L., Mentruit, M. P., Delgado, M. R. g. & Palomino, G. T. Preparation and characterization of mesoporous γ-Ga_2O_3. *Microporous Mesoporous Mater.* **40**, 35-42 (2000).

22. Serykh, A. I. & Kolesnikov, S. P. On the nature of gallium species in gallium-modified mordenite and MFI zeolites. A comparative DRIFT study of carbon monoxide adsorption and hydrogen dissociation. *Phys. Chem. Chem. Phys.* **13**, 6892-6900 (2011).

23. Kanazirev, V. *et al.* IR study of the active sites formed by H_2 treatment of Ga/HZSM-5 catalysts. *J. Mol. Catal.* **70**, 111-117 (1991).

24. Meng, L. *et al.* On the Role of Acidity in Bulk and Nanosheet [T]MFI (T=Al^{3+}, Ga^{3+}, Fe^{3+}, B^{3+}) Zeolites in the Methanol-to-Hydrocarbons Reaction. *ChemCatChem* **9**, 3942-3954 (2017).

25. Schreiber, M. W. *et al.* Lewis-Brønsted Acid Pairs in Ga/H-ZSM-5 To Catalyze Dehydrogenation of Light Alkanes. *J. Am. Chem. Soc.* **140**, 4849-4859 (2018).

26. Yuan, Y., Lee, J. S. & Lobo, R. F. Ga^+-Chabazite Zeolite: A Highly Selective Catalyst for Nonoxidative Propane Dehydrogenation. *J. Am. Chem. Soc.* **144**, 15079-15092 (2022).

27. Yuan, Y., Brady, C., Annamalai, L., Lobo, R. F. & Xu, B. Ga speciation in Ga/H-ZSM-5 by in-situ transmission FTIR spectroscopy. *J. Catal.* **393**, 60-69 (2021).

28. Faro, A. C., Jr., Rodrigues, V. d. O. & Eon, J.-G. In Situ X-ray Absorption Study of the Genesis and Nature of the Reduced Gallium Species in Ga/HZSM5 Catalysts. *J. Phys. Chem. C* **115**, 4749-4756 (2011).

29. Getsoian, A. B. *et al.* Organometallic model complexes elucidate the active gallium species in alkane dehydrogenation catalysts based on ligand effects in Ga K-edge XANES. *Catal. Sci. Technol.* **6**, 6339-6353 (2016).

30. Li, L., Chalmers, J. A., Bare, S. R., Scott, S. L. & Vila, F. D. Rigorous Oxidation State Assignments for Supported Ga-Containing Catalysts Using Theory-Informed X-ray Absorption Spectroscopy Signatures from Well-Defined Ga(I) and Ga(III) Compounds. *ACS Catal.* **13**, 6549-6561 (2023).

31. Liu, Y. *et al.* Efficient Catalytic Production of Hydrogen Peroxide Using Tin-containing Zeolite Fixed Palladium Nanoparticles with Oxidation Resistance. *Angew. Chem. Int. Ed.* **62**, e202312377 (2023).

32. Qu, J., Zhou, X., Xu, F., Gong, X.-Q. & Tsang, S. C. E. Shape Effect of Pd-Promoted Ga_2O_3 Nanocatalysts for Methanol Synthesis by CO_2 Hydrogenation. *J. Phys. Chem. C* **118**, 24452-24466 (2014).

33. Fujitani, T. *et al.* Development of an active Ga_2O_3 supported palladium catalyst for the synthesis of methanol from carbon dioxide and hydrogen. *Appl. Catal., A* **125**, L199-L202 (1995).

34. Li, L. *et al.* Ga-Pd/Ga_2O_3 Catalysts: The Role of Gallia Polymorphs, Intermetallic Compounds, and Pretreatment Conditions on Selectivity and Stability in Different Reactions. *ChemCatChem* **4**, 1764-1775 (2012).

35. Collins, S. E. *et al.* The role of Pd-Ga bimetallic particles in the bifunctional mechanism of

selective methanol synthesis via CO_2 hydrogenation on a Pd/Ga_2O_3 catalyst. *J. Catal.* **292**, 90-98 (2012).

36. Collins, S. E., Baltanás, M. A. & Bonivardi, A. L. An infrared study of the intermediates of methanol synthesis from carbon dioxide over Pd/β-Ga_2O_3. *J. Catal.* **226**, 410-421 (2004).

37. Manrique, R. *et al.* The nature of the active sites of Pd-Ga catalysts in the hydrogenation of CO_2 to methanol. *Catal. Sci. Technol.* **10**, 6644-6658 (2020).

38. Collins, S. E., Baltanás, M. A., Delgado, J. J., Borgna, A. & Bonivardi, A. L. CO_2 hydrogenation to methanol on Ga_2O_3-Pd/SiO_2 catalysts: Dual oxide-metal sites or (bi)metallic surface sites? *Catal. Today* **381**, 154-162 (2021).

39. Liao, F. *et al.* A promising low pressure methanol synthesis route from CO_2 hydrogenation over Pd@Zn core-shell catalysts. *Green Chem.* **19**, 270-280 (2017).

40. Zhang, L. *et al.* Size-dependent strong metal-support interaction in Pd/ZnO catalysts for hydrogenation of CO_2 to methanol. *Catal. Sci. Technol.* **11**, 4398-4405 (2021).

41. Zabilskiy, M. *et al.* Mechanistic Study of Carbon Dioxide Hydrogenation over Pd/ZnO-Based Catalysts: The Role of Palladium-Zinc Alloy in Selective Methanol Synthesis. *Angew. Chem. Int. Ed.* **60**, 17053-17059 (2021).

42. Kattel, S., Liu, P. & Chen, J. G. Tuning Selectivity of CO_2 Hydrogenation Reactions at the Metal/Oxide Interface. *J. Am. Chem. Soc.* **139**, 9739-9754 (2017).

43. Liu, C. *et al.* Gallium nitride catalyzed the direct hydrogenation of carbon dioxide to dimethyl ether as primary product. *Nat. Commun.* **12**, 2305 (2021).

44. Feng, W.-H. *et al.* Insights into Bimetallic Oxide Synergy during Carbon Dioxide Hydrogenation to Methanol and Dimethyl Ether over $GaZrO_x$ Oxide Catalysts. *ACS Catal.* **11**, 4704-4711 (2021).

45. Bahruji, H. *et al.* Hydrogenation of CO_2 to Dimethyl Ether over Brønsted Acidic PdZn Catalysts. *Ind. Eng. Chem. Res.* **57**, 6821-6829 (2018).

46. Ahoba-Sam, C. *et al.* On the conversion of CO_2 to value added products over composite PdZn and H-ZSM-5 catalysts: excess Zn over Pd, a compromise or a penalty? *Catal. Sci. Technol.* **10**, 4373-4385 (2020).

47. Li, W., Wang, K., Zhan, G., Huang, J. & Li, Q. Hydrogenation of CO_2 to Dimethyl Ether over Tandem Catalysts Based on Biotemplated Hierarchical ZSM-5 and Pd/ZnO. *ACS Sustainable Chem. Eng.* **8**, 14058-14070 (2020).

48. Li, L. *et al.* Facile preparation of highly efficient CuO-ZnO-ZrO_2/HZSM-5 bifunctional catalyst for one-step CO_2 hydrogenation to dimethyl ether: Influence of calcination temperature. *Chem. Eng. Res. Des.* **111**, 100-108 (2016).

49. Witoon, T., Kidkhunthod, P., Chareonpanich, M. & Limtrakul, J. Direct synthesis of dimethyl ether from CO_2 and H_2 over novel bifunctional catalysts containing CuO-ZnO-ZrO_2 catalyst admixed with WO_x/ZrO_2 catalysts. *Chem. Eng. J.* **348**, 713-722 (2018).

50. Ihm, S.-K., Baek, S.-W., Park, Y.-K. & Jeon, J.-K. in *Utilization of Greenhouse Gases* Vol. 852 *ACS Symposium Series* Ch. 12, 183-194 (American Chemical Society, 2003).

51. Frusteri, F. *et al.* Direct CO_2-to-DME hydrogenation reaction: New evidences of a superior behaviour of FER-based hybrid systems to obtain high DME yield. *J. CO2 Util.* **18**, 353-361 (2017).

52. Ereña, J., Garoña, R., Arandes, J. M., Aguayo, A. T. & Bilbao, J. Direct Synthesis of Dimethyl Ether From (H_2+CO) and (H_2+CO_2) Feeds. Effect of Feed Composition. *Int. J. Chem. Reactor Eng.* **3** (2005).

53. Naik, S. P. *et al.* Synthesis of DME from CO_2/H_2 gas mixture. *Chem. Eng. J.* **167**, 362-368

(2011).

54. Gao, W., Wang, H., Wang, Y., Guo, W. & Jia, M. Dimethyl ether synthesis from CO_2 hydrogenation on La-modified CuO-ZnO-Al_2O_3/HZSM-5 bifunctional catalysts. *J. Rare Earths* **31**, 470-476 (2013).

55. Krim, K., Sachse, A., Le Valant, A., Pouilloux, Y. & Hocine, S. One Step Dimethyl Ether (DME) Synthesis from CO_2 Hydrogenation over Hybrid Catalysts Containing Cu/ZnO/Al_2O_3 and Nano-Sized Hollow ZSM-5 Zeolites. *Catal. Lett.* **153**, 83-94 (2023).

56. Şeker, B., Dizaji, A. K., Balci, V. & Uzun, A. MCM-41-supported tungstophosphoric acid as an acid function for dimethyl ether synthesis from CO_2 hydrogenation. *Renew. Energy* **171**, 47-57 (2021).

57. Yao, L., Shen, X., Pan, Y. & Peng, Z. Unravelling Proximity-Driven Synergetic Effect within CIZO-SAPO Bifunctional Catalyst for CO_2 Hydrogenation to DME. *Energy Fuels* **34**, 8635-8643 (2020).

58. Fang, X. *et al.* A novel in situ grown Cu-ZnO-ZrO_2/HZSM-5 hybrid catalyst for CO_2 hydrogenation to liquid fuels of methanol and DME. *J. Environ. Chem. Eng.* **9**, 105299 (2021).

59. Li, H. *et al.* Selective hydrogenation of CO_2 into dimethyl ether over hydrophobic and gallium-modified copper catalysts. *Chin. J. Catal.* **54**, 178-187 (2023).

60. Wang, X. *et al.* Catalytic activity for direct CO_2 hydrogenation to dimethyl ether with different proximity of bifunctional Cu-ZnO-Al_2O_3 and ferrierite. *Appl. Catal., B* **327**, 122456 (2023).

61. Tan, K. B. *et al.* Green synthesis of microspherical-confined nano-Pd/In_2O_3 integrated with H-ZSM-5 as bifunctional catalyst for CO_2 hydrogenation into dimethyl ether: A carbonized alginate templating strategy. *Sep. Purif. Technol.* **297**, 121559 (2022).

62. Movick, W. J., Yun, G.-N., Tyrone Ghampson, I. & Ted Oyama, S. The Delplot kinetic method applied to systems with adsorbates: Hydrodeoxygenation of benzofuran on a bimetallic CoPd phosphide catalyst supported on KUSY. *J. Catal.* **404**, 786-801 (2021).

63. Silva Sousa, L. d. *et al.* Identification of Transient Intermediates and Active Species in Atomic CZA Catalysts for CO_2 Hydrogenation to Methanol. *J. Am. Chem. Soc.* (2025).

64. Cored, J. *et al.* Enhanced Methanol Production over Non-promoted Cu-MgO-Al_2O_3 Materials with Ex-solved 2 nm Cu Particles: Insights from an Operando Spectroscopic Study. *ACS Catal.* **12**, 3845-3857 (2022).

65. Camp Jr, C. H. PyMCR: A python library for multivariatecurve resolution analysis with alternating regression (MCR-AR). *J. Res. Nat. Inst. Stand. Technol.* **124**, 1 (2019).

66. Lawton, W. H. & Sylvestre, E. A. Self Modeling Curve Resolution. *Technometrics* **13**, 617-633 (1971).

67. Jaumot, J., Gargallo, R., de Juan, A. & Tauler, R. A graphical user-friendly interface for MCR-ALS: a new tool for multivariate curve resolution in MATLAB. *Chemom. Intell. Lab. Syst.* **76**, 101-110 (2005).

68. Felten, J. *et al.* Vibrational spectroscopic image analysis of biological material using multivariate curve resolution–alternating least squares (MCR-ALS). *Nat. Protoc.* **10**, 217-240 (2015).

69. Calvin, S. *et al.* Determination of crystallite size in a magnetic nanocomposite using extended x-ray absorption fine structure. *J. Appl. Phys.* **94**, 778-783 (2003).

70. Luo, Y. *et al.* Anchoring IrPdAu Nanoparticles on NH_2-SBA-15 for Fast Hydrogen Production from Formic Acid at Room Temperature. *ACS Appl. Mater. Interfaces* **12**, 8082-

8090 (2020).
